# Comparative efficacy of 24 exercise types on postural instability in adults with Parkinson’s disease: a systematic review and network meta-analysis

**DOI:** 10.1186/s12877-023-04239-9

**Published:** 2023-08-28

**Authors:** Yujia Qian, Xueying Fu, Haoyang Zhang, Yong Yang, Guotuan Wang

**Affiliations:** 1 School of Sports Science and Physical Education, Nanjing Normal University, Nanjing, China; 2 Laboratory of Kinesiology and Rehabilitation, School of Physical Education and Sport, Henan University, 85 Minglun Rd, Shunhe District, Kaifeng City, 475001 China; 3 Laboratory of Kinesiology and Rehabilitation, School of Physical Education and Sport, Chaohu University, No. 1 Xuefu Road, Chaohu Economic Development Zone, Hefei, Anhui Province 238000 China; 4 School of Physical Education and Health, Krasnoyarsk State Pedagogical University named after V.P. Astafyev Street. Ada Lebedeva, House 89, Krasnoyarsk City, 660049 Russia

**Keywords:** Parkinson disease, Exercise, Meta-analysis, Physiotherapy

## Abstract

**Objective:**

To compare, rank and evaluate the 24 exercise types that improve postural instability in patients with Parkinson’s disease (PD).

**Methods:**

We searched the data in PubMed, MEDLINE, Embase, PsycINFO, Cochrane library, and Web of Science from their inception date to January 23, 2023. Randomized controlled trials (RCTs) that aimed at determining the effectiveness of physical activity interventions on postural instability in adults with PD. This review focused on different balance outcome categories: (a) balance test batteries (BBS); (b) static steady-state balance (sSSB); (c) dynamic steady-state balance (dSSB); (d) proactive balance (PB); (e) reactive balance (RB).

**Results:**

Among 10,474 records, 199 studies (patients = 9523) were eligible for qualitative synthesis. The random-effects NMA model revealed that the following exercise training modalities had the highest p score of being best when compared with control group: body-weight support treadmill training (BWS_TT) for BBS (p score = 0.97; pooled standardised mean difference (95% CI): 1.56 (0.72 to 2.39)) and dSSB (1.00; 1.53 (1.07 to 2.00)), aquatic exercise (AQE) for sSSB (0.85; 0.94 (0.33 to 1.54)), Pilates for PB (0.95; 1.42 (0.59 to 2.26)). Balance and gait training with the external cue or attention (BGT_ECA) and robotic assisted gait balance (RA_GT) had similar superior effects in improving RB. The confidence in evidence was often low according to Confidence in Network Meta-Analysis.

**Conclusions:**

There is low quality evidence that BWS_TT, AQE, Pilates, BGT_ECA and RA_GT are possibly the most effective treatments, pending outcome of interest, for adults with PD.

**Supplementary Information:**

The online version contains supplementary material available at 10.1186/s12877-023-04239-9.

## Introduction

Parkinson’s disease (PD) is characterized by progressive postural instability manifested by impairment of postural control systems, including biomechanical limitations, limits of stability and perception of verticality, anticipatory postural adjustments, postural responses, sensorimotor integration, and dynamic control of gait [1]. At its core, PD is a neurodegenerative disease with early prominent death of dopaminergic neurons in the substantia nigra pars compacta (SNpc) [2]. The mainstay of Parkinson’s disease management is symptomatic treatment with drugs that increase dopamine concentrations or directly stimulate dopamine receptors [3, 4]. However, even with optimal drug treatments, PD still experiences movement disorders in balance and gait, often leads to falls, and serious potential complications [5–7].

Previous pairwise meta-analyses have shown that exercises are effective treatment for balance in Parkinson’s disease [8–10]. Recent studies have often attempted to compare the differences between exercise types in improving balance in PD patients through head-to-head randomized controlled trials to explore the preferred exercise type. For example, the results of Arcolin et al. [11] showed that 3 weeks of aerobic exercise and treadmill training performed similar effects in improving balance in PD patients. The results of de Melo et al. [12] showed that 4-week VR gait training and traditional treadmill training both effectively improved the balance ability of PD patients. Atan and colleagues conducted a 6-week exercise program for PD patients, and found that body weight support treadmill training was more effective than traditional treadmill training in improving balance [13]. In addition, robot-assisted gait training also showed better advantages compared to traditional treadmill training [14]. The network meta-analysis results of Tang et al. [15] showed that among six exercise modalities (Dance, Qigong, Tango, resistance training, Tai Chi, and yoga), Tango was the most effective in improving balance in PD patients. However, the results of a 1 randomized controlled trial showed that treadmill training (12 weeks) alone significantly improved balance in PD patients, with no apparent benefit in the Tango training group [16]. Consequently, there remains a lack of pooled evidence on the relative effects of more different exercise interventions on balance. In addition, traditional pairwise meta-analyses are limited to head-to-head comparisons of two different interventions, potentially leading to the exclusion of many RCTs of exercise interventions that met the inclusion criteria. To tackle this problem, a network meta-analysis is well suited, because it facilitates comparisons of multiple pairs of interventions in one statistical model [17].

A recent network meta-analysis pooling 113 RCTs assessed the effects of 18 exercise types on total balance in Parkinson’s disease patients [18]. However, balance control is highly task-specific, in previous studies, balance control has been divided into static/dynamic steady-state balance, proactive balance, reactive balance, and total balance ability [19, 20], and which were poorly correlated [21, 22]. How to comprehensively improve the balance ability of Parkinson’s disease patients through targeted exercise is still unclear. Therefore, in this study, we set out to conduct a network meta-analysis to comprehensively and systematically compare the effects of different exercise types on improving each specific balance ability in PD patients.

## Methods

This review was conducted in accordance with Preferred Reporting Items for Systematic Reviews and Meta-Analyses for Network Meta-Analyses (PRISMA-NMA) [23]. In addition, this study has been registered with PROSPERO, under the number CRD42021220052.

### Searches strategy

We searched the data in PubMed, MEDLINE, Embase, PsycINFO, Cochrane Central Register of Controlled Trials (CENTRAL), and Web of Science from their inception date to January 23, 2023 using Medical Subject Headings (MeSH) for ‘Parkinson disease’ and ‘exercise’ search terms in Additional file 1: Appendix 1. Additional searches included reviewing the reference lists of previously published systematic reviews identified via the Cochrane Database of Systematic Reviews (search terms: Parkinson disease, exercise; limits: none) and PubMed (search terms: Parkinson disease, exercise; limits: systematic reviews or meta-analysis).

### Inclusion and exclusion criteria

The inclusion criteria were based on the PICOS (participants, interventions, comparators, outcomes, and study design) approach [23]. To be eligible for inclusion, studies had to meet the following criteria and report specific experimental characteristics: (a) participants were diagnosed as PD, the mean age ≥ 50 years, Hoehn and Yahr stages < 4; (b) the physical activities and exercises were divided into 24 types according to their content. The specific type of exercise training was determined by the group names chosen by authors and the definitions presented in Additional file 1: Appendix 2; (c) the non-exercise training treatment group included health education and usual care. Besides, for head-to-head studies, the comparator may be any of the 24 physical activity types; (d) the study tested at least one behavioral balance outcome (e.g., static/dynamic steady-state balance, proactive balance, reactive balance, and total balance ability [19, 20, 24]); (e) In the study design, we included published and unpublished RCTs (individual design, cluster design, or the first half of crossover) and compared an exercise training intervention with a non-exercise training intervention or another exercise training intervention for network meta-analysis. Studies with the following features were excluded: (a) non-randomized design; (b) used fewer than six sessions (i.e., acute studies); (c) unavailability of means and standard deviations in the results or if authors did not reply to our request for data; (d) studies that did not clearly describe the types of exercises and the duration of training. Based on the defined inclusion and exclusion criteria, two independent reviewers (XYF, HYZ) screened potentially relevant papers by analyzing titles, abstracts, and full texts of respective articles to elucidate their eligibility.

### Data extraction

Relevant publication information (ie, author, title, year, journal), number of patients, patient characteristics (eg, age and sex), interventions considered (Table 1) and outcome measures (ie, balance test batteries, static/dynamic steady-state balance, proactive balance reactive balance) were extracted by two independent assessors. In the process of extracting data, if the original study reported a standard error in the experimental and control groups, the standard deviation was calculated by the formula: standard deviation (SD) = standard error (SE) × √n. If both are missing, we will estimate SD based on the confidence interval, t-value, quartile, range, or *p*-values as described in section 7.7.3 of the Cochrane Handbook for Systematic Reviews. When only figures were presented, data were extracted using GetData (http://getdata-graph-digitizer.com) to measure the length (in pixels) of the axes to calibrate and then the length in pixels from the relevant axis to the data points of interest. If the data needed for the study cannot be extracted from the above methods, we will ask the authors about the data at least 4 times within 6 weeks.

**Table 1:** Characteristics of the included studies

**Exercise type**	**Years covered**	**N(s)**	**n(s)**	**Male(%)**	**Age(m)**	**Duration ill in years(m)**	**Hoehn and Yahr stage(m)**	**Exercise duration(m)**	**Exercise frequency(m)**	**Course time(m)**
Total	1996–2020	199	9523	60.54	67.54	6.84	2.37	12.34	3.10	54.16
AE	2009–2019	17	356	58.50	66.10	4.97	2.32	11.67	3.29	49.44
AQE	2011–2020	15	210	66.84	67.10	5.62	2.48	7.76	3.33	54.06
BGT	1996–2020	37	607	62.78	69.49	6.68	2.43	9.10	2.84	48.42
BGT_ECA	1996–2020	24	553	57.30	68.51	7.57	2.52	5.82	3.43	45.14
BGT_ICA	2015–2017	2	31	76.36	71.65	6.7	2.75	7.50	3.00	52.50
BWS_TT	2002–2019	5	61	51.36	66.49	5.68	2.58	6.00	4.00	37.50
CPP	1998–2020	29	506	56.39	67.10	6.81	2.50	16.81	3.67	61.92
Dance	2013–2019	9	125	50.97	68.29	7.17	2.27	11.40	1.80	62.22
DT_BGT	2008–2020	27	753	62.89	68.48	6.99	2.43	6.88	3.08	54.04
Mul_C	2003–2020	33	975	58.93	67.59	6.67	2.33	14.59	3.33	60.51
Mul_D	2012–2020	13	346	70.01	68.05	7.21	2.34	11.79	3.13	62.69
NW	2010–2018	5	69	62.02	64.24	5.45	2.08	7.50	3.50	60.00
Pilates	2013–2017	3	28	45.9	59.0	6.8	2.4	9.33	3.67	55.00
PT	2014–2020	3	49	58.66	70.57	7.73	2.17	10.00	2.50	56.25
Qigong	2013–2017	5	145	42.56	67.12	7.33	2.13	12.88	3.63	56.25
RA_GT	2012–2019	7	171	54.40	69.71	8.17	2.78	4.00	4.00	43.33
RT	2007–2020	34	687	64.51	66.06	7.69	2.36	24.48	2.32	59.96
Stretch	2010–2020	11	291	61.61	67.24	6.13	2.38	18.08	2.64	56.69
Tai Chi	2008–2020	9	198	65.31	66.32	6.83	2.25	14.62	2.31	56.54
Tango	2007–2018	8	106	65.45	68.45	6.21	2.21	15.10	2.00	56.67
TT	2007–2020	31	587	57.567	66.457	6.897	2.367	8.20	3.44	41.82
VR	2011–2020	20	411	64.29	67.54	7.32	2.20	7.30	3.41	44.43
WBV	2008	1	10	70	72.5	7	-	6.33	5.33	73.33
Yoga	2016–2020	6	136	65.09	67.22	6.55	2.28	11.50	3.13	48.57

*N* number of studies,* n* number of sample size, *s* sum, *m* mean, *AE* Aerobic Exercise, *AQE* Aquatic Exercise, *BGT* Balance and Gait Training, *BGT_ECA* Balance and Gait Training with External Cue or Attention, *BWS_TT* Body Weight Support Treadmill Training, *CON* Control group, *CPP* Classic Physiotherapy Program, *DT_BGT* Dual Task Balance and Gait Training, *Mul_C* Multicomponent Exercise Program, *Mul_D* Multidisciplinary Exercise Program, *RA_GT* Robotic Assisted Gait Training, *RT* Resistance Training, *TC* Tai Chi, *TT* Treadmill Training, *VR* Virtual Reality, *WBV* Whole Body Vibratio

### Risk of bias assessment and CINeMA

Risk of bias in RCTs for each individual study was assessed independently using the Cochrane Collaboration’s risk of bias tool (Additional file 1: Appendix 5) [25], which examined potential selection bias (random sequence generation and allocation concealment), performance bias (blinding of patients and personnel), detection bias (blinding of outcome assessment), attrition bias (incomplete outcome data), reporting bias (selective outcome reporting) and other bias. For each source of bias, studies were classified as having a low, high or unclear (if reporting was not sufficient to assess a particular domain) risk. The overall risk of bias was classified into high, moderate, or low as proposed in a large network meta-analysis for antidepressants [26]. The certainty of evidence produced by the synthesis for each outcome was evaluated using the framework described by Salanti and colleagues [27] and implemented using the CINeMA (Confidence in Network Meta- Analysis) web application which allows confidence in the results to be graded as high, moderate, low, and very low [28].

### Statistical analysis

We used R software netmeta package (version 3.6.3) to perform a network meta-analysis combining direct and indirect comparisons in the Frequentist model [29, 30]. The effect sizes measure chose the standardized mean difference (SMD), because the studies used different rating scales or units of balance outcomes, and a random-effects network meta-analysis model was used. In addition, league table presented the summary SMD, 95% credible intervals (CrIs) for all pairwise comparisons, and the results of comparing the outcomes of each exercise intervention group and the control group in the form of a forest plot. We used P-scores to rank exercise types on the basis of the degree of improvement in each balance outcome categories [31]. P-scores ranged from 0 to 1, a higher P-score indicating a greater degree of improvement in balance ability. The transitivity assumption was evaluated by comparing the distribution of potential effect modifiers (publication year, sample size, mean age, percentage male, years of diagnosis, and disease grade) (Additional file 1: Appendix 3) across studies grouped before analyzing the results. I^2^ is a parameter for quantitative analysis of the heterogeneity between the results of each study, and when I^2^ > 50%, it means that there is substantial heterogeneity. We used global [32] and local methods [33] to test the inconsistency of the research results. The potential reasons of heterogeneity (publish year, sample size, mean age, percentage male, years of diagnosis, disease grade, exercise duration, exercise frequency, the total time of single session, and outcomes test ON/OFF dopaminergic medication) was explored by network meta-regression with R gemtc package. We compared the adjusted funnel plot to assess the risk of publication bias under specific circumstances, and Egger’s test suggestive of publication bias when *p* < 0.05. We assessed the sensitivity of our findings by repeating each network meta-analysis after excluding studies, such as studies with an overall high risk of bias (Additional file 1: Appendix 8).

## Results

**Fig. 1:**
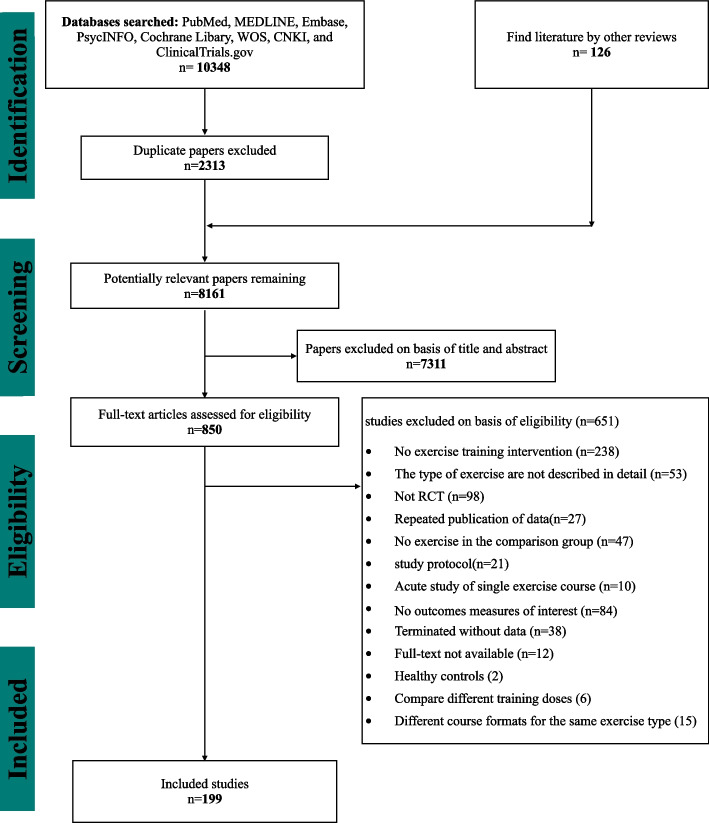
PRISMA Flow diagram of the search process for studies. *RCT* randomized controlled trials

**Fig. 2:**
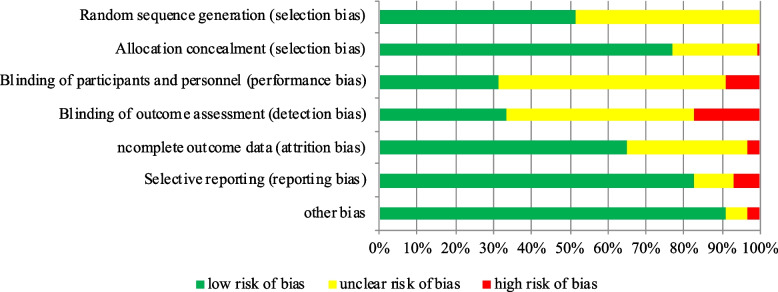
Summary of the risk of bias assessment in the individual domains of the included studies

The flow diagram of the search process for studies of the systematic review is presented in Fig. 1. After excluding 7311 reports based on titles and abstracts, 850 full-text articles were retrieved. Three pairs of investigators confirmed the outcomes of interest by viewing the full text, and finally included 199 studies with 9523 participants. 5765 (60.54%) participants were male and 3758 (39.46%) female. Sample size ranged from 4 to 238 (mean age, 67.54), and mean illness duration was 6.84 years, the mean of Hoehn and Yahr stage was 2.37 (Table 1). Among the 199 studies included (Additional file 1: Appendix 4), we divided them into 24 exercise types according to the exercise content (Additional file 1: Appendix 2). The exercise period ranged from 2 to 96 weeks (mean period, 12.34 weeks), the frequency of exercise training per week ranged from 1–12 (mean frequency, 3.1), and the total time of single session ranged from 15 to 150 min (mean time, 54.16 min). The risk of bias assessment for each individual study is presented in online Additional file 1: Appendix 5 and summary data in Fig. 2. Overall, the percentage of studies with low risk of bias for the random sequence generation was 57.76%, the allocation concealment was 76.88%, the blinding of participants and personnel was 31.16%, the blinding of outcome assessment was 33.17%, the uncomplete outcome data was 64.82%, the selective reporting was 82.91%, and the other bias was 90.95%.

### Balance test batteries (overall balance ability)

**Fig. 3:**
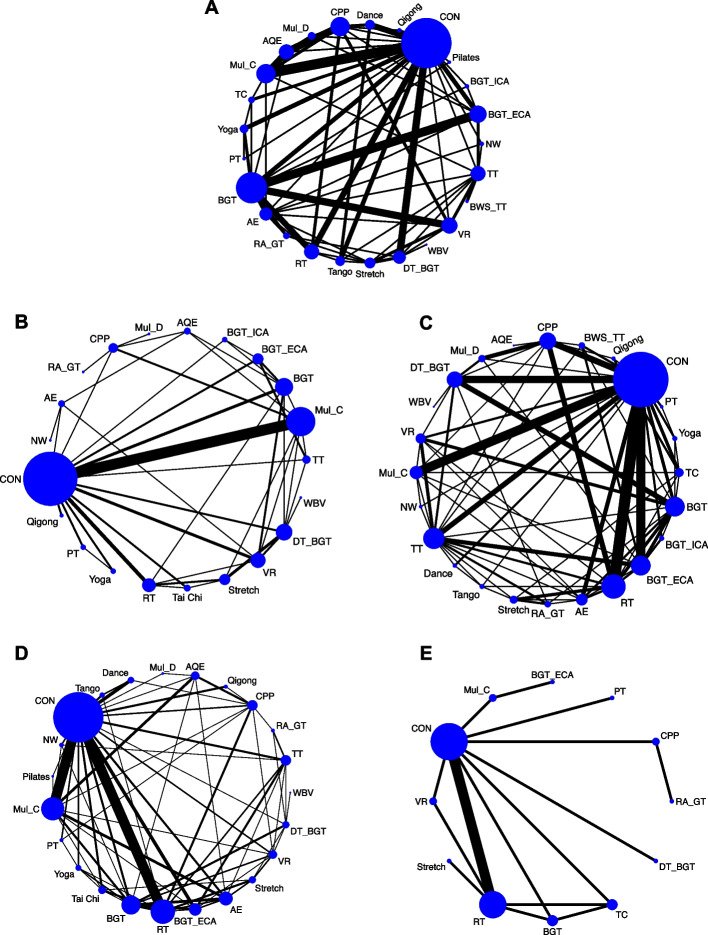
Network plot of balance outcomes. **A** Overall balance; **B** Static steady−state balance; **C** Dynamic steady−state balance; **D** Proactive balance; **E** Reactive balance. The size of the nodes corresponds to the number of participants randomized to each Exercise type. Exercise type with direct comparisons are linked with a line; its thickness corresponds to the number of trials evaluating the comparison. *AE* Aerobic Exercise, *AQE* Aquatic Exercise, *BGT* Balance and Gait Training, *BGT_ECA* Balance and Gait Training with External Cue or Attention, *BGT_ICA* Balance and Gait Training with Internal Cue or Attention, *BWS_TT* Body Weight Support Treadmill Training, *CON* Control group, *CPP* Classic Physiotherapy Program, *DT_BGT* Dual Task Balance and Gait Training, *Mul_C* Multicomponent Exercise Program, *Mul_D* Multidisciplinary Exercise Program, *NW* Nordic Walking, *PT* Power Training, *RA_GT* Robotic Assisted Gait Training, *RT* Resistance Training, *TC* Tai Chi, *TT* Treadmill Training, *VR* Virtual Reality, *WBV* Whole Body Vibration

**Fig. 4:**
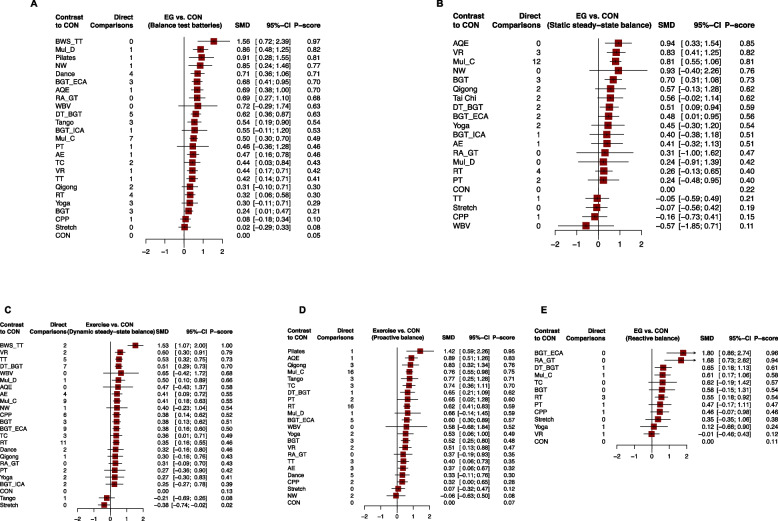
Forest plot change in efficacy of balance outcomes. **A** Overall balance; **B** Static steady−state balance; **C** Dynamic steady−state balance; **D** Proactive balance; **E** Reactive balance. Exercise type are ranked according to the surface under the curved cumulative ranking probabilities. Treatments crossing the y-axis are not significantly different from CON. *SMD* standardized Mean Difference, *CrI* Credible Interval, *AE* Aerobic Exercise, *AQE* Aquatic Exercise, *BGT* Balance and Gait Training, *BGT_ECA* Balance and Gait Training with External Cue or Attention, *BGT_ICA* Balance and Gait Training with Internal Cue or Attention, *BWS_TT* Body Weight Support Treadmill Training, *CON* Control group, *CPP* Classic Physiotherapy Program, *DT_BGT* Dual Task Balance and Gait Training, *Mul_C* Multicomponent Exercise Program, *Mul_D* Multidisciplinary Exercise Program, *NW* Nordic Walking, *PT* Power Training, *RA_GT* Robotic Assisted Gait Training, *RT* Resistance Training, *TC* Tai Chi, *TT* Treadmill Training, *VR* Virtual Reality, *WBV* Whole Body Vibration

One hundred and four studies (52.26%) with 4536 (47.63%) assessed overall balance ability and were eligible for NMA (Fig. 3A). Compared with the control group (CON), 17 (71%) exercise types significantly improved the overall balance ability, and the SMDs (95% CrI) ranged between 1.56 (0.72; 2.39) for BWS_TT to 0.24 (0.01; 0.47) for balance and gait training (BGT). Ranking on the basis of degree of associated overall balance ability alteration defined BWS_TT as the best and Stretch the worst (Fig. 4A). NMA results showed that BWS_TT, multidisciplinary exercise program (Mul_D), Pilates, Dance, aquatic exercise (AQE), Balance and gait training with the external cue or attention (BGT_ECA), RA_GT, and dual task balance and gait training (DT_BGT) significantly more than many other exercise types (more than 2) (Additional file 1: Appendix 6).

### Static steady-state balance

Fifty one studies (25.63%) with 2155 (22.63%) showed available results for change of static steady-state balance (Fig. 3B). 6 out of 20 exercise types significantly improved the static steady-state balance compared to the CON with the SMDs (95%CrI) ranged from between 0.94 (0.33; 1.54) for AQE to 0.48 (0.01; 0.95) for BGT_ECA. Ranking on the basis of degree of associated static steady-state balance alteration defined AQE as the best and whole body vibration (WBV) the worst (Fig. 4B). NMA results showed that AQE, VR, multicomponent exercise program (Mul_C), Dance, and BGT significantly more than many other exercise types (more than 2) (Additional file 1: Appendix 6).

### Dynamic steady-state balance

For change in dynamic steady-state balance, one hundred and ten studies with 4673 (49.07%) compared 23 different exercise types with CON (Fig. 3C). Compared with CON, we found 12 exercise types (52.17) significantly improved the dynamic steady-state balance, with the SMDs (95%CrI) ranged from 1.53 (1.07; 2.00) for BWS_TT to 0.35 (0.16; 0.55) for RT. Ranking on the basis of degree of associated dynamic steady-state balance alteration defined BWS_TT as the best and Stretch the worst (Fig. 4C). NMA results showed that only BWS_TT significantly more than many other exercise types (more than 2) (Additional file 1: Appendix 6).

### Proactive balance

One hundred and eleven studies (59.80%) with 5157 (54.15%) showed available results for change of proactive balance (Fig. 3D). 16 out of 23 exercise types significantly improved the proactive balance compared to the CON with the SMDs (95%CrI) ranged from between 1.42 (0.59; 2.26) for Pilates to 0.32 (0.00; 0.665) for classic physiotherapy program (CPP). Ranking on the basis of degree of associated proactive balance alteration defined Pilates as the best and Nordic walking (NW) the worst (Fig. 4D). NMA results showed that Pilates, AQE, and Mul_C significantly more than many other exercise types (more than 2) (Additional file 1: Appendix 6).

### Reactive balance

Thirteen studies (6.53%) with 540 (5.67%) showed available results for change of reactive balance (Fig. 3E). 5 out of 13 exercise types significantly improved the reactive balance compared to the CON with the SMDs (95%CrI) ranged from between 1.80 (0.86; 2.74) for BGT_ECA to 0.32 (0.18; 0.92) for RT. Ranking on the basis of degree of associated reactive balance alteration defined BGT_ECA as the best and VR the worst (Fig. 4E). NMA results showed that BGT_ECA and RA_GT significantly more than many other exercise types (more than 2), and most differences between the remaining exercise types were small or very uncertain (Additional file 1: Appendix 6).

### Heterogeneity and certainty of evidence

For heterogeneity, most outcomes were moderate. Additionally, the results of design-by-treatment interaction test showed that global inconsistency was not significant for all outcomes. The SIDE test of all outcomes showed that the percentage of comparisons with evidence of inconsistency ranged from 0–9.6% (Table 2). Overall, we found no evidence of important heterogeneity or inconsistency in the NMA. Moreover, for all outcomes, the certainty of the evidence was low overall (Additional file 1: Appendix 9).

**Table 2:** Evaluation of heterogeneity and inconsistency

**Outcomes**	**Number of studies**	**Heterogeneity**						**SIDE splitting**		**The Design-by-Treatment test**			
		τ^2^	Q	df	*P*	I^2^	Heterogeneity assessment	Number of inconsistent comparisons out of total	Percentage of inconsistent comparisons out of total	Q	df	τ^2^	*p*-value
Balance test batteries	104	0.0584	153.86	98	0.0003	36.2%	moderate	10	9.6%	46.61	50	0.0595	0.6104
Static steady-state balance	51	0.1542	112.19	49	< 0.0001	56.3%	moderate to high	1	2.0%	17.75	27	0.2381	0.9108
Dynamic steady-state balance	110	0.0958	238.07	124	< 0.0001	47.9%	moderate	6	5.5%	65.51	68	0.1086	0.5631
Proactive balance	119	0.1717	332.11	127	< 0.0001	61.8%	moderate to high	7	5.9%	66.52	62	0.2026	0.3243
Reactive balance	13	0.0037	3.1	3	0.3768	3.2%	low	0	0%	3.01	1	0	0.0825

### Meta-regression and sensitivity analysis

**Fig. 5:**
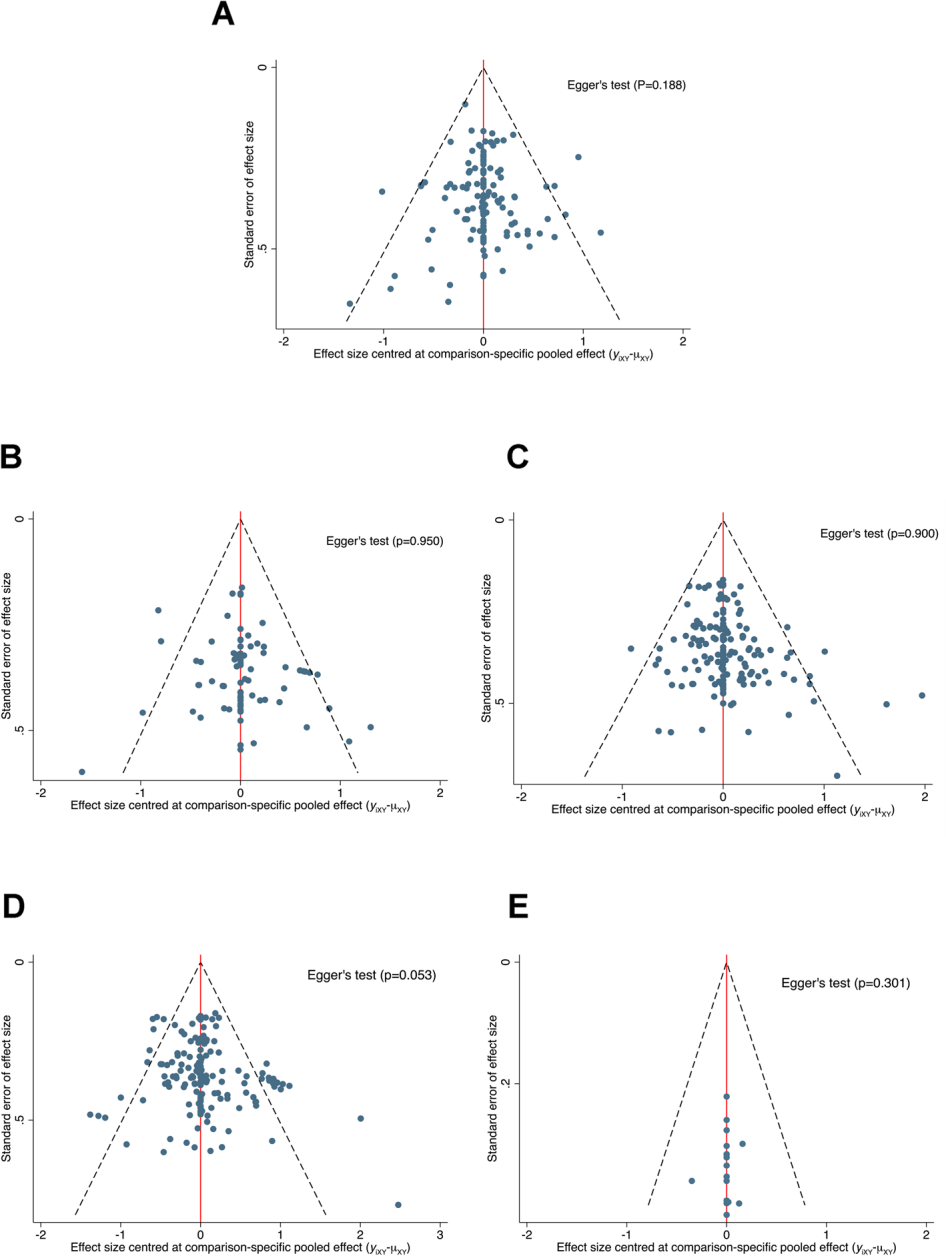
Comparison adjusted funnel plots for balance outcomes. **A** Overall balance; **B** Static steady−state balance; **C** Dynamic steady−state balance; **D** Proactive balance; **E** Reactive balance

The potential threats of the baseline characteristics, training dose, and the ON/OFF state of outcomes test of the included studies to the transitivity assumption and the source of heterogeneity were resolved through meta-regression and sensitivity analysis for all outcomes (Table 3). Sensitivity analyses removing studies with overall high risk of bias, exercise duration less than 4 weeks and more than 24 weeks, exercise frequency less than 2 and more than 4, OFF state during the test, and data were extracted using GetData and estimated standard deviations value did not affect the results (Additional file 1: Appendix 8). In addition, our comparison-adjusted funnel plot had good symmetry for all outcomes, and the results of Egger’s test showed that no small study effect was found (Fig. 5). Thus, overall, we concluded that the stability of our findings were not a source of concern in this NMA.

**Table 3:** Network meta-regression

**Covariate**	**Shared beta** **(median and 95% CrI)**	**Heterogeneity** **τ (median and 95% CrI)**	**% of variance explained**
Balance test batteries			
None	-	0.26 (0.18; 0.35)	-
Publish Year	-0.05 (-0.30; 0.20)	0.26 (0.18; 0.35)	0%
Mean Age	-0.27(-0.54; 0.02)	0.24 (0.16; 0.34)	-7.7%
Years of Diagnosis	-0.04 (-0.43; 0.36)	0.26 (0.18; 0.35)	0%
Hoehn and Yahr stage	0.35 (-0.07; 0.80)	0.26 (0.18; 0.36)	0%
Sample Size	-0.12 (-0.26; 0.02)	0.25 (0.17; 0.34)	-3.8%
Percentage Male	-0.17 (-0.45; 0.10)	0.26 (0.18; 0.35)	0%
Exercise Period	-0.14 (-0.59; 0.29)	0.26 (0.18; 0.35)	0%
Exercise Frequency	-0.27 (-0.55; 0.01)	0.25 (0.17; 0.34)	-3.8%
Time of single session	0.20 (-0.08; 0.47)	0.25 (0.17; 0.34)	-3.8%
ON/OFF	0.13 (-0.42; 0.67)	0.26 (0.18; 0.36)	0%
Static steady-state balance			
None	-	0.43 (0.30; 0.60)	-
Publish Year	0.34 (-0.09; 0.78)	0.42 (0.29; 0.58)	-2.3%
Mean Age	-0.13(-0.62; 0.25)	0.43 (0.30; 0.60)	0%
Years of Diagnosis	-0.33 (-0.82; 0.14)	0.42 (0.29; 0.59)	-2.3%
Hoehn and Yahr stage	-0.07 (-0.60; 0.47)	0.44 (0.31; 0.61)	0%
Sample Size	-0.23 (-0.59; 0.13)	0.42 (0.29; 0.60)	-2.3%
Percentage Male	-0.50 (-0.96; 0.01)	0.41 (0.27; 0.57)	-4.7%
Exercise Period	-0.46 (-0.94; 0.02)	0.41 (0.28; 0.58)	-4.7%
Exercise Frequency	-0.37 (-0.06; 0.79)	0.41 (0.27; 0.57)	-4.7%
Time of single session	0.42 (-0.04; 0.89)	0.42 (0.28; 0.58)	-2.3%
ON/OFF	0.88 (-0.07; 1.80)	0.41 (0.27; 0.57)	-4.7%
Dynamic steady-state balance			
None	-	0.34 (0.26; 0.43)	-
Publish Year	-0.01 (-0.24; 0.20)	0.34 (0.25; 0.43)	0%
Mean Age	-0.18 (-0.42; 0.07)	0.33 (0.25; 0.43)	-2.9%
Years of Diagnosis	0.38 (0.07; 0.69)^a^	0.33 (0.25; 0.42)	-2.9%
Hoehn and Yahr stage	-0.08 (-0.34; 0.18)	0.34 (0.26; 0.43)	0%
Sample Size	-0.10 (-0.31; 0.10)	0.34 (0.26; 0.43)	0%
Percentage Male	-0.18 (-0.07; 0.45)	0.34 (0.26; 0.44)	0%
Exercise Period	0.02 (-0.17; 0.23)	0.34 (0.26; 0.44)	0%
Exercise Frequency	-0.24 (-0.64; 0.13)	0.34 (0.26; 0.44)	0%
Time of single session	-0.16 (-0.39; 0.05)	0.34 (0.26; 0.43)	0%
ON/OFF	0.22 (-0.23; 0.68)	0.34 (0.26; 0.44)	0%
Proactive balance			
None	-	0.45 (0.36; 0.55)	-
Publish Year	0.25 (-0.02; 0.50)	0.45 (0.36; 0.55)	0%
Mean Age	-0.16(-0.47; 0.15)	0.45 (0.36; 0.55)	0%
Years of Diagnosis	0.16 (-0.16; 0.49)	0.45 (0.36; 0.56)	0%
Hoehn and Yahr stage	0.21 (-0.14; 0.54)	0.45 (0.36; 0.55)	0%
Sample Size	-0.39 (-0.63; -0.16)^a^	0.42 (0.32; 0.52)	-6.7%
Percentage Male	-0.13 (-0.41; 0.17)	0.45 (0.36; 0.55)	0%
Exercise Period	0.35 (0.08; 0.61)^a^	0.43 (0.35; 0.54)	-4.4%
Exercise Frequency	0.89 (-0.53; 1.26)	0.38 (0.30; 0.48)	-15.6%
Time of single session	0.01 (-0.29; 0.29)	0.45 (0.37; 0.56)	0%
ON/OFF	0.92 (-0.25; 2.18)	0.44 (0.35; 0.55)	-2.2%
Reactive balance			
None	-	0.32 (0.01; 1.06)	-
Publish Year	0.09 (-4.81; 3.75)	0.35 (0.02; 1.01)	9.4%
Mean Age	0.03 (-1.08; 1.11)	0.41 (0.01; 1.13)	28.1%
Years of Diagnosis	-0.09 (-0.91; 0.70)	0.39 (0.02; 1.13)	21.8%
Hoehn and Yahr stage	0.72 (-2.34; 5.77)	0.29 (0.01; 1.06)	-9.4%
Sample Size	0.47 (-0.88; 1.78)	0.31 (0.02; 1.09)	-3.1%
Percentage Male	-0.34 (-1.46; 0.88)	0.33 (0.02; 1.11)	3.1%
Exercise Period	0.12 (-1.81; 2.05)	0.39 (0.02; 1.11)	21.8%
Exercise Frequency	0.80 (-1.63; 4.24)	0.27 (0.02; 1.04)	15.6%
Time of single session	-0.61 (-2.05; 0.74)	0.25 (0.01; 1.06)	-21.9%
ON/OFF	-0.02 (-3.78; 4.08)	0.33 (0.02; 1.09)	3.1%

*CrI* Credible Interval

^a^Significant influence factors, 95% CrI does not contain zero

## Discussion

This is the first systematic review and network meta-analysis that examined the effects of 24 exercise types on measures of balance in Parkinson’s disease. The main finding of our analyses supported many exercise programs as effective means of improving balance in PD. In addition, we have found (1) 17 exercise types significantly improved the overall balance ability, and BWS_TT showed the large effects; (2) BWS_TT showed the best effective for improvements in dynamic steady-state balance, and that only BWS_TT is significantly more than many other exercise types (more than 2); (3) 6 exercise types significantly improved the static steady-state balance compared to the CON, and AQE ranks the best according to the p-score; (4) Pilates showed the best effective for improvements in proactive balance, and significantly more than many other exercise types; (5) BGT_ECA and RA_GT showed similarly large effect in reactive balance compared to CON, and significantly more than many other exercise types.

Our findings showed that exercises are an effective treatment for improving balance in Parkinson’s patients, as had been confirmed in numerous previous studies [9, 34–36]. Postural instability is a prominent movement disability in people with PD [37]. Some specific parameters of exercise, such as exercise intensity, repetition, specificity, difficulty and complexity, can effectively improve neuroplasticity in PD patients. On the other hand, motor performance requires cognitive engagement, which can be enhanced by feedback, cues, or the attentional demands of dual-tasking, as well as motivation. Therefore, designing or implementing exercises to improve specific motor skills, such as balance, can ultimately improve postural instability symptoms in PD patients [38]. That had been demonstrated in previous studies such as treadmill training (e.g., with [13, 39] or without body support [40, 41]), balance training (e.g., dual task balance training [42, 43], balance training with attention and cues [44, 45]), Tai Chi [46, 47], Tango [48, 49], and exercises during the environment of virtual reality [50, 51]. Therefore, these may be the reasons why the many types of exercise in this study significantly improved the balance ability of Parkinson’s disease patients.

It is unlikely that one kind of exercise training is the single best approach to treating Parkinson disease. Our study provides evidence that 17 (71%) exercise types significantly improved the overall balance ability compared to CON, and BWS_TT, Mul_D, Pilates, Dance, AQE, BGT_ECA, RA_GT, and DT_BGT significantly more than many other exercise types (more than 2). In addition, our NMA for improving overall balance ability identified BWS_TT, Mul_D, Pilates and NW as the four treatments most likely to be the best. If the pooled SMDs of these comparisons are considered as effect sizes, all three of these findings are large (i.e., > 0.8) [52]. These suggest that a range of distinctively different exercise training modalities may improve overall balance ability in patients with PD; clinicians who prescribe exercise training should work with patients to identify a modality suitable for their capabilities and interests to increase the likelihood of efficacy. However, since the evidence from only three studies (direct comparison with control group), and the confidence in evidence was low. More homogeneity studies are needed in the future to verify our research results. Our findings showed that BWS_TT ranked according to P score is the best in improving overall balance ability and dynamic steady-state balance in PD patients. Previous research results showed that BWS_TT uses higher treadmill training speed compared to traditional treadmill training [40], and BWS_TT increases the safety of PD during training due to the body support device, making it dare to move at a larger pace [39, 40]. These are obviously more beneficial for BWS_TT to improve the overall balance ability and dynamic steady-state balance in PD. In addition, previous studies also had shown that specific ability tests are used as training content, and specific skills will be improved to the greatest extent [53]. In this study, the dynamic steady-state balance was evaluated by the speed and time of the walking test. Therefore, BWS_TT, which is similar to the dynamic steady-state balance test method, may also be the reason for the additional improvement of the dynamic steady-state balance ability of PD patients.

Our study showed that 6 exercise types significantly improved the static steady-state balance compared to the CON. Previous study had shown that many exercise types can consistently improve motor symptoms such as static steady-state balance in PD patients [54]. Exercise may increase synaptic strength and enhance functional circuitry, thereby improving behavior in patients with PD. Therefore, exercise-induced brain plasticity – the ability of the central nervous system to modify its structure and function in response to external stimuli – is likely to be the neural basis of rehabilitation in PD [38]. Moreover, exercises can promote the synthesis of neurotransmitters and brain neurotrophic factors [55]. Both neurochemical phenomena contribute to neuroplasticity [56]. These may be the reasons why the many types of exercise in this study significantly improved the static steady-state balance of PD patients compared with the CON. Our study showed that AQE ranks the best according to the p-score. Previous study provide evidence that water-based exercise is significantly superior to land-based exercise in improving static steady-state balance in PD patients [57], and water-based exercise had been widely used in physical therapy to improve balance in patients with different diseases [58–60]. The aquatic environment has unique properties such as buoyancy, turbulence, hydrostatic pressure and resistance. Water buoyancy reduces the effects of gravity, in fact, the aquatic environment is considered a microgravity environment. The results of previous research have documented improvements in static postural control due to prolonged periods spent in microgravity environments [61]. The control of body posture is altered due to inappropriate signals from the underwater vestibular system, these findings highlight a major role for the proprioceptive system in postural control in aquatic environments [62]. In addition, due to the effect of buoyancy in water reduces the patient’s fear of falling compared to the land [63], patients dare to try large-magnitude training movements during exercise, and the turbulent, hydrostatic pressure and resistance also increase the difficulty of training to varying degrees. These may be the reasons why AQE additionally improves static steady-state balance in PD patients. However, there were no control studies available, which makes this evidence completely indirect. In the future, more high-quality randomized controlled trials are needed to verify the benefits of AQE in improving static steady-state balance in PD patients. At the same time, VR and Mul_C also showed a large effect size (i.e., > 0.8) [52] in improving the static steady-state balance of PD patients, which also allowed clinicians and PD patients to choose appropriate alternative treatment methods according to the actual situation.

Our study showed that Pilates showed the best effective for improvements in proactive balance, and significantly more than many other exercise types. Pilates training could be considered a form of physical exercise focused on the improvement of strength, core stability, flexibility, muscular control, posture and breathing [64]. In addition, Pilates is to improve coordination and core muscle control, leading to the optimal lumbopelvic stabilization needed for daily life activities and functions [65]. The proactive balance improvement resulted from the effect of Pilates exercises can be studied based on the theory of systems. According to systems theory, body control is the result of simultaneous and complex interactions of the nervous, muscular and skeletal systems (posture control systems). A combination of sensory data (used to determine physical conditions in space) and the ability of the musculoskeletal system to exert the appropriate force is necessary in order to be controlled by the systems described above to maintain balance and therefore movement. Pilates exercises use information from the visual, vestibular and proprioceptive systems (including joint position sense and environmental sense) and support surface conditions to improve the central nervous system and actively adjust its own balance [66]. Therefore, with reference to systems theory and the impact of exercise on the improvement of these systems, Pilates exercise may additionally improve proactive balance in Parkinson’s patients with balance disorders due to decreased central nervous system function. However, since the evidence from only one study, the confidence in evidence was low. More homogeneity studies are needed in the future to verify our research results. In addition, AQE and Qigong also showed a large effect size (i.e., > 0.8) [52] in improving the proactive balance of PD patients, which also allowed clinicians and PD patients to choose appropriate alternative treatment methods according to the actual situation.

Our study showed that BGT_ECA and RA_GT showed similarly large effect in reactive balance compared to CON, and significantly more than many other exercise types. In daily life, reactive balance, referred to as the ability to control balance in response to mechanical disturbances, plays a critical role in avoiding and adapting to the complex environments that menace postural stability [67]. The use of rhythmic auditory or visual external stimuli can improve response balance training, as this allows PD patients to shift their habitual motor control (predominantly relying on the posterior putamen) to more goal-directed motor control (involving the anterior putamen), thereby improving motor learning [68]. In addition, external cues can improve attention and task prioritization (better executive control) in PD patients [69–71], and promoting prioritize balance control over other tasks, thereby improving reaction time for body control under unpredictable distractions, and external stimuli can also act as external rewards, further promoting the motor learning process [72]. At the same time, RA_GT provides repetitive, bilateral, distal-guided gait training, and it is most likely that several repetitions of gait-like movements could have had a positive influence on activation patterns within the leg muscles [73]. Moreover, this guided gait repetition provides an external rhythm that can compensate for deficits in the internal rhythm of the basal ganglia [74]. These may be the reason why both BGT_ECA and RA_GT have better effects in improving the reactive balance of PD patients. However, there were no control studies available, which makes this evidence completely indirect. In the future, more high-quality randomized controlled trials are needed to verify the benefits of BGT_ECA and RA_GT in improving reactive balance in PD patients.

## Strengths and limitations

The strengths of this study included that searches were not limited by publication data of language, and studies included were not restricted to a specific type of intervention or comparator. A significant advantage of network meta-analyses over traditional pairwise meta-analyses is the ability to make indirect comparisons, allowing the effects of multiple interventions to be considered in a single statistical model [75]. Thus, network meta-analyses summarize both direct and indirect comparisons between multiple interventions, and enable more sophisticated statistical models and more comprehensive interpretations. In addition, due to the specificity of balance ability, we evaluated the effect of exercise on overall balance, static steady-state balance, dynamic steady-state balance, proactive balance, and reactive balance, respectively, and based on the results provided a better and alternative exercise types. Therefore, clinicians can formulate exercise prescriptions according to the actual situation of patients.

There are limitations to our study. We did not consider the safety differences between exercise types. Underreporting of adverse events is a known issue associated with the reporting of exercise training studies [76]. Exercise experiments tend to be small in scale. In our study, 166 included studies (83.41%) with a sample size of less than 30 (a consensus of the minimum sample size for a trial [77]), and only one included study with a sample size of more than 100. Small-study effects may reflect publication bias, differential presence of quality issues in smaller trials, but also many other factors [78]. This is also reflected in meta-regression results, where the sample size significantly affects the proactive balancing. However, the results of publication bias showed that there is no funnel plot asymmetry and small sample effects. For the training dose, the included studies have a large training period span (4–96 weeks). Previous studies have showed that training period is a significant factor affecting exercise effects [79, 80]. This put the heterogeneity of our included studies at moderate levels, and our meta-regression results found that training period also significantly affected the exercise benefits in proactive balance. It is worth affirming that this also proves that the extension of the exercise training period can increase the exercise effects. In addition, we defined and classified 24 exercise types according to their content or modalities. This may have biased our findings. For example, Mul_C is a combination of various types of exercise, but not all of them are the same combination of several forms of exercise. Another example is AQE, we only consider the environment of the exercise, and do not consider the modalities of exercise in the water. Therefore, more research is needed in the future to support our findings.

## Conclusion

In conclusion, this NMA confirms that exercise therapy has clear benefits in balance for people with PD and also shows that the magnitude of effect varies according to type of exercise and outcome of interest. There was low-quality evidence that BWS_TT was found to be the best for overall balance and dynamic steady-state balance, AQE showed the best effects in static steady-state balance, Pilates had the best effects in improving proactive balance, moreover, BGT_ECA and RA_GT showed similar larger effects in improving reactive balance. The findings of this review may help clinicians guide their prescription of exercise type with respect to treatment outcomes.

### Supplementary Information

**Additional file 1: Appendix 1.** Search Strategy. **Appendix 2.** Definitions of exercise types and non-exercise training control. **Appendix 3.** Assessment of the transitivity. **Appendix 4.** Characteristics of studies and subjects included in the review. **Appendix 5.** The risk of bias assessment for the individual included studies. **Appendix 6.** Network meta-analysis results. **Appendix 7.** Details of SIDE splitting results. **Appendix 8.** Sensitivity analyses. **Appendix 9.** Grading the evidence for primary outcome of the network meta-analysis using CINeMA.

## Data Availability

All data generated or analysed during this study are included in this published article [and its supplementary information files].
