# Supplementary material for: Comparative efficacy of 24 exercise types on postural instability in adults with Parkinson’s disease: a systematic review and network meta-analysis
Source: BMC Geriatr. 2023 Aug 28;23:522. doi: 10.1186/s12877-023-04239-9 (PMC10463698; doi:10.1186/s12877-023-04239-9)
Supplement: Supplementary file 1 — Additional file 1: Appendix 1. Search Strategy. Appendix 2. Definitions of exercise types and non-exercise training control. Appendix 3. Assessment of the transitivity. Appendix 4. Characteristics of studies and subjects included in the review. Appendix 5. The risk of bias assessment for the individual included studies. Appendix 6. Network meta-analysis results. Appendix 7. Details of SIDE splitting results. Appendix 8. Sensitivity analyses. Appendix 9. Grading the evidence for primary outcome of the network meta-analysis using CINeMA. [file 12877_2023_4239_MOESM1_ESM.docx]

Supplementary

[Appendix 1: Search Strategy 4](#_Toc136423774)

[1.1 Database: PubMed <inception to January 23 2023> 4](#_Toc136423775)

[1.2 Database: Ovid MEDLINE(R) <1946 to January 23 2023> 7](#_Toc136423776)

[1.3 Database: Embase <1974 to January 23 2023> 8](#_Toc136423777)

[1.4 Database: PsycINFO <1806 to January 23 2023> 9](#_Toc136423778)

[1.5 Cochrane 11](#_Toc136423779)

[1.6 Database: Web of Science <1965 to January 23 2023> 12](#_Toc136423780)

[Appendix 2: Definitions of exercise types and non-exercise training control 15](#_Toc136423781)

[Appendix 3: Assessment of the transitivity 17](#_Toc136423782)

[3.1 Publish years 17](#_Toc136423783)

[3.2 Mean age 18](#_Toc136423784)

[3.3 Years of diagnosis 19](#_Toc136423785)

[3.4 Hoehn and Yahr stage 20](#_Toc136423786)

[3.5 Percentage male 21](#_Toc136423787)

[3.6 Sample size 22](#_Toc136423788)

[Appendix 4: Characteristics of studies and subjects included in the review 23](#_Toc136423789)

[List of included studies 32](#_Toc136423790)

[Appendix 5: The risk of bias assessment for the individual included studies. 49](#_Toc136423791)

[Appendix 6: Network meta-analysis results 64](#_Toc136423792)

[Table 6.1: League Table of Balance Test Batteries 64](#_Toc136423793)

[Table 6.2: League Table of Static Steady-State Balance 66](#_Toc136423794)

[Table 6.3: League Table of Dynamic Steady-State Balance 67](#_Toc136423795)

[Table 6.4: League Table of Proactive Balance 68](#_Toc136423796)

[Table 6.5: League Table of Reactive Balance 69](#_Toc136423797)

[Appendix 7: Details of SIDE splitting results 70](#_Toc136423798)

[Table 7.1 Details of SIDE splitting results (Balance test batteries) 70](#_Toc136423799)

[Table 7.2 Details of SIDE splitting results (Static steady-state balance) 81](#_Toc136423800)

[Table 7.3 Details of SIDE splitting results (dynamic steady-state balance) 89](#_Toc136423801)

[Table 7.4 Details of SIDE splitting results (Proactive balance) 100](#_Toc136423802)

[Table 7.5 Details of SIDE splitting results (Reactive balance) 110](#_Toc136423803)

[Appendix 8: Sensitivity analyses 114](#_Toc136423804)

[Table 8.1 Changes in heterogeneity 114](#_Toc136423805)

[Figure 8.1 Exclude studies at overall high risk of bias (balance test batteries) 116](#_Toc136423806)

[Figure 8.2 Exclude studies with exercise period less than 4 and more than 24 weeks (balance test batteries) 117](#_Toc136423807)

[Figure 8.3 Exclude studies with exercise frequency less than 2 and more than 4 (balance test batteries) 118](#_Toc136423808)

[Figure 8.4 Exclude studies that were OFF state during testing (balance test batteries) 119](#_Toc136423809)

[Figure 8.5 Exclude studies that use GetData to extract data and estimated standard deviations value (balance test batteries) 120](#_Toc136423810)

[Figure 8.6 Exclude studies at overall high risk of bias (static steady-state balance) 121](#_Toc136423811)

[Figure 8.7 Exclude studies with exercise period less than 4 and more than 24 weeks (Static steady-state balance) 122](#_Toc136423812)

[Figure 8.8 Exclude studies with exercise frequency less than 2 and more than 4 (static steady-state balance) 123](#_Toc136423813)

[Figure 8.9 Exclude studies that were OFF state during testing (static steady-state balance) 124](#_Toc136423814)

[Figure 8.10 Exclude studies that use GetData to extract data and estimated standard deviations value (Static steady-state balance) 125](#_Toc136423815)

[Figure 8.11 Exclude studies at overall high risk of bias (dynamic steady-state balance) 126](#_Toc136423816)

[Figure 8.12 Exclude studies with exercise period less than 4 and more than 24 weeks (dynamic steady-state balance) 127](#_Toc136423817)

[Figure 8.13 Exclude studies with exercise frequency less than 2 and more than 4 (dynamic steady-state balance) 128](#_Toc136423818)

[Figure 8.14 Exclude studies that were OFF state during testing (dynamic steady-state balance) 129](#_Toc136423819)

[Figure 8.15 Exclude studies that use GetData to extract data and estimated standard deviations value (dynamic steady-state balance) 130](#_Toc136423820)

[Figure 8.16 Exclude studies at overall high risk of bias (proactive balance) 131](#_Toc136423821)

[Figure 8.17 Exclude studies with exercise period less than 4 and more than 24 weeks (proactive balance) 132](#_Toc136423822)

[Figure 8.18 Exclude studies with exercise frequency less than 2 and more than 4 (proactive balance) 133](#_Toc136423823)

[Figure 8.19 Exclude studies that were OFF state during testing (proactive balance) 134](#_Toc136423824)

[Figure 8.20 Exclude studies that use GetData to extract data and estimated standard deviations value (proactive balance) 135](#_Toc136423825)

[Figure 8.21 Exclude studies with exercise period less than 4 and more than 24 weeks (reactive balance) 136](#_Toc136423826)

[Figure 8.23 Exclude studies with exercise frequency less than 2 and more than 4 (reactive balance) 137](#_Toc136423827)

[Appendix 9: Grading the evidence for primary outcome of the network meta-analysis using CINeMA 138](#_Toc136423828)

[9.1.1 Overall balance ability 138](#_Toc136423829)

[9.1.2 Static steady-state balance 139](#_Toc136423830)

[9.1.3 Dynamic steady-state balance 140](#_Toc136423831)

[9.1.4 Proactive balance 141](#_Toc136423832)

[9.1.5 Reactive balance 142](#_Toc136423833)

[9.2 Reasons for downgrading 143](#_Toc136423834)

[9.3.1 CINeMA for overall balance ability 145](#_Toc136423835)

[9.3.2 CINeMA for static steady-state balance 159](#_Toc136423836)

[9.3.3 CINeMA for dynamic steady-state balance 169](#_Toc136423837)

[9.3.4 CINeMA for proactive balance 182](#_Toc136423838)

[9.3.5 CINeMA for reactive balance 194](#_Toc136423839)

# Appendix 1: Search Strategy

## 1.1 Database: PubMed <inception to January 23 2023>

***Search Strategy:***

| #25 | Search: ((Parkinson disease[MeSH Terms]) AND ((((((((((exercise*[MeSH Terms]) OR (resistance training[MeSH Terms])) OR (Tai Ji[MeSH Terms])) OR (Qigong[MeSH Terms])) OR (Exercise Movement Techniques[MeSH Terms])) OR (Yoga[MeSH Terms])) OR (Virtual Reality[MeSH Terms])) OR (hydrotherapy[MeSH Terms])) OR (Dance Therapy[MeSH Terms])) OR ("aerobic exercise" or "aquatic exercise" or "balance training" or "body weight support treadmill" or "gait training" or "high-speed resistance training" or "multicomponent exercise program" or "multidisciplinary exercise program" or "Nordic Walking" or Physiotherapy or pilates or "power training" or "Robotic-assisted gait training" or stretch or Tango or "treadmill training" or "walking" or "whole body vibration"))) AND ((((((((randomized controlled trial[Publication Type]) OR (controlled clinical trial[Publication Type])) OR (randomized[Title/Abstract])) OR (placebo[Title/Abstract])) OR (randomly[Title/Abstract])) OR (trial[Title])) OR (clinical trials as topic[MeSH Terms])) NOT ((animals[MeSH Terms]) NOT (humans[MeSH Terms]))) | 890 |
| --- | --- | --- |
| #24 | Search: (((((((randomized controlled trial[Publication Type]) OR (controlled clinical trial[Publication Type])) OR (randomized[Title/Abstract])) OR (placebo[Title/Abstract])) OR (randomly[Title/Abstract])) OR (trial[Title])) OR (clinical trials as topic[MeSH Terms])) NOT ((animals[MeSH Terms]) NOT (humans[MeSH Terms])) | 1,341,164 |
| #23 | Search: ((((((randomized controlled trial[Publication Type]) OR (controlled clinical trial[Publication Type])) OR (randomized[Title/Abstract])) OR (placebo[Title/Abstract])) OR (randomly[Title/Abstract])) OR (trial[Title])) OR (clinical trials as topic[MeSH Terms]) | 1,449,600 |
| #22 | Search: (((((((((exercise*[MeSH Terms]) OR (resistance training[MeSH Terms])) OR (Tai Ji[MeSH Terms])) OR (Qigong[MeSH Terms])) OR (Exercise Movement Techniques[MeSH Terms])) OR (Yoga[MeSH Terms])) OR (Virtual Reality[MeSH Terms])) OR (hydrotherapy[MeSH Terms])) OR (Dance Therapy[MeSH Terms])) OR ("aerobic exercise" or "aquatic exercise" or "balance training" or "body weight support treadmill" or "gait training" or "high-speed resistance training" or "multicomponent exercise program" or "multidisciplinary exercise program" or "Nordic Walking" or Physiotherapy or pilates or "power training" or "Robotic-assisted gait training" or stretch or Tango or "treadmill training" or "walking" or "whole body vibration") | 551,542 |
| #21 | Search: (animals[MeSH Terms]) NOT (humans[MeSH Terms]) | 4,815,925 |
| #20 | Search: humans[MeSH Terms] | 19,183,084 |
| #19 | Search: animals[MeSH Terms] | 23,999,009 |
| #18 | Search: clinical trials as topic[MeSH Terms] | 355,600 |
| #17 | Search: trial[Title] | 238,308 |
| #16 | Search: randomly[Title/Abstract] | 356,459 |
| #15 | Search: placebo[Title/Abstract] | 223,336 |
| #14 | Search: randomized[Title/Abstract] | 561,707 |
| #13 | Search: controlled clinical trial[Publication Type] | 617,986 |
| #12 | Search: randomized controlled trial[Publication Type] | 528,725 |
| #11 | Search: "aerobic exercise" or "aquatic exercise" or "balance training" or "body weight support treadmill" or "gait training" or "high-speed resistance training" or "multicomponent exercise program" or "multidisciplinary exercise program" or "Nordic Walking" or Physiotherapy or pilates or "power training" or "Robotic-assisted gait training" or stretch or Tango or "treadmill training" or "walking" or "whole body vibration" | 361,398 |
| #10 | Search: Dance Therapy[MeSH Terms] | 396 |
| #9 | Search: hydrotherapy[MeSH Terms] | 20,257 |
| #8 | Search: Virtual Reality[MeSH Terms] | 2,684 |
| #7 | Search: Yoga[MeSH Terms] | 3,002 |
| #6 | Search: Exercise Movement Techniques[MeSH Terms] | 8,700 |
| #5 | Search: Qigong[MeSH Terms] | 229 |
| #4 | Search: Tai Ji[MeSH Terms] | 1,183 |
| #3 | Search: resistance training[MeSH Terms] | 9,538 |
| #2 | Search: exercise*[MeSH Terms] | 297,336 |
| #1 | Search: Parkinson disease[MeSH Terms] | 69,308 |

## 1.2 Database: Ovid MEDLINE(R) <1946 to January 23 2023>

***Search Strategy: --------------------------------------------------------------------------------***

1 Parkinson$.mp. (136173)

2 exp parkinson disease/ (69312)

3 (aerobic exercise or aquatic exercise or balance training or body weight support treadmill or Dance Therapy or exercise$ or Exercise Movement Techniques or gait training or high-speed resistance training or hydrotherapy or multicomponent exercise program or multidisciplinary exercise program or Nordic Walking or Physiotherapy or pilates or power training or Qigong or resistance training or Robotic-assisted gait training or stretch or tai ji or Tango or treadmill training or walking or Virtual Reality or whole body vibration or Yoga).mp. (540012)

4 exp resistance training/ (9532)

5 exp exercise$/ (206975)

6 exp tai ji/ (1182)

7 exp Qigong/ (228)

8 exp Exercise Movement Techniques/ (8695)

9 exp Yoga/ (2999)

10 exp Virtual Reality/ (2682)

11 exp hydrotherapy/ (20254)

12 exp Dance Therapy/ (396)

13 randomized controlled trial.pt. (527440)

14 controlled clinical trial.pt. (94123)

15 randomized.ab. (517037)

16 clinical trials as topic.sh. (195553)

17 randomly.ab. (355668)

18 trial.ti. (238446)

19 exp clinical trial/ (888782)

20 exp randomized controlled trials/ (145969)

21 exp cross-over studies/ (49955)

22 (clinic$ adj2 trial).mp. (746815)

23 (random$ adj5 control$ adj5 trial$).mp. (770827)

24 (crossover or cross-over).mp. (100433)

25 randomi$.mp. (943880)

26 (random$ adj5 (assign$ or allocat$ or assort$ or reciev$)).mp. (256419)

27 1 or 2 (136173)

28 3 or 4 or 5 or 6 or 7 or 8 or 9 or 10 or 11 or 12 (586531)

29 13 or 14 or 15 or 16 or 17 or 18 or 19 or 20 or 21 or 22 or 23 or 24 or 25 or 26 (1770322)

30 27 and 28 and 29 (1246)

1.3 Database: Embase <1974 to January 23 2023>
***Search Strategy:***

--------------------------------------------------------------------------------

1 Parkinson$.mp. (219918)

2 exp parkinson disease/ (163492)

3 (aerobic exercise or aquatic exercise or balance training or body weight support treadmill or Dance Therapy or exercise$ or Exercise Movement Techniques or gait training or high-speed resistance training or hydrotherapy or multicomponent exercise program or multidisciplinary exercise program or Nordic Walking or Physiotherapy or pilates or power training or Qigong or resistance training or Robotic-assisted gait training or stretch or tai ji or Tango or treadmill training or walking or Virtual Reality or whole body vibration or Yoga).mp. (816955)

4 exp resistance training/ (20137)

5 exp exercise$/ (363106)

6 exp tai ji/ (3173)

7 exp Qigong/ (836)

8 exp Exercise Movement Technique/ (82933)

9 exp Yoga/ (8492)

10 exp Virtual Reality/ (18896)

11 exp hydrotherapy/ (3829)

12 exp Dance Therapy/ (527)

13 randomized.ab. (757440)

14 randomly.ab. (481077)

15 trial.ti. (332106)

16 exp clinical trial/ (1627821)

17 exp randomized controlled trials/ (200725)

18 exp cross-over studies/ (66963)

19 (clinic$ adj2 trial).mp. (1639410)

20 (random$ adj5 control$ adj5 trial$).mp. (961796)

21 (crossover or cross-over).mp. (123532)

22 randomi$.mp. (1323771)

23 (random$ adj5 (assign$ or allocat$ or assort$ or reciev$)).mp. (209949)

24 1 or 2 (219918)

25 3 or 4 or 5 or 6 or 7 or 8 or 9 or 10 or 11 or 12 (841979)

26 13 or 14 or 15 or 16 or 17 or 18 or 19 or 20 or 21 or 22 or 23 (2779253)

27 24 and 25 and 26 (2562)

## 1.4 Database: PsycINFO <1806 to January 23 2023>

***Search Strategy:***

| Set No. Searched for Databases Results | | | |
| --- | --- | --- | --- |
| S1 | Parkinson* | APA PsycInfo® | 39453 |
| S2 | mainsubject(parkinson disease) | APA PsycInfo® | 25842 |
| S3 | su((aerobic exercise or aquatic exercise or balance training or body weight support treadmill or Dance Therapy or exercise$ or Exercise Movement Techniques or gait training or high-speed resistance training or hydrotherapy or multicomponent exercise program or multidisciplinary exercise program or Nordic Walking or Physiotherapy or pilates or power training or Qigong or resistance training or Robotic-assisted gait training or stretch or tai ji or Tango or treadmill training or walking or Virtual Reality or whole body vibration or Yoga)) | APA PsycInfo® | 63328 |
| S4 | su(exercise$) | APA PsycInfo® | 39377 |
| S6 | su(physical activity) | APA PsycInfo® | 39490 |
| S7 | ab(randomized) | APA PsycInfo® | 83500 |
| S8 | ab(randomly) | APA PsycInfo® | 75844 |
| S9 | ti(trial) | APA PsycInfo® | 41193 |
| S10 | ab(clinical trial) | APA PsycInfo® | 51602 |
| S11 | ab(randomized controlled trials) | APA PsycInfo® | 36991 |
| S12 | ab(cross-over studies) | APA PsycInfo® | 2076 |
| S13 | ab(crossover studies) | APA PsycInfo® | 5363 |
| S14 | ab(randomi*) | APA PsycInfo® | 83917 |
| S15 | su(animals) | APA PsycInfo® | 459210 |
| S16 | S1 OR S2 | APA PsycInfo® These databases are searched for part of your query. | 39453 |
| S17 | S3 OR S4 OR "S5" | APA PsycInfo® These databases are searched for part of your query. | 63473 |
| S18 | S6 OR S7 OR "S8" OR "S9" OR "S10" OR "S11" OR "S12" OR "S13" OR "S14" | APA PsycInfo® These databases are searched for part of your query. | 120428 |
| S19 | S16 AND S17 | APA PsycInfo® These databases are searched for part of your query. | 1074 |
| S20 | S18 AND S19 | APA PsycInfo® These databases are searched for part of your query. | 277 |
| S21 | S20 NOT S15 | APA PsycInfo® These databases are searched for part of your query. | 251 |

## 1.5 Cochrane

#1  MeSH descriptor: [Parkinson disease] explode all trees (4376)

#2 (aerobic exercise or aquatic exercise or balance training or body weight support treadmill or Dance Therapy or exercise* or Exercise Movement Techniques or gait training or high-speed resistance training or hydrotherapy or multicomponent exercise program or multidisciplinary exercise program or Nordic Walking or Physiotherapy or pilates or power training or Qigong or resistance training or Robotic-assisted gait training or stretch or tai ji or Tango or treadmill training or walking or Virtual Reality or whole body vibration or Yoga) in Trials (Word variations have been searched) (155706)

#3 MeSH descriptor: [resistance training] explode all trees (3641)

#4 MeSH descriptor: [exercise] explode all trees (25628)

#5 MeSH descriptor: [tai ji] explode all trees (373)

#6 MeSH descriptor: [Qigong] explode all trees (79)

#7 MeSH descriptor: [Exercise Movement Technique] explode all trees (2215)

#8 MeSH descriptor: [Yoga] explode all trees (699)

#9 MeSH descriptor: [Virtual Reality] explode all trees (284)

#10 MeSH descriptor: [hydrotherapy] explode all trees (1575)

#11 MeSH descriptor: [Dance Therapy] explode all trees (89)

#12 #2 or #3 or #4 or #5 or #6 or #7 or #8 or #9 or #10 or #11 (147882)

#13 #1 and #12 (906)

## 1.6 Database: Web of Science <1965 to January 23 2023>

| # 13 | 2,403 | #12 AND #11 AND #1  Indexes=SCI-EXPANDED, SSCI, A&HCI, CPCI-S, CPCI-SSH, BKCI-S, BKCI-SSH, ESCI, CCR-EXPANDED, IC Timespan=All years |  |  |
| --- | --- | --- | --- | --- |
| # 12 | 981,618 | #10 OR #9 OR #8 OR #7 OR #6 OR #5 OR #4 OR #3 OR #2  Indexes=SCI-EXPANDED, SSCI, A&HCI, CPCI-S, CPCI-SSH, BKCI-S, BKCI-SSH, ESCI, CCR-EXPANDED, IC Timespan=All years |  |  |
| # 11 | 6,290,817 | TOPIC: ((“randomized controlled trial*” or “controlled clinical trial” or “random*” or “clinical trial*” or randomly or trial or “clinical trial” or “randomized controlled trial*” or “cross-over studies” or clinic*) )  Indexes=SCI-EXPANDED, SSCI, A&HCI, CPCI-S, CPCI-SSH, BKCI-S, BKCI-SSH, ESCI, CCR-EXPANDED, IC Timespan=All years |  |  |
| # 10 | 7,671 | TOPIC: ((Yoga or “Muscle Stretching Exercises”) )  Indexes=SCI-EXPANDED, SSCI, A&HCI, CPCI-S, CPCI-SSH, BKCI-S, BKCI-SSH, ESCI, CCR-EXPANDED, IC Timespan=All years |  |  |
| # 9 | 373 | TOPIC: ((“Dance Therapy” or “Therapy, Dance” or “Dance Therapies” or “Therapies, Dance”) )  Indexes=SCI-EXPANDED, SSCI, A&HCI, CPCI-S, CPCI-SSH, BKCI-S, BKCI-SSH, ESCI, CCR-EXPANDED, IC Timespan=All years |  |  |
| # 8 | 1,197 | TOPIC: ((hydrotherapy or Hydrotherapies or “Whirlpool Baths” or “Bath, Whirlpool” or “Baths, Whirlpool” or “Whirlpool Bath”) )  Indexes=SCI-EXPANDED, SSCI, A&HCI, CPCI-S, CPCI-SSH, BKCI-S, BKCI-SSH, ESCI, CCR-EXPANDED, IC Timespan=All years |  |  |
| # 7 | 46,480 | TOPIC: (("Virtual Reality" or "Reality, Virtual" or "Virtual Reality, Educational" or "Educational Virtual Realities" or "Educational Virtual Reality" or "Reality, Educational Virtual" or "Virtual Realities, Educational" or "Virtual Reality, Instructional" or "Instructional Virtual Realities" or "Instructional Virtual Reality" or "Realities, Instructional Virtual" or "Reality, Instructional Virtual" or "Virtual Realities, Instructional") )  Indexes=SCI-EXPANDED, SSCI, A&HCI, CPCI-S, CPCI-SSH, BKCI-S, BKCI-SSH, ESCI, CCR-EXPANDED, IC Timespan=All years |  |  |
| # 6 | 234 | TOPIC: (("Exercise Movement Techniques" or "Movement Techniques, Exercise" or "Exercise Movement Technics" or "Pilates-Based Exercises" or "Exercises, Pilates-Based" or "Pilates Based Exercises" or "Pilates Training" or "Training, Pilates"）)  Indexes=SCI-EXPANDED, SSCI, A&HCI, CPCI-S, CPCI-SSH, BKCI-S, BKCI-SSH, ESCI, CCR-EXPANDED, IC Timespan=All years |  |  |
| # 5 | 4,058 | TOPIC: (“Tai-ji” or “Tai Chi” or “Chi, Tai” or “Tai Ji Quan” or “Ji Quan, Tai” or “Quan, Tai Ji” or Taiji or Taijiquan or “T'ai Chi” or “Tai Chi Chuan” Qigong or “Qi Gong” or “Ch'i Kung”)  Indexes=SCI-EXPANDED, SSCI, A&HCI, CPCI-S, CPCI-SSH, BKCI-S, BKCI-SSH, ESCI, CCR-EXPANDED, IC Timespan=All years |  |  |
| # 4 | 625,989 | TOPIC: (Exercise* or “Exercise Program, Weight-Bearing” or “Exercise Programs, Weight-Bearing” or “Weight Bearing Exercise Program” or “Weight-Bearing Exercise Programs” Exercise* or “Physical Activity” or “Activities, Physical” or “Activity, Physical” or “Physical Activities” or “Exercise, Physical” or “Exercises, Physical” or “Physical Exercise” or “Physical Exercises” or “Exercise, Isometric” or “Exercises, Isometric” or “Isometric Exercises” or “Isometric Exercise” or “Exercise, Aerobic” or “Aerobic Exercise” or “Aerobic Exercises” or “Exercises, Aerobic” or “Exercise Training” or “Exercise Trainings” or “Training, Exercise” or “Trainings, Exercise”)  Indexes=SCI-EXPANDED, SSCI, A&HCI, CPCI-S, CPCI-SSH, BKCI-S, BKCI-SSH, ESCI, CCR-EXPANDED, IC Timespan=All years |  |  |
| # 3 | 17,738 | TOPIC: ("Resistance training” or “Training, Resistance” or “Strength Training” or “Training, Strength” or “Weight-Lifting Strengthening Program” or “Strengthening Program, Weight-Lifting” or “Strengthening Programs, Weight-Lifting” or “Weight Lifting Strengthening Program” or “Weight-Lifting Strengthening Programs” or “Weight-Lifting Exercise Program” or “Exercise Program, Weight-Lifting” or “Exercise Programs, Weight-Lifting” or “Weight Lifting Exercise Program” or “Weight-Lifting Exercise Programs” or “Weight-Bearing Strengthening Program” or “Strengthening Program, Weight-Bearing” or “Strengthening Programs, Weight-Bearing” or “Weight Bearing Strengthening Program” or “Weight-Bearing Strengthening Programs” or “Weight-Bearing Exercise Program”)  Indexes=SCI-EXPANDED, SSCI, A&HCI, CPCI-S, CPCI-SSH, BKCI-S, BKCI-SSH, ESCI, CCR-EXPANDED, IC Timespan=All years |  |  |
| # 2 | 424,147 | TOPIC: (“aerobic exercise” or “aquatic exercise” or “balance training” or “body weight support treadmill” or “Dance Therapy or exercise*” or “Exercise Movement Techniques” or “gait training” or “high-speed resistance training” or “hydrotherapy” or “multicomponent exercise program” or “multidisciplinary exercise program” or “Nordic Walking” or “Physiotherapy” or pilates or “power training” or Qigong or “resistance training” or “Robotic-assisted gait training” or stretch or “tai ji” or Tango or “treadmill training” or “walking” or “Virtual Reality” or “whole body vibration” or Yoga)  Indexes=SCI-EXPANDED, SSCI, A&HCI, CPCI-S, CPCI-SSH, BKCI-S, BKCI-SSH, ESCI, CCR-EXPANDED, IC Timespan=All years |  |  |
| # 1 | 113,262 | TOPIC: ("Idiopathic Parkinson's Disease" or "Lewy Body Parkinson's Disease" or "Parkinson's Disease, Idiopathic" or "Parkinson's Disease, Lewy Body" or "Parkinson Disease, Idiopathic" or "Parkinson's Disease" or "Idiopathic Parkinson Disease" or "Lewy Body Parkinson Disease" or "Primary Parkinsonism" or "Parkinsonism, Primary" or "Paralysis Agitans")  Indexes=SCI-EXPANDED, SSCI, A&HCI, CPCI-S, CPCI-SSH, BKCI-S, BKCI-SSH, ESCI, CCR-EXPANDED, IC Timespan=All years |  |  |

# Appendix 2: Definitions of exercise types and non-exercise training control

| abbreviation | Full name | Definitions |
| --- | --- | --- |
| AE | Aerobic Exercise | Aerobic exercise is performed by repeating sequences of light-to-moderate intensity activities for extended periods of time.^1^ e.g., walking, bicycle, etc. Exclude treadmill training, because this study treats treadmill training as a separate exercise type |
| AQE | Aquatic Exercise | Gait training, balance training, resistance training , or aerobic training performed in deep or shallow water.^2^ |
| BGT | Balance and Gait Training | Single-task balance and gait training without external cues or internal and external attention |
| BGT-ECA | Balance and Gait Training with external Cue or Attention | Focus on external cues or things while doing balance and gait training.^3^ |
| BGT-ICA | Balance and Gait Training with Internal Cue or Attention | Participants have been asked to focus on the movement of their limbs in the physical space while performing balance and gait training. An example of guidance given to this group during knee lift exercises is "focus on raising the knee in a slow, controlled manner.^4^ |
| BWS-TT | Body Weight Support Treadmill Training | Walking on a treadmill after reducing the weight of the body through the equipment |
| CON | Control group | Non-exercise intervention, usual care,^5^ or health education |
| CPP | Classic Physiotherapy Program | This program was performed according to the guidelines for physical therapy in patients with PD and included flexibility, strengthening, posture, breathing balance, walking exercises, and other functional activities.^6^ |
| Dance | - | Group dances other than tango, such as waltz, Irish set dancing etc. |
| DT-BGT | Dual Task Balance and Gait Training | The dual-task paradigm entails the simultaneous performance of two tasks with different objectives.^7^ This research is mainly to perform cognitive or motor tasks in addition to balance and gait training. |
| Mul_C | Multicomponent exercise program | Two or more of the above specific types of exercise training (if it is only part of warm-up or relaxation, it is not considered as multi-mode) |
| Mul_D | Multidisciplinary exercise program | The content of intervention includes the above types of exercise, in addition to other disciplines of intervention content (e.g., cognitive training, nutritional supplements, electrical stimulation, etc.) |
| NW | Nordic Walking | A walking exercise that uses walking poles with both hands |
| Pilates | - | Pilates exercise focuses on posture symmetry, breathing control, abdominal strength, spine, pelvic and shoulder stability, muscle flexibility, joint flexibility, and enhancement of the full range of motion through all joints. Not isolated muscle groups, but whole body training, integrating the upper and lower limbs with the trunk.^8^ |
| PT | Power Training | Similar to resistance training, however participants were instructed to exert force as fast as possible during the concentric phase and move slowly through the eccentric phase.^9^ |
| Qigong | - | It is a system of coordinated body-posture and movement, breathing, and meditation used for the purposes of health, spirituality, and martial-arts training. |
| RA_GT | Robotic-Assisted Gait Training | Robot-assisted gait training uses electromechanical equipment to assist the stepping cycle by supporting body weight, while automating the gait process by supporting and promoting the movement of one or more lower limb joints.^10^ |
| RT | Resistance Training | Exercise training designed to improve the strength, power, endurance and size of skeletal muscles.^11^ |
| Stretch | - | Reverse extension of muscle length to improve joint mobility |
| Tango | - | An argentine couple dance. When dancing tango, the two sides lean closely together. The right arm of the man and the left arm of the woman should be inward. The body is in contact with each other and the center of gravity is shifted. The man is mainly on the right foot and the woman is on the left foot. Both men and women do not look at each other, and both men and women look to their left side when positioning. |
| TC | Tai Chi | It is an internal Chinese martial art practiced for defense training, health benefits, and meditation. |
| TT | Treadmill Training | Walking on a treadmill at a constant speed. |
| VR | Virtual Reality | Use computer simulation to generate a virtual environment, provide users with a simulation of vision and other senses, so that users can feel as if they are immersed in the environment, and perform the above types of exercises on this basis |
| WBV | Whole Body Vibration | Exposure to low-amplitude, low-frequency mechanical stimulation throughout the body for a certain period of time^12^ |
| Yoga | - | Mainly a series of methods for self-cultivation, including body-adjusting asanas (refer to yoga asana collection), breathing-adjusting breathing methods, and mind-adjusting meditation, etc., to achieve the unity of body and mind.^13^ |

**Reference**

1. Plowman SA, Smith DL. Exercise physiology for health fitness and performance: Lippincott Williams & Wilkins; 2013.

2. Konlian C. Aquatic therapy: making a wave in the treatment of low back injuries. *Orthop Nurs* 1999; **18**(1).

3. Abdollahipour R, Wulf G, Psotta R, Palomo Nieto M. Performance of gymnastics skill benefits from an external focus of attention. *J Sports Sci* 2015; **33**(17): 1807-13.

4. Beck EN, Intzandt BN, Almeida QJ. Can Dual Task Walking Improve in Parkinson's Disease After External Focus of Attention Exercise? A Single Blind Randomized Controlled Trial. *Neurorehabil Neural Repair* 2018; **32**(1): 18-33.

5. Goh S-L, Persson MSM, Stocks J, et al. Relative Efficacy of Different Exercises for Pain, Function, Performance and Quality of Life in Knee and Hip Osteoarthritis: Systematic Review and Network Meta-Analysis. *Sports Med* 2019; **49**(5): 743-61.

6. Keus SHJ, Bloem BR, Hendriks EJM, Bredero-Cohen AB, Munneke M. Evidence-based analysis of physical therapy in Parkinson's disease with recommendations for practice and research. *Mov Disord* 2007; **22**(4).

7. McIsaac TL, Lamberg EM, Muratori LM. Building a framework for a dual task taxonomy. *Biomed Res Int* 2015; **2015**: 591475.

8. Muscolino JE, Cipriani S. Pilates and the “powerhouse”—I. *Journal of bodywork and movement therapies* 2004; **8**(1): 15-24.

9. Ni M, Signorile JF, Mooney K, et al. Comparative Effect of Power Training and High-Speed Yoga on Motor Function in Older Patients With Parkinson Disease. *Arch Phys Med Rehabil* 2016; **97**(3).

10. Nedergård H, Arumugam A, Sandlund M, Bråndal A, Häger CK. Effect of robotic-assisted gait training on objective biomechanical measures of gait in persons post-stroke: a systematic review and meta-analysis. *J Neuroeng Rehabil* 2021; **18**(1): 64.

11. Powell KE, Paluch AE, Blair SN. Physical activity for health: What kind? How much? How intense? On top of what? *Annu Rev Public Health* 2011; **32**: 349-65.

12. Bidonde J, Busch AJ, van der Spuy I, Tupper S, Kim SY, Boden C. Whole body vibration exercise training for fibromyalgia. *Cochrane Database Syst Rev* 2017; **9**: CD011755.

13. Cramer H, Lauche R, Haller H, Dobos G. A systematic review and meta-analysis of yoga for low back pain. *Clin J Pain* 2013; **29**(5): 450-60.

# Appendix 3: Assessment of the transitivity

Different clinical trials need to ensure that their baseline levels are consistent. If the baseline levels are inconsistent, the results cannot be transitive. Therefore, the transitivity assumption was evaluated by comparing the distribution of potential effect modifiers (publication year, sample size, mean age, percentage male, years of diagnosis, and disease grade) across studies grouped before analyzing the results, and we use the R ggplot2 package to draw boxplots between the above potential influencing factors and various types of exercise.

## 3.1 Publish years

We checked the publication year distribution of the included studies. The range is from 1996 to 2020, with a median of 2016. In addition, we examined the impact of the included study’s publication year as a potential influencing factor in a meta-regression.

**Figure 3.1:** Boxplot for distribution of publication year. *AE* Aerobic Exercise, *AQE* Aquatic Exercise, *BGT* Balance and Gait Training, *BGT_ECA* Balance and Gait Training with External Cue or Attention, *BGT_ICA* Balance and Gait Training with Internal Cue or Attention, *BWS_TT* Body Weight Support Treadmill Training, *CON* Control group, *CPP* Classic Physiotherapy Program, *DT_BGT* Dual Task Balance and Gait Training, *Mul_C* Multicomponent Exercise Program, *Mul_D* Multidisciplinary Exercise Program, *NW* Nordic Walking, *PT* Power Training, *RA_GT* Robotic Assisted Gait Training, *RT* Resistance Training, *TC* Tai Chi, *TT* Treadmill Training, *VR* Virtual Reality, *WBV* Whole Body Vibration

## 3.2 Mean age

We checked the mean age distribution of the included study participants . The range is from 53.2 to 81.4, with a median of 67.5. In addition, we examined the impact of the mean age as a potential influencing factor in a meta-regression.

**Figure 3.2 :** Boxplot for distribution of mean age. *AE* Aerobic Exercise, *AQE* Aquatic Exercise, *BGT* Balance and Gait Training, *BGT_ECA* Balance and Gait Training with External Cue or Attention, *BGT_ICA* Balance and Gait Training with Internal Cue or Attention, *BWS_TT* Body Weight Support Treadmill Training, *CON* Control group, *CPP* Classic Physiotherapy Program, *DT_BGT* Dual Task Balance and Gait Training, *Mul_C* Multicomponent Exercise Program, *Mul_D* Multidisciplinary Exercise Program, *NW* Nordic Walking, *PT* Power Training, *RA_GT* Robotic Assisted Gait Training, *RT* Resistance Training, *TC* Tai Chi, *TT* Treadmill Training, *VR* Virtual Reality, *WBV* Whole Body Vibration

## 3.3 Years of diagnosis

We checked the year of diagnosis distribution of the included study participants . The range is from 0.73 to 17, with a median of 6.9. In addition, we examined the impact of the mean age as a potential influencing factor in a meta-regression.

**Figure 3.3 :** Boxplot for distribution of year of diagnosis. *AE* Aerobic Exercise, *AQE* Aquatic Exercise, *BGT* Balance and Gait Training, *BGT_ECA* Balance and Gait Training with External Cue or Attention, *BGT_ICA* Balance and Gait Training with Internal Cue or Attention, *BWS_TT* Body Weight Support Treadmill Training, *CON* Control group, *CPP* Classic Physiotherapy Program, *DT_BGT* Dual Task Balance and Gait Training, *Mul_C* Multicomponent Exercise Program, *Mul_D* Multidisciplinary Exercise Program, *NW* Nordic Walking, *PT* Power Training, *RA_GT* Robotic Assisted Gait Training, *RT* Resistance Training, *TC* Tai Chi, *TT* Treadmill Training, *VR* Virtual Reality, *WBV* Whole Body Vibration

## 3.4 Hoehn and Yahr stage

We checked the Hoehn and Yahr stage distribution of the included study participants . The range is from 1.3 to 3.5, with a median of 2.4 In addition, we examined the impact of the Hoehn and Yahr stage as a potential influencing factor in a meta-regression.

**Figure 3.4 :** Boxplot for distribution of Hoehn and Yahr stage. *AE* Aerobic Exercise, *AQE* Aquatic Exercise, *BGT* Balance and Gait Training, *BGT_ECA* Balance and Gait Training with External Cue or Attention, *BGT_ICA* Balance and Gait Training with Internal Cue or Attention, *BWS_TT* Body Weight Support Treadmill Training, *CON* Control group, *CPP* Classic Physiotherapy Program, *DT_BGT* Dual Task Balance and Gait Training, *Mul_C* Multicomponent Exercise Program, *Mul_D* Multidisciplinary Exercise Program, *NW* Nordic Walking, *PT* Power Training, *RA_GT* Robotic Assisted Gait Training, *RT* Resistance Training, *TC* Tai Chi, *TT* Treadmill Training, *VR* Virtual Reality, *WBV* Whole Body Vibration

## 3.5 Percentage male

We checked the percentage male distribution of the included study participants . The range is from 11.8 to 100%, with a median of 60. In addition, we examined the impact of the percentage male as a potential influencing factor in a meta-regression.

**Figure 3.5 :** Boxplot for distribution of percentage male. *AE* Aerobic Exercise, *AQE* Aquatic Exercise, *BGT* Balance and Gait Training, *BGT_ECA* Balance and Gait Training with External Cue or Attention, *BGT_ICA* Balance and Gait Training with Internal Cue or Attention, *BWS_TT* Body Weight Support Treadmill Training, *CON* Control group, *CPP* Classic Physiotherapy Program, *DT_BGT* Dual Task Balance and Gait Training, *Mul_C* Multicomponent Exercise Program, *Mul_D* Multidisciplinary Exercise Program, *NW* Nordic Walking, *PT* Power Training, *RA_GT* Robotic Assisted Gait Training, *RT* Resistance Training, *TC* Tai Chi, *TT* Treadmill Training, *VR* Virtual Reality, *WBV* Whole Body Vibration

## 3.6 Sample size

We checked the sample size distribution of the included studies. The range is from 4 to 238, with a median of 15. In addition, we examined the impact of the included study’s sample size as a potential influencing factor in a meta-regression (appendix 14.6)

**Figure 3.6 :** Boxplot for distribution of sample size. *AE* Aerobic Exercise, *AQE* Aquatic Exercise, *BGT* Balance and Gait Training, *BGT_ECA* Balance and Gait Training with External Cue or Attention, *BGT_ICA* Balance and Gait Training with Internal Cue or Attention, *BWS_TT* Body Weight Support Treadmill Training, *CON* Control group, *CPP* Classic Physiotherapy Program, *DT_BGT* Dual Task Balance and Gait Training, *Mul_C* Multicomponent Exercise Program, *Mul_D* Multidisciplinary Exercise Program, *NW* Nordic Walking, *PT* Power Training, *RA_GT* Robotic Assisted Gait Training, *RT* Resistance Training, *TC* Tai Chi, *TT* Treadmill Training, *VR* Virtual Reality, *WBV* Whole Body Vibration

# Appendix 4: Characteristics of studies and subjects included in the review

After screening the studies, all relevant articles were assessed for eligibility based on their full texts. At this stage, Three pairs of investigator extracted information on (1) relevant data regarding participant characteristics (e.g., the sample size, age, sex, years of diagnosis, and disease grade); (2) types and daily doses of medicines taken; (3) OFF (>12 hour withdrawal from dopaminergic medication) and ON (1-2 hour after taking their normal dopaminergic medication); (3) exercise type; (4) training variable (e.g., duration, frequency, and single course time); and (5) the main result of the study. The extracted data of the included studies were depicted in Table 5

Table 4.1 Characteristics of eligible RCTs included in network meta-analysis

| Study | **Age (Mean±SD)** | **Number (men)** | **Years of diagnosis** | **Hoehn and Yahr stage** | **Drug(mg)** | **state (ON/OFF)** | **Duration (weeks)** | **Frequency** | **Time (minutes)** | **static steady-state balance** | **dynamic steady-state balance** | **proactive balance** | **reactive balance** | **balance test batteries** |
| --- | --- | --- | --- | --- | --- | --- | --- | --- | --- | --- | --- | --- | --- | --- |
| Li et al. (2012) | TC: 68±9 RT: 69±8 Stretch: 69±9 | TC: 65 (20) RT: 65 (27) Stretch: 65 (26) | TC: 8±9 RT: 8±9 Stretch: 6±5 | TC: 2.2-2.87 RT: 2.2-2.84 Stretch: 2.3-3.05 | stable medication usage | ON | 24 | 2 | 60 | Maximum excursion | 4.3m walking test, gait velocity | FR; maximal reach distance; | NA | NA |
| Fil-Balkan et al. (2018) | Mul_C: 71.83±9.71 CPP: 72.75±9.23 | Mul_C: 12(7) CPP: 12(6) | Mul_C: 6.83±3.78 CPP: 6.91±5.07 | Mul_C: 2.75±0.26 CPP: 2.67±0.25 | stable medication usage | ON | 6 | 2 | 75 | CDP-SOT Total, score | NA | FR; maximal reach distance | NA | BBS, score |
| Tollár et al. (2018) | Mul_C: 67.3±3.4 CON: 67.6±4.1 | Mul_C: 35(17) CON: 20(12) | Mul_C: 6.7±2.3 CON: 7.1±2.8 | 2-3 | L-Dopa equivalent Mul_C: 843.4±308.8 CON: 884.8±332.0 | ON | 3 | 5 | 60 | 20-s narrow stance with eyes opened, Combined value of displacements | NA | TUG, time | NA | NA |
| Conradsson et al. (2015) | DT_BGT: 73.1±5.8  CON: 73.0±5.5 | DT_BGT: 51(32) CON: 49(25) | DT_BGT: 5.9±5.1 CON: 5.6±4.8 | DT_BGT: 2.55±0.5 CON: 2.57±0.5 | levodopa equivalent dose DT_BGT: 578±299 CON: 640±380 | ON | 10 | 3 | 60 | NA | 9m walking test, gait velocity | NA | NA | Mini-BESTest, score |
| Pohl et al. (2020) | BGT_ECA: 69.7 ± 7.0 CON: 70.4 ± 6.0 | BGT_ECA: 26(19) CON: 20(13) | BGT_ECA 6.0 ± 4.4 CON: 6.8 ± 3.6 | BGT_ECA: 2.4±0.69 CON: 2.3±0.65 | Levodopa equivalent dosage BGT_ECA: 727.7±327.3 CON: 690.0±231.0 | ON | 12 | 2 | 60 | NA | NA | Timed Up and Go subtracting 7’s, time | NA | Mini-BESTest, score |
| Myers et al. (2020) | CON: 65.0±8.7 Yoga: 70.5±8.7 | CON: 13(8) Yoga: 13(7) | NA | A:2(2-3) B:2(2-3) | stable medication usage | ON | 12 | 2 | 60 | BESTest-Sensory orientation, score | BESTest-Stability in gait, score | BESTest-Transitions/anticipatory, score | BESTest-Reactive, score | BESTest, score |
| Amano et al. (2013) | TC: 64±13 Qigong: 68±7 TC: 66±11  CON: 66±7 | TC: 12(5) Qigon: 9(2) TC: 15(8) CON: 9(2) | TC: 7±7 Qigong: 12±7 TC: 8±5 CON: 5±3 | TC: 2.3±0.4 Qigong: 2.2±0.4 TC: 2.4±0.6 CON: 2.4±0.4 | stable medication usage | ON | 16 | 2 | 60 | NA | gait velocity | NA | NA | NA |
| Beck et al. (2018) | BGT_ECA: 68.6±9.9 BGT_ICA: 73.1±7.8 CON: 71.3±6.6 | BGT_ECA: 19 (15) BGT_ICA: 20 (16) CON: 11 (10） | BGT_ECA: 7.0 ±5.0 BGT_ICA: 6.7±4.2 CON: 8.4±5.9 | NA | Levodopa (mg/d) BGT_ECA: 648.0±232.9 BGT_ICA: 594.0±358.3 CON: 867.7±674.6 | ON/OFF | 11 | 3 | 60 | NA | walking test, gait velocity | NA | NA | NA |
| S. M. Santos et al. (2017) | BGT: 68.5±6.5  RT: 67.0±7.9 | BGT: 21(7) RT: 19(11) | BGT: 5.4±5.3 RT: 5.6±4.2 | BGT: 2.3±0.6 RT: 2.3±0.5 | stable medication usage | ON | 8 | 2 | 60 | single leg stand with open eyes,COP Area | BESTest-stability in gait, score | BESTest-transitions and anticipatory postural adjustments, score | BESTest-reactive postural responses, score | BESTest, score |
| Capecci et al. (2014) | BGT: 66.8±4.9 CON: 68.1±5.6 | BGT: 7(4) CON: 7(4) | BGT: 9.5±7.4 CON: 9.6±4.9 | BGT: 3.3±0.7 CON: 3.3±0.9 | stable medication usage | ON | 4 | 3 | 40 | NA | NA | TUG, time | NA | BBS, score |
| Cherup et al. (2021) | Yoga: 69.8±7.3   BGT: 71.4±12.1 | Yoga: 15(10)  BGT: 18(11) | NA | Yoga: 1.7±0.5 BGT: 2±0.8 | stable medication usage | ON | 12 | 2 | 45 | NA | NA | TUG, time | NA | Tinetti assessment scale, score |
| Choi (2016) | TC: 60.8 ± 7.6  CON: 65.5 ± 6.8 | TC: 11 (NA) CON: 9 (NA) | TC: 5.2± 2.7  CON: 5.2± 2.7 | TC: 1.6±0.6  CON: 1.8±0.3 | stable medication usage | ON | 12 | 3 | 60 | stand on foot with eyes open, COP Aera | NA | FR, maximal reach distance | NA | NA |
| Conradsson et al. (2015) | DT_BGT: 72.9±6.0 CON: 73.6±5.3 | DT_BGT: 47 (28)  CON: 44(23) | DT_BGT: 6.0±5.1 CON: 5.6±5.0 | DT_BGT: 2.6±0.5 CON: 2.6±0.5 | Levodopa equivalent dosage DT_BGT: 581±295 CON: 645±404 | ON | 10 | 3 | 60 | NA | 9m walking test, velocity | NA | NA | Mini-BESTest |
| de Melo et al. (2018) | BGT: 65.6±13.0 TT: 61±10.7 VR: 60.3±9.3 | BGT: 12(5) TT: 13(12)  VR: 12(11) | NA | BGT: 2.1±0.9 TT: 1.5±0.7 VR: 1.4±0.5 | stable medication usage | ON | 4 | 3 | 20 | NA | 6 min walking test, velocity | NA | NA | NA |
| Ferraz et al. (2018) | BGT: 71±6.67 AE: 67±5.18 VR: 67±1.49 | BGT: 22(16) AE: 20(11) VR: 20(10) | BGT: 4  AE: 6  VR: 4 | BGT: 2.5 AE: 2.5  VR: 2.5 | stable medication usage | ON | 8 | 3 | 50 | NA | 10m walking test, velocity | NA | NA | NA |
| Gandolfi et al. (2017) | VR: 67.5±7.2 BGT: 69.8±9.4 | VR: 38(23) BGT: 38(28) | VR: 6.2±3.8 BGT: 7.5±3.9 | 2.5 | stable medication usage | ON | 7 | 3 | 50 | NA | 10m walking test, velocity | NA | NA | BBS, score |
| Hackney et al. (2008) | TC: 64.9± 8.3 CON: 62.6±10.2 | TC: 17(11) CON: 15(10) | TC: 8.7±4.7 CON: 5.5±3.3 | TC: 2±0.4 CON: 1.9± 0.2 | stable medication usage | ON | 13 | 2 | 60 | one-leg stance with open eyes, time | walking test, velocity | TUG, time | NA | BBS, score |
| Khuzema et al. (2020) | TC: 72±5.22 Yoga: 68.11± 4.23 CON: 70.89±6.01 | TC: 9(6) Yoga: 9(6) CON: 9(7) | TC: 5.67±2.33 Yoga: 6.2±1.67 CON: 5.23±3.12 | TC: 2.83±0.24 Yoga: 2.83±0.24 CON: 2.78±0.25 | stable medication usage | ON | 8 | 5 | 35 | NA | 10m walking test, time | TUG, time | NA | BBS, score |
| Kwok et al. (2019) | Yoga: 63.7±8.2  CON: 63.5±9.3 | Yoga: 71(37)  CON: 67( 28) | NA | Yoga: 2.68 ± 0.47  CON: 2.66 ±0.54 | levodopa equivalent dose Yoga: 2685±7870.6  CON: 2541.1 ±6442.0 | ON | 8 | 3 | 85 | NA | NA | TUG, time | NA | NA |
| Liao et al. (2015) | Mul_C: 64.6 ± 8.6 TT: 65.1 ± 6.7 VR: 67.3 ± 7.1 | Mul_C: 12(5) TT: 12(6) VR: 12(6) | Mul_C: 6.4 ± 3.0 TT: 6.9 ± 2.8 VR: 7.9 ± 2.7 | Mul_C: 1.9 ± 0.8 TT: 2.0 ± 0.8 VR: 2.0 ± 0.7 | stable medication usage | ON | 6 | 2 | 60 | sensory organization test, score | 10m walking test, velocity | TUG, time | NA | NA |
| Liu et al. (2016) | Qigong: 65.84 ± 5.45 CON: 62.5 ± 3.13 | experiment group: 28(11) control group: 26(14) | NA | NA | stable medication usage | ON | 10 | 5 | 60 | single leg stance with closed stand, time | NA | TUG, time | NA | NA |
| Feng et al. (2019) | VR: 67.47±4.79 CPP: 66.93±4.64 | VR: 14(8) CPP: 14(9) | VR: 7.07±1.44 CPP: 6.60±1.45 | VR: 3.03±0.55 CPP: 2.97±0.58 | Levodopa equivalent daily dose  VR: 203.6±47.2 CPP: 227.5±54.3 | ON | 12 | 5 | 45 | NA | NA | TUG, time | NA | BBS, score |
| Mollinedo-Cardalda et al. (2018) | Pilates: 62.85 ± 9.75 CON: 66.00 ± 13.14 | Pilates: 13(5) CON: 13(4) | Pilates: 5.77 ± 3.39 CON: 5.69 ± 4.40 | Pilates: 2.08 ± 0.49 CON: 2.00 ± 0.82 | stable medication usage | ON | 12 | 2 | 60 | NA | NA | TUG, time | NA | NA |
| Ni et al. (2016) | PT: 71.6±6.6 Yoga: 71.2±6.5 CON: 74.9± 8.3 | PT: 14(9) Yoga: 13(11) CON: 10(4) | PT: 6.6±4.4 Yoga: 6.9±6.3 CON: 5.9±6.2 | PT: 2.2±0.6 Yoga: 2.2±0.7 CON: 2.1± 0.7 | stable medication usage | ON | 12 | 2 | 60 | single leg stand with open eyes, time | 10m walking test, gait velocity | FR, maximal reach distance | NA | BBS, score |
| Hirsch et al. (2003) | BGT: 75.7±1.8 Mul_C: 70.8±2.8 | BGT: 9(NA) Mul_C: 6(NA) | BGT: 8.3±9.8 Mul_C: 5.5±3.91 | BGT: 1.9±0.6 Mul_C: 1.8±0.3 | stable medication usage | ON | 10 | 3 | 40 | Summary Equi Test, score | NA | NA | NA | NA |
| Schlenstedt et al. (2015) | RT: 75.7 ± 5.5 BGT: 75.7 ± 7.2 | RT:17(12) BGT: 15(9) | RT: 10.1 ± 6.0 BGT: 9.3 ± 7.9 | RT: 2.8 ± 0.26 BGT: 2.7 ± 0.4 | levodopa equivalent daily dose RT: 817.4 ± 468.0 BGT: 674.7 ± 294.9 | ON | 7 | 2 | 60 | NA | 5m walking test, velocity | TUG, time | NA | Fullerton Advanced Balance, score |
| Protas et al. (2005) | BGT: 71.3±7.4 CON: 73.7 ±8.5 | BGT: 9 (9) CON: 9 (9) | BGT: 7.1±5.1 CON: 8.1±4.4 | BGT: 2.8±0.35 CON: 2.9±0.17 | stable medication usage | ON | 8 | 3 | 60 | NA | 3m walking test, velocity | NA | NA | NA |
| Margaret Schenkman et al. (2012) | Mul_C: 66.3±10.1 BGT: 64.5±10 AE: 63.4±11.2 | Mul_C: 41(26) BGT: 39 (24) AE: 41 (26) | Mul_C: 4.5±3.8 BGT: 4.9±3.7 AE: 3.9±4.2 | Mul_C: 2.3±0.4 BGT: 2.3±0.4 AE: 2.2±0.5 | stable medication usage | ON | 16 | 3 | 50 | NA | NA | FR, maximal reach distance | NA | NA |
| Ribas et al. (2017) | VR: 61.7±6.8 RT: 60.2±11.2 | VR: 10 (4) RT: 10 (4) | VR: 6.5±4 RT: 7±2.8 | VR: 1.4±0.5 RT: 1.5±0.5 | stable medication usage | ON | 12 | 2 | 30 | NA | NA | NA | NA | BBS, score |
| L. Santos, J. Fernandez-Rio, K. Winge, B. Barragán-Pérez, V. Rodríguez-Pérez, et al. (2017) | BGT: 73±9.8 CON: 78±5.2 | BGT: 11(6) CON: 11(5) | BGT: 10.7±4.1 CON: 10.9±3.2 | BGT: 2.2±0.6 CON: 1.9±0.5 | stable medication usage | ON | 6 | 2 | 23 | bipeda stand with open eyes, COP distance | NA | NA | NA | NA |
| Santos et al. (2019) | VR: 61.7±7.3 Stretch: 64.5±9.8 VR: 66.6±8.2 | VR: 13(11) Stretch: 14(11) VR: 14(9) | VR: 7±2.8 Stretch: 6.5±2.0 VR: 7.8±3.7 | VR: 1.4±0.6 Stretch: 1.3±0.3 VR: 1.5±0.4 | stable medication usage | ON | 8 | 2 | 50 | NA | NA | TUG, time | NA | BBS, score |
| Song et al. (2018) | VR: 68±7 CON: 65±7 | VR: 31(15) CON: 29(9) | VR: 7±4 CON: 9±6 | NA | Daily levodopa equivalent dose VR: 668±405 CON: 757±498 | ON | 12 | 3 | 15 | NA | 6m walking test, velocity | TUG, time | choice stepping reaction time, time | NA |
| Steib et al. (2017) | DT_BGT: 67.5 ±8.2 TT: 62.5±7.9 | DT_BGT: 18(11) TT: 20(16) | DT_BGT: 7.9±4.0 TT: 7.3±4.4 | DT_BGT: 2.6±0.5 TT: 2.5±0.5 | levodopa DT_BGT: 630.4±331.1 TT: 645.7±280.8 | ON | 8 | 2 | 40 | Mini-BESTest-sensory orientation | 6m walking test, velocity | TUG, time | Mini-BESTest-reactive balance | Mini-BESTest, score |
| Steib et al. (2019) | DT_BGT: 67.6±8.2 TT: 62.5±7.9 | DT_BGT: 18(11) TT: 20(16) | DT_BGT: 7.9±4.0 TT: 7.3±4.4 | DT_BGT: 2.6±0.5 TT: 2.5±0.5 | levodopa DT_BGT: 630.4±331.1 TT: 645.7±280.8 | ON | 8 | 2 | 40 | NA | 1 min walking test, velocity | NA | NA | NA |
| Strouwen et al. (2017) | BGT: 66.0±9.3 DT_BGT: 65.8±9.2 | BGT: 65(49) DT_BGT: 56(39) | BGT: 8.9±6.3 DT_BGT: 8.4±5.3 | BGT: 2.4±0.5 DT_BGT: 2.3±0.5 | levodopa BGT: 752.3±453.2 DT_BGT: 613.0±396.1 | ON | 6 | 4 | 40 | NA | 7.92m walking test, velocity | NA | NA | NA |
| Van Puymbroeck et al. (2018) | Yoga: 65.5±6.1 CON: 70.5±4.4 | Yoga: 15(10) CON: 12(7) | NA | NA | stable medication usage | ON | 8 | 2 | NA | NA | NA | NA | NA | Mini-BESTest, score |
| C.-M. Xiao et al. (2016) | CON: 66.5±2.1 Qigong: 68.1±2.3 | CON: 48(34) Qigong: 48(33) | CON: 6.2±2.6 Qigong: 5.5±3.6 | CON: 2.1±0.2 Qigong: 2.2±0.2 | stable medication usage | OFF | 24 | 4 | 50 | NA | walking test, velocity | TUG, time | NA | BBS, score |
| C. Xiao et al. (2016) | 67.8±9.4 | Qigong: 49 CON: 49 | NA | NA | stable medication usage | ON | 24 | 4 | 60 | NA | 6 min walking test, velocity | TUG, time | NA | BBS, score |
| W.-C. Yang et al. (2016) | VR: 72.5±8.4 BGT: 75.4±6.3 | VR: 11(7) BGT: 12(7） | VR: 9.4±3.6 BGT: 8.3±4.1 | VR: 3±0 BGT: 3±0 | stable medication usage | ON | 6 | 2 | 50 | NA | NA | TUG, time | NA | BBS, score |
| Y.-R. Yang et al. (2019) | DT_BGT: 65.0±57.5 DT_BGT: 69.5±65.0 BGT: 66.5±55.5 | DT_BGT: 6(4) DT_BGT: 6(4) BGT: 6(4) | DT_BGT: 5.5±2.8 DT_BGT: 5.0±0.1 BGT: 3.0±0.3 | DT_BGT: 2.0±1.6 DT_BGT: 2.0±1.8 BGT: 1.5±0.9 | LED, Levodopa equivalent dose DT_BGT: 892.0(432.2-1307.5) DT_BGT: 798.0(534.8-1074.2) BGT: 557(205.7-1234.7) | ON | 4 | 3 | 30 | NA | NA | TUG, time | NA | NA |
| Zhang et al. (2015) | TC: 66.0±11.8 Mul_C: 64.4±10.5 | TC: 20(13) Mul_C: 20(11) | TC: 6.8±5.4 Mul_C: 4.9±3.7 | TC: 2.0±0.5 Mul_C: 2.2±0.4 | Levodopa equivalent dose TC: 474.0±331.9 Mul_C: 389.3±322.8 | ON | 12 | 2 | 60 | NA | 10m walking test, velocity | TUG, time | NA | BBS, score |
| Daneshmandi et al. (2017) | Pilates: 57±6.2 CON: 58.3±7.4 | Pilates: 15(8) CON: 15(10) | Pilates: 7.3±3.8 CON: 8.2±3.1 | Pilates: 2.7±0.5 CON: 2.6±0.5 | stable medication usage | ON | 8 | 3 | 60 | NA | NA | TUG, time | NA | Fullerton Advanced Balance, score |
| Shih et al. (2016) | VR: 67.5±10.0 BGT: 68.8±9.7 | VR: 10(9) BGT: 10(7) | VR: 4.0±3.7 BGT: 5.2±4.9 | VR: 1.6±0.8 BGT: 1.4±0.5 | stable medication usage | ON | 8 | 2 | 50 | one leg stance with eyes open, time | NA | TUG, time | NA | BBS, score |
| Droby et al. (2020) | VR: 72.8±6.7 TT: 73.6±6.5 | VR: 18(11)  TT: 19(11) | VR: 8.4±6.0 TT: 9.6±6.6 | VR: 2.5±0.4 TT: 2.6±0.4 | levodopa equivalent daily dose VR: 770 ±563 TT: 1390±1434 | ON | 6 | 3 | NA | NA | 30m walking test, velocity | NA | NA | Mini-BESTest |
| Calabrò et al. (2019) | BGT_ECA: 70±8 TT: 73±8 | BGT_ECA: 25(11) TT: 25(14) | BGT_ECA: 10.0±3.0 TT: 9.3±3.0 | BGT_ECA: 3.0±1.0 TT: 3.0±1.0 | Levodopa  BGT_ECA: 450±55 TT: 435±49 | ON | 8 | 5 | 25 | NA | 10m walking test, velocity | TUG, time | NA | BBS, score |
| Tollár, Nagy, Kovács, et al. (2019) | Mul_C: 67.5±3.9 Mul_C: 67.6±3.3 CON: 67.6±4.1 | Mul_C: 19(11) Mul_C: 16(6) CON: 20(12) | Mul_C: 6.5±2.7 Mul_C: 6.8±1.8 CON: 7.1±2.8 | Mul_C: 2.5±0.5 Mul_C: 2.3±0.5 CON: 2.4±0.5 | L-dopa equivalent Mul_C: 774.2±381.5 Mul_C: 912.6±380.1 CON: 884.8±332.0 | ON | 3 | 5 | NA | narrow stance with eyes open, COP path | NA | TUG, time | NA | NA |
| Demonceau et al. (2017) | AE: 65±8 RT: 67±10 CON: 63.3±6 | AE: 16(12) RT:15(8) CON: 15(10) | AE: 5±4.07 RT: 7±5.08 CON: 5±2.96 | AE: 1.5±1.11 RT: 2±1.11 CON: 1.5±0.74 | AE: 402±240.7 RT: 594±517.03 CON: 381±439.25 | ON | 12 | 2-3 | 75 | NA | 30m walking test, velocity | TUG, time | NA | NA |
| L. Santos, J. Fernandez-Rio, K. Winge, B. Barragán-Pérez, L. González-Gómez, et al. (2017) | RT: 73.4±8.8 CON: 73.8±7.1 | RT: 13(5) CON: 15(10) | RT: 10.8±4.1 CON: 10.5±4.0 | RT: 1.9±0.5 CON: 1.9±0.4 | Levodopa dosage RT: 457.5±164 CON: 473.7±178 | ON | 8 | 2 | 75 | 30s bipedal standing with eyes open, COP Aera | 10m walking test, velocity | NA | NA | NA |
| Pérez de la Cruz (2017) | AQE: 66.8±5.3 CPP: 67.5 ±9.9 | AQE: 15(6) CPP: 15(7) | AQE: 6.2±2.5 CPP: 6.7 ±3.2 | AQE: 2.8±0.2 CPP: 2.7±1.0 | stable medication usage | OFF | 10 | 2 | 35 | NA | NA | TUG, time | NA | BBS, score |
| Galli et al. (2016) | RA_GT: 68.8 ±6.9  TT: 66.4±9.7 | RA_GT: 25(14) TT: 25(12) | RA_GT: 9.9 TT: 8.1 | NA | Levodopa equivalent dose RA_GT: 650.8±176.2 TT: 781.8±321.2 | ON | 4 | 5 | 45 | NA | 10m walking test, gait velocity | NA | NA | NA |
| Volpe et al. (2013) | Dance: 61.6±4.5 CPP: 65.0±5.3 | Dance: 12(7)  CPP: 12(6) | Dance: 9.0±3.6 CPP: 8.9±2.5 | Dance: 2.2±0.4 CPP: 2.2±0.4 | Levodopa Dance: 725.0 ±234 CPP: 645.0±216 | ON | 24 | 1 | 90 | NA | NA | NA | NA | BBS, score |
| Carda et al. (2012) | RA_GT: 67.8±7.1 TT: 66.9±5.1 | RA_GT: 15(NA) TT: 15(NA) | RA_GT: 3.7±2.5 TT: 3.7±1.9 | RA_GT: 2.2±0.2 TT: 2.2±0.3 | L-Dopa RA_GT: 393.8±165.7 TT: 371.4±99.4 | ON | 4 | 3 | 30 | NA | 10m walking test, velocity | TUG, time | NA | NA |
| Abraham et al. (2018) | Mul_D: 66.4±12.5 CON: 65.1±7.5 | Mul_D:10(9) CON:10(7) | Mul_D: 6.1±3.8 CON: 8.5±4.5 | Mul_D: 2.0±0.52 CON: 2.0±0.37 | stable medication use | ON | 2 | 5 | 120 | NA | 6m walking test, velocity | TUG, time | NA | Mini-BESTest |
| Carvalho et al. (2015) | AE: 64.8±11.9 RT: 64.1±9.9 BGT: 62.1±11.7 | AE: 5(4) RT: 8(6) BGT: 9(5) | AE: 6.6±1.5 RT: 6.0±2.6 BGT: 4.3±2.8 | AE: 2.6±0.5 RT: 2.1±0.6 BGT: 2.3±0.5 | stable medication use | ON | 12 | 2 | 40 | NA | 10m walking test, time | TUG, time | NA | BBS, score |
| Allen et al. (2010) | MUL_C: 66.0±10.0 CON: 68.0±7.0 | Mul_C: 24(13) CON: 24(13) | Mul_C: 7.0±5.0  CON: 9.0±6.0 | NA | stable medication use | ON | 24 | 3 | 50 | bipedal standing with eyes open, COP distance | 2.5m walking test, velocity | NA | NA | NA |
| Arcolin et al. (2016) | TT: 67.8±8.8 AE: 68.7±8.3 | TT: 13(6) AE: 16(9) | TT: 6.5±2.9 AE: 4.7±2.9 | TT: 2.3±0.5 AE: 2.3±0.5 | stable medication usage | ON | 3 | 5 | 60 | NA | 2m walking test, gait speed | TUG, time | NA | mini-BESTest, score |
| Arfa-Fatollahkhani et al. (2019) | CON: 61.55±8.57  TT: 60.63±9.36 | CON: 9(7) TT: 11(8) | CON: 8.50±6.34 TT: 8.89±5.14 | CON: 2.0±0.35 TT: 2.13±0.32 | Dopamine agonists CON: 1019.44±430.62 TT: 1000.0±474.34 | ON | 10 | 2 | 30 | NA | NA | TUG, time | NA | NA |
| Ashburn et al. (2007) | Mul_C: 72.7±9.6 CON: 71.6±8.8 | Mul_C: 70(38) CON: 72(48) | Mul_C: 7.7±5.8 CON: 9.0±5.8 | Mul_C: 3.14±0.59 CON: 3.09±0.55 | stable medication usage | ON | 6 | NA | 60 | NA | NA | Functional reach | NA | BBS, score |
| Atan et al. (2019) | TT: 69.7±8 BWS_TT 72.2±7.9 BWS_TT: 68.6±8.2 | TT: 10(3) BWS_TT: 10(4) BWS_TT: 10(4) | TT: 69.7±8 BWS_TT: 72.2±7.9 BWS_TT: 68.6±8.2 | TT: 2.6±0.7 BWS_TT: 2.8±0.6 BWS_TT: 2.7±0.7 | Levodopa equivalent dose TT: 698.1±207.2 BWS_TT: 696.5±195.5 BWS_TT: 884±253.6 | ON | 6 | 5 | 30 | NA | NA | NA | NA | BBS, score |
| Bang et al. (2017) | NW: 58.30±7.71 TT: 60.60±6.74 | NW: 10(5) TT: 10(4) | months NW: 18.10±6.77 TT: 17.98±3.28 | NW: 2.32±0.52 TT: 2.56±0.51 | stable medication use | ON | 4 | 5 | 60 | NA | 10m walking test, time | TUG, time | NA | BBS, score |
| Bello et al. (2013) | TT: 59.45±11.32 BGT_ECA: 58±9.38 | TT: 11(7) BGT_ECA: 11(5) | TT: 4.82±3.28 BGT_ECA: 4.95±2.59 | TT: 2.27±0.41 BGT_ECA: 2.05±0.52 | stable medication use | ON | 5 | 3 | 25 | 60s eyes open, COP Aera | 4 min walking test, velocity | TUG, time | NA | NA |
| Cakit et al. (2007) | 71.8±6.4 (n=31) | CON: 10(NA) TT: 21(NA) | 5.58±2.9 | NA | stable medication usage | ON | 8 | NA | 30 | NA | maximum speed on treadmill | NA | NA | BBS, score |
| Canning et al. (2012) | TT: 60.7±5.9  CON: 62.9±9.9 | TT: 10(5) CON: 10(6) | TT: 6.1±4.0  CON: 5.2±4.1 | 1-2 | levodope: 100 to 1200 mg/day; | ON | 6 | 4 | 35 | NA | 10m walking test, gait velocity | NA | NA | NA |
| Ebersbach et al. (2010) | Mul_C: 67.1±3.6 NW: 65.5 ±9.0 CON: 69.3 ±8.4 | Mul_C: 20(7) NW: 19(7) CON: 19(8) | Mul_C: 6.1±3.0 NW: 7.8±4.4 CON: 7.4±5.9 | Mul_C: 2.8±0.37 NW: 2.6±0.4 CON: 2.5±0.7 | LED, L-dopa equivalence dose. Mul_C: 486±301 NW: 530±288 CON: 463±260 | ON | 5 | 3 | 60 | NA | 10m walking test, time | TUG, time | NA | NA |
| Fernandes et al. (2015) | BGT: 62.3±12.9 DT_BGT: 63.4±9.5 | BGT: 8(6) DT_BGT: 7(5) | BGT: 7.7±7.5 DT_BGT: 8.8±4.3 | 3 | stable medication usage | ON | 6 | 2 | NA | leg stance with eyes opean, COP velocity | NA | TUG, time | NA | NA |
| Fisher et al. (2008) | TT: 63.1±11.5 CPP: 61.5±9.8 BWS_TT: 64.0±14.5 | TT: 10(8) CPP: 10(5) BWS_TT: 10(6) | months TT: 17.7±13.3 CPP: 8.8±7.9 BWS_TT: 14.7±9.9 | TT: 1.9±0.3 CPP: 1.9±0.3 BWS_TT: 1.9±0.5 | stable medication use | ON | 8 | 3 | 45 | NA | 10m walking test, gait velocity | NA | NA | NA |
| Capecci et al. (2019) | RA_GT: 68.1±9.8 TT: 67.0±7.6 | RA_GT: 48(19) TT: 48(24) | RA_GT: 8.9±5.3 TT: 8.9±4.3 | RA_GT: 3±0.5 TT: 3±0.5 | levodopa equivalent daily dose RA_GT: 739.8±328 TT: 739.4±301 dopamine-agonists-LEDD RA_GT 105.3±112 TT: 115.3±96 | ON | 4 | 5 | 45 | NA | 10m walking test, gait velocity | TUG, time | NA | NA |
| Carpinella et al. (2017) | DT_BGT: 73.0±7.1 CON: 75.6±8.2 | DT_BGT: 17(14) CON: 20(9) | DT_BGT: 7.5±3.2 CON: 10.3±5.7 | DT_BGT: 12.7±0.7 CON: 2.9±0.5 | stable medication usage | ON | 7 | 3 | 45 | one leg stance with open eyes CoP sway, distance | 10m walking test, gait velocity | TUG, time | NA | BBS, score |
| Cheng et al. (2016) | Mul_C: 66.4±7.8 DT_BGT: 65.8±11.5 Stretch: 67.3±6.4 | Mul_C: 12(8) DT_BGT: 12(9) Stretch: 12(8) | Mul_C: 6.5±2.4 DT_BGT: 6.1±4.1 Stretch: 8.1±4.6 | Mul_C: 2.25±0.58 DT_BGT: 2.17±0.62 Stretch: 2.21±0.66 | stable medication usage | ON | 5 | 2.5 | 30 | SOT Total, score | NA | NA | NA | Tinetti assessment scale, score |
| Cheng et al. (2017) | TT: 65.8 ± 11.5 CON: 67.3 ± 6.4 | TT: 12(9) CON: 12(8) | TT: 6.1 ± 4.1 CON: 8.1 ± 4.6 | TT: 1.8 ± 0.6 CON: 2.0 ± 0.8 | Daily levodopa dosage TT: 401.7 ± 199.4 CON: 420.8 ± 243.3 | ON | 5 | 2.5 | 40 | NA | 6m walking test, gait velocity | TUG, time | NA | NA |
| Chivers Seymour et al. (2019) | Mul_C: 71 ±7.7 CON: 73 ±7.7 | Mul_C: 238(147) CON: 236(119) | Mul_C: 8±6.6 CON: 8±5.8 | Mul_C: 2.59±2.67 CON: 0.86±0.89 | stable medication usage | ON | 26 | 7 | 30 | NA | NA | NA | NA | Mini-BESTest, score |
| Combs et al. (2013) | Mul_C: 68.0±7.75 AE: 66.5±7 | Mul_C: 14(10) AE: 17(11) | Mul_C: 12.5±5.6 AE: 10.4±9.1 | Mul_C: 2.0±0.75 AE: 2.0±0.75 | stable medication usage | ON | 12 | NA | 90 | NA | 4.8m walking test, gait velocity | TUG, time | NA | BBS, score |
| Rios Romenets et al. (2015) | CON: 64.3±8.1 Tango: 63.2±9.9 | CON: 15(7) Tango: 18(12) | CON 7.7±4.6 Tango: 5.5±4.4 | CON: 2.0±0.5 Tango: 1.7±0.6 | Levodopa: CON: 485±347.5 Tango: 450±349.7 | ON | 12 | 2 | 60 | NA | NA | TUG, time | NA | Mini-BESTest, score |
| Costa-Ribeiro et al. (2017) | Mul_C: 61.1±9.1 BGT_ECA: 62.0±16.7 | Mul_C: 11(8) BGT_ECA: 11(7) | Mul_C: 2.4±0.7 BGT_ECA: 2.3±0.4 | Mul_C: 6.1±3.8 BGT_ECA: 6.3±3.7 | Levodopa Mul_C: 740.9±924.3 BGT_ECA: 890.9±836.0 | ON | 4 | 3 | 43 | NA | 10m walking test, gait velocity | TUG, time | NA | BBS, score |
| Cugusi et al. (2015) | NW: 68.1 ± 8.7 CON: 66.6±7.3 | NW: 10( 8) CON: 10(8) | NW: 7±2 CON: 7±4 | NW: 2.4 ± 0.8 CON: 2.3 ± 0.5 | stable medication usage | ON | 12 | 2 | 60 | NA | NA | TUG, time | NA | BBS, score |
| de Lima et al. (2019) | CON: 67.2 ± 5.2 RT: 66.2 ± 5.5 | CON: 16(NA) RT: 17(NA) | NA | CON: 1.93±0.80 RT: 2.07±0.80 | stable medication usage | ON | 20 | 2 | 35 | NA | 10m walking test, gait velocity | TUG, time | NA | NA |
| Ebersbach et al. (2008) | WBV: 72.5±6.0 DT_BGT: 75.0±6.8 | WBV: 10(7) DT_BGT 11(7) | WBV: 7.0 ± 3.3 DT_BGT: 7.5 ± 2.7 | NA | levodopa WBV: 532.0±226.0 DT_BGT: 600.0±207.0 | ON | 4 | 5 | 150 | one leg stance with open eyes CoP sway, distance | 10m walking test, gait velocity | TUG, time | NA | Tinetti assessment scale, score |
| Serrao et al. (2019) | Mul_C: 68.9±8.6 CPP: 71.2±7.5 | Mul_C: 21(11) CPP: 19(11) | Mul_C: 9.0±4.9 CPP: 8.5±3.5 | Mul_C: 2.9±0.9 CPP: 2.9±1.2 | Mul_C: 593.7±331.5 CPP: 623.5±328.6 | ON | 8 | 3 | 60 | NA | walking test, velocity | NA | NA | NA |
| Giuseppe Frazzitta et al. (2009) | TT: 71±8 DT_BGT: 71±7 | TT: 20(8) DT_BGT: 20(9) | TT: 13.2±4.1 DT_BGT: 12.9±4.6 | 3 | TT: 685±246 DT_BGT: 720±232 | ON | 4 | 7 | 20 | NA | walking test, velocity | NA | NA | NA |
| G. Frazzitta et al. (2015) | BGT_ECA: 66.6±10.0  AE: 65.0±8.8 | BGT_ECA: 30(13) AE: 30(17) | NA | BGT_ECA: 2.8 ±0.4 AE: 2.8±0.4 | dopaminergic replacement therapy BGT_ECA:608.7 ±307.6 AE: 740.9 ±297.8 | ON | 4 | 6 | 35 | NA | NA | TUG, time | NA | BBS, score |
| Furnari et al. (2017) | RA_GT: 71.5 ±11.7  BGT: 77.7 ±8.3 | RA_GT:19(11) BGT:19(10) | NA | RA_GT: 3.1±0.9  BGT: 2.2±0.5 | stable medication usage | ON | 4 | 6 | 60 | NA | Tinetti Walking, score | NA | NA | Tinetti assessment scale, score |
| Gandolfi et al. (2019) | Mul_C: 72.42±6.4 CPP: 70.72±6.6 | Mul_C: 19(9) CPP: 18(15) | Mul_C: 8.01±5.9 CPP: 6.57±4.29 | ≤4 | Levodopa equivalent daily dose Mul_C: 803.31 ±405.81 CPP: 623.44±447.38 | ON | 4 | 5 | 60 | one leg stance with eyes open CoP sway, area | NA | NA | NA | Mini-BESTest, score |
| Ganesan et al. (2015) | 58.15 ± 8.7 | BWS_TT:20 (NA) BGT_ECA: 20 (NA) CON: 20 (NA) | NA | >3 | stable medication usage | ON | 4 | 4 | 30 | NA | 10m walking test, gait velocity | NA | NA | NA |
| Ghielen et al. (2017) | CPP: 66.6 ±8.4 Mul_D: 59.6±9.7 | CPP: 19(9) Mul_D: 19(7) | CPP: 12.3±4.3 Mul_D: 10.5±5.7 | 2-3 | CPP: 1340±534 Mul_D 1165±405 | ON | 6 | 2 | 60 | one leg stance with eyes open (right), time | 10m walking test, time | NA | NA | NA |
| Ginis et al. (2016) | NA | BGT_ECA: 20 BGT: 18 | NA | 2-3 | stable medication usage | ON | 6 | 3 | 30 | NA | 1min walking test, gait velocity | NA | NA | Mini-BESTest, score |
| Goodwin et al. (2011) | Mul_C: 72.0±8.6 CON: 70.1±8.3 | Mul_C: 64(39) CON: 66(35) | Mul_C: 9.1±6.4 CON 8.2±6.4 | Mul_C: 2.6 ±0.9 CON: 2.4 ±0.9 | stable medication usage | ON | 10 | 1 | 60 | NA | NA | TUG, time | NA | BBS, score |
| Grobbelaar et al. (2017) | BGT: 70 ± 11 DT_BGT: 72 ± 6 | BGT: 14(10) DT_BGT: 15(9) | BGT: 7 ± 6  DT_BGT: 5 ± 3 | BGT: 2.7 ± 0.5  DT_BGT: 2.7 ± 0.9 | stable medication usage | ON | 8 | 3 | 50 | NA | 10-m walk test, velocity (km/h) | NA | NA | NA |
| Hackney et al. (2007) | Tango: 72.6±2.2 RT: 69.6±2.1 | Tango: 9(6) RT: 10(6) | Tango: 6.2±1.5 RT: 3.3±0.5 | Tango: 2.3±0.7 RT: 2.2±0.6 | stable medication usage | ON | 13 | 2 | 60 | NA | 5m walking test, gait velocity | TUG, time | NA | BBS, score |
| Hackney et al. (2009) | Tango: 68.2±1.4  CON: 66.5±2.8 Dance: 66.8±2.4 | Tango: 14(11)  CON: 17(12)  Dance 17(11) | Tango: 6.9±1.3  CON: 5.9±1.0 Dance: 9.2±1.5 | Tango: 2.1±0.1  CON: 2.2±0.2 Dance: 2.0±0.2 | stable medication usage | ON | 13 | 2 | NA | NA | 5m walking test, gait velocity | TUG, time | NA | BBS, score |
| Harro et al. (2014) | BGT_ECA: 67.3±10.9 TT: 64.9±9.0 | BGT_ECA: 10(8) TT: 10(5) | BGT_ECA: 4.0±2.1 TT: 4.3±2.3 | BGT_ECA: 1.9±0.5 TT: 2.0±0.6 | stable medication usage | ON | 6 | 3 | 30 | SOT Total, score | NA | NA | NA | BBS, score |
| Hashimoto et al. (2015) | Dance: 67.9±7.0 CPP: 62.7±14.9 CON: 69.7±4.0 | Dance: 15(3) CPP: 17(2) CON: 14(7) | Dance: 6.3±4.6 CPP: 7.8±6.2 CON: 6.9±4.0 | Dance: 2.7±0.46 CPP: 2.7±0.47 CON: 3±0.56 | stable medication usage | ON | 12 | 1 | 60 | NA | NA | TUG, time | NA | BBS, score |
| Hass et al. (2012) | PRT: 64.0±7.0 Control: 67.0±8.0 | PRT: 9(7) Control: 9(7) | PRT: 11.1±9.8 Control: 6.4±2.5 | PRT: 2.3±0.6 Control: 2.3±0.7 | stable medication usage | ON | 10 | 2 | NA | NA | NA | COP, anticipatory postural adjustments phase posterior | NA | NA |
| Hubble et al. (2018) | Mul_C: 67.5±5.8 CON: 63.3±4.9 | Mul_C: 11(8) CON: 11(7) | Mul_C: 7.0 ±5.0 CON: 6.5±5.2 | Mul_C: 2.0±0.7  CON: 1.8±0.6 | Levodopa Daily Equivalent Dose (mg) Mul_C: 868.2±475.7 CON: 564.8±327.6 | ON | 12 | 1 | 90 | NA | NA | TUG, time | NA | NA |
| Stożek et al. (2016) | CPP: 64.0±9.9 CON: 67.0 ± 11.3 | CPP: 30(13) CON: 31(16) | CPP: 4.6 ± 2.7 CON: 4.3 ± 2.6 | CPP: 2.3 ± 0.6 CON: 2.3 ± 0.6 | stable medication usage | ON | 4 | 2.5 | 120 | tandem stance, time | 10m walking test, time | NA | pastor test, score | NA |
| Pérez-de la Cruz (2018) | AQE: 65.87 ± 7.090 CPP: 66.44 ± 5.726 | AQE: 14(5) CPP: 15(7) | > 2 years | NA | stable medication usage | OFF | 11 | 2 | 45 | single leg stand with open eyes, time | NA | TUG, time | NA | NA |
| Löfgren et al. (2019) | DT_BGT: 72.5±5.8 CON: 73.5±5.6 | DT_BGT: 45(27) CON: 42(21) | DT_BGT: 5.8±5.3 CON: 5.4±4.7 | DT_BGT: 2.5 ± 0.4 CON: 2.4 ± 0.4 | L-dopa equivalent DT_BGT: 591.8±287.1 CON: 639.5±422.2 | ON | 10 | 3 | 60 | NA | 8.3m walking test, gait velocity | NA | NA | NA |
| Silva et al. (2019) | AQE: 63.12 ± 13.61 CON: 64.23 ± 13.45 | AQE: 14(6) CON: 11(5) | NA | AUR: 3±1 CON: 3±1 | stable medication usage | ON | 10 | 2 | 60 | NA | NA | TUG, time | NA | BBS, score |
| Tollár, Nagy, and Hortobágyi (2019) | VR: 70.0±4.69 AE: 70.6±4.10 CON: 67.5±4.28 | VR: 25(12) AE: 25(11) CON: 24(13) | VR: 7.5±1.76 AE: 7.5±2.16 CON: 7.3±2.21 | VR: 2.3±0.48 AE: 2.4±0.51 CON: 2.4±0.51 | L-dopa equivalent VR: 805.2±130.83 AE: 786.4±120.93 CON: 825.4±126.55 | ON | 5 | 5 | 60 | tandem stance with eyes open, cop path | NA | NA | NA | Mini-BESTest, score |
| Yen et al. (2011) | VR: 70.4±6.5 DT_BGT: 70.1±6.9 CON: 71.6±5.8 | VR: 14(12) DT_BGT: 14(12) CON: 14(9) | VR: 6.0±2.9 DT_BGT: 6.1±3.3 CON: 7.8±4.2 | VR: 2.6±0.5 DT_BGT: 2.4±0.5 CON: 2.6±0.4 | stable medication usage | ON | 6 | 2 | 30 | Sensory orientation test, score | NA | NA | NA | NA |
| Rosenfeldt et al. (2019) | Mul_D: 65±8 DT_BGT: 59±9 | Mul_D: 10(9) DT_BGT: 10(5) | Mul_D: 4±2.2 DT_BGT: 8±5.9 | Mul_D: 2.2±0.4 DT_BGT: 2.4±0.5 | Levodopa Equivalent Daily Dose Mul_D: 550.0±254.8 DT_BGT: 437.5±378.7 | ON | 8 | 3 | 45 | NA | 2 min walking test, velocity | NA | NA | NA |
| Sale et al. (2013) | RA_GT: 70.3±9.8 DT_BGT: 68.4±9.4 | RA_GT: 10(6) DT_BGT: 10(5) | RA_GT: 8.4±5.0 DT_BGT: 8.7±4.7 | NA | stable medication usage | ON | 4 | 5 | 40 | NA | 10 meter walking test, velocity | NA | NA | NA |
| Pompeu et al. (2012) | 67.4±8.1 | 32(17) | NA | 1.7±0.5 | stable medication usage | ON | 7 | 2 | 60 | Unipedal Stance Test with eyes open | NA | NA | NA | Berg Balance Scale, score |
| Rennie et al. (2021) | DT_BGT: 73.1±5.8 CON: 73.0±5.5 | DT_BGT: 51(32) CON: 49(25) | DT_BGT: 5.9±5.1 CON: 5.6±4.8 | DT_BGT: 2.5±0.5 CON: 2.6±0.5 | DT_BGT: 578±299 CON: 640±380 | ON | 10 | 3 | 60 | NA | Mean step velocity | NA | NA | NA |
| Schabrun et al. (2016) | Mul_D: 72±4.9 DT_BGT: 63.0±11.0 | Mul_D: 8(8) DT_BGT: 8(2) | Mul_D: 6.9±4.4 DT_BGT: 4.6±3.9 | Mul_D: 2.0±1.5 DT_BGT: 2.0±1.5 | Levodopa equivalent daily dose Mul_D: 730±341 DT_BGT: 523±398 | ON | 3 | 3 | 60 | NA | Gait speed | NA | NA | NA |
| I. S. K. Wong-Yu et al. (2015) | Mul_C: 59.4±9.0 CON: 62.6±8.9 | Mul_C: 41(25) CON: 39(21) | Mul_C: 7.1±4.3 CON: 5.6±3.8 | Mul_C: 2.5±0.3 CON: 2.4±0.3 | Daily levodopa equivalent dosage Mul_C: 409.3±288.2 CON: 320.8±391.0 | ON | 8 | 1 | 120 | sensory orientation test, score | 7m walking test, Gait speed | BESTest-Transitions/anticipatory, score | NA | BESTest total, score |
| Shen et al. (2015) | VR: 63.3±8.0 RT: 65.3±8.5 | VR: 22(13) RT: 23(12) | VR: 8.1±4.3 RT: 6.6±4.0 | VR: 2.5±0.5 RT: 2.5±0.5 | levodopa VR: 505.1±347.4 RT: 618.1±619.6 | ON | 12 | 4 | 80 | single leg stand, time | Gait velocity | NA | Latency of postural response | NA |
| Volpe, Giantin, and Fasano (2014) | BGT_ECA: 66.5±10.4 BGT: 69.5±6.5 | BGT_ECA: 20(7) BGT: 20(9) | BGT_ECA: 6.0±5.0 BGT: 6.5±3.7 | BGT_ECA: 3.0±0.0 BGT: 3.0±0.7 | L-dopa LEDD  BGT_ECA: 487.5±277.8 BGT: 450.0±226.8 | ON | 8 | 5 | 60 | sway area,mm2 | NA | TUG, time | NA | Berg Balance Scale score |
| Thaut et al. (1996) | BGT_ECA: 69.0±8.0 CON: 71.0±8.0  BGT: 74.0±3.0 | BGT_ECA: 15(10) CON: 11(8) BGT: 11(8) | BGT_ECA: 7.2±4.0 CON: 8.5±4.0 BGT: 5.4±3.0 | BGT_ECA: 2.4±NA CON: 2.6±NA BGT: 2.5±NA | stable medication usage | ON | 3 | NA | 30 | NA | Gait Velocity (m/min) | NA | NA | NA |
| Sage et al. (2009) | BGT_ECA: 64.2±10.3 AE: 65.1±9.3 CON: 68.6±8.7 | BGT_ECA: 18(12) AE: 13(6) CON: 15(7) | BGT_ECA: 4.7±4.9 AE: 3.2±2.9  CON: 2.5±2.2 | NA | stable medication usage | ON | 12 | 3 | 50 | NA | 4m walking test, Velocity | TUG, time | NA | NA |
| Kadivar et al. (2011) | BGT_ECA: 73.3±2.2 BGT: 70.5±2.2 | BGT_ECA: 8(5) BGT: 8(6) | BGT_ECA: 8.9±1.8 BGT: 7.5±1.2 | BGT_ECA: 2.69±0.56 BGT: 2.69±0.56 | levodopa BGT_ECA: 456.25±317.8 BGT: 509.4±207.0 | ON | 6 | 3 | 50 | NA | NA | TUG, time | NA | Tinetti-gait and balance tests, score |
| Bakhshayesh et al. (2017) | Pilates: 57.0±6.24  BGT: 58.31±7.37 | 30(18) | Pilates: 7.27±3.80  BGT: 8.19±3.14 | 2-3 | stable medication usage | OFF | 8 | 3 | 60 | NA | NA | NA | NA | Fullerton advanced balance, score |
| Picelli et al. (2012) | 68.3 | RA_GT: 16(NA) Stretch: 15(NA) | 7.5 | 3.45 | stable medication usage | ON | 4 | 3 | 40 | NA | 10m walking test, time | TUG, time | Nutt’s rating, score | BBS, score; |
| Picelli et al. (2013) | RA_GT: 68.50±10.10 TT: 68.80±7.72 BGT: 67.55±7.08 | RA_GT: 20(9) TT: 20(6) BGT: 20(8) | RA_GT: 6.52±5.30 TT: 6.99±6.17 BGT: 6.79±6.30 | 3 | stable medication usage | ON | 4 | 3 | 45 | NA | 10m walking test, velocity | NA | NA | BBS, score |
| Picelli et al. (2015) | RA_GT: 68.2±9.2 DT_BGT: 69.7±7.2 | RA_GT: 33(26) DT_BGT: 33(22) | RA_GT: 7.5±5.6 DT_BGT: 8.3±4.1 | NA | stable medication usage | ON | 4 | 3 | 45 | NA | NA | TUG, time | NA | BBS, score |
| Solla et al. (2019) | Dance: 67.8±5.9 CON: 67.1±6.3 | Dance: 10(6) CON: 10(7) | Dance: 4.4±4.5 CON: 5.0±2.9 | Dance: 2.1±0.6 CON: 2.3±0.4 | levodopa  Dance: 481.1±213.1 CON: 487.5±198.5 | ON | 12 | 2 | 90 | NA | 7m walking test, velocity | TUG, time | NA | BBS, score |
| Michels et al. (2018) | Tango: 66.44±NA CON: 75.50±NA | Tango: 9(NA) CON: 4(NA) | NA | Tango: 2.11±0.33 CON: 2.50±1.00 | stable medication usage | ON | 10 | 2 | 60 | NA | NA | TUG, time | NA | BBS, score |
| Kunkel et al. (2017) | Dance: 71.3±7.7 CON: 69.7±6.0 | Dance: 36(19) CON: 15(6) | Dance: 4.7±3.5 CON: 7.0±4.9 | Dance: 2.11±0.84 CON: 2.13±0.72 | Dance: 5.8±2.7 CON: 4.7±3.0 | ON | 10 | 2 | 60 | NA | NA | TUG, time | NA | BBS, score |
| Smania et al. (2010) | DT_BGT: 67.64±7.41 Stretch: 67.26±7.18 | DT_BGT: 28（14） Stretch:27（15） | DT_BGT: 10.39±4.76 Stretch: 8.63±5.39 | DT_BGT: 14.6±5.9 Stretch: 3.1±0.3 | stable medication usage | ON | 7 | 3 | 50 | center of foot pressure, score | NA | NA | NA | BBS, score |
| van der Kolk et al. (2019) | AE: 59.3±8.3 Stretch 59.4±9.3 | AE: 65(42) Stretch: 65(38) | AE: 3.4±4.4 Stretch: 3.1±3.8 | AE: 1.94±0.24 Stretch: 1.95±0.21 | levodopa  AE: 600±128.75 Stretch: 532±134.50 | ON | 24 | 3 | 30-45 | NA | NA | TUG, time | NA | Mini-BESTest, score |
| Sacheli et al. (2019) | AE: 66.76±5.98 Stretch: 67.85±8.50 | AE: 20(13) Stretch: 15(9) | AE: 3.91±2.85  Stretch: 5.17±4.26 | 1-3 | stable medication usage | OFF | 12 | 3 | 40-60 | NA | NA | TUG, time | NA | NA |
| Shulman et al. (2013) | TT: 66.1±9.7 TT: 65.8±11.5 RT: 65.3±11.3 | TT: 23(16) TT: 22(16) RT: 22(18) | TT: 5.9±3.9 TT: 6.3±3.5 RT: 6.3±4.0 | TT: 2.15±0.34 TT: 2.16±0.35 RT: 2.23±0.39 | stable medication usage | ON | 12 | 3 | 45 | NA | 10m walking test, time | TUG, time | NA | NA |
| Khalil et al. (2017) | Mul_C: 58.4±13.5 CON: 60.7±15.4 | Mul_C: 16(12) CON: 14(7) | Mul_C: 8.0±6.4 CON: 7.5±4.0 | Mul_C: 2.4±0.72 CON: 2.2±0.8 | stable medication usage | ON | 8 | 3 | 45 | NA | 10m walking test, gait velocity | NA | NA | Mini-BESTest, score |
| Kurt et al. (2018) | AQE: 62.41 ± 6.76 Mul_C: 63.61 ± 7.18 | AQE: 20(11) Mul_C: 20(13) | NA | AQE: 2.37±0.39 Mul_C: 2.32±0.40 | Daily Levodopa equivalent dose AQE: 764.36 ± 96.09 Mul_C: 742.67 ± 113.64 | ON | 5 | 5 | 60 | 25s bipedal stance with open eyes, score | NA | TUG, time | NA | BBS, score |
| Kurtais et al. (2008) | TT: 63.8±10.6 CON: 65.7±5.3 | TT: 12(5) CON: 12(7) | TT: 5.3±0.8 CON: 5.4±1.2 | TT: 2.5±0.7 CON: 2.2±0.8 | stable medication usage | ON | 6 | 3 | 40 | single leg stand with open eyes, time | 20m walking test, gait velocity | TUG, time | NA | NA |
| Landers et al. (2016) | BGT_ECA: 72.2±4.4 BGT_ICA: 70.2±4.4 BGT: 70.1± 9.5 CON: 74.3±8.8 | BGT_ECA: 10(4) BGT_ICA: 11(8) BGT: 10(7) CON: 10(6) | NA | BGT_ECA: 2.25±0.86 BGT_ICA: 2.75±0.75 BGT: 2.45±0.44 CON: 2.75±0.63 | stable medication usage | ON | 4 | 3 | 45 | Sensory Organization Test | Self-Selected Gait Velocity | NA | NA | Berg Balance Scale, score |
| Leal et al. (2019) | CON: 64.9±2.32  RT: 65.2±2.05 | CON: 27(13)  RT: 27(14) | NA | CON: 2±0.5  RT: 2±0.5 | stable medication usage | ON | 24 | 2 | 32.5 | NA | 6-m walking speed test, velocity | TUG, time | NA | Tinetti mobility test, score |
| Lee et al. (2018) | Qigong: 65.8±7.2 CON: 65.7±6.4 | Qigong: 25(10) CON: 16(7) | Qigong: 4.5±3.3 CON: 4.4±3.0 | Qigong: 2.0±0.7 CON: 1.8±0.8 | stable medication usage | ON | 8 | 2 | 60 | NA | NA | NA | NA | Berg Balance Scale, score |
| Granziera et al. (2021) | NW: 64.9±10.2 BGT: 70.5±5.8 | NW: 16(13) BGT: 17(7) | NW: 5.5±3.3 BGT: 5.09±4.1 | NW: 1.5±0.5 BGT: 2.0±1.0 | stable medication usage | ON | 6 | 2 | 60 | NA | NA | TUG, time | NA | NA |
| Monticone et al. (2015) | Mul_D: 74.1±6.0 CPP: 73.4±7.0 | Mul_D: 35(24) CPP: 35(22) | Mul_D: 15.7±2.6 CPP: 15.3±3.0 | Mul_D: 3.08±0.50 CPP: 3.07±0.47 | Daily Levodopa equivalent dose Mul_D: 928.7±86.7 CPP: 937.6±91.1 | ON | 8 | 2 | 90 | NA | NA | NA | NA | Berg Balance Scale, score |
| Morris et al. (2015) | RT: 67.4±10.4 BGT_ECA: 68.4±9.9 CON: 67.9±8.4 | RT: 70(42) BGT_ECA 69(46) CON: 71(52) | RT: 7.2±6.2 BGT_ECA: 6±5.5 CON: 6.9±5.2 | RT: 2.39±0.77 BGT_ECA: 2.40±0.81 CON: 2.61±0.90 | stable medication usage | ON | 8 | 1 | 120 | NA | 6-m walk test, velocity | TUG, time | NA | NA |
| Ortiz-Rubio et al. (2018) | RT: 74.2±5.8 CON: 75.4±6.5 | RT: 23(NA) CON: 23(NA) | RT: 4.0±2.2 CON: 4.3±2.0 | RT: 2.5±0.5 CON: 2.4±0.5 | Levodopa:  "RT: 683.6±316.2 CON: 743.1±256.8" | ON | 8 | 2 | 60 | Mini-BESTest,Sensory orientation | Mini-BESTest, Dynamic gait | Mini-BESTest, Anticipatory | Mini-BESTest, Reactive postural control | Mini-BESTest, score |
| Palamara et al. (2017) | AQE: 70.9±5.7 Mul_C: 70.8±5.3 | AQE: 17(9) Mul_C: 17(11) | NA | AQE: 2.8±0.5 Mul_C: 3.1±0.2 | Levodopa AQE: 583.2±235.3 Mul_C: 720.4±269.9 | ON | 4 | 4 | 60 | NA | NA | TUG, time | NA | BBS, score |
| Park et al. (2014) | RT: 60.1±6.6 CON: 59.8±6.3 | RT: 15(10) CON: 16(10) | NA | NA | stable medication usage | ON | 48 | 3 | 60 | NA | NA | TUG, time | NA | Tinetti mobility test, score |
| Paul et al. (2014) | PT: 68.1±5.6 CON: 64.5±7.4 | PT: 20(13) CON: 20(12) | PT: 7.8±5.2 CON: 7.8±5.9 | PT: 2.0±0.7 CON: 1.9±0.9 | stable medication usage | ON | 12 | 2 | 45 | single leg stand with open eyes, time | 10m walking test, gait velocity | FR; maximal reach distance; | choice stepping reaction time | NA |
| Picelli et al. (2016) | TT: 71.2±9.2 CON: 71.6±7.2 | TT: 9(5) CON: 8(4) | TT: 11.2±5.6 CON: 10.8±4.1 | NA | stable medication usage | ON | 4 | 3 | 45 | NA | 10m walking test, time | NA | NA | NA |
| Poliakoff et al. (2013) | Mul_C: 68.8±7.3 CON: 66.6±7.3 | Mul_C: 12(9) CON: 10(8) | Mul_C: 7.9±3.0 CON: 4.6±3.9 | NA | stable medication usage | ON | 10 | 1 | 60 | NA | NA | TUG, time | NA | NA |
| Prodoehl et al. (2015) | CPP: 58.6±5.6 RT: 59.0±4.6 | CPP: 24 (14) RT: 24(14) | CPP: 6.5±4.7 RT: 6.5±4.1 | CPP: 1.9±0.3 RT: 2.0±0.4 | Levodopa equivalent CPP: 705±405 RT: 598±355 | ON/OFF | 96 | 2 | 75 | NA | 50-ft Walk Test, velocity(m/s) | FR; maximal reach distance; | NA | BBS, score |
| Qutubuddin et al. (2007) | CON 71.89±8.46 BGT: 73.67±5.13 | CON: 9(NA) BGT: 6(NA) | NA | NA | stable medication usage | ON | 4 | 2 | 30 | SOT Total, score | NA | NA | NA | BBS, score |
| Rafferty et al. (2017) | RT: 59.0±4.6 CPP: 58.6±5.6 CON: 61.2±7.7 | RT: 24(14) CPP: 24(14) CON: 23(12) | NA | NA | stable medication usage | ON/OFF | 96 | 1.5 | 75 | NA | walking test, step velocity | NA | NA | NA |
| M. Schenkman et al. (1998) | CPP: 70.6±6.2 CON: 71.2±27.3 | CPP: 23(18) CON: 23(16) | NA | CPP: 2.7±0.7 CON: 2.5±0.6 | stable medication usage | ON | 10 | 3 | NA | NA | 10m walking test, time | FR; maximal reach distance | NA | NA |
| Schlick et al. (2016) | BGT_ECA: 71.2±10.9  TT: 68.9±6.8 | BGT_ECA: 10(2) TT: 10(4) | BGT_ECA: 10.4±5.2 TT: 9.1±3.1 | BGT_ECA: 2.8±0.9 TT: 2.7±0.7 | stable medication usage | ON | 5 | 2.5 | 35 | NA | walking test, velocity (m/s) | TUG, time | NA | NA |
| Shen et al. (2012) | BGT_ECA: 63.0±8.5  RT: 66.5±8.6 | BGT_ECA: 14(9) RT: 14(7) | BGT_ECA: 7.1±3.2 RT: 5.8±2.2 | BGT_ECA: 2.2±0.5 RT: 2.3±0.5 | Daily levodopa dosage, mg BGT_ECA: 267.0±177.2 RT: 289.3±249.7 | ON | 4 | 3 | 60 | NA | 5-m walk test, velocity(cm/s) | NA | NA | NA |
| Silva-Batista et al. (2017) | CON: 64.2±8.3  RT: 64.1±9.1  RT: 64.2±10.6 | CON: 13(9)  RT: 13(10)  RT: 13(10) | CON: 10.7±6.1  RT: 9.6±3.9  RT: 10.5±4.1 | CON: 2.5±0.4  RT: 2.5±0.5  RT: 2.5±0.4 | L-Dopa equivalent  CON: 796.7±151.3  RT: 835.8±287.0  RT: 875.9±223.4 | ON | 12 | 2 | 60 | NA | NA | TUG, time | NA | NA |
| Silva-Batista et al. (2018) | CON: 64.2±8.3  RT: 64.1±9.1  RT: 64.2±10.6 | CON: 13(9)  RT: 13(10)  RT: 13(10) | CON: 10.7±6.1  RT: 9.6±3.9  RT: 10.5±4.1 | CON: 2.5±0.4  RT: 2.5±0.5  RT: 2.5±0.4 | L-Dopa equivalent  CON: 796.7±151.3  RT: 835.8±287.0  RT: 875.9±223.4 | ON | 12 | 2 | 60 | BESTest, Sensory orientation (%) | BESTest, Stability in gait | BESTest, Anticipatory | BESTest, Reactive postural responses (%) | BESTest total score (%) |
| Silva-Batista et al. (2016) | CON: 64.2±8.3  RT: 64.1±9.1  RT: 64.2±10.6 | CON: 13(9)  RT: 13(10)  RT: 13(10) | CON: 10.7±6.1  RT: 9.6±3.9  RT: 10.5±4.1 | CON: 2.5±0.4  RT: 2.5±0.5  RT: 2.5±0.4 | L-Dopa equivalent  CON: 796.7±151.3  RT: 835.8±287.0  RT: 875.9±223.4 | ON | 12 | 2 | 60 | NA | NA | TUG, time | NA | NA |
| Stuckenschneider et al. (2015) | AE: 71.0±4.6 CON: 71.5±5.7  AE: 71.4±4.9 | AE: 10(4) CON: 10(4) AE: 12(5) | NA | AE: 3±0.4 CON: 3.0±3 AE: 3.0±3 | stable medication usage | ON | 12 | 3 | 40 | NA | walking test, velocity (km/h) | NA | NA | NA |
| van den Heuvel et al. (2014) | VR: 66.3±6.39 DT_BGT: 68.8±9.68 | VR: 17(12) DT_BGT: 16(8) | VR: 9.0±2.3 DT_BGT: 8.8±2.3 | VR: 2.5±0.4 DT_BGT: 2.5±0.4 | stable medication usage | ON | 5 | 2 | 60 | single leg stand with open eyes, time | 10m walking test, velocity | FR; maximal reach distance | NA | BBS, score |
| van der Kolk et al. (2018) | NA | AE: 22(NA) CON: 15(NA) | NA | NA | stable medication usage | OFF | 24 | 3 | 30 | NA | NA | TUG, time | NA | NA |
| Vivas et al. (2011) | AQE: 65.67±3.67 CPP: 68.33±6.92 | AQE: 6(3) CPP: 6(4) | AQE: 4.17±1.6 CPP: 7.83±3.92 | AQE: 2.67±0.58 CPP: 2.4±0.55 | stable medication usage | OFF | 4 | 2 | 45 | NA | NA | NA | NA | BBS, score |
| Volpe, Giantin, Maestri, et al. (2014) | AQE: 68 ± 7 DT_BGT: 66 ± 8 | AQE:17(NA) DT_BGT:17(NA) | AQE: 7.5 ± 5.1 DT_BGT: 7.6 ± 4.63 | AQE: 2.82 ± 0.3 DT_BGT: 2.65 ± 0.49 | L-dopa-equivalent  AQE: 645.4 ± 206 DT_BGT: 625.2 ± 244.3 | ON | 8 | 5 | 60 | cop, aera | NA | TUG, time | NA | BBS, score |
| Volpe et al. (2017) | AQE: 70.6 ± 7.8  Mul_C: 70 ± 7.8 | AQE: 15(9) Mul_C: 15(10) | AQE: 9.4 ± 7.5  Mul_C: 9 ± 7.0 | AQE: 2.6 ± 0.5 Mul_C: 2.7 ± 0.5 | L-dopa-equivalent  AQE: 437.2 ± 179.9  Mul_C: 353.1 ± 280.9 | ON | 8 | 5 | 60 | NA | NA | TUG, time | NA | BBS, score |
| White et al. (2009) | CON: 66.0±8.4  Mul_C: 65.6±9.2  Mul_C: 68.2±9.8 | CON: 25(17) Mul_C: 21(15) Mul_C: 28(20) | CON: 5.6±3.5  Mul_C: 5.1±4.1 Mul_C: 6.5±4.3 | CON: 2.3±0.4 Mul_C: 2.3±0.4 Mul_C: 2.3±0.3 | stable medication usage | ON | 6 | 2 | 90 | NA | 10s walking test, m/s | NA | NA | NA |
| I. S. Wong-Yu et al. (2015) | DT_BGT: 60.2±9.0  Stretch: 61.9±8.5 | DT_BGT: 32(19) Stretch: 36(20) | DT_BGT: 7.3±4.6  Stretch: 5.4±3.6 | DT_BGT: 2.5±0.3  Stretch: 2.4±0.3 | levodopa DT_BGT: 362.9±260.8  Stretch: 294.7±362.7 | ON | 8 | NA | 120 | single leg stance with open eyes, time | NA | FR; maximal reach distance; | NA | Mini-BESTest, score |
| Y.-R. Yang et al. (2010) | DT_BGT: 68.07±7.51 CPP: 66.27±10.72 | DT_BGT: 15(9) CPP: 15(7) | DT_BGT: 4.77±4.83 CPP: 5.27±5.55 | DT_BGT: 2.23±0.53 CPP: 2.17±0.72 | Levodopa DT_BGT: 440.0±186.5 CPP: 571.6±250.1   Ropinirole  DT_BGT: 4.3±2.3 CPP: 4.0±1.7 | ON | 4 | 3 | 30 | NA | 3.66m walking test, velocity | NA | NA | NA |
| Ellis et al. (2005) | Mul_C: 64±8.4  CON: 63±8.8 | Mul_C: 35(25) CON: 33(26) | NA | Mul_C: 2.5±0.5 CON: 2.4±0.5 | stable medication usage | ON | 6 | 2 | 90 | NA | Walking Test, velocity(m/s) | NA | NA | NA |
| Morris et al. (2009) | BGT_ECA: 72.5±5.8 Mul_C: 73.5±5.7 | BGT_ECA: 14(NA) Mul_C: 14(NA) | NA | NA | stable medication usage | ON | 2 | 8 | 40 | NA | 10m walking test, velocity | TUG, time | balance pull test, score | NA |
| Schilling et al. (2010) | RT: 61.3±8.6 CON: 57.0±7.1 | RT: 8(5) CON:7 (4) | NA | RT: 2 ±0.1 CON: 1.9 ±0.3 | stable medication usage | ON | 8 | 2 | NA | NA | NA | TUG, time | NA | NA |
| de Bruin et al. (2010) | BGT_ECA: 64.1± 4.2 CON: 67±8.1 | BGT_ECA: 11(6)  CON: 11(5) | BGT_ECA: 6.4± 4.2 CON: 4.5±3.3 | BGT_ECA: 2.3± 0.4 CON: 2.1±0.4 | stable medication usage | ON | 13 | 3 | 30 | NA | walking test, velocity | NA | NA | NA |
| Silva-Batista et al. (2020) | RT: 64.6±10.5 CPP: 66.8±8.9 | RT: 17(12) CPP: 15(9) | RT: 7.7±4.0 CPP: 10.0±5.6 | RT: 3.1±0.3 CPP: 3.2±0.4 | Levodopa RT: 437.5±211.7 CPP: 503.3±185.6 | ON | 12 | 3 | 85 | NA | NA | anticipatory postural adjustment, amplitude | NA | NA |
| Vieira-Yano et al. (2021) | CPP: 66.8±9.0 RT: 64.6±10.6 | CPP: 15(9) RT: 17(12) | CPP: 10.0±5.6 RT: 7.7±4.0 | CPP: 3.2±0.4 RT: 3.1±0.3 | Levodopa CPP: 503.3±185.6 RT: 437.5±211.7 | ON | 12 | 3 | 85 | NA | walking test, velocity | NA | NA | NA |
| Cabrera-Martos et al. (2020) | RT: 77.2±6.2 Stretch: 75.9±1.2 | RT: 22(15) Stretch: 22(11) | NA | 2-3 | Levodopa RT: 278.7±132.5 Stretch: 334.1±86.4 | ON | 8 | 3 | 45 | Mini-BESTest-sensory orientation, score | Mini-BESTest-dynamic gait, score | Mini-BESTest-anticipatory, score | Mini-BESTest-reactive postural control, score | Mini-BESTest, score |
| Sedaghati et al. (2018) | BGT: 64.9±2.6 CON: 63.2±3.3 | BGT: 13(8) CON: 13(6) | BGT: 5.3±1.8 CON: 4.3±0.7 | BGT: 2.6±0.5 CON: 2.7±0.5 | stable medication usage | ON | 8 | 3 | 60 | NA | NA | FR; maximal reach distance | NA | NA |
| Cancela et al. (2020) | AQE: 67.7±4.6 Mul_C: 69.2±4.4 | AQE: 7(6) Mul_C: 5(3) | NA | AQE: 2.3±0.8 Mul_C: 2.2±0.5 | stable medication usage | ON | 8 | 3 | 50 | NA | NA | TUG, time | NA | Tinetti assessment scale, score |
| Capato, de Vries, et al. (2020) | BGT_ECA: 74±8 BGT: 67±13 CON: 73±10 | BGT_ECA: 56(27) BGT: 50(32) CON: 48(29) | BGT_ECA: 5±5.2 BGT: 6±5.9 CON: 8±9.6 | BGT_ECA: 2.3±0.8 BGT: 2.2±0.8 CON: 2.3±0.7 | Levodopa BGT_ECA: 615±424 BGT: 701±466 CON: 698±389 | ON | 5 | 2 | 45 | NA | NA | TUG, time | NA | Mini-BESTest, score |
| Capato, Nonnekes, et al. (2020) | BGT_ECA: 77±7 BGT: 78±10 | BGT_ECA: 17(9) BGT: 18(12) | BGT_ECA: 17±9 BGT: 11±4 | NA | Levodopa BGT_ECA: 749±381 BGT: 869±327 | ON | 5 | 2 | 45 | NA | NA | TUG, time | NA | BBS, score |
| Gao et al. (2014) | TC: 69.5±7.3 CON: 68.3±8.5 | TC: 37(23) CON: 39(27) | TC: 9.2±8.6 CON: 8.4±8.2 | TC: 2.4±0.5 CON: 2.4±0.7 | stable medication usage | ON | 12 | 3 | 60 | NA | NA | TUG, time | NA | BBS, score |
| Johansson et al. (2020) | DT_BGT: 72±13.3 CON: 67.5±5.2 | DT_BGT: 7(6)  CON: 6(3) | DT_BGT: 10±7.4 CON: 7±5.9 | DT_BGT: 2±NA CON: 2.5±NA | levodopa equivalent dose  DT_BGT: 700±400 CON: 765.5±478.5 | ON | 10 | 2 | 60 | NA | walking test, velocity | NA | NA | Mini-BESTest, score |
| Joseph et al. (2019) | DT_BGT: 73.1±5.8  CON: 73.0±5.5 | DT_BGT: 51(32) CON: 49(25) | DT_BGT: 5.9±5.1 CON: 5.6±4.8 | DT_BGT: 2.55±0.5 CON: 2.57±0.5 | levodopa equivalent dose DT_BGT: 578±299 CON: 640±380 | ON | 10 | 3 | 60 | NA | walking test, velocity | NA | NA | Mini-BESTest, score |
| King et al. (2013) | Mul_C:65.7 ± 8.3 TT: 65.1 ± 7.3 | Mul_C: 20(12) TT: 19(13) | NA | Mul_C: 2.5 ± 0.8 TT: 2.4 ± 0.6 | stable medication usage | ON | 4 | 4 | 75 | Sway range | 7 meters away, Stride velocity | NA | NA | mini-BESTest, score |
| Maidan et al. (2018) | TT: 73.1 ± 1.1 VR: 70.1 ± 1.3 | TT: 34(23) VR: 30(22) | TT: 9.7 ± 1.0 VR: 8.9 ± 1.1 | 2-3 | levodopa equivalent dose TT: 1186 ± 238 VR: 833 ± 102 | ON | 6 | NA | 45 | NA | Gait speed, m\s | NA | NA | NA |
| McNeely et al. (2015) | Dance: 68.25 ± 10.90 Tango: 67.66 ± 8.62 | Dance: 8(4) Tango: 8(4) | Dance: 10.06 ± 4.14 Tango: 5.38 ± 4.83 | Dance: 2.25 ± 0.27 Tango: 2.13 ± 0.58 | stable medication usage | OFF | 12 | 2 | 50 | NA | walking test, velocity | TUG, time | NA | Mini-BESTest, score |
| Pazzaglia et al. (2020) | VR: 72±7 CPP: 70±10 | VR: 25(18) CPP: 26(17) | months VR: 89±92 CPP: 57±53 | NA | stable medication usage | ON | 6 | 3 | 40 | NA | NA | NA | NA | BBS, score |
| Michels et al. (2018) | Tango: 70.2±5.5 Dance: 72.9±5.5 | Tango: 10(NA) Dance: 11(NA) | Tango: 7.2±4.9 Dance: 8.4±5.2 | Tango: 2.5±0.5 Dance: 2.5±0.62 | stable medication usage | ON | 8 | 1 | 60 | NA | NA | TUG, time | NA | BBS, score |
| Jung et al. (2020) | Mul_C: 67.7±6.7 CON: 70.0±8.2 | Mul_C: 44(30) CON: 42(28) | Mul_C: 6.2±4.4 CON: 6.7±5.5 | Mul_C: 2.11±0.44 CON: 2.42±0.76 | stable medication usage | OFF | 6 | 3 | 80 | sensory organization test, score | walking test, score | Anticipatory Postural Adjustment | Automatic Postural Response | mini-BESTest, score |
| Schlenstedt et al. (2018) | RT: 78.3±5.8 BGT: 81.4±7.3 | RT: 12(9) BGT: 8(6) | RT: 11.2±6.6 BGT: 8.4 ±7.3 | RT: 2.8±0.3 BGT: 2.9±0.5 | Levodopa therapy RT: 765±448 BGT: 652±286 | ON | 7 | 2 | 60 | NA | NA | NA | NA | Fullerton Advanced Balance, score |
| Stack et al. (2012) | CON: 74±5.92 BGT_ECA: 75±5.92 | CON: 23(18) BGT_ECA: 24(17) | CON: 7±5.92 BGT_ECA: 8±5.18 | CON: 3±0.85 BGT_ECA: 3.13±0.69 | stable medication usage | ON | 4 | 3 | 60 | NA | NA | FR; maximal reach distance | NA | NA |
| Nieuwboer et al. (2007) | BGT_ECA: 67.5±7.8 CON: 69±7.8 | BGT_ECA: 76(48) CON: 77(40) | BGT_ECA: 7±5.2 CON: 8±5.9 | BGT_ECA: 2.6±0.7 CON: 2.7±0.7 | Levodopa Equivalency Dosage BGT_ECA: 500±296.3 CON: 350±259.3 | ON | 3 | 3 | 30 | Single stance, time | 10m walking test, gait speed(m\s) | TUG, time | NA | NA |
| San Martín Valenzuela et al. (2020) | BGT: 64.8±8.8 DT_BGT: 66.4±7.1 | BGT: 17(12) DT_BGT: 23(11) | BGT: 5.3±3.8 DT_BGT: 6.3±6.0 | BGT: 2.5±0.7 DT_BGT: 2.7±0.6 | stable medication usage | ON | 10 | 2 | 60 | NA | 10m walking test, velocity(m/s) | NA | NA | NA |
| Helgerud et al. (2020) | PT: 72±8.0 CPP: 62.0±11 | PT: 15(7) CPP: 7(2) | PT: 8.8±4.9 CPP: 7.3±2.5 | PT: 2.3±0.1 CPP: 2.7±0.7 | Levodopa Equivalency Dosage PT: 693.0±473.0 CPP: 692.0±322.0 | ON | 4 | 3 | 60 | NA | NA | TUG, time | NA | NA |
| Bekkers et al. (2020) | TT: 70.9±6.0 VR: 71.1±6.3 | TT: 59(37) VR: 62(37) | TT: 9.6±7.2 VR: 9.1±5.5 | TT: 2.5±0.5 VR: 2.4±0.5 | stable medication usage | ON | 6 | 3 | 45 | NA | NA | NA | NA | mini-BESTest, score |
| Terrens et al. (2020) | AQE: 74.1±6.6 AQE: 65.6±7.7 CPP: 76.4±7.4 | AQE: 11(10) AQE: 10(7)  CPP: 9(7) | AQE: 6.7±6.3 AQE: 5.2±7.1  CPP: 4.2±3.1 | AQE: 3±1.5 AQE: 2±1.5  CPP: 3±0.7 | stable medication usage | ON | 12 | NA | 60 | NA | NA | NA | NA | mini-BESTest, score |
| Yi-zhao et al. (2017) | CPP: 64.5±6.8 AQE: 63.4±7.2 | CPP: 20(12) AQE: 20(14) | CPP: 4.3±1.7 AQE: 3.8±1.4 | CPP: 2.0±0.6 AQE: 1.9±0.5 | stable medication usage | ON | 8 | 5 | 50 | NA | 10m walking test, velocity(m/s) | TUGT, time | NA | BBS, score |
| Vitório et al. (2011) | CPP: 67.5±8.3 CON: 71.3±8.1 | CPP: 19(NA) CON: 10(NA) | CPP: 3.8±3.9 CON: 4.4±2.8 | CPP: 1.5±0.8 CON: 1.6±0.9 | stable medication usage | ON | 24 | 3 | 60 | NA | 8m walking test, velocity(m/s) | NA | NA | NA |
| Clerici et al. (2019) | Mul_D: 67.0±8.0 AQE: 67.0±11.0 | Mul_D: 27(19) AQE: 25(20) | NA | Mul_D: 2.7±0.4 AQE: 2.7±0.7 | Levodopa equivalent daily dose Mul_D: 919±407 AQE: 951±328 | ON | 4 | 6 | 60 | NA | NA | TUG, time | NA | BBS, score |
| El-Tamawy et al. (2012) | CPP: 61.4±7.3 Mul_D: 63.2±5.6 | CPP: 15(NA) Mul_D: 15(NA) | CPP: 4.0±0.9 Mul_D: 3.8±0.9 | NA | stable medication usage | ON | 8 | 3 | 60 | NA | walking test, Walking speed (m/s) | NA | NA | NA |
| Franzoni et al. (2018) | NW: 64.4±8.7 AE: 71.1±6.2 | NW: 14(NA) AE: 11(NA) | NA | NW: 1.6±0.6 AE: 2.0±1.1 | stable medication usage | ON | 9 | 4 | NA | COP, anteroposterior root mean square | NA | NA | NA | BBS, score |
| Medijainen et al. (2019) | CPP: 77.1±4.2 CON: 69.9±5.1 | CPP: 12(5) CON: 12(5) | CPP: 8.0±6.9 CON: 7.7±5.4 | CPP: 2.2±0.5 CON: 2.3±0.7 | stable medication usage | ON | 8 | 2 | 60 | NA | 3-meter gait test, velocity(m/s) | NA | NA | NA |
| Miyai et al. (2002) | BWS_TT: 69.5±6.3  BGT: 69.8±4.5 | BWS_TT: 11(5) BGT: 9(5) | BWS_TT: 4.1±2.7 BGT: 4.5±2.1 | BWS_TT: 2.9±0.3 BGT: 2.8±0.3 | Levodopa equivalent daily dose BWS_TT: 241.0±97.8  BGT: 255.6±68.4 | ON | 4 | 3 | 45 | NA | 10m walking test, time(s) | NA | NA | NA |
| Oliveira et al. (2020) | AQE: 65.5±2.2 Mul_D: 68.3±0.4 | AQE: 9(8) Mul_D: 10(9) | NA | AQE: 1.7±0.8 Mul_D: 2.0±1.0 | stable medication usage | ON | 4 | 2 | 60 | NA | NA | TUG, time | NA | BBS, score |
| Paolucci et al. (2017) | Mul_C: 67.0±8.1 Mul_D: 66.0±13.7 | Mul_C: 17(9) Mul_C: 17(10) | 3.0±1.2 | 1.5±0.8 | stable medication usage | ON | 5 | 2 | 60 | NA | NA | NA | NA | BBS, score |
| Pohl et al. (2013) | 68.2±5.1 | Dance: 12(NA) CON: 6(NA) | 8.8±3.8 | 2.4±0.7 | stable medication usage | ON | 6 | 2 | 60 | NA | NA | TUG, time | NA | NA |
| Morrone et al. (2016) | Mul_D: 75.0±2.6 CPP: 70.0±3.9 | Mul_D: 10(6) CPP: 10(6) | Mul_D: 6.3±3.3 CPP: 6.5±3.5 | Mul_D: 3.0±0 CPP: 3.0±0.7 | stable medication usage | ON | 10 | 3 | 45 | Anterior Posterior Stability Index | NA | NA | NA | NA |
| Shahmohammadi et al. (2017) | AQE: 60.5±5.4 AE: 53.2±4.9 | AQE: 10(10) AE: 10(10) | NA | NA | stable medication usage | ON | 8 | 3 | 55 | COP, Sway range | NA | NA | NA | NA |
| Vergara-Diaz et al. (2018) | TC: 65.7±3.9 CON: 62.0±7.8 | TC: 16(9) CON: 16(7) | TC: 2.9±2.4 CON: 2.9±2.2 | TC: 2.2±0.2 CON: 2.1±0.2 | stable medication usage | OFF | 24 | 2 | 60 | NA | 15m walking test, velocity(m/s) | TUG, time | NA | NA |
| Vieira de Moraes Filho et al. (2020) | RT: 64.7±9.0 CON: 64.4±5.8 | RT: 25(20) CON: 15(10) | RT: 5.7±4.0 CON: 7.2±7.4 | NA | stable medication usage | ON | 9 | 2 | 55 | NA | 10m walking test, velocity(m/s) | TUG, time | NA | NA |
| Yotnuengnit et al. (2018) | Mul_D: 68.2±9.8 CPP: 62.7±8.8 | Mul_D: 17(11) CPP: 718(12) | Mul_D: 9.4±5.3 CPP: 6.6±3.6 | Mul_D: 2.5±0.5 CPP: 2.4±0.5 | Levodopa equivalent daily dose Mul_D: 829.0±360.6 CPP: 912.0±472.9 | ON | 2 | 3 | 30 | NA | walking test, Gait speed(m/s) | NA | NA | NA |
| Youm et al. (2020) | RT: 68.0±6.8 Stretch: 72.1±6.0 | RT: 10(6) Stretch: 7(4) | RT: 6.4±3.6 Stretch: 8.0±4.0 | RT: 2.4±0.3 Stretch: 2.3±0.4 | Levodopa equivalent daily dose RT: 567.0±274.6 Stretch: 852.9±564.4 | ON | 12 | 3 | 75 | NA | Sit-to-Walk Test, Step speed (m/s) | TUG, time | NA | NA |
| NCT01156714 | TT: 63.2±11.28 Mul_D: 64.55±7.07 | TT: 20(14) Mul_D: 20(16) | NA | TT: 2.34±0.41 Mul_D: 2.35±0.54 | stable medication usage | ON | 12 | NA | NA | NA | NA | NA | NA | NA |
| NCT01768832 | TT: 68.52±9.54 Tango: 66.73±9.52 Stretch: 66.18±7.3 | TT: 31(17) Tango: 39(25) Stretch: 26(14) | TT: 5.59±3.81 Tango: 6.10±4.82 Stretch: 4.4±5.04 | NA | stable medication usage | ON | 12 | 2 | 60 | NA | walking test, velocity(cm/s) | NA | NA | Mini-BESTest, score |

*NA* not available, *TUG* Time up and go, *BBS* Berg balance scale, *Mini-BESTest* Mini balance evaluation systems test, *FR* Functional reach, *COP* center of press, *AE* Aerobic Exercise, *AQE* Aquatic Exercise, *BGT* Balance and Gait Training, *BGT_ECA* Balance and Gait Training with External Cue or Attention, *BGT_ICA* Balance and Gait Training with Internal Cue or Attention, *BWS_TT* Body Weight Support Treadmill Training, *CON* Control group, *CPP* Classic Physiotherapy Program, *DT_BGT* Dual Task Balance and Gait Training, *Mul_C* Multicomponent Exercise Program, *Mul_D* Multidisciplinary Exercise Program, *NW* Nordic Walking, *PT* Power Training, *RA_GT* Robotic Assisted Gait Training, *RT* Resistance Training, *TC* Tai Chi, *TT* Treadmill Training, *VR* Virtual Reality, *WBV* Whole Body Vibration.

## List of included studies

Abraham, A., Hart, A., Andrade, I., & Hackney, M. E. (2018). Dynamic Neuro-Cognitive Imagery Improves Mental Imagery Ability, Disease Severity, and Motor and Cognitive Functions in People with Parkinson's Disease. *Neural plasticity, 2018*, 6168507. doi:10.1155/2018/6168507

Allen, N. E., Canning, C. G., Sherrington, C., Lord, S. R., Latt, M. D., Close, J. C. T., . . . Fung, V. S. C. (2010). The effects of an exercise program on fall risk factors in people with Parkinson's disease: a randomized controlled trial. *Movement disorders : official journal of the Movement Disorder Society, 25*(9), 1217-1225. doi:10.1002/mds.23082

Amano, S., Nocera, J. R., Vallabhajosula, S., Juncos, J. L., Gregor, R. J., Waddell, D. E., . . . Hass, C. J. (2013). The effect of Tai Chi exercise on gait initiation and gait performance in persons with Parkinson's disease. *Parkinsonism & related disorders, 19*(11), 955-960. doi:10.1016/j.parkreldis.2013.06.007

Arcolin, I., Pisano, F., Delconte, C., Godi, M., Schieppati, M., Mezzani, A., . . . Nardone, A. (2016). Intensive cycle ergometer training improves gait speed and endurance in patients with Parkinson's disease: A comparison with treadmill training. *Restorative neurology and neuroscience, 34*(1), 125-138. doi:10.3233/RNN-150506

Arfa-Fatollahkhani, P., Safar Cherati, A., Habibi, S. A. H., Shahidi, G. A., Sohrabi, A., & Zamani, B. (2019). Effects of treadmill training on the balance, functional capacity and quality of life in Parkinson's disease: A randomized clinical trial. *Journal of complementary & integrative medicine, 17*(1). doi:10.1515/jcim-2018-0245

Ashburn, A., Fazakarley, L., Ballinger, C., Pickering, R., McLellan, L. D., & Fitton, C. (2007). A randomised controlled trial of a home based exercise programme to reduce the risk of falling among people with Parkinson's disease. *Journal of neurology, neurosurgery, and psychiatry, 78*(7), 678-684. Retrieved from https://pubmed.ncbi.nlm.nih.gov/17119004

Atan, T., Özyemişci Taşkıran, Ö., Bora Tokçaer, A., Kaymak Karataş, G., Karakuş Çalışkan, A., & Karaoğlan, B. (2019). Effects of different percentages of body weight-supported treadmill training in Parkinson’s disease: a double-blind randomized controlled trial. *Turkish journal of medical sciences, 49*(4). doi:10.3906/sag-1812-57

Bakhshayesh, B., Sayyar, S., & Daneshmandi, H. (2017). Pilates Exercise and Functional Balance in Parkinson's Disease. *Caspian Journal of Neurological Sciences, 3*(1), 25-38.

Bang, D.-H., & Shin, W.-S. (2017). Effects of an intensive Nordic walking intervention on the balance function and walking ability of individuals with Parkinson's disease: a randomized controlled pilot trial. *Aging clinical and experimental research, 29*(5), 993-999. doi:10.1007/s40520-016-0648-9

Beck, E. N., Intzandt, B. N., & Almeida, Q. J. (2018). Can Dual Task Walking Improve in Parkinson's Disease After External Focus of Attention Exercise? A Single Blind Randomized Controlled Trial. *Neurorehabilitation and neural repair, 32*(1), 18-33. doi:10.1177/1545968317746782

Bekkers, E. M. J., Mirelman, A., Alcock, L., Rochester, L., Nieuwhof, F., Bloem, B. R., . . . Nieuwboer, A. (2020). Do Patients With Parkinson's Disease With Freezing of Gait Respond Differently Than Those Without to Treadmill Training Augmented by Virtual Reality? *Neurorehabilitation and neural repair, 34*(5), 440-449. doi:10.1177/1545968320912756

Bello, O., Sanchez, J. A., Lopez-Alonso, V., Márquez, G., Morenilla, L., Castro, X., . . . Fernandez-del-Olmo, M. (2013). The effects of treadmill or overground walking training program on gait in Parkinson's disease. *Gait & posture, 38*(4), 590-595. doi:10.1016/j.gaitpost.2013.02.005

Cabrera-Martos, I., Jiménez-Martín, A. T., López-López, L., Rodríguez-Torres, J., Ortiz-Rubio, A., & Valenza, M. C. (2020). Effects of a core stabilization training program on balance ability in persons with Parkinson's disease: a randomized controlled trial. *Clinical rehabilitation, 34*(6), 764-772. doi:10.1177/0269215520918631

Cakit, B. D., Saracoglu, M., Genc, H., Erdem, H. R., & Inan, L. (2007). The effects of incremental speed-dependent treadmill training on postural instability and fear of falling in Parkinson's disease. *Clinical rehabilitation, 21*(8), 698-705. Retrieved from https://pubmed.ncbi.nlm.nih.gov/17846069

Calabrò, R. S., Naro, A., Filoni, S., Pullia, M., Billeri, L., Tomasello, P., . . . Bramanti, P. (2019). Walking to your right music: a randomized controlled trial on the novel use of treadmill plus music in Parkinson's disease. *Journal of neuroengineering and rehabilitation, 16*(1), 68. doi:10.1186/s12984-019-0533-9

Cancela, J. M., Mollinedo, I., Montalvo, S., & Vila Suárez, M. E. (2020). Effects of a High-Intensity Progressive-Cycle Program on Quality of Life and Motor Symptomatology in a Parkinson's Disease Population: A Pilot Randomized Controlled Trial. *Rejuvenation research, 23*(6), 508-515. doi:10.1089/rej.2019.2267

Canning, C. G., Allen, N. E., Dean, C. M., Goh, L., & Fung, V. S. C. (2012). Home-based treadmill training for individuals with Parkinson's disease: a randomized controlled pilot trial. *Clinical rehabilitation, 26*(9), 817-826. doi:10.1177/0269215511432652

Capato, T. T. C., de Vries, N. M., IntHout, J., Barbosa, E. R., Nonnekes, J., & Bloem, B. R. (2020). Multimodal Balance Training Supported by Rhythmical Auditory Stimuli in Parkinson's Disease: A Randomized Clinical Trial. *Journal of Parkinson's disease, 10*(1), 333-346. doi:10.3233/JPD-191752

Capato, T. T. C., Nonnekes, J., de Vries, N. M., IntHout, J., Barbosa, E. R., & Bloem, B. R. (2020). Effects of multimodal balance training supported by rhythmical auditory stimuli in people with advanced stages of Parkinson's disease: a pilot randomized clinical trial. *Journal of the neurological sciences, 418*, 117086. doi:10.1016/j.jns.2020.117086

Capecci, M., Pournajaf, S., Galafate, D., Sale, P., Le Pera, D., Goffredo, M., . . . Franceschini, M. (2019). Clinical effects of robot-assisted gait training and treadmill training for Parkinson's disease. A randomized controlled trial. *Annals of physical and rehabilitation medicine, 62*(5), 303-312. doi:10.1016/j.rehab.2019.06.016

Capecci, M., Serpicelli, C., Fiorentini, L., Censi, G., Ferretti, M., Orni, C., . . . Ceravolo, M. G. (2014). Postural rehabilitation and Kinesio taping for axial postural disorders in Parkinson's disease. *Archives of physical medicine and rehabilitation, 95*(6), 1067-1075. doi:10.1016/j.apmr.2014.01.020

Carda, S., Invernizzi, M., Baricich, A., Comi, C., Croquelois, A., & Cisari, C. (2012). Robotic gait training is not superior to conventional treadmill training in parkinson disease: a single-blind randomized controlled trial. *Neurorehabilitation and neural repair, 26*(9), 1027-1034.

Carpinella, I., Cattaneo, D., Bonora, G., Bowman, T., Martina, L., Montesano, A., & Ferrarin, M. (2017). Wearable Sensor-Based Biofeedback Training for Balance and Gait in Parkinson Disease: A Pilot Randomized Controlled Trial. *Archives of physical medicine and rehabilitation, 98*(4). doi:10.1016/j.apmr.2016.11.003

Carvalho, A., Barbirato, D., Araujo, N., Martins, J. V., Cavalcanti, J. L. S., Santos, T. M., . . . Deslandes, A. C. (2015). Comparison of strength training, aerobic training, and additional physical therapy as supplementary treatments for Parkinson's disease: pilot study. *Clinical interventions in aging, 10*, 183-191. doi:10.2147/CIA.S68779

Cheng, F.-Y., Yang, Y.-R., Chen, L.-M., Wu, Y.-R., Cheng, S.-J., & Wang, R.-Y. (2016). Positive Effects of Specific Exercise and Novel Turning-based Treadmill Training on Turning Performance in Individuals with Parkinson's disease: A Randomized Controlled Trial. *Scientific reports, 6*, 33242. doi:10.1038/srep33242

Cheng, F.-Y., Yang, Y.-R., Wu, Y.-R., Cheng, S.-J., & Wang, R.-Y. (2017). Effects of curved-walking training on curved-walking performance and freezing of gait in individuals with Parkinson's disease: A randomized controlled trial. *Parkinsonism & related disorders, 43*, 20-26. doi:10.1016/j.parkreldis.2017.06.021

Cherup, N. P., Strand, K. L., Lucchi, L., Wooten, S. V., Luca, C., & Signorile, J. F. (2021). Yoga Meditation Enhances Proprioception and Balance in Individuals Diagnosed With Parkinson's Disease. *Perceptual and motor skills, 128*(1), 304-323. doi:10.1177/0031512520945085

Chivers Seymour, K., Pickering, R., Rochester, L., Roberts, H. C., Ballinger, C., Hulbert, S., . . . Ashburn, A. (2019). Multicentre, randomised controlled trial of PDSAFE, a physiotherapist-delivered fall prevention programme for people with Parkinson's. *Journal of neurology, neurosurgery, and psychiatry, 90*(7), 774-782. doi:10.1136/jnnp-2018-319448

Choi, H.-J. (2016). Effects of therapeutic Tai chi on functional fitness and activities of daily living in patients with Parkinson disease. *Journal of exercise rehabilitation, 12*(5), 499-503. Retrieved from https://pubmed.ncbi.nlm.nih.gov/27807532

Clerici, I., Maestri, R., Bonetti, F., Ortelli, P., Volpe, D., Ferrazzoli, D., & Frazzitta, G. (2019). Land Plus Aquatic Therapy Versus Land-Based Rehabilitation Alone for the Treatment of Freezing of Gait in Parkinson Disease: A Randomized Controlled Trial. *Physical therapy, 99*(5), 591-600. doi:10.1093/ptj/pzz003

Combs, S. A., Diehl, M. D., Chrzastowski, C., Didrick, N., McCoin, B., Mox, N., . . . Wayman, J. (2013). Community-based group exercise for persons with Parkinson disease: a randomized controlled trial. *NeuroRehabilitation, 32*(1), 117-124. doi:10.3233/NRE-130828

Conradsson, D., Löfgren, N., Nero, H., Hagströmer, M., Ståhle, A., Lökk, J., & Franzén, E. (2015). The Effects of Highly Challenging Balance Training in Elderly With Parkinson's Disease: A Randomized Controlled Trial. *Neurorehabilitation and neural repair, 29*(9), 827-836. doi:10.1177/1545968314567150

Costa-Ribeiro, A., Maux, A., Bosford, T., Aoki, Y., Castro, R., Baltar, A., . . . Monte-Silva, K. (2017). Transcranial direct current stimulation associated with gait training in Parkinson's disease: A pilot randomized clinical trial. *Developmental neurorehabilitation, 20*(3), 121-128. doi:10.3109/17518423.2015.1131755

Cugusi, L., Solla, P., Serpe, R., Carzedda, T., Piras, L., Oggianu, M., . . . Mercuro, G. (2015). Effects of a Nordic Walking program on motor and non-motor symptoms, functional performance and body composition in patients with Parkinson's disease. *NeuroRehabilitation, 37*(2), 245-254. doi:10.3233/NRE-151257

Daneshmandi, H., Sayyar, S., & Bakhshayesh, B. (2017). The effect of a selective Pilates program on functional balance and falling risk in patients with Parkinson’s disease. *Zahedan Journal of Research in Medical Sciences, 19*(4).

de Bruin, N., Doan, J. B., Turnbull, G., Suchowersky, O., Bonfield, S., Hu, B., & Brown, L. A. (2010). Walking with music is a safe and viable tool for gait training in Parkinson's disease: the effect of a 13-week feasibility study on single and dual task walking. *Parkinson's disease, 2010*, 483530. doi:10.4061/2010/483530

de Lima, T. A., Ferreira-Moraes, R., Alves, W. M. G. d. C., Alves, T. G. G., Pimentel, C. P., Sousa, E. C., . . . Cortinhas-Alves, E. A. (2019). Resistance training reduces depressive symptoms in elderly people with Parkinson disease: A controlled randomized study. *Scandinavian journal of medicine & science in sports, 29*(12), 1957-1967. doi:10.1111/sms.13528

de Melo, G. E. L., Kleiner, A. F. R., Lopes, J. B. P., Dumont, A. J. L., Lazzari, R. D., Galli, M., & Oliveira, C. S. (2018). Effect of virtual reality training on walking distance and physical fitness in individuals with Parkinson's disease. *NeuroRehabilitation, 42*(4), 473-480. doi:10.3233/NRE-172355

Demonceau, M., Maquet, D., Jidovtseff, B., Donneau, A. F., Bury, T., Croisier, J. L., . . . Garraux, G. (2017). Effects of twelve weeks of aerobic or strength training in addition to standard care in Parkinson's disease: a controlled study. *European journal of physical and rehabilitation medicine, 53*(2), 184-200. doi:10.23736/S1973-9087.16.04272-6

Droby, A., Maidan, I., Jacob, Y., Giladi, N., Hausdorff, J. M., & Mirelman, A. (2020). Distinct Effects of Motor Training on Resting-State Functional Networks of the Brain in Parkinson's Disease. *Neurorehabilitation and neural repair, 34*(9), 795-803. doi:10.1177/1545968320940985

Ebersbach, G., Ebersbach, A., Edler, D., Kaufhold, O., Kusch, M., Kupsch, A., & Wissel, J. (2010). Comparing exercise in Parkinson's disease--the Berlin LSVT®BIG study. *Movement disorders : official journal of the Movement Disorder Society, 25*(12), 1902-1908. doi:10.1002/mds.23212

Ebersbach, G., Edler, D., Kaufhold, O., & Wissel, J. (2008). Whole body vibration versus conventional physiotherapy to improve balance and gait in Parkinson's disease. *Archives of physical medicine and rehabilitation, 89*(3), 399-403. doi:10.1016/j.apmr.2007.09.031

El-Tamawy, M. S., Darwish, M. H., & Khallaf, M. E. (2012). Effects of augmented proprioceptive cues on the parameters of gait of individuals with Parkinson's disease. *Annals of Indian Academy of Neurology, 15*(4), 267-272. doi:10.4103/0972-2327.104334

Ellis, T., de Goede, C. J., Feldman, R. G., Wolters, E. C., Kwakkel, G., & Wagenaar, R. C. (2005). Efficacy of a physical therapy program in patients with Parkinson's disease: a randomized controlled trial. *Archives of physical medicine and rehabilitation, 86*(4), 626-632. Retrieved from https://pubmed.ncbi.nlm.nih.gov/15827910

Feng, H., Li, C., Liu, J., Wang, L., Ma, J., Li, G., . . . Wu, Z. (2019). Virtual Reality Rehabilitation Versus Conventional Physical Therapy for Improving Balance and Gait in Parkinson's Disease Patients: A Randomized Controlled Trial. *Medical science monitor : international medical journal of experimental and clinical research, 25*, 4186-4192. doi:10.12659/MSM.916455

Fernandes, Â., Rocha, N., Santos, R., & Tavares, J. M. R. S. (2015). Effects of dual-task training on balance and executive functions in Parkinson's disease: A pilot study. *Somatosensory & motor research, 32*(2), 122-127. doi:10.3109/08990220.2014.1002605

Ferraz, D. D., Trippo, K. V., Duarte, G. P., Neto, M. G., Bernardes Santos, K. O., & Filho, J. O. (2018). The Effects of Functional Training, Bicycle Exercise, and Exergaming on Walking Capacity of Elderly Patients With Parkinson Disease: A Pilot Randomized Controlled Single-blinded Trial. *Archives of physical medicine and rehabilitation, 99*(5), 826-833. doi:10.1016/j.apmr.2017.12.014

Fil-Balkan, A., Salci, Y., Keklicek, H., Armutlu, K., Aksoy, S., Kayihan, H., & Elibol, B. (2018). Sensorimotor integration training in Parkinson`s disease. *Neurosciences (Riyadh, Saudi Arabia), 23*(4), 208-215. doi:10.17712/nsj.2018.3.20180021

Fisher, B. E., Wu, A. D., Salem, G. J., Song, J., Lin, C.-H. J., Yip, J., . . . Petzinger, G. (2008). The effect of exercise training in improving motor performance and corticomotor excitability in people with early Parkinson's disease. *Archives of physical medicine and rehabilitation, 89*(7), 1221-1229. doi:10.1016/j.apmr.2008.01.013

Franzoni, L. T., Monteiro, E. P., Oliveira, H. B., da Rosa, R. G., Costa, R. R., Rieder, C., . . . Peyré-Tartaruga, L. A. (2018). A 9-Week Nordic and Free Walking Improve Postural Balance in Parkinson's Disease. *Sports medicine international open, 2*(2), E28-E34. doi:10.1055/s-0043-124757

Frazzitta, G., Bossio, F., Maestri, R., Palamara, G., Bera, R., & Ferrazzoli, D. (2015). Crossover versus Stabilometric Platform for the Treatment of Balance Dysfunction in Parkinson's Disease: A Randomized Study. *BioMed research international, 2015*, 878472. doi:10.1155/2015/878472

Frazzitta, G., Maestri, R., Uccellini, D., Bertotti, G., & Abelli, P. (2009). Rehabilitation treatment of gait in patients with Parkinson's disease with freezing: a comparison between two physical therapy protocols using visual and auditory cues with or without treadmill training. *Movement disorders : official journal of the Movement Disorder Society, 24*(8), 1139-1143. doi:10.1002/mds.22491

Furnari, A., Calabrò, R. S., De Cola, M. C., Bartolo, M., Castelli, A., Mapelli, A., . . . Casale, R. (2017). Robotic-assisted gait training in Parkinson's disease: a three-month follow-up randomized clinical trial. *The International journal of neuroscience, 127*(11). doi:10.1080/00207454.2017.1288623

Galli, M., Cimolin, V., De Pandis, M. F., Le Pera, D., Sova, I., Albertini, G., . . . Franceschini, M. (2016). Robot-assisted gait training versus treadmill training in patients with Parkinson's disease: a kinematic evaluation with gait profile score. *Functional neurology, 31*(3), 163-170. Retrieved from https://pubmed.ncbi.nlm.nih.gov/27678210

Gandolfi, M., Geroin, C., Dimitrova, E., Boldrini, P., Waldner, A., Bonadiman, S., . . . Smania, N. (2017). Virtual Reality Telerehabilitation for Postural Instability in Parkinson's Disease: A Multicenter, Single-Blind, Randomized, Controlled Trial. *BioMed research international, 2017*, 7962826. doi:10.1155/2017/7962826

Gandolfi, M., Tinazzi, M., Magrinelli, F., Busselli, G., Dimitrova, E., Polo, N., . . . Geroin, C. (2019). Four-week trunk-specific exercise program decreases forward trunk flexion in Parkinson's disease: A single-blinded, randomized controlled trial. *Parkinsonism & related disorders, 64*, 268-274. doi:10.1016/j.parkreldis.2019.05.006

Ganesan, M., Sathyaprabha, T. N., Pal, P. K., & Gupta, A. (2015). Partial Body Weight-Supported Treadmill Training in Patients With Parkinson Disease: Impact on Gait and Clinical Manifestation. *Archives of physical medicine and rehabilitation, 96*(9), 1557-1565. doi:10.1016/j.apmr.2015.05.007

Gao, Q., Leung, A., Yang, Y., Wei, Q., Guan, M., Jia, C., & He, C. (2014). Effects of Tai Chi on balance and fall prevention in Parkinson's disease: a randomized controlled trial. *Clinical rehabilitation, 28*(8), 748-753. Retrieved from https://pubmed.ncbi.nlm.nih.gov/24519923

Ghielen, I., van Wegen, E. E. H., Rutten, S., de Goede, C. J. T., Houniet-de Gier, M., Collette, E. H., . . . van den Heuvel, O. A. (2017). Body awareness training in the treatment of wearing-off related anxiety in patients with Parkinson's disease: Results from a pilot randomized controlled trial. *Journal of psychosomatic research, 103*, 1-8. doi:10.1016/j.jpsychores.2017.09.008

Ginis, P., Nieuwboer, A., Dorfman, M., Ferrari, A., Gazit, E., Canning, C. G., . . . Mirelman, A. (2016). Feasibility and effects of home-based smartphone-delivered automated feedback training for gait in people with Parkinson's disease: A pilot randomized controlled trial. *Parkinsonism & related disorders, 22*, 28-34. Retrieved from https://pubmed.ncbi.nlm.nih.gov/26777408

Goodwin, V. A., Richards, S. H., Henley, W., Ewings, P., Taylor, A. H., & Campbell, J. L. (2011). An exercise intervention to prevent falls in people with Parkinson's disease: a pragmatic randomised controlled trial. *Journal of neurology, neurosurgery, and psychiatry, 82*(11), 1232-1238. doi:10.1136/jnnp-2011-300919

Granziera, S., Alessandri, A., Lazzaro, A., Zara, D., & Scarpa, A. (2021). Nordic Walking and Walking in Parkinson's disease: a randomized single-blind controlled trial. *Aging clinical and experimental research, 33*(4), 965-971. doi:10.1007/s40520-020-01617-w

Grobbelaar, R., Venter, R., & Welman, K. E. (2017). Backward compared to forward over ground gait retraining have additional benefits for gait in individuals with mild to moderate Parkinson's disease: A randomized controlled trial. *Gait & posture, 58*, 294-299. doi:10.1016/j.gaitpost.2017.08.019

Hackney, M. E., & Earhart, G. M. (2008). Tai Chi improves balance and mobility in people with Parkinson disease. *Gait & posture, 28*(3), 456-460. doi:10.1016/j.gaitpost.2008.02.005

Hackney, M. E., & Earhart, G. M. (2009). Effects of dance on movement control in Parkinson's disease: a comparison of Argentine tango and American ballroom. *Journal of rehabilitation medicine, 41*(6), 475-481. doi:10.2340/16501977-0362

Hackney, M. E., Kantorovich, S., Levin, R., & Earhart, G. M. (2007). Effects of tango on functional mobility in Parkinson's disease: a preliminary study. *Journal of neurologic physical therapy : JNPT, 31*(4), 173-179. doi:10.1097/NPT.0b013e31815ce78b

Harro, C. C., Shoemaker, M. J., Frey, O., Gamble, A. C., Harring, K. B., Karl, K. L., . . . VanHaitsma, R. J. (2014). The effects of speed-dependent treadmill training and rhythmic auditory-cued overground walking on balance function, fall incidence, and quality of life in individuals with idiopathic Parkinson's disease: a randomized controlled trial. *NeuroRehabilitation, 34*(3), 541-556. doi:10.3233/NRE-141048

Hashimoto, H., Takabatake, S., Miyaguchi, H., Nakanishi, H., & Naitou, Y. (2015). Effects of dance on motor functions, cognitive functions, and mental symptoms of Parkinson's disease: a quasi-randomized pilot trial. *Complementary therapies in medicine, 23*(2), 210-219. doi:10.1016/j.ctim.2015.01.010

Hass, C. J., Buckley, T. A., Pitsikoulis, C., & Barthelemy, E. J. (2012). Progressive resistance training improves gait initiation in individuals with Parkinson's disease. *Gait & posture, 35*(4), 669-673. doi:10.1016/j.gaitpost.2011.12.022

Helgerud, J., Thomsen, S. N., Hoff, J., Strandbråten, A., Leivseth, G., Unhjem, R., & Wang, E. (2020). Maximal strength training in patients with Parkinson's disease: impact on efferent neural drive, force-generating capacity, and functional performance. *Journal of applied physiology (Bethesda, Md. : 1985), 129*(4), 683-690. doi:10.1152/japplphysiol.00208.2020

Hirsch, M. A., Toole, T., Maitland, C. G., & Rider, R. A. (2003). The effects of balance training and high-intensity resistance training on persons with idiopathic Parkinson's disease. *Archives of physical medicine and rehabilitation, 84*(8), 1109-1117. Retrieved from https://pubmed.ncbi.nlm.nih.gov/12917847

Hubble, R. P., Naughton, G., Silburn, P. A., & Cole, M. H. (2018). Trunk Exercises Improve Gait Symmetry in Parkinson Disease: A Blind Phase II Randomized Controlled Trial. *American journal of physical medicine & rehabilitation, 97*(3), 151-159. doi:10.1097/PHM.0000000000000858

Johansson, H., Freidle, M., Ekman, U., Schalling, E., Leavy, B., Svenningsson, P., . . . Franzén, E. (2020). Feasibility Aspects of Exploring Exercise-Induced Neuroplasticity in Parkinson's Disease: A Pilot Randomized Controlled Trial. *Parkinson's disease, 2020*, 2410863. doi:10.1155/2020/2410863

Joseph, C., Brodin, N., Leavy, B., Hagströmer, M., Löfgren, N., & Franzén, E. (2019). Cost-effectiveness of the HiBalance training program for elderly with Parkinson's disease: analysis of data from a randomized controlled trial. *Clinical rehabilitation, 33*(2), 222-232. doi:10.1177/0269215518800832

Jung, S. H., Hasegawa, N., Mancini, M., King, L. A., Carlson-Kuhta, P., Smulders, K., . . . Horak, F. B. (2020). Effects of the agility boot camp with cognitive challenge (ABC-C) exercise program for Parkinson's disease. *NPJ Parkinson's disease, 6*(1), 31. doi:10.1038/s41531-020-00132-z

Kadivar, Z., Corcos, D. M., Foto, J., & Hondzinski, J. M. (2011). Effect of step training and rhythmic auditory stimulation on functional performance in Parkinson patients. *Neurorehabilitation and neural repair, 25*(7), 626-635.

Khalil, H., Busse, M., Quinn, L., Nazzal, M., Batyha, W., Alkhazaleh, S., & Alomari, M. A. (2017). A pilot study of a minimally supervised home exercise and walking program for people with Parkinson's disease in Jordan. *Neurodegenerative disease management, 7*(1), 73-84. doi:10.2217/nmt-2016-0041

Khuzema, A., Brammatha, A., & Arul Selvan, V. (2020). Effect of home-based Tai Chi, Yoga or conventional balance exercise on functional balance and mobility among persons with idiopathic Parkinson's disease: An experimental study. *Hong Kong physiotherapy journal : official publication of the Hong Kong Physiotherapy Association Limited = Wu li chih liao, 40*(1), 39-49. doi:10.1142/S1013702520500055

King, L. A., Salarian, A., Mancini, M., Priest, K. C., Nutt, J., Serdar, A., . . . Horak, F. B. (2013). Exploring outcome measures for exercise intervention in people with Parkinson's disease. *Parkinson's disease, 2013*, 572134. doi:10.1155/2013/572134

Kunkel, D., Fitton, C., Roberts, L., Pickering, R. M., Roberts, H. C., Wiles, R., . . . Ashburn, A. (2017). A randomized controlled feasibility trial exploring partnered ballroom dancing for people with Parkinson's disease. *Clinical rehabilitation, 31*(10), 1340-1350. doi:10.1177/0269215517694930

Kurt, E. E., Büyükturan, B., Büyükturan, Ö., Erdem, H. R., & Tuncay, F. (2018). Effects of Ai Chi on balance, quality of life, functional mobility, and motor impairment in patients with Parkinson's disease<sup/>. *Disability and rehabilitation, 40*(7), 791-797. doi:10.1080/09638288.2016.1276972

Kurtais, Y., Kutlay, S., Tur, B. S., Gok, H., & Akbostanci, C. (2008). Does treadmill training improve lower-extremity tasks in Parkinson disease? A randomized controlled trial. *Clinical journal of sport medicine : official journal of the Canadian Academy of Sport Medicine, 18*(3), 289-291. doi:10.1097/JSM.0b013e318170626d

Kwok, J. Y. Y., Kwan, J. C. Y., Auyeung, M., Mok, V. C. T., Lau, C. K. Y., Choi, K. C., & Chan, H. Y. L. (2019). Effects of Mindfulness Yoga vs Stretching and Resistance Training Exercises on Anxiety and Depression for People With Parkinson Disease: A Randomized Clinical Trial. *JAMA neurology, 76*(7), 755-763. doi:10.1001/jamaneurol.2019.0534

Landers, M. R., Hatlevig, R. M., Davis, A. D., Richards, A. R., & Rosenlof, L. E. (2016). Does attentional focus during balance training in people with Parkinson's disease affect outcome? A randomised controlled clinical trial. *Clinical rehabilitation, 30*(1), 53-63. doi:10.1177/0269215515570377

Leal, L. C., Abrahin, O., Rodrigues, R. P., da Silva, M. C., Araújo, A. P., de Sousa, E. C., . . . Cortinhas-Alves, E. A. (2019). Low-volume resistance training improves the functional capacity of older individuals with Parkinson's disease. *Geriatrics & gerontology international, 19*(7), 635-640. doi:10.1111/ggi.13682

Lee, H.-J., Kim, S.-Y., Chae, Y., Kim, M.-Y., Yin, C., Jung, W.-S., . . . Lee, H. (2018). Turo (Qi Dance) Program for Parkinson's Disease Patients: Randomized, Assessor Blind, Waiting-List Control, Partial Crossover Study. *Explore (New York, N.Y.), 14*(3), 216-223. doi:10.1016/j.explore.2017.11.002

Li, F., Harmer, P., Fitzgerald, K., Eckstrom, E., Stock, R., Galver, J., . . . Batya, S. S. (2012). Tai chi and postural stability in patients with Parkinson's disease. *The New England journal of medicine, 366*(6), 511-519. doi:10.1056/NEJMoa1107911

Liao, Y.-Y., Yang, Y.-R., Cheng, S.-J., Wu, Y.-R., Fuh, J.-L., & Wang, R.-Y. (2015). Virtual Reality-Based Training to Improve Obstacle-Crossing Performance and Dynamic Balance in Patients With Parkinson's Disease. *Neurorehabilitation and neural repair, 29*(7), 658-667. doi:10.1177/1545968314562111

Liu, X. L., Chen, S., & Wang, Y. (2016). Effects of Health Qigong Exercises on Relieving Symptoms of Parkinson's Disease. *Evidence-based complementary and alternative medicine : eCAM, 2016*, 5935782. Retrieved from https://pubmed.ncbi.nlm.nih.gov/27891159

Löfgren, N., Conradsson, D., Rennie, L., Moe-Nilssen, R., & Franzén, E. (2019). The effects of integrated single- and dual-task training on automaticity and attention allocation in Parkinson's disease: A secondary analysis from a randomized trial. *Neuropsychology, 33*(2), 147-156. doi:10.1037/neu0000496

Maidan, I., Nieuwhof, F., Bernad-Elazari, H., Bloem, B. R., Giladi, N., Hausdorff, J. M., . . . Mirelman, A. (2018). Evidence for Differential Effects of 2 Forms of Exercise on Prefrontal Plasticity During Walking in Parkinson's Disease. *Neurorehabilitation and neural repair, 32*(3), 200-208. doi:10.1177/1545968318763750

McNeely, M. E., Mai, M. M., Duncan, R. P., & Earhart, G. M. (2015). Differential Effects of Tango Versus Dance for PD in Parkinson Disease. *Frontiers in aging neuroscience, 7*, 239. doi:10.3389/fnagi.2015.00239

Medijainen, K., Pääsuke, M., Lukmann, A., & Taba, P. (2019). Versatile guideline-based physiotherapy intervention in groups to improve gait speed in Parkinson's disease patients. *NeuroRehabilitation, 44*(4), 579-586. doi:10.3233/NRE-192723

Michels, K., Dubaz, O., Hornthal, E., & Bega, D. (2018). "Dance Therapy" as a psychotherapeutic movement intervention in Parkinson's disease. *Complementary therapies in medicine, 40*, 248-252. doi:10.1016/j.ctim.2018.07.005

Miyai, I., Fujimoto, Y., Yamamoto, H., Ueda, Y., Saito, T., Nozaki, S., & Kang, J. (2002). Long-term effect of body weight-supported treadmill training in Parkinson's disease: a randomized controlled trial. *Archives of physical medicine and rehabilitation, 83*(10), 1370-1373. Retrieved from https://pubmed.ncbi.nlm.nih.gov/12370870

Mollinedo-Cardalda, I., Cancela-Carral, J. M., & Vila-Suárez, M. H. (2018). Effect of a Mat Pilates Program with TheraBand on Dynamic Balance in Patients with Parkinson's Disease: Feasibility Study and Randomized Controlled Trial. *Rejuvenation research, 21*(5), 423-430. doi:10.1089/rej.2017.2007

Monticone, M., Ambrosini, E., Laurini, A., Rocca, B., & Foti, C. (2015). In-patient multidisciplinary rehabilitation for Parkinson's disease: A randomized controlled trial. *Movement disorders : official journal of the Movement Disorder Society, 30*(8), 1050-1058. doi:10.1002/mds.26256

Morris, M. E., Iansek, R., & Kirkwood, B. (2009). A randomized controlled trial of movement strategies compared with exercise for people with Parkinson's disease. *Movement disorders : official journal of the Movement Disorder Society, 24*(1), 64-71. doi:10.1002/mds.22295

Morris, M. E., Menz, H. B., McGinley, J. L., Watts, J. J., Huxham, F. E., Murphy, A. T., . . . Iansek, R. (2015). A Randomized Controlled Trial to Reduce Falls in People With Parkinson's Disease. *Neurorehabilitation and neural repair, 29*(8), 777-785. doi:10.1177/1545968314565511

Morrone, M., Miccinilli, S., Bravi, M., Paolucci, T., Melgari, J. M., Salomone, G., . . . Sterzi, S. (2016). Perceptive rehabilitation and trunk posture alignment in patients with Parkinson disease: a single blind randomized controlled trial. *European journal of physical and rehabilitation medicine, 52*(6), 799-809. Retrieved from https://pubmed.ncbi.nlm.nih.gov/27171537

Myers, P. S., Harrison, E. C., Rawson, K. S., Horin, A. P., Sutter, E. N., McNeely, M. E., & Earhart, G. M. (2020). Yoga Improves Balance and Low-Back Pain, but Not Anxiety, in People with Parkinson's Disease. *International journal of yoga therapy, 30*(1), 41-48. doi:10.17761/2020-D-18-00028

Ni, M., Signorile, J. F., Mooney, K., Balachandran, A., Potiaumpai, M., Luca, C., . . . Perry, A. C. (2016). Comparative Effect of Power Training and High-Speed Yoga on Motor Function in Older Patients With Parkinson Disease. *Archives of physical medicine and rehabilitation, 97*(3). doi:10.1016/j.apmr.2015.10.095

Nieuwboer, A., Kwakkel, G., Rochester, L., Jones, D., van Wegen, E., Willems, A. M., . . . Lim, I. (2007). Cueing training in the home improves gait-related mobility in Parkinson's disease: the RESCUE trial. *Journal of neurology, neurosurgery, and psychiatry, 78*(2), 134-140. Retrieved from https://pubmed.ncbi.nlm.nih.gov/17229744

Oliveira, G. S. D., Iraci, L., Pinheiro, G. S., Casal, M. Z., Haas, A. N., Pochmann, D., . . . Dani, C. (2020). Effect of exercise and grape juice on epigenetic modulation and functional outcomes in PD: A randomized clinical trial. *Physiology & behavior, 227*, 113135. doi:10.1016/j.physbeh.2020.113135

Ortiz-Rubio, A., Cabrera-Martos, I., Torres-Sánchez, I., Casilda-López, J., López-López, L., & Valenza, M. C. (2018). Effects of a resistance training program on balance and fatigue perception in patients with Parkinson's disease: A randomized controlled trial. *Medicina clinica, 150*(12), 460-464. doi:10.1016/j.medcli.2017.10.022

Palamara, G., Gotti, F., Maestri, R., Bera, R., Gargantini, R., Bossio, F., . . . Frazzitta, G. (2017). Land Plus Aquatic Therapy Versus Land-Based Rehabilitation Alone for the Treatment of Balance Dysfunction in Parkinson Disease: A Randomized Controlled Study With 6-Month Follow-Up. *Archives of physical medicine and rehabilitation, 98*(6), 1077-1085. doi:10.1016/j.apmr.2017.01.025

Paolucci, T., Zangrando, F., Piccinini, G., Deidda, L., Basile, R., Bruno, E., . . . Saraceni, V. M. (2017). Impact of Mézières Rehabilitative Method in Patients with Parkinson's Disease: A Randomized Controlled Trial. *Parkinson's disease, 2017*, 2762987. doi:10.1155/2017/2762987

Park, A., Zid, D., Russell, J., Malone, A., Rendon, A., Wehr, A., & Li, X. (2014). Effects of a formal exercise program on Parkinson's disease: a pilot study using a delayed start design. *Parkinsonism & related disorders, 20*(1), 106-111. doi:10.1016/j.parkreldis.2013.10.003

Paul, S. S., Canning, C. G., Song, J., Fung, V. S. C., & Sherrington, C. (2014). Leg muscle power is enhanced by training in people with Parkinson's disease: a randomized controlled trial. *Clinical rehabilitation, 28*(3), 275-288. doi:10.1177/0269215513507462

Pazzaglia, C., Imbimbo, I., Tranchita, E., Minganti, C., Ricciardi, D., Lo Monaco, R., . . . Padua, L. (2020). Comparison of virtual reality rehabilitation and conventional rehabilitation in Parkinson's disease: a randomised controlled trial. *Physiotherapy, 106*, 36-42. doi:10.1016/j.physio.2019.12.007

Pérez de la Cruz, S. (2017). Effectiveness of aquatic therapy for the control of pain and increased functionality in people with Parkinson's disease: a randomized clinical trial. *European journal of physical and rehabilitation medicine, 53*(6), 825-832. doi:10.23736/S1973-9087.17.04647-0

Pérez-de la Cruz, S. (2018). A bicentric controlled study on the effects of aquatic Ai Chi in Parkinson disease. *Complementary therapies in medicine, 36*, 147-153. doi:10.1016/j.ctim.2017.12.001

Picelli, A., Melotti, C., Origano, F., Neri, R., Verzè, E., Gandolfi, M., . . . Smania, N. (2015). Robot-assisted gait training is not superior to balance training for improving postural instability in patients with mild to moderate Parkinson's disease: a single-blind randomized controlled trial. *Clinical rehabilitation, 29*(4), 339-347. doi:10.1177/0269215514544041

Picelli, A., Melotti, C., Origano, F., Neri, R., Waldner, A., & Smania, N. (2013). Robot-assisted gait training versus equal intensity treadmill training in patients with mild to moderate Parkinson's disease: a randomized controlled trial. *Parkinsonism & related disorders, 19*(6), 605-610. doi:10.1016/j.parkreldis.2013.02.010

Picelli, A., Melotti, C., Origano, F., Waldner, A., Gimigliano, R., & Smania, N. (2012). Does robotic gait training improve balance in Parkinson's disease? A randomized controlled trial. *Parkinsonism & related disorders, 18*(8), 990-993. doi:10.1016/j.parkreldis.2012.05.010

Picelli, A., Varalta, V., Melotti, C., Zatezalo, V., Fonte, C., Amato, S., . . . Smania, N. (2016). Effects of treadmill training on cognitive and motor features of patients with mild to moderate Parkinson's disease: a pilot, single-blind, randomized controlled trial. *Functional neurology, 31*(1), 25-31. Retrieved from https://pubmed.ncbi.nlm.nih.gov/27027891

Pohl, P., Dizdar, N., & Hallert, E. (2013). The Ronnie Gardiner Rhythm and Music Method - a feasibility study in Parkinson's disease. *Disability and rehabilitation, 35*(26), 2197-2204. doi:10.3109/09638288.2013.774060

Pohl, P., Wressle, E., Lundin, F., Enthoven, P., & Dizdar, N. (2020). Group-based music intervention in Parkinson's disease - findings from a mixed-methods study. *Clinical rehabilitation, 34*(4), 533-544. doi:10.1177/0269215520907669

Poliakoff, E., Galpin, A. J., McDonald, K., Kellett, M., Dick, J. P. R., Hayes, S., & Wearden, A. J. (2013). The effect of gym training on multiple outcomes in Parkinson's disease: a pilot randomised waiting-list controlled trial. *NeuroRehabilitation, 32*(1), 125-134. doi:10.3233/NRE-130829

Pompeu, J. E., Mendes, F. A. D. S., Silva, K. G. d., Lobo, A. M., Oliveira, T. d. P., Zomignani, A. P., & Piemonte, M. E. P. (2012). Effect of Nintendo Wii™-based motor and cognitive training on activities of daily living in patients with Parkinson's disease: a randomised clinical trial. *Physiotherapy, 98*(3), 196-204. doi:10.1016/j.physio.2012.06.004

Prodoehl, J., Rafferty, M. R., David, F. J., Poon, C., Vaillancourt, D. E., Comella, C. L., . . . Robichaud, J. A. (2015). Two-year exercise program improves physical function in Parkinson's disease: the PRET-PD randomized clinical trial. *Neurorehabilitation and neural repair, 29*(2), 112-122. doi:10.1177/1545968314539732

Protas, E. J., Mitchell, K., Williams, A., Qureshy, H., Caroline, K., & Lai, E. C. (2005). Gait and step training to reduce falls in Parkinson's disease. *NeuroRehabilitation, 20*(3), 183-190. Retrieved from https://pubmed.ncbi.nlm.nih.gov/16340099

Qutubuddin, A. A., Cifu, D. X., Armistead-Jehle, P., Carne, W., McGuirk, T. E., & Baron, M. S. (2007). A comparison of computerized dynamic posturography therapy to standard balance physical therapy in individuals with Parkinson's disease: a pilot study. *NeuroRehabilitation, 22*(4), 261-265. Retrieved from https://pubmed.ncbi.nlm.nih.gov/17971615

Rafferty, M. R., Prodoehl, J., Robichaud, J. A., David, F. J., Poon, C., Goelz, L. C., . . . Corcos, D. M. (2017). Effects of 2 Years of Exercise on Gait Impairment in People With Parkinson Disease: The PRET-PD Randomized Trial. *Journal of neurologic physical therapy : JNPT, 41*(1), 21-30. Retrieved from https://pubmed.ncbi.nlm.nih.gov/27977518

Rennie, L., Opheim, A., Dietrichs, E., Löfgren, N., & Franzén, E. (2021). Highly challenging balance and gait training for individuals with Parkinson's disease improves pace, rhythm and variability domains of gait - A secondary analysis from a randomized controlled trial. *Clinical rehabilitation, 35*(2), 200-212. doi:10.1177/0269215520956503

Ribas, C. G., Alves da Silva, L., Corrêa, M. R., Teive, H. G., & Valderramas, S. (2017). Effectiveness of exergaming in improving functional balance, fatigue and quality of life in Parkinson's disease: A pilot randomized controlled trial. *Parkinsonism & related disorders, 38*, 13-18. doi:10.1016/j.parkreldis.2017.02.006

Rios Romenets, S., Anang, J., Fereshtehnejad, S.-M., Pelletier, A., & Postuma, R. (2015). Tango for treatment of motor and non-motor manifestations in Parkinson's disease: a randomized control study. *Complementary therapies in medicine, 23*(2), 175-184. doi:10.1016/j.ctim.2015.01.015

Rosenfeldt, A. B., Penko, A. L., Streicher, M. C., Zimmerman, N. M., Koop, M. M., & Alberts, J. L. (2019). Improvements in temporal and postural aspects of gait vary following single- and multi-modal training in individuals with Parkinson's disease. *Parkinsonism & related disorders, 64*, 280-285. doi:10.1016/j.parkreldis.2019.05.021

Sacheli, M. A., Neva, J. L., Lakhani, B., Murray, D. K., Vafai, N., Shahinfard, E., . . . Stoessl, A. J. (2019). Exercise increases caudate dopamine release and ventral striatal activation in Parkinson's disease. *Movement disorders : official journal of the Movement Disorder Society, 34*(12), 1891-1900. doi:10.1002/mds.27865

Sage, M. D., & Almeida, Q. J. (2009). Symptom and gait changes after sensory attention focused exercise vs aerobic training in Parkinson's disease. *Movement disorders : official journal of the Movement Disorder Society, 24*(8), 1132-1138. doi:10.1002/mds.22469

Sale, P., De Pandis, M. F., Le Pera, D., Sova, I., Cimolin, V., Ancillao, A., . . . Franceschini, M. (2013). Robot-assisted walking training for individuals with Parkinson's disease: a pilot randomized controlled trial. *BMC neurology, 13*, 50. doi:10.1186/1471-2377-13-50

San Martín Valenzuela, C., Moscardó, L. D., López-Pascual, J., Serra-Añó, P., & Tomás, J. M. (2020). Effects of Dual-Task Group Training on Gait, Cognitive Executive Function, and Quality of Life in People With Parkinson Disease: Results of Randomized Controlled DUALGAIT Trial. *Archives of physical medicine and rehabilitation, 101*(11). doi:10.1016/j.apmr.2020.07.008

Santos, L., Fernandez-Rio, J., Winge, K., Barragán-Pérez, B., González-Gómez, L., Rodríguez-Pérez, V., . . . Rodríguez-Gómez, J. (2017). Effects of progressive resistance exercise in akinetic-rigid Parkinson's disease patients: a randomized controlled trial. *European journal of physical and rehabilitation medicine, 53*(5), 651-663. doi:10.23736/S1973-9087.17.04572-5

Santos, L., Fernandez-Rio, J., Winge, K., Barragán-Pérez, B., Rodríguez-Pérez, V., González-Díez, V., . . . Rodríguez-Gómez, J. (2017). Effects of supervised slackline training on postural instability, freezing of gait, and falls efficacy in people with Parkinson's disease. *Disability and rehabilitation, 39*(16), 1573-1580. doi:10.1080/09638288.2016.1207104

Santos, P., Machado, T., Santos, L., Ribeiro, N., & Melo, A. (2019). Efficacy of the Nintendo Wii combination with Conventional Exercises in the rehabilitation of individuals with Parkinson's disease: A randomized clinical trial. *NeuroRehabilitation, 45*(2), 255-263. doi:10.3233/NRE-192771

Santos, S. M., da Silva, R. A., Terra, M. B., Almeida, I. A., de Melo, L. B., & Ferraz, H. B. (2017). Balance versus resistance training on postural control in patients with Parkinson's disease: a randomized controlled trial. *European journal of physical and rehabilitation medicine, 53*(2), 173-183. doi:10.23736/S1973-9087.16.04313-6

Schabrun, S. M., Lamont, R. M., & Brauer, S. G. (2016). Transcranial Direct Current Stimulation to Enhance Dual-Task Gait Training in Parkinson's Disease: A Pilot RCT. *PloS one, 11*(6), e0158497. doi:10.1371/journal.pone.0158497

Schenkman, M., Cutson, T. M., Kuchibhatla, M., Chandler, J., Pieper, C. F., Ray, L., & Laub, K. C. (1998). Exercise to improve spinal flexibility and function for people with Parkinson's disease: a randomized, controlled trial. *Journal of the American Geriatrics Society, 46*(10), 1207-1216. Retrieved from https://pubmed.ncbi.nlm.nih.gov/9777901

Schenkman, M., Hall, D. A., Barón, A. E., Schwartz, R. S., Mettler, P., & Kohrt, W. M. (2012). Exercise for people in early- or mid-stage Parkinson disease: a 16-month randomized controlled trial. *Physical therapy, 92*(11), 1395-1410. doi:10.2522/ptj.20110472

Schilling, B. K., Pfeiffer, R. F., Ledoux, M. S., Karlage, R. E., Bloomer, R. J., & Falvo, M. J. (2010). Effects of moderate-volume, high-load lower-body resistance training on strength and function in persons with Parkinson's disease: a pilot study. *Parkinson's disease, 2010*, 824734. doi:10.4061/2010/824734

Schlenstedt, C., Paschen, S., Kruse, A., Raethjen, J., Weisser, B., & Deuschl, G. (2015). Resistance versus Balance Training to Improve Postural Control in Parkinson's Disease: A Randomized Rater Blinded Controlled Study. *PloS one, 10*(10), e0140584. doi:10.1371/journal.pone.0140584

Schlenstedt, C., Paschen, S., Seuthe, J., Raethjen, J., Berg, D., Maetzler, W., & Deuschl, G. (2018). Moderate Frequency Resistance and Balance Training Do Not Improve Freezing of Gait in Parkinson's Disease: A Pilot Study. *Frontiers in neurology, 9*, 1084. doi:10.3389/fneur.2018.01084

Schlick, C., Ernst, A., Bötzel, K., Plate, A., Pelykh, O., & Ilmberger, J. (2016). Visual cues combined with treadmill training to improve gait performance in Parkinson's disease: a pilot randomized controlled trial. *Clinical rehabilitation, 30*(5), 463-471. doi:10.1177/0269215515588836

Sedaghati, P., Goudarzian, M., Daneshmandi, H., & Ardjmand, A. (2018). Effects of Alexander-based corrective techniques on forward flexed posture, risk of fall, and fear of falling in idiopathic Parkinson’s disease. *Archives of Neuroscience, 5*(2).

Serrao, M., Pierelli, F., Sinibaldi, E., Chini, G., Castiglia, S. F., Priori, M., . . . Monari, G. (2019). Progressive Modular Rebalancing System and Visual Cueing for Gait Rehabilitation in Parkinson's Disease: A Pilot, Randomized, Controlled Trial With Crossover. *Frontiers in neurology, 10*, 902. doi:10.3389/fneur.2019.00902

Shahmohammadi, R., Sharifi, G.-R., Melvin, J. M., & Sadeghi-Demneh, E. (2017). A comparison between aquatic and land-based physical exercise on postural sway and quality of life in people with Parkinson’s disease: a randomized controlled pilot study. *Sport Sciences for Health, 13*(2), 341-348.

Shen, X., & Mak, M. K. Y. (2012). Repetitive step training with preparatory signals improves stability limits in patients with Parkinson's disease. *Journal of rehabilitation medicine, 44*(11), 944-949. doi:10.2340/16501977-1056

Shen, X., & Mak, M. K. Y. (2015). Technology-assisted balance and gait training reduces falls in patients with Parkinson's disease: a randomized controlled trial with 12-month follow-up. *Neurorehabilitation and neural repair, 29*(2), 103-111. doi:10.1177/1545968314537559

Shih, M.-C., Wang, R.-Y., Cheng, S.-J., & Yang, Y.-R. (2016). Effects of a balance-based exergaming intervention using the Kinect sensor on posture stability in individuals with Parkinson's disease: a single-blinded randomized controlled trial. *Journal of neuroengineering and rehabilitation, 13*(1), 78. doi:10.1186/s12984-016-0185-y

Shulman, L. M., Katzel, L. I., Ivey, F. M., Sorkin, J. D., Favors, K., Anderson, K. E., . . . Macko, R. F. (2013). Randomized clinical trial of 3 types of physical exercise for patients with Parkinson disease. *JAMA neurology, 70*(2), 183-190. doi:10.1001/jamaneurol.2013.646

Silva, A. Z. d., & Israel, V. L. (2019). Effects of dual-task aquatic exercises on functional mobility, balance and gait of individuals with Parkinson's disease: A randomized clinical trial with a 3-month follow-up. *Complementary therapies in medicine, 42*, 119-124. doi:10.1016/j.ctim.2018.10.023

Silva-Batista, C., Corcos, D. M., Barroso, R., David, F. J., Kanegusuku, H., Forjaz, C., . . . Ugrinowitsch, C. (2017). Instability Resistance Training Improves Neuromuscular Outcome in Parkinson's Disease. *Medicine and science in sports and exercise, 49*(4), 652-660. doi:10.1249/MSS.0000000000001159

Silva-Batista, C., Corcos, D. M., Kanegusuku, H., Piemonte, M. E. P., Gobbi, L. T. B., de Lima-Pardini, A. C., . . . Ugrinowitsch, C. (2018). Balance and fear of falling in subjects with Parkinson's disease is improved after exercises with motor complexity. *Gait & posture, 61*, 90-97. doi:10.1016/j.gaitpost.2017.12.027

Silva-Batista, C., Corcos, D. M., Roschel, H., Kanegusuku, H., Gobbi, L. T. B., Piemonte, M. E. P., . . . Ugrinowitsch, C. (2016). Resistance Training with Instability for Patients with Parkinson's Disease. *Medicine and science in sports and exercise, 48*(9), 1678-1687. doi:10.1249/MSS.0000000000000945

Silva-Batista, C., de Lima-Pardini, A. C., Nucci, M. P., Coelho, D. B., Batista, A., Piemonte, M. E. P., . . . Ugrinowitsch, C. (2020). A Randomized, Controlled Trial of Exercise for Parkinsonian Individuals With Freezing of Gait. *Movement disorders : official journal of the Movement Disorder Society, 35*(9), 1607-1617. doi:10.1002/mds.28128

Smania, N., Corato, E., Tinazzi, M., Stanzani, C., Fiaschi, A., Girardi, P., & Gandolfi, M. (2010). Effect of balance training on postural instability in patients with idiopathic Parkinson's disease. *Neurorehabilitation and neural repair, 24*(9), 826-834. doi:10.1177/1545968310376057

Solla, P., Cugusi, L., Bertoli, M., Cereatti, A., Della Croce, U., Pani, D., . . . Mercuro, G. (2019). Sardinian Folk Dance for Individuals with Parkinson's Disease: A Randomized Controlled Pilot Trial. *Journal of alternative and complementary medicine (New York, N.Y.), 25*(3), 305-316. doi:10.1089/acm.2018.0413

Song, J., Paul, S. S., Caetano, M. J. D., Smith, S., Dibble, L. E., Love, R., . . . Allen, N. E. (2018). Home-based step training using videogame technology in people with Parkinson's disease: a single-blinded randomised controlled trial. *Clinical rehabilitation, 32*(3), 299-311. doi:10.1177/0269215517721593

Stack, E., Roberts, H., & Ashburn, A. (2012). The PIT: SToPP Trial-A Feasibility Randomised Controlled Trial of Home-Based Physiotherapy for People with Parkinson's Disease Using Video-Based Measures to Preserve Assessor Blinding. *Parkinson's disease, 2012*, 360231. doi:10.1155/2012/360231

Steib, S., Klamroth, S., Gaßner, H., Pasluosta, C., Eskofier, B., Winkler, J., . . . Pfeifer, K. (2017). Perturbation During Treadmill Training Improves Dynamic Balance and Gait in Parkinson's Disease: A Single-Blind Randomized Controlled Pilot Trial. *Neurorehabilitation and neural repair, 31*(8), 758-768. doi:10.1177/1545968317721976

Steib, S., Klamroth, S., Gaßner, H., Pasluosta, C., Eskofier, B., Winkler, J., . . . Pfeifer, K. (2019). Exploring gait adaptations to perturbed and conventional treadmill training in Parkinson's disease: Time-course, sustainability, and transfer. *Human movement science, 64*, 123-132. doi:10.1016/j.humov.2019.01.007

Stożek, J., Rudzińska, M., Pustułka-Piwnik, U., & Szczudlik, A. (2016). The effect of the rehabilitation program on balance, gait, physical performance and trunk rotation in Parkinson's disease. *Aging clinical and experimental research, 28*(6), 1169-1177. Retrieved from https://pubmed.ncbi.nlm.nih.gov/26661467

Strouwen, C., Molenaar, E. A. L. M., Münks, L., Keus, S. H. J., Zijlmans, J. C. M., Vandenberghe, W., . . . Nieuwboer, A. (2017). Training dual tasks together or apart in Parkinson's disease: Results from the DUALITY trial. *Movement disorders : official journal of the Movement Disorder Society, 32*(8), 1201-1210. doi:10.1002/mds.27014

Stuckenschneider, T., Helmich, I., Raabe-Oetker, A., Froböse, I., & Feodoroff, B. (2015). Active assistive forced exercise provides long-term improvement to gait velocity and stride length in patients bilaterally affected by Parkinson's disease. *Gait & posture, 42*(4), 485-490. doi:10.1016/j.gaitpost.2015.08.001

Terrens, A. F., Soh, S.-E., & Morgan, P. (2020). The safety and feasibility of a Halliwick style of aquatic physiotherapy for falls and balance dysfunction in people with Parkinson's Disease: A single blind pilot trial. *PloS one, 15*(7), e0236391. doi:10.1371/journal.pone.0236391

Thaut, M. H., McIntosh, G. C., Rice, R. R., Miller, R. A., Rathbun, J., & Brault, J. M. (1996). Rhythmic auditory stimulation in gait training for Parkinson's disease patients. *Movement disorders : official journal of the Movement Disorder Society, 11*(2), 193-200. Retrieved from https://pubmed.ncbi.nlm.nih.gov/8684391

Tollár, J., Nagy, F., & Hortobágyi, T. (2019). Vastly Different Exercise Programs Similarly Improve Parkinsonian Symptoms: A Randomized Clinical Trial. *Gerontology, 65*(2), 120-127. doi:10.1159/000493127

Tollár, J., Nagy, F., Kovács, N., & Hortobágyi, T. (2018). A High-Intensity Multicomponent Agility Intervention Improves Parkinson Patients' Clinical and Motor Symptoms. *Archives of physical medicine and rehabilitation, 99*(12). doi:10.1016/j.apmr.2018.05.007

Tollár, J., Nagy, F., Kovács, N., & Hortobágyi, T. (2019). Two-Year Agility Maintenance Training Slows the Progression of Parkinsonian Symptoms. *Medicine and science in sports and exercise, 51*(2), 237-245. doi:10.1249/MSS.0000000000001793

van den Heuvel, M. R. C., Kwakkel, G., Beek, P. J., Berendse, H. W., Daffertshofer, A., & van Wegen, E. E. H. (2014). Effects of augmented visual feedback during balance training in Parkinson's disease: a pilot randomized clinical trial. *Parkinsonism & related disorders, 20*(12), 1352-1358. doi:10.1016/j.parkreldis.2014.09.022

van der Kolk, N. M., de Vries, N. M., Kessels, R. P. C., Joosten, H., Zwinderman, A. H., Post, B., & Bloem, B. R. (2019). Effectiveness of home-based and remotely supervised aerobic exercise in Parkinson's disease: a double-blind, randomised controlled trial. *The Lancet. Neurology, 18*(11). doi:10.1016/S1474-4422(19)30285-6

van der Kolk, N. M., de Vries, N. M., Penko, A. L., van der Vlugt, M., Mulder, A. A., Post, B., . . . Bloem, B. R. (2018). A remotely supervised home-based aerobic exercise programme is feasible for patients with Parkinson's disease: results of a small randomised feasibility trial. *Journal of neurology, neurosurgery, and psychiatry, 89*(9), 1003-1005. doi:10.1136/jnnp-2017-315728

Van Puymbroeck, M., Walter, A. A., Hawkins, B. L., Sharp, J. L., Woschkolup, K., Urrea-Mendoza, E., . . . Schmid, A. A. (2018). Functional Improvements in Parkinson's Disease Following a Randomized Trial of Yoga. *Evidence-based complementary and alternative medicine : eCAM, 2018*, 8516351. doi:10.1155/2018/8516351

Vergara-Diaz, G., Osypiuk, K., Hausdorff, J. M., Bonato, P., Gow, B. J., Miranda, J. G., . . . Wayne, P. M. (2018). Tai Chi for Reducing Dual-task Gait Variability, a Potential Mediator of Fall Risk in Parkinson's Disease: A Pilot Randomized Controlled Trial. *Global advances in health and medicine, 7*, 2164956118775385. doi:10.1177/2164956118775385

Vieira de Moraes Filho, A., Chaves, S. N., Martins, W. R., Tolentino, G. P., de Cássia Pereira Pinto Homem, R., Landim de Farias, G., . . . Jacó de Oliveira, R. (2020). Progressive Resistance Training Improves Bradykinesia, Motor Symptoms and Functional Performance in Patients with Parkinson's Disease. *Clinical interventions in aging, 15*, 87-95. doi:10.2147/CIA.S231359

Vieira-Yano, B., Martini, D. N., Horak, F. B., de Lima-Pardini, A., Almeida, F., Santana, V. P., . . . Silva-Batista, C. (2021). The Adapted Resistance Training with Instability Randomized Controlled Trial for Gait Automaticity. *Movement disorders : official journal of the Movement Disorder Society, 36*(1), 152-163. doi:10.1002/mds.28298

Vitório, R., Teixeira-Arroyo, C., Lirani-Silva, E., Barbieri, F. A., Caetano, M. J. D., Gobbi, S., . . . Teresa Bucken Gobbi, L. (2011). Effects of 6-month, Multimodal Exercise Program on Clinical and Gait Parameters of Patients with Idiopathic Parkinson's Disease: A Pilot Study. *ISRN neurology, 2011*, 714947. doi:10.5402/2011/714947

Vivas, J., Arias, P., & Cudeiro, J. (2011). Aquatic therapy versus conventional land-based therapy for Parkinson's disease: an open-label pilot study. *Archives of physical medicine and rehabilitation, 92*(8), 1202-1210. doi:10.1016/j.apmr.2011.03.017

Volpe, D., Giantin, M. G., & Fasano, A. (2014). A wearable proprioceptive stabilizer (Equistasi®) for rehabilitation of postural instability in Parkinson's disease: a phase II randomized double-blind, double-dummy, controlled study. *PloS one, 9*(11), e112065. doi:10.1371/journal.pone.0112065

Volpe, D., Giantin, M. G., Maestri, R., & Frazzitta, G. (2014). Comparing the effects of hydrotherapy and land-based therapy on balance in patients with Parkinson's disease: a randomized controlled pilot study. *Clinical rehabilitation, 28*(12), 1210-1217. doi:10.1177/0269215514536060

Volpe, D., Giantin, M. G., Manuela, P., Filippetto, C., Pelosin, E., Abbruzzese, G., & Antonini, A. (2017). Water-based vs. non-water-based physiotherapy for rehabilitation of postural deformities in Parkinson's disease: a randomized controlled pilot study. *Clinical rehabilitation, 31*(8), 1107-1115. doi:10.1177/0269215516664122

Volpe, D., Signorini, M., Marchetto, A., Lynch, T., & Morris, M. E. (2013). A comparison of Irish set dancing and exercises for people with Parkinson's disease: a phase II feasibility study. *BMC geriatrics, 13*, 54. doi:10.1186/1471-2318-13-54

White, D. K., Wagenaar, R. C., Ellis, T. D., & Tickle-Degnen, L. (2009). Changes in walking activity and endurance following rehabilitation for people with Parkinson disease. *Archives of physical medicine and rehabilitation, 90*(1), 43-50. doi:10.1016/j.apmr.2008.06.034

Wong-Yu, I. S., & Mak, M. K. (2015). Task- and Context-Specific Balance Training Program Enhances Dynamic Balance and Functional Performance in Parkinsonian Nonfallers: A Randomized Controlled Trial With Six-Month Follow-Up. *Archives of physical medicine and rehabilitation, 96*(12), 2103-2111. doi:10.1016/j.apmr.2015.08.409

Wong-Yu, I. S. K., & Mak, M. K. Y. (2015). Multi-dimensional balance training programme improves balance and gait performance in people with Parkinson's disease: A pragmatic randomized controlled trial with 12-month follow-up. *Parkinsonism & related disorders, 21*(6), 615-621. doi:10.1016/j.parkreldis.2015.03.022

Xiao, C., Zhuang, Y., & Kang, Y. (2016). Effect of Health Qigong Baduanjin on Fall Prevention in Individuals with Parkinson's Disease. *Journal of the American Geriatrics Society, 64*(11), e227-e228. doi:10.1111/jgs.14438

Xiao, C.-M., & Zhuang, Y.-C. (2016). Effect of health Baduanjin Qigong for mild to moderate Parkinson's disease. *Geriatrics & gerontology international, 16*(8), 911-919. doi:10.1111/ggi.12571

Yang, W.-C., Wang, H.-K., Wu, R.-M., Lo, C.-S., & Lin, K.-H. (2016). Home-based virtual reality balance training and conventional balance training in Parkinson's disease: A randomized controlled trial. *Journal of the Formosan Medical Association = Taiwan yi zhi, 115*(9), 734-743. doi:10.1016/j.jfma.2015.07.012

Yang, Y.-R., Cheng, S.-J., Lee, Y.-J., Liu, Y.-C., & Wang, R.-Y. (2019). Cognitive and motor dual task gait training exerted specific training effects on dual task gait performance in individuals with Parkinson's disease: A randomized controlled pilot study. *PloS one, 14*(6), e0218180. doi:10.1371/journal.pone.0218180

Yang, Y.-R., Lee, Y.-Y., Cheng, S.-J., & Wang, R.-Y. (2010). Downhill walking training in individuals with Parkinson's disease: a randomized controlled trial. *American journal of physical medicine & rehabilitation, 89*(9), 706-714. doi:10.1097/PHM.0b013e3181e721c5

Yen, C.-Y., Lin, K.-H., Hu, M.-H., Wu, R.-M., Lu, T.-W., & Lin, C.-H. (2011). Effects of virtual reality-augmented balance training on sensory organization and attentional demand for postural control in people with Parkinson disease: a randomized controlled trial. *Physical therapy, 91*(6), 862-874. doi:10.2522/ptj.20100050

Yi-zhao, W., Hua, Z., Shi-chun, F., Wei-jia, H., & ZHANG, Y. (2017). Effect of water-based exercise on motor function, balance function and walking ability in patients with Parkinson's disease. *Chinese Journal of Contemporary Neurology & Neurosurgery, 17*(5), 346.

Yotnuengnit, P., Bhidayasiri, R., Donkhan, R., Chaluaysrimuang, J., & Piravej, K. (2018). Effects of Transcranial Direct Current Stimulation Plus Physical Therapy on Gait in Patients With Parkinson Disease: A Randomized Controlled Trial. *American journal of physical medicine & rehabilitation, 97*(1). doi:10.1097/PHM.0000000000000783

Youm, C., Kim, Y., Noh, B., Lee, M., Kim, J., & Cheon, S.-M. (2020). Impact of Trunk Resistance and Stretching Exercise on Fall-Related Factors in Patients with Parkinson's Disease: A Randomized Controlled Pilot Study. *Sensors (Basel, Switzerland), 20*(15). doi:10.3390/s20154106

Zhang, T.-Y., Hu, Y., Nie, Z.-Y., Jin, R.-X., Chen, F., Guan, Q., . . . Jin, L.-J. (2015). Effects of Tai Chi and Multimodal Exercise Training on Movement and Balance Function in Mild to Moderate Idiopathic Parkinson Disease. *American journal of physical medicine & rehabilitation, 94*(10 Suppl 1), 921-929. doi:10.1097/PHM.0000000000000351

NCT01156714. Exercise and Cognitive Training in Parkinson's Disease: https://clinicaltrials.gov/ct2/show/study/NCT01156714</web_address>. 2010

NCT01768832. Exercise and Parkinson's: Comparing Interventions and Exploring Neural Mechanisms: https://clinicaltrials.gov/ct2/show/study/NCT01768832</web_address>. 2013

# Appendix 5: The risk of bias assessment for the individual included studies.

| Author | Random sequence generation (selection bias) | Allocation concealment (selection bias) | Blinding of participants and personnel (performance bias) | Blinding of outcome assessment (detection bias) | uncomplete outcome data (attrition bias) | Selective reporting (reporting bias) | other bias |
| --- | --- | --- | --- | --- | --- | --- | --- |
| Abraham, Hart ^1^ | ？ | + | + | - | ？ | + | + |
| Allen, Canning ^2^ | + | + | ？ | + | + | + | + |
| Amano, Nocera ^3^ | ？ | ？ | + | ？ | + | + | + |
| Arcolin, Pisano ^4^ | ？ | + | + | ？ | + | + | + |
| Arfa-Fatollahkhani, Safar Cherati ^5^ | + | + | ？ | ? | ？ | + | + |
| Ashburn, Fazakarley ^6^ | ？ | + | - | + | ？ | + | + |
| Atan, Özyemişci Taşkıran ^7^ | + | + | + | + | + | + | + |
| Bakhshayesh, Sayyar ^8^ | + | + | ？ | ？ | + | + | + |
| Bang and Shin ^9^ | + | ？ | ? | + | ？ | + | + |
| Beck, Intzandt ^10^ | ？ | + | ？ | + | + | + | + |
| Bekkers, Mirelman ^11^ | ？ | + | + | + | + | + | + |
| Bello, Sanchez ^12^ | + | ？ | ？ | - | ？ | - | + |
| Xiao, Zhuang ^13^ | ？ | ？ | ？ | ？ | ？ | + | + |
| Xiao and Zhuang ^14^ | ？ | + | ? | ？ | ？ | + | + |
| Cabrera-Martos, Jiménez-Martín ^15^ | ？ | + | ？ | ？ | ？ | + | - |
| Cakit, Saracoglu ^16^ | + | + | - | + | ？ | + | + |
| Calabrò, Naro ^17^ | + | + | ？ | + | + | + | + |
| Cancela, Mollinedo ^18^ | ？ | + | + | - | + | ？ | + |
| Canning, Allen ^19^ | ？ | + | ? | + | ？ | + | + |
| Capato, de Vries ^20^ | + | + | + | ？ | + | + | ？ |
| Capato, Nonnekes ^21^ | ？ | + | + | + | + | ？ | + |
| Capecci, Serpicelli ^22^ | + | + | + | ？ | ？ | + | + |
| Capecci, Pournajaf ^23^ | ？ | + | + | + | ？ | + | + |
| Carda, Invernizzi ^24^ | + | + | ？ | + | ？ | + | + |
| Carpinella, Cattaneo ^25^ | + | ？ | ？ | - | ？ | + | + |
| Carvalho, Barbirato ^26^ | + | + | ？ | ？ | + | ？ | + |
| Cheng, Yang ^27^ | ？ | ？ | + | - | + | + | + |
| Cheng, Yang ^28^ | + | + | + | ？ | ？ | + | + |
| Cherup, Strand ^29^ | + | ？ | ？ | + | + | + | + |
| Chivers Seymour, Pickering ^30^ | + | + | ？ | + | ？ | + | + |
| Choi ^31^ | ？ | + | ? | + | + | + | + |
| Clerici, Maestri ^32^ | ？ | + | ？ | + | + | + | + |
| Combs, Diehl ^33^ | ？ | + | + | ？ | ？ | + | + |
| Conradsson, Löfgren ^34^ | ？ | ？ | ？ | ？ | ？ | + | + |
| Conradsson, Löfgren ^34^ | + | + | - | - | ？ | + | + |
| Costa-Ribeiro, Maux ^35^ | + | + | + | + | ？ | + | + |
| Cugusi, Solla ^36^ | ？ | + | ？ | - | + | + | + |
| Daneshmandi, Sayyar ^37^ | + | + | ？ | ？ | + | - | + |
| de Bruin, Doan ^38^ | ？ | + | ？ | + | + | + | + |
| de Lima, Ferreira-Moraes ^39^ | + | + | ？ | ？ | - | + | + |
| de Melo, Kleiner ^40^ | + | + | ？ | + | ？ | + | + |
| Demonceau, Maquet ^41^ | + | + | ？ | - | ？ | + | + |
| Droby, Maidan ^42^ | ？ | ？ | ？ | - | + | + | + |
| Ebersbach, Edler ^43^ | + | + | + | ？ | + | - | ？ |
| Ebersbach, Ebersbach ^44^ | ？ | ？ | ？ | ? | + | ？ | ？ |
| Ellis, de Goede ^45^ | ？ | + | ？ | ？ | ？ | + | + |
| El-Tamawy, Darwish ^46^ | ？ | + | + | + | - | + | + |
| Feng, Li ^47^ | ？ | + | ? | ? | ？ | + | + |
| Fernandes, Rocha ^48^ | + | + | ? | ? | + | + | + |
| Ferraz, Trippo ^49^ | ？ | + | ？ | + | + | + | + |
| Fil-Balkan, Salci ^50^ | ？ | ？ | ？ | ？ | ？ | + | + |
| Fisher, Wu ^51^ | ？ | + | ？ | - | + | - | + |
| Franzoni, Monteiro ^52^ | ？ | + | ？ | + | + | + | + |
| Furnari, Calabrò ^53^ | ？ | ？ | ？ | ？ | ？ | + | + |
| Frazzitta, Bossio ^54^ | ？ | + | ？ | - | + | + | + |
| Galli, Cimolin ^55^ | ？ | + | + | + | ？ | + | + |
| Gandolfi, Geroin ^56^ | ？ | ? | ? | + | + | ？ | + |
| Gandolfi, Tinazzi ^57^ | ？ | + | - | ？ | + | + | + |
| Ganesan, Sathyaprabha ^58^ | ？ | ？ | ？ | ？ | + | + | + |
| Gao, Leung ^59^ | + | ？ | + | ？ | + | + | + |
| Ghielen, van Wegen ^60^ | ？ | ？ | ？ | ？ | + | + | + |
| Ginis, Nieuwboer ^61^ | + | + | - | ？ | + | + | + |
| Frazzitta, Maestri ^62^ | ？ | + | ？ | ？ | ？ | + | ？ |
| Goodwin, Richards ^63^ | + | + | + | - | + | ？ | + |
| Granziera, Alessandri ^64^ | + | + | ？ | ？ | + | + | + |
| Grobbelaar, Venter ^65^ | ？ | + | + | ？ | ？ | ？ | + |
| Hackney, Kantorovich ^66^ | ？ | + | + | ？ | ？ | + | + |
| Hackney and Earhart ^67^ | ？ | + | ? | + | ？ | + | ？ |
| Hackney and Earhart ^68^ | + | + | ？ | - | + | + | + |
| Harro, Shoemaker ^69^ | + | + | + | - | + | + | + |
| Hashimoto, Takabatake ^70^ | + | + | ？ | ？ | + | + | + |
| Hass, Buckley ^71^ | ？ | ？ | ？ | ？ | + | + | + |
| Helgerud, Thomsen ^72^ | ？ | + | + | + | + | + | + |
| Hirsch, Toole ^73^ | ？ | ？ | ？ | ？ | + | + | + |
| Hubble, Naughton ^74^ | ？ | + | ？ | ？ | ？ | + | + |
| Wong-Yu and Mak ^75^ | + | ？ | ？ | + | + | + | + |
| Wong-Yu and Mak ^76^ | + | + | ？ | + | ？ | + | + |
| Johansson, Freidle ^77^ | ？ | + | + | ？ | + | ？ | + |
| Joseph, Brodin ^78^ | ？ | ？ | + | + | + | + | + |
| Jung, Hasegawa ^79^ | + | + | + | - | + | + | + |
| Kadivar, Corcos ^80^ | + | + | - | ？ | + | + | + |
| Khalil, Busse ^81^ | + | + | ？ | + | + | + | + |
| Khuzema, Brammatha ^82^ | ？ | ？ | - | - | + | + | ? |
| King, Salarian ^83^ | + | + | + | + | + | + | + |
| Kunkel, Fitton ^84^ | + | + | ？ | + | ？ | + | + |
| Kurt, Büyükturan ^85^ | + | + | ？ | ？ | ？ | + | + |
| Kurtais, Kutlay ^86^ | + | + | ？ | ？ | + | + | + |
| Kwok, Kwan ^87^ | + | + | ？ | ？ | ？ | + | + |
| Santos, Fernandez-Rio ^88^ | ？ | + | + | ？ | ？ | + | + |
| Santos, Fernandez-Rio ^89^ | ？ | + | ？ | ？ | + | + | + |
| Landers, Hatlevig ^90^ | ？ | ？ | - | ？ | + | + | + |
| Leal, Abrahin ^91^ | ？ | + | ？ | ？ | + | + | + |
| Lee, Kim ^92^ | + | ？ | ？ | - | + | + | + |
| Li, Harmer ^93^ | ？ | + | ？ | ？ | + | + | + |
| Liao, Yang ^94^ | + | + | + | ？ | + | + | + |
| Liu, Chen ^95^ | + | + | ？ | ？ | + | + | + |
| Löfgren, Conradsson ^96^ | + | + | - | - | + | ？ | + |
| Schenkman, Cutson ^97^ | + | + | - | ？ | - | + | + |
| Maidan, Nieuwhof ^98^ | ？ | + | ？ | + | - | + | ？ |
| Schenkman, Hall ^99^ | ？ | + | + | + | + | + | + |
| McNeely, Mai ^100^ | ？ | + | ？ | + | + | - | + |
| Medijainen, Pääsuke ^101^ | ？ | ？ | ？ | ？ | + | + | ？ |
| Michels, Dubaz ^102^ | + | + | ？ | - | ？ | + | + |
| Michels, Dubaz ^102^ | + | + | ？ | ？ | + | - | + |
| Miyai, Fujimoto ^103^ | ？ | + | + | + | + | + | + |
| Mollinedo-Cardalda, Cancela-Carral ^104^ | ？ | + | ？ | ？ | ？ | + | + |
| Monticone, Ambrosini ^105^ | + | + | ？ | ？ | + | + | + |
| Morris, Iansek ^106^ | + | + | + | ？ | ？ | + | + |
| Morris, Menz ^107^ | + | + | ？ | ？ | + | - | ？ |
| Morrone, Miccinilli ^108^ | + | + | + | - | + | + | + |
| Myers, Harrison ^109^ | ？ | + | ？ | ？ | ？ | + | + |
| Ni, Signorile ^110^ | ？ | ？ | ？ | - | + | + | + |
| Nieuwboer, Kwakkel ^111^ | + | + | ？ | + | + | + | + |
| Oliveira, Iraci ^112^ | ？ | ？ | + | + | + | + | + |
| Ortiz-Rubio, Cabrera-Martos ^113^ | + | + | - | + | ？ | + | + |
| Palamara, Gotti ^114^ | + | + | ？ | ？ | + | + | + |
| Paolucci, Zangrando ^115^ | + | + | + | - | ？ | + | + |
| Park, Zid ^116^ | + | + | ？ | ？ | + | ？ | + |
| Paul, Canning ^117^ | + | ？ | ？ | ？ | + | ？ | + |
| Pazzaglia, Imbimbo ^118^ | + | + | + | - | + | + | + |
| Pérez de la Cruz ^119^ | ？ | + | + | ？ | ？ | + | + |
| Pérez-de la Cruz ^120^ | + | + | + | - | ？ | + | + |
| Picelli, Melotti ^121^ | ？ | + | ？ | + | + | + | + |
| Picelli, Melotti ^122^ | + | + | ？ | - | ？ | + | + |
| Picelli, Melotti ^123^ | ？ | + | ？ | + | + | - | + |
| Picelli, Varalta ^124^ | + | + | ？ | ？ | + | ？ | + |
| Pohl, Dizdar ^125^ | + | + | + | - | + | + | + |
| Pohl, Wressle ^126^ | + | ？ | + | ？ | + | + | + |
| Poliakoff, Galpin ^127^ | + | + | ？ | ？ | ？ | + | + |
| Pompeu, Mendes ^128^ | ？ | + | ？ | + | + | + | + |
| Prodoehl, Rafferty ^129^ | ？ | + | + | ？ | + | + | + |
| Protas, Mitchell ^130^ | ？ | ？ | - | + | + | + | + |
| Qutubuddin, Cifu ^131^ | + | + | ？ | ？ | ？ | + | - |
| Rafferty, Prodoehl ^132^ | + | + | ？ | - | ？ | + | + |
| Rennie, Opheim ^133^ | + | ？ | ？ | ？ | + | + | + |
| Ribas, Alves da Silva ^134^ | + | + | ? | + | + | + | + |
| Rios Romenets, Anang ^135^ | + | + | ？ | ？ | ？ | + | + |
| Rosenfeldt, Penko ^136^ | ？ | + | ？ | + | + | + | + |
| Santos, da Silva ^137^ | ？ | + | + | + | ？ | + | + |
| Sacheli, Neva ^138^ | + | + | + | ？ | + | + | + |
| Sage and Almeida ^139^ | + | + | + | ？ | + | + | + |
| Sale, De Pandis ^140^ | + | + | ？ | + | + | - | + |
| San Martín Valenzuela, Moscardó ^141^ | + | + | + | - | + | ？ | + |
| Santos, Machado ^142^ | + | + | ？ | + | + | + | + |
| Schabrun, Lamont ^143^ | + | + | + | - | + | ？ | + |
| Schilling, Pfeiffer ^144^ | ？ | + | ？ | ？ | + | - | - |
| Schlenstedt, Paschen ^145^ | + | + | + | + | + | + | + |
| Schlenstedt, Paschen ^146^ | + | + | + | + | ？ | + | + |
| Schlick, Ernst ^147^ | ？ | ？ | ？ | ？ | + | - | + |
| Sedaghati, Goudarzian ^148^ | ？ | ？ | ？ | ？ | - | + | + |
| Serrao, Pierelli ^149^ | ？ | ？ | ？ | ？ | + | + | ？ |
| Shahmohammadi, Sharifi ^150^ | ？ | ？ | + | + | ？ | + | + |
| Shen and Mak ^151^ | + | + | ？ | + | + | + | + |
| Shen and Mak ^152^ | ？ | + | - | ？ | + | + | + |
| Shih, Wang ^153^ | ？ | + | + | ？ | + | + | + |
| Shulman, Katzel ^154^ | + | + | + | ？ | + | - | + |
| Silva and Israel ^155^ | + | + | ？ | - | + | + | + |
| Silva-Batista, Corcos ^156^ | ？ | ？ | ？ | ？ | + | + | + |
| Silva-Batista, Corcos ^157^ | + | ？ | ？ | ？ | + | + | + |
| Silva-Batista, Corcos ^158^ | ？ | ？ | ？ | - | + | + | + |
| Silva-Batista, de Lima-Pardini ^159^ | ？ | ？ | ？ | ？ | + | + | + |
| Smania, Corato ^160^ | + | + | ？ | ？ | + | + | - |
| Solla, Cugusi ^161^ | + | + | ？ | - | + | - | + |
| Song, Paul ^162^ | ？ | ？ | ？ | + | + | + | - |
| Stack, Roberts ^163^ | + | + | ？ | + | + | + | + |
| Steib, Klamroth ^164^ | + | + | - | + | + | + | + |
| Steib, Klamroth ^165^ | + | + | ? | + | + | + | + |
| Stożek, Rudzińska ^166^ | ？ | + | ？ | ？ | ？ | + | + |
| Strouwen, Molenaar ^167^ | + | ？ | + | + | + | + | + |
| Stuckenschneider, Helmich ^168^ | + | + | ？ | ？ | + | + | + |
| Terrens, Soh ^169^ | + | + | + | + | - | + | + |
| Thaut, McIntosh ^170^ | ？ | + | ？ | ？ | ？ | ？ | + |
| Tollár, Nagy ^171^ | ？ | + | ？ | - | ？ | + | + |
| Tollár, Nagy ^172^ | + | + | + | + | ？ | ？ | + |
| Tollár, Nagy ^173^ | + | + | ？ | ？ | ？ | + | + |
| van den Heuvel, Kwakkel ^174^ | ？ | - | ？ | + | + | ？ | + |
| van der Kolk, de Vries ^175^ | + | + | ？ | ？ | + | + | + |
| van der Kolk, de Vries ^176^ | + | + | + | + | + | + | - |
| Van Puymbroeck, Walter ^177^ | + | + | ？ | ？ | + | + | + |
| Vergara-Diaz, Osypiuk ^178^ | ？ | ？ | ？ | ？ | + | + | - |
| Vieira de Moraes Filho, Chaves ^179^ | ？ | + | + | ？ | + | ？ | + |
| Vieira-Yano, Martini ^180^ | ？ | ？ | - | ？ | ？ | + | + |
| Vitório, Teixeira-Arroyo ^181^ | ？ | ？ | ？ | + | + | + | + |
| Vivas, Arias ^182^ | ？ | + | + | ？ | + | ？ | + |
| Volpe, Signorini ^183^ | + | + | + | ？ | + | + | + |
| Volpe, Giantin ^184^ | + | + | + | + | ？ | ？ | + |
| Volpe, Giantin ^185^ | + | + | + | + | ？ | + | ？ |
| Volpe, Giantin ^186^ | + | + | + | ？ | + | + | + |
| Yang, Wang ^187^ | ？ | ？ | ？ | ？ | + | + | + |
| White, Wagenaar ^188^ | ？ | + | ？ | ？ | + | + | + |
| Yang, Lee ^189^ | ？ | + | ？ | - | + | - | + |
| Yang, Cheng ^190^ | ？ | + | ? | - | ？ | + | + |
| Yen, Lin ^191^ | + | + | ？ | ？ | + | + | + |
| Yi-zhao, Hua ^192^ | ？ | ？ | - | ？ | + | + | + |
| Yotnuengnit, Bhidayasiri ^193^ | + | + | + | ？ | ？ | + | + |
| Youm, Kim ^194^ | + | + | ？ | + | + | + | + |
| Zhang, Hu ^195^ | + | + | - | ？ | + | + | + |
| NCT01506479 | ？ | + | ？ | + | + | + | + |
| NCT01768832 | ？ | + | ？ | + | - | + | + |

# Appendix 6: Network meta-analysis results

## Table 6.1: League Table of Balance Test Batteries

| **BWS** | NA | NA | NA | NA | NA | NA | NA | NA | NA | NA | NA | NA | NA | NA | NA | NA | **1.13 ( 0.35; 1.91)** | NA | NA | NA | NA | NA | NA | NA |
| --- | --- | --- | --- | --- | --- | --- | --- | --- | --- | --- | --- | --- | --- | --- | --- | --- | --- | --- | --- | --- | --- | --- | --- | --- |
| 0.69 (-0.21; 1.60) | **MD** | NA | NA | NA | -0.22 (-1.18; 0.74) | 0.15 (-0.44; 0.74) | NA | NA | NA | NA | NA | 0.80 (-0.05; 1.64) | NA | NA | NA | NA | NA | NA | NA | NA | NA | **0.99 ( 0.30; 1.68)** | NA | 0.34 (-0.67; 1.34) |
| 0.64 (-0.40; 1.68) | -0.05 (-0.79; 0.68) | **PIL** | NA | NA | NA | NA | NA | NA | NA | NA | NA | NA | NA | NA | NA | NA | NA | NA | NA | NA | 0.70 (-0.18; 1.58) | NA | NA | **0.89 ( 0.00; 1.78)** |
| 0.71 (-0.28; 1.70) | 0.01 (-0.69; 0.72) | 0.07 (-0.81; 0.94) | **NW** | NA | NA | NA | NA | NA | NA | NA | NA | NA | NA | -0.20 (-1.12; 0.72) | NA | NA | 0.75 (-0.28; 1.78) | NA | NA | NA | NA | NA | NA | **1.29 ( 0.20; 2.38)** |
| 0.85 (-0.05; 1.74) | 0.15 (-0.35; 0.65) | 0.20 (-0.52; 0.93) | 0.14 (-0.56; 0.83) | **DA** | NA | NA | NA | NA | NA | -0.03 (-0.79; 0.72) | NA | NA | NA | NA | NA | NA | NA | NA | NA | NA | NA | **0.74 ( 0.10; 1.37)** | NA | **0.77 ( 0.34; 1.20)** |
| **0.88 ( 0.03; 1.72)** | 0.18 (-0.25; 0.61) | 0.23 (-0.44; 0.90) | 0.17 (-0.47; 0.80) | 0.03 (-0.40; 0.46) | **ECA** | NA | NA | NA | NA | NA | -0.05 (-1.03; 0.93) | NA | NA | -0.05 (-0.74; 0.65) | NA | NA | 0.12 (-0.47; 0.71) | NA | NA | NA | **0.61 ( 0.28; 0.94)** | 1.04 (-0.18; 2.27) | NA | **0.56 ( 0.12; 1.00)** |
| 0.87 (-0.01; 1.74) | 0.17 (-0.21; 0.56) | 0.22 (-0.47; 0.92) | 0.16 (-0.51; 0.83) | 0.02 (-0.42; 0.47) | -0.01 (-0.38; 0.37) | **AQE** | NA | NA | NA | NA | NA | 0.14 (-0.32; 0.59) | NA | NA | NA | NA | NA | NA | NA | NA | 0.71 (-0.13; 1.55) | **0.43 ( 0.00; 0.86)** | NA | **1.56 ( 0.53; 2.60)** |
| 0.87 (-0.04; 1.78) | 0.18 (-0.37; 0.73) | 0.23 (-0.51; 0.97) | 0.16 (-0.55; 0.87) | 0.02 (-0.51; 0.56) | -0.00 (-0.46; 0.45) | 0.00 (-0.49; 0.50) | **RA** | NA | 0.00 (-0.68; 0.68) | NA | NA | NA | NA | 0.35 (-0.44; 1.13) | NA | NA | NA | NA | NA | NA | 0.30 (-0.26; 0.87) | NA | **1.28 ( 0.37; 2.20)** | NA |
| 0.83 (-0.46; 2.13) | 0.14 (-0.94; 1.22) | 0.19 (-1.00; 1.38) | 0.12 (-1.05; 1.29) | -0.01 (-1.08; 1.06) | -0.04 (-1.08; 1.00) | -0.03 (-1.09; 1.02) | -0.04 (-1.10; 1.03) | **WBV** | 0.11 (-0.87; 1.09) | NA | NA | NA | NA | NA | NA | NA | NA | NA | NA | NA | NA | NA | NA | NA |
| **0.94 ( 0.09; 1.79)** | 0.25 (-0.20; 0.70) | 0.30 (-0.38; 0.97) | 0.23 (-0.41; 0.87) | 0.09 (-0.33; 0.52) | 0.06 (-0.28; 0.41) | 0.07 (-0.31; 0.46) | 0.07 (-0.35; 0.49) | 0.11 (-0.87; 1.09) | **DT** | NA | NA | NA | NA | NA | NA | -0.39 (-1.22; 0.45) | 0.45 (-0.36; 1.25) | NA | NA | NA | NA | NA | **0.99 ( 0.49; 1.50)** | **0.49 ( 0.17; 0.80)** |
| **1.02 ( 0.14; 1.90)** | 0.32 (-0.19; 0.83) | 0.37 (-0.35; 1.10) | 0.31 (-0.38; 0.99) | 0.17 (-0.27; 0.61) | 0.14 (-0.28; 0.56) | 0.15 (-0.31; 0.60) | 0.15 (-0.38; 0.67) | 0.18 (-0.88; 1.25) | 0.08 (-0.34; 0.49) | **TA** | NA | NA | NA | NA | NA | NA | 0.00 (-0.67; 0.67) | NA | 0.68 (-0.36; 1.73) | NA | NA | NA | 0.26 (-0.43; 0.95) | 0.47 (-0.08; 1.02) |
| 1.01 (-0.04; 2.06) | 0.32 (-0.43; 1.06) | 0.37 (-0.53; 1.27) | 0.30 (-0.58; 1.18) | 0.16 (-0.57; 0.90) | 0.13 (-0.53; 0.80) | 0.14 (-0.57; 0.85) | 0.14 (-0.62; 0.90) | 0.18 (-1.02; 1.38) | 0.07 (-0.62; 0.76) | -0.01 (-0.74; 0.73) | **ICA** | NA | NA | NA | NA | NA | NA | NA | NA | NA | 0.20 (-0.78; 1.18) | NA | NA | 0.50 (-0.50; 1.49) |
| **1.06 ( 0.21; 1.90)** | 0.36 (-0.03; 0.75) | 0.41 (-0.25; 1.08) | 0.35 (-0.28; 0.98) | 0.21 (-0.18; 0.60) | 0.18 (-0.13; 0.50) | 0.19 (-0.11; 0.49) | 0.19 (-0.26; 0.64) | 0.22 (-0.81; 1.25) | 0.12 (-0.20; 0.43) | 0.04 (-0.36; 0.44) | 0.05 (-0.63; 0.73) | **MC** | NA | 0.04 (-0.82; 0.89) | 0.20 (-0.59; 0.98) | NA | 0.29 (-0.50; 1.08) | NA | NA | NA | NA | **0.62 ( 0.00; 1.24)** | NA | **0.46 ( 0.21; 0.70)** |
| 1.10 (-0.07; 2.26) | 0.40 (-0.50; 1.30) | 0.45 (-0.58; 1.49) | 0.39 (-0.63; 1.40) | 0.25 (-0.64; 1.14) | 0.22 (-0.63; 1.08) | 0.23 (-0.64; 1.10) | 0.23 (-0.69; 1.14) | 0.26 (-1.04; 1.56) | 0.16 (-0.70; 1.01) | 0.08 (-0.81; 0.97) | 0.09 (-0.96; 1.13) | 0.04 (-0.80; 0.88) | **PT** | NA | NA | NA | NA | NA | NA | 0.03 (-0.86; 0.92) | NA | NA | NA | 0.63 (-0.33; 1.58) |
| **1.09 ( 0.23; 1.94)** | 0.39 (-0.08; 0.86) | 0.44 (-0.25; 1.13) | 0.38 (-0.23; 0.99) | 0.24 (-0.22; 0.70) | 0.21 (-0.13; 0.55) | 0.22 (-0.19; 0.63) | 0.22 (-0.23; 0.66) | 0.25 (-0.79; 1.30) | 0.15 (-0.21; 0.51) | 0.07 (-0.37; 0.51) | 0.08 (-0.63; 0.78) | 0.03 (-0.31; 0.37) | -0.01 (-0.88; 0.86) | **AE** | NA | 0.24 (-0.49; 0.97) | -0.27 (-1.15; 0.60) | NA | -0.19 (-1.40; 1.03) | NA | 0.51 (-0.15; 1.16) | NA | -0.04 (-0.63; 0.54) | 0.67 (-0.07; 1.42) |
| **1.12 ( 0.20; 2.04)** | 0.42 (-0.12; 0.97) | 0.48 (-0.27; 1.22) | 0.41 (-0.31; 1.13) | 0.27 (-0.26; 0.80) | 0.24 (-0.23; 0.72) | 0.25 (-0.24; 0.74) | 0.25 (-0.32; 0.82) | 0.29 (-0.80; 1.37) | 0.18 (-0.29; 0.65) | 0.10 (-0.43; 0.64) | 0.11 (-0.65; 0.87) | 0.06 (-0.36; 0.49) | 0.02 (-0.88; 0.92) | 0.03 (-0.46; 0.53) | **TC** | NA | NA | NA | NA | **0.66 (-0.40; 1.72)** | 0.30 (-0.75; 1.34) | NA | NA | 0.40 (-0.14; 0.93) |
| **1.12 ( 0.28; 1.96)** | 0.42 (-0.02; 0.86) | 0.48 (-0.20; 1.15) | 0.41 (-0.22; 1.04) | 0.27 (-0.15; 0.70) | 0.24 (-0.08; 0.57) | 0.25 (-0.12; 0.62) | 0.25 (-0.20; 0.69) | 0.29 (-0.75; 1.32) | 0.18 (-0.14; 0.50) | 0.10 (-0.31; 0.52) | 0.11 (-0.58; 0.80) | 0.06 (-0.25; 0.38) | 0.02 (-0.83; 0.88) | 0.03 (-0.31; 0.37) | -0.00 (-0.47; 0.47) | **VR** | 0.10 (-0.38; 0.59) | NA | NA | NA | 0.06 (-0.32; 0.45) | **0.67 ( 0.10; 1.24)** | 0.05 (-0.57; 0.68) | 0.44 (-0.30; 1.17) |
| **1.13 ( 0.35; 1.91)** | 0.44 (-0.02; 0.89) | 0.49 (-0.20; 1.17) | 0.42 (-0.19; 1.04) | 0.29 (-0.15; 0.72) | 0.26 (-0.07; 0.58) | 0.26 (-0.13; 0.66) | 0.26 (-0.20; 0.72) | 0.30 (-0.74; 1.33) | 0.19 (-0.15; 0.53) | 0.12 (-0.29; 0.52) | 0.12 (-0.58; 0.82) | 0.08 (-0.25; 0.40) | 0.04 (-0.83; 0.90) | 0.05 (-0.31; 0.40) | 0.01 (-0.47; 0.50) | 0.01 (-0.29; 0.32) | **TT** | NA | NA | NA | NA | NA | 0.26 (-0.44; 0.97) | **0.95 ( 0.02; 1.87)** |
| **1.25 ( 0.33; 2.17)** | **0.56 ( 0.02; 1.10)** | 0.61 (-0.15; 1.36) | 0.54 (-0.19; 1.27) | 0.41 (-0.12; 0.93) | 0.38 (-0.10; 0.85) | 0.38 (-0.10; 0.87) | 0.38 (-0.20; 0.96) | 0.42 (-0.67; 1.51) | 0.31 (-0.16; 0.79) | 0.23 (-0.30; 0.77) | 0.24 (-0.53; 1.01) | 0.19 (-0.25; 0.64) | 0.15 (-0.76; 1.07) | 0.16 (-0.34; 0.67) | 0.13 (-0.44; 0.70) | 0.13 (-0.34; 0.61) | 0.12 (-0.37; 0.61) | **QIG** | NA | NA | NA | 0.12 (-0.55; 0.80) | NA | 0.36 (-0.13; 0.86) |
| **1.24 ( 0.38; 2.10)** | **0.54 ( 0.10; 0.98)** | 0.59 (-0.08; 1.27) | 0.53 (-0.12; 1.18) | 0.39 (-0.03; 0.81) | **0.36 ( 0.02; 0.70)** | **0.37 ( 0.00; 0.74)** | 0.37 (-0.09; 0.83) | 0.40 (-0.63; 1.44) | 0.30 (-0.05; 0.64) | 0.22 (-0.19; 0.63) | 0.23 (-0.46; 0.92) | 0.18 (-0.13; 0.49) | 0.14 (-0.71; 1.00) | 0.15 (-0.22; 0.52) | 0.12 (-0.35; 0.59) | 0.12 (-0.21; 0.45) | 0.11 (-0.25; 0.46) | -0.01 (-0.48; 0.46) | **RT** | NA | 0.09 (-0.37; 0.54) | -0.05 (-0.58; 0.47) | 0.64 (-0.13; 1.41) | **0.46 ( 0.06; 0.86)** |
| **1.26 ( 0.34; 2.18)** | **0.56 ( 0.01; 1.12)** | 0.61 (-0.13; 1.36) | 0.55 (-0.18; 1.28) | 0.41 (-0.13; 0.95) | 0.38 (-0.09; 0.85) | 0.39 (-0.11; 0.89) | 0.39 (-0.18; 0.96) | 0.42 (-0.66; 1.51) | 0.32 (-0.16; 0.80) | 0.24 (-0.30; 0.78) | 0.25 (-0.51; 1.01) | 0.20 (-0.25; 0.65) | 0.16 (-0.64; 0.97) | 0.17 (-0.33; 0.67) | 0.14 (-0.40; 0.68) | 0.14 (-0.33; 0.61) | 0.13 (-0.36; 0.62) | 0.01 (-0.57; 0.58) | 0.02 (-0.45; 0.50) | **YOG** | 0.16 (-0.50; 0.82) | NA | NA | 0.29 (-0.25; 0.82) |
| **1.32 ( 0.48; 2.16)** | **0.63 ( 0.21; 1.04)** | **0.68 ( 0.04; 1.31)** | 0.61 (-0.01; 1.24) | **0.47 ( 0.07; 0.88)** | **0.45 ( 0.19; 0.71)** | **0.45 ( 0.11; 0.80)** | **0.45 ( 0.04; 0.86)** | 0.49 (-0.54; 1.51) | **0.38 ( 0.07; 0.69)** | 0.30 (-0.10; 0.70) | 0.31 (-0.35; 0.97) | 0.26 (-0.02; 0.55) | 0.22 (-0.61; 1.06) | 0.23 (-0.09; 0.56) | 0.20 (-0.24; 0.65) | 0.20 (-0.06; 0.47) | 0.19 (-0.13; 0.50) | 0.07 (-0.39; 0.53) | 0.08 (-0.20; 0.37) | 0.06 (-0.37; 0.49) | **BGT** | NA | NA | 0.37 (-0.12; 0.85) |
| **1.48 ( 0.62; 2.34)** | **0.79 ( 0.40; 1.17)** | **0.84 ( 0.16; 1.52)** | **0.77 ( 0.13; 1.42)** | **0.63 ( 0.24; 1.03)** | **0.61 ( 0.27; 0.94)** | **0.61 ( 0.31; 0.91)** | **0.61 ( 0.14; 1.08)** | 0.65 (-0.39; 1.69) | **0.54 ( 0.20; 0.89)** | **0.46 ( 0.05; 0.88)** | 0.47 (-0.22; 1.16) | **0.42 ( 0.14; 0.70)** | 0.38 (-0.47; 1.24) | **0.39 ( 0.02; 0.77)** | 0.36 (-0.11; 0.83) | **0.36 ( 0.04; 0.68)** | 0.35 (-0.01; 0.70) | 0.23 (-0.20; 0.66) | 0.24 (-0.07; 0.56) | 0.22 (-0.25; 0.70) | 0.16 (-0.14; 0.46) | **CPP** | NA | 0.05 (-0.80; 0.90) |
| **1.53 ( 0.68; 2.39)** | **0.84 ( 0.37; 1.31)** | **0.89 ( 0.19; 1.58)** | **0.82 ( 0.18; 1.47)** | **0.69 ( 0.23; 1.14)** | **0.66 ( 0.29; 1.02)** | **0.66 ( 0.25; 1.08)** | **0.66 ( 0.22; 1.10)** | 0.70 (-0.33; 1.73) | **0.59 ( 0.27; 0.91)** | **0.52 ( 0.10; 0.93)** | 0.52 (-0.19; 1.23) | **0.48 ( 0.12; 0.83)** | 0.44 (-0.43; 1.31) | **0.45 ( 0.10; 0.79)** | 0.41 (-0.09; 0.91) | **0.41 ( 0.08; 0.75)** | **0.40 ( 0.05; 0.75)** | 0.28 (-0.22; 0.79) | 0.29 (-0.06; 0.65) | 0.27 (-0.23; 0.78) | 0.21 (-0.12; 0.54) | 0.05 (-0.32; 0.42) | **STR** | NA |
| **1.56 ( 0.72; 2.39)** | **0.86 ( 0.48; 1.25)** | **0.91 ( 0.28; 1.55)** | **0.85 ( 0.24; 1.46)** | **0.71 ( 0.36; 1.06)** | **0.68 ( 0.41; 0.95)** | **0.69 ( 0.38; 1.00)** | **0.69 ( 0.27; 1.10)** | 0.72 (-0.29; 1.74) | **0.62 ( 0.36; 0.87)** | **0.54 ( 0.19; 0.90)** | 0.55 (-0.11; 1.20) | **0.50 ( 0.30; 0.70)** | 0.46 (-0.36; 1.28) | **0.47 ( 0.16; 0.78)** | **0.44 ( 0.03; 0.84)** | **0.44 ( 0.17; 0.71)** | **0.42 ( 0.14; 0.71)** | 0.31 (-0.10; 0.71) | **0.32 ( 0.06; 0.58)** | 0.30 (-0.11; 0.71) | **0.24 ( 0.01; 0.47)** | 0.08 (-0.18; 0.34) | 0.02 (-0.29; 0.33) | **CON** |

## Table 6.2: League Table of Static Steady-State Balance

| **AQE** | NA | -0.28 (-1.27; 0.71) | NA | 0.31 (-0.72; 1.33) | NA | NA | NA | NA | NA | NA | 0.33 (-0.85; 1.50) | NA | NA | NA | NA | NA | NA | NA | **1.79 ( 0.61; 2.96)** | NA |
| --- | --- | --- | --- | --- | --- | --- | --- | --- | --- | --- | --- | --- | --- | --- | --- | --- | --- | --- | --- | --- |
| 0.10 (-0.58; 0.79) | **VR** | 1.02 (-0.12; 2.16) | NA | 0.12 (-0.65; 0.90) | NA | NA | 0.07 (-0.67; 0.81) | NA | NA | NA | 0.38 (-0.57; 1.33) | NA | NA | 0.37 (-0.60; 1.34) | NA | **0.95 ( 0.33; 1.56)** | NA | NA | NA | NA |
| 0.13 (-0.47; 0.73) | 0.03 (-0.43; 0.49) | **MC** | NA | 0.41 (-0.89; 1.71) | NA | NA | -0.75 (-1.88; 0.38) | NA | NA | NA | NA | NA | NA | NA | NA | **0.85 ( 0.56; 1.13)** | 0.75 (-0.26; 1.75) | 0.85 (-0.28; 1.99) | **1.11 ( 0.31; 1.91)** | NA |
| 0.00 (-1.36; 1.37) | -0.10 (-1.44; 1.24) | -0.13 (-1.47; 1.21) | **NW** | NA | NA | NA | NA | NA | NA | NA | 0.53 (-0.59; 1.64) | NA | NA | NA | NA | NA | NA | NA | NA | NA |
| 0.24 (-0.39; 0.87) | 0.14 (-0.34; 0.61) | 0.11 (-0.32; 0.54) | 0.23 (-1.12; 1.59) | **BGT** | NA | NA | 0.77 (-0.54; 2.09) | 0.18 (-0.58; 0.94) | NA | 0.48 (-0.68; 1.64) | NA | NA | NA | **1.03 ( 0.02; 2.05)** | NA | 0.39 (-0.30; 1.09) | NA | NA | NA | NA |
| 0.36 (-0.57; 1.29) | 0.26 (-0.56; 1.08) | 0.23 (-0.52; 0.98) | 0.36 (-1.14; 1.86) | 0.12 (-0.68; 0.93) | **QIG** | NA | NA | NA | NA | NA | NA | NA | NA | NA | NA | 0.57 (-0.13; 1.28) | NA | NA | NA | NA |
| 0.38 (-0.45; 1.20) | 0.27 (-0.41; 0.96) | 0.25 (-0.38; 0.87) | 0.37 (-1.07; 1.81) | 0.14 (-0.54; 0.81) | 0.01 (-0.90; 0.93) | **TC** | NA | NA | NA | NA | NA | NA | NA | 0.33 (-0.52; 1.17) | NA | 0.50 (-0.31; 1.32) | NA | 0.70 (-0.14; 1.55) | NA | NA |
| 0.42 (-0.29; 1.13) | 0.32 (-0.18; 0.81) | 0.29 (-0.17; 0.75) | 0.42 (-0.96; 1.79) | 0.18 (-0.33; 0.69) | 0.06 (-0.76; 0.88) | 0.05 (-0.62; 0.71) | **DT** | NA | NA | NA | NA | NA | NA | NA | NA | 0.36 (-0.37; 1.10) | 0.00 (-1.00; 1.00) | **0.74 ( 0.17; 1.31)** | NA | 1.08 (-0.13; 2.29) |
| 0.46 (-0.28; 1.19) | 0.35 (-0.24; 0.95) | 0.33 (-0.19; 0.85) | 0.45 (-0.95; 1.85) | 0.22 (-0.30; 0.73) | 0.09 (-0.75; 0.94) | 0.08 (-0.66; 0.82) | 0.04 (-0.56; 0.63) | **ECA** | NA | -0.11 (-1.27; 1.04) | NA | NA | NA | NA | NA | 0.18 (-0.50; 0.86) | **1.00 ( 0.10; 1.89)** | NA | NA | NA |
| 0.48 (-0.48; 1.45) | 0.38 (-0.48; 1.24) | 0.35 (-0.44; 1.15) | 0.48 (-1.05; 2.01) | 0.25 (-0.60; 1.09) | 0.12 (-0.91; 1.15) | 0.11 (-0.84; 1.06) | 0.06 (-0.80; 0.93) | 0.03 (-0.86; 0.92) | **YOG** | NA | NA | NA | NA | NA | 0.27 (-0.81; 1.35) | 0.47 (-0.32; 1.25) | NA | NA | NA | NA |
| 0.54 (-0.43; 1.50) | 0.44 (-0.43; 1.30) | 0.41 (-0.40; 1.22) | 0.53 (-1.00; 2.06) | 0.30 (-0.50; 1.10) | 0.18 (-0.88; 1.23) | 0.16 (-0.80; 1.13) | 0.12 (-0.75; 0.99) | 0.08 (-0.74; 0.90) | 0.05 (-1.03; 1.14) | **ICA** | NA | NA | NA | NA | NA | 0.39 (-0.77; 1.54) | NA | NA | NA | NA |
| 0.53 (-0.26; 1.32) | 0.43 (-0.32; 1.17) | 0.40 (-0.34; 1.14) | 0.53 (-0.59; 1.64) | 0.29 (-0.48; 1.06) | 0.17 (-0.84; 1.17) | 0.15 (-0.76; 1.07) | 0.11 (-0.70; 0.91) | 0.07 (-0.77; 0.92) | 0.05 (-1.00; 1.09) | -0.01 (-1.06; 1.04) | **AE** | NA | NA | NA | NA | 0.16 (-0.79; 1.11) | NA | NA | NA | NA |
| 0.63 (-0.75; 2.00) | 0.53 (-0.84; 1.89) | 0.50 (-0.81; 1.80) | 0.62 (-1.22; 2.47) | 0.39 (-0.96; 1.74) | 0.26 (-1.22; 1.75) | 0.25 (-1.18; 1.68) | 0.21 (-1.16; 1.57) | 0.17 (-1.21; 1.56) | 0.14 (-1.37; 1.65) | 0.09 (-1.43; 1.61) | 0.10 (-1.37; 1.57) | **RA** | NA | NA | NA | NA | NA | NA | 0.47 (-0.71; 1.65) | NA |
| 0.70 (-0.53; 1.92) | 0.59 (-0.62; 1.81) | 0.57 (-0.58; 1.72) | 0.69 (-1.05; 2.43) | 0.46 (-0.74; 1.66) | 0.33 (-1.02; 1.68) | 0.32 (-0.97; 1.61) | 0.28 (-0.94; 1.49) | 0.24 (-1.00; 1.48) | 0.21 (-1.17; 1.59) | 0.16 (-1.23; 1.54) | 0.17 (-1.17; 1.50) | 0.07 (-1.48; 1.62) | **MD** | NA | NA | NA | NA | NA | 0.40 (-0.60; 1.41) | NA |
| 0.67 (-0.02; 1.37) | **0.57 ( 0.08; 1.07)** | **0.55 ( 0.10; 0.99)** | 0.67 (-0.70; 2.04) | 0.44 (-0.05; 0.92) | 0.31 (-0.49; 1.12) | 0.30 (-0.30; 0.90) | 0.25 (-0.25; 0.76) | 0.22 (-0.37; 0.81) | 0.19 (-0.66; 1.04) | 0.14 (-0.72; 0.99) | 0.15 (-0.65; 0.94) | 0.05 (-1.31; 1.41) | -0.02 (-1.23; 1.19) | **RT** | NA | 0.44 (-0.09; 0.96) | NA | 0.24 (-0.40; 0.88) | NA | NA |
| 0.70 (-0.24; 1.63) | 0.59 (-0.23; 1.42) | 0.57 (-0.19; 1.33) | 0.69 (-0.82; 2.20) | 0.46 (-0.35; 1.27) | 0.33 (-0.67; 1.34) | 0.32 (-0.60; 1.24) | 0.28 (-0.56; 1.11) | 0.24 (-0.62; 1.10) | 0.21 (-0.66; 1.08) | 0.16 (-0.90; 1.22) | 0.17 (-0.85; 1.18) | 0.07 (-1.42; 1.56) | 0.00 (-1.36; 1.36) | 0.02 (-0.79; 0.83) | **PT** | 0.28 (-0.46; 1.02) | NA | NA | NA | NA |
| **0.94 ( 0.33; 1.54)** | **0.83 ( 0.41; 1.25)** | **0.81 ( 0.55; 1.06)** | 0.93 (-0.40; 2.26) | **0.70 ( 0.31; 1.08)** | 0.57 (-0.13; 1.28) | 0.56 (-0.02; 1.14) | **0.51 ( 0.09; 0.94)** | **0.48 ( 0.01; 0.95)** | 0.45 (-0.30; 1.20) | 0.40 (-0.38; 1.18) | 0.41 (-0.32; 1.13) | 0.31 (-1.00; 1.62) | 0.24 (-0.91; 1.39) | 0.26 (-0.13; 0.65) | 0.24 (-0.48; 0.95) | **CON** | 0.15 (-0.96; 1.26) | NA | -0.45 (-1.38; 0.47) | NA |
| **0.98 ( 0.20; 1.77)** | **0.88 ( 0.23; 1.53)** | **0.85 ( 0.30; 1.41)** | 0.98 (-0.44; 2.40) | **0.74 ( 0.13; 1.36)** | 0.62 (-0.27; 1.51) | 0.61 (-0.17; 1.38) | 0.56 (-0.04; 1.16) | 0.53 (-0.07; 1.13) | 0.50 (-0.43; 1.42) | 0.44 (-0.47; 1.36) | 0.45 (-0.43; 1.34) | 0.36 (-1.05; 1.76) | 0.29 (-0.98; 1.55) | 0.31 (-0.33; 0.95) | 0.29 (-0.61; 1.18) | 0.05 (-0.49; 0.59) | **TT** | NA | NA | NA |
| **1.01 ( 0.25; 1.76)** | **0.91 ( 0.33; 1.48)** | **0.88 ( 0.35; 1.40)** | 1.00 (-0.40; 2.41) | **0.77 ( 0.19; 1.35)** | 0.64 (-0.21; 1.50) | **0.63 ( 0.00; 1.27)** | **0.59 ( 0.11; 1.06)** | 0.55 (-0.11; 1.21) | 0.52 (-0.38; 1.42) | 0.47 (-0.44; 1.38) | 0.48 (-0.37; 1.33) | 0.38 (-1.01; 1.77) | 0.31 (-0.93; 1.56) | 0.33 (-0.16; 0.83) | 0.31 (-0.56; 1.18) | 0.07 (-0.42; 0.56) | 0.03 (-0.66; 0.71) | **STR** | NA | NA |
| **1.10 ( 0.39; 1.81)** | **1.00 ( 0.31; 1.68)** | **0.97 ( 0.41; 1.53)** | 1.09 (-0.33; 2.51) | **0.86 ( 0.20; 1.52)** | 0.74 (-0.17; 1.64) | 0.72 (-0.08; 1.53) | 0.68 (-0.01; 1.37) | 0.64 (-0.08; 1.37) | 0.61 (-0.33; 1.56) | 0.56 (-0.40; 1.52) | 0.57 (-0.31; 1.45) | 0.47 (-0.71; 1.65) | 0.40 (-0.60; 1.41) | 0.42 (-0.25; 1.10) | 0.40 (-0.51; 1.32) | 0.16 (-0.41; 0.73) | 0.12 (-0.65; 0.88) | 0.09 (-0.65; 0.83) | **CPP** | NA |
| **1.51 ( 0.10; 2.91)** | **1.40 ( 0.10; 2.71)** | **1.38 ( 0.08; 2.67)** | 1.50 (-0.33; 3.33) | 1.27 (-0.05; 2.58) | 1.14 (-0.32; 2.61) | 1.13 (-0.25; 2.51) | 1.08 (-0.13; 2.29) | 1.05 (-0.30; 2.40) | 1.02 (-0.47; 2.51) | 0.97 (-0.52; 2.46) | 0.98 (-0.48; 2.43) | 0.88 (-0.95; 2.70) | 0.81 (-0.91; 2.53) | 0.83 (-0.48; 2.14) | 0.81 (-0.66; 2.28) | 0.57 (-0.71; 1.85) | 0.52 (-0.83; 1.87) | 0.50 (-0.80; 1.80) | 0.41 (-0.99; 1.80) | **WBV** |

## Table 6.3: League Table of Dynamic Steady-State Balance

| **BWS** | NA | 0.21 (-0.86; 1.28) | NA | NA | NA | NA | NA | NA | NA | 0.21 (-0.86; 1.28) | 0.37 (-0.71; 1.45) | **1.61 ( 0.96; 2.26)** | NA | NA | NA | NA | NA | NA | NA | NA | **2.30 ( 1.61; 2.98)** | NA | NA |
| --- | --- | --- | --- | --- | --- | --- | --- | --- | --- | --- | --- | --- | --- | --- | --- | --- | --- | --- | --- | --- | --- | --- | --- |
| **0.93 ( 0.39; 1.46)** | **VR** | 0.40 (-0.11; 0.90) | 0.55 (-0.37; 1.48) | NA | NA | NA | 0.31 (-0.56; 1.18) | 0.20 (-0.81; 1.20) | NA | NA | 0.22 (-0.28; 0.72) | NA | NA | -0.13 (-0.97; 0.71) | NA | NA | NA | NA | NA | NA | 0.25 (-0.39; 0.88) | NA | NA |
| **1.00 ( 0.51; 1.49)** | 0.07 (-0.24; 0.39) | **TT** | 0.08 (-0.55; 0.70) | NA | NA | NA | 0.26 (-0.69; 1.21) | -0.20 (-1.07; 0.67) | -0.64 (-1.73; 0.45) | 0.00 (-1.07; 1.07) | 0.57 (-0.44; 1.57) | 0.27 (-0.20; 0.74) | NA | 0.20 (-0.40; 0.80) | -0.16 (-0.98; 0.66) | NA | 0.28 (-0.30; 0.86) | NA | NA | NA | 0.43 (-0.03; 0.90) | **1.68 ( 0.88; 2.49)** | **1.58 ( 0.74; 2.41)** |
| **1.02 ( 0.52; 1.53)** | 0.10 (-0.24; 0.43) | 0.02 (-0.25; 0.30) | **DT** | -0.14 (-1.19; 0.91) | 0.10 (-0.69; 0.88) | NA | NA | NA | NA | 0.43 (-0.51; 1.38) | **0.49 ( 0.06; 0.92)** | NA | NA | NA | NA | NA | -0.19 (-1.26; 0.87) | NA | NA | NA | **0.38 ( 0.09; 0.68)** | NA | NA |
| 0.88 (-0.28; 2.05) | -0.05 (-1.15; 1.06) | -0.12 (-1.20; 0.97) | -0.14 (-1.19; 0.91) | **WBV** | NA | NA | NA | NA | NA | NA | NA | NA | NA | NA | NA | NA | NA | NA | NA | NA | NA | NA | NA |
| **1.03 ( 0.44; 1.63)** | 0.11 (-0.37; 0.59) | 0.04 (-0.40; 0.47) | 0.01 (-0.40; 0.43) | 0.15 (-0.98; 1.28) | **MD** | NA | NA | NA | NA | 0.30 (-0.19; 0.79) | NA | -0.10 (-1.13; 0.94) | NA | NA | NA | NA | NA | NA | NA | NA | 0.00 (-1.07; 1.07) | NA | NA |
| **1.06 ( 0.06; 2.07)** | 0.14 (-0.81; 1.08) | 0.06 (-0.85; 0.98) | 0.04 (-0.88; 0.96) | 0.18 (-1.21; 1.58) | 0.03 (-0.92; 0.98) | **AQE** | NA | NA | NA | 0.08 (-0.78; 0.95) | NA | NA | NA | NA | NA | NA | NA | NA | NA | NA | NA | NA | NA |
| **1.12 ( 0.58; 1.67)** | 0.20 (-0.20; 0.59) | 0.12 (-0.22; 0.47) | 0.10 (-0.27; 0.47) | 0.24 (-0.87; 1.36) | 0.09 (-0.40; 0.58) | 0.06 (-0.89; 1.01) | **AE** | -0.04 (-0.97; 0.89) | NA | NA | 0.26 (-0.46; 0.98) | 0.34 (-0.60; 1.28) | NA | 0.10 (-0.65; 0.85) | NA | NA | -0.70 (-1.58; 0.18) | NA | NA | NA | **0.50 ( 0.00; 1.00)** | NA | **0.89 ( 0.01; 1.77)** |
| **1.13 ( 0.62; 1.64)** | 0.20 (-0.16; 0.56) | 0.13 (-0.17; 0.42) | 0.10 (-0.21; 0.41) | 0.24 (-0.85; 1.34) | 0.09 (-0.35; 0.53) | 0.06 (-0.86; 0.98) | 0.00 (-0.36; 0.37) | **MC** | 0.47 (-0.40; 1.35) | 0.60 (-0.28; 1.48) | NA | -0.41 (-1.37; 0.56) | 0.01 (-0.85; 0.88) | NA | NA | NA | NA | NA | NA | NA | **0.34 ( 0.07; 0.60)** | NA | NA |
| **1.13 ( 0.35; 1.91)** | 0.20 (-0.49; 0.89) | 0.13 (-0.51; 0.77) | 0.11 (-0.56; 0.77) | 0.25 (-0.99; 1.49) | 0.10 (-0.64; 0.83) | 0.07 (-1.03; 1.16) | 0.01 (-0.69; 0.70) | 0.00 (-0.64; 0.65) | **NW** | NA | NA | NA | NA | NA | NA | NA | NA | NA | NA | NA | 0.13 (-0.75; 1.00) | NA | NA |
| **1.15 ( 0.65; 1.65)** | 0.22 (-0.15; 0.59) | 0.15 (-0.15; 0.45) | 0.12 (-0.18; 0.43) | 0.27 (-0.83; 1.36) | 0.11 (-0.27; 0.49) | 0.08 (-0.78; 0.95) | 0.02 (-0.36; 0.40) | 0.02 (-0.29; 0.33) | 0.02 (-0.65; 0.69) | **CPP** | NA | NA | NA | 0.15 (-0.23; 0.53) | NA | 0.40 (-0.38; 1.17) | NA | NA | NA | NA | **0.39 ( 0.03; 0.74)** | NA | NA |
| **1.15 ( 0.66; 1.65)** | 0.23 (-0.10; 0.55) | 0.15 (-0.13; 0.44) | 0.13 (-0.14; 0.40) | 0.27 (-0.81; 1.36) | 0.12 (-0.32; 0.56) | 0.09 (-0.84; 1.01) | 0.03 (-0.33; 0.39) | 0.03 (-0.29; 0.35) | 0.02 (-0.65; 0.69) | 0.01 (-0.31; 0.33) | **BGT** | -0.18 (-0.74; 0.38) | -0.24 (-1.34; 0.87) | 0.31 (-0.26; 0.87) | NA | NA | **1.00 ( 0.09; 1.91)** | NA | 0.01 (-1.09; 1.12) | 0.10 (-0.95; 1.15) | 0.42 (-0.20; 1.04) | NA | NA |
| **1.16 ( 0.68; 1.63)** | 0.23 (-0.12; 0.58) | 0.16 (-0.10; 0.42) | 0.13 (-0.16; 0.43) | 0.28 (-0.82; 1.37) | 0.12 (-0.30; 0.55) | 0.09 (-0.83; 1.01) | 0.03 (-0.33; 0.39) | 0.03 (-0.27; 0.33) | 0.03 (-0.63; 0.69) | 0.01 (-0.30; 0.32) | 0.00 (-0.28; 0.29) | **ECA** | NA | 0.06 (-0.51; 0.62) | NA | NA | NA | NA | NA | 0.13 (-0.54; 0.81) | **0.43 ( 0.14; 0.72)** | NA | NA |
| **1.17 ( 0.60; 1.75)** | 0.24 (-0.20; 0.69) | 0.17 (-0.22; 0.57) | 0.15 (-0.26; 0.55) | 0.29 (-0.83; 1.42) | 0.14 (-0.38; 0.65) | 0.11 (-0.85; 1.07) | 0.05 (-0.41; 0.50) | 0.05 (-0.35; 0.44) | 0.04 (-0.67; 0.76) | 0.03 (-0.38; 0.43) | 0.02 (-0.39; 0.42) | 0.02 (-0.39; 0.42) | **TC** | 0.03 (-0.67; 0.72) | NA | -0.08 (-0.81; 0.66) | NA | NA | 0.25 (-0.86; 1.36) | NA | 0.38 (-0.26; 1.02) | NA | 0.67 (-0.03; 1.37) |
| **1.18 ( 0.69; 1.67)** | 0.25 (-0.07; 0.58) | 0.18 (-0.07; 0.43) | 0.16 (-0.12; 0.43) | 0.30 (-0.79; 1.38) | 0.15 (-0.27; 0.56) | 0.12 (-0.79; 1.02) | 0.06 (-0.28; 0.39) | 0.05 (-0.23; 0.34) | 0.05 (-0.60; 0.71) | 0.03 (-0.23; 0.29) | 0.03 (-0.25; 0.30) | 0.02 (-0.24; 0.29) | 0.01 (-0.36; 0.38) | **RT** | NA | NA | NA | NA | NA | NA | **0.51 ( 0.24; 0.78)** | 0.00 (-1.09; 1.09) | **0.60 ( 0.11; 1.09)** |
| **1.21 ( 0.55; 1.87)** | 0.28 (-0.27; 0.83) | 0.21 (-0.28; 0.70) | 0.19 (-0.33; 0.71) | 0.33 (-0.84; 1.50) | 0.18 (-0.44; 0.79) | 0.15 (-0.87; 1.16) | 0.09 (-0.48; 0.65) | 0.09 (-0.44; 0.61) | 0.08 (-0.70; 0.87) | 0.06 (-0.47; 0.59) | 0.06 (-0.47; 0.58) | 0.05 (-0.46; 0.57) | 0.04 (-0.55; 0.62) | 0.03 (-0.47; 0.53) | **DAN** | NA | NA | NA | NA | NA | 0.24 (-0.40; 0.89) | 0.06 (-1.09; 1.21) | NA |
| **1.23 ( 0.59; 1.88)** | 0.30 (-0.24; 0.84) | 0.23 (-0.26; 0.73) | 0.21 (-0.29; 0.71) | 0.35 (-0.81; 1.51) | 0.20 (-0.38; 0.78) | 0.17 (-0.82; 1.16) | 0.11 (-0.44; 0.65) | 0.11 (-0.39; 0.61) | 0.10 (-0.67; 0.88) | 0.08 (-0.39; 0.56) | 0.08 (-0.43; 0.59) | 0.08 (-0.42; 0.58) | 0.06 (-0.43; 0.55) | 0.05 (-0.43; 0.53) | 0.02 (-0.64; 0.68) | **QIG** | NA | NA | NA | NA | 0.44 (-0.30; 1.18) | NA | NA |
| **1.22 ( 0.63; 1.82)** | 0.30 (-0.16; 0.76) | 0.22 (-0.16; 0.61) | 0.20 (-0.22; 0.62) | 0.34 (-0.79; 1.47) | 0.19 (-0.35; 0.73) | 0.16 (-0.81; 1.13) | 0.10 (-0.35; 0.55) | 0.10 (-0.34; 0.54) | 0.09 (-0.64; 0.83) | 0.08 (-0.37; 0.52) | 0.07 (-0.35; 0.49) | 0.07 (-0.36; 0.49) | 0.05 (-0.45; 0.56) | 0.04 (-0.36; 0.45) | 0.01 (-0.59; 0.61) | -0.01 (-0.60; 0.59) | **RA** | NA | NA | NA | NA | NA | **0.95 ( 0.30; 1.61)** |
| **1.26 ( 0.48; 2.04)** | 0.33 (-0.36; 1.03) | 0.26 (-0.40; 0.93) | 0.24 (-0.43; 0.90) | 0.38 (-0.86; 1.62) | 0.23 (-0.52; 0.97) | 0.20 (-0.90; 1.29) | 0.14 (-0.57; 0.84) | 0.14 (-0.53; 0.80) | 0.13 (-0.76; 1.02) | 0.11 (-0.56; 0.79) | 0.11 (-0.56; 0.78) | 0.10 (-0.56; 0.77) | 0.09 (-0.62; 0.80) | 0.08 (-0.58; 0.74) | 0.05 (-0.74; 0.84) | 0.03 (-0.75; 0.80) | 0.04 (-0.70; 0.78) | **PT** | -0.14 (-1.11; 0.83) | NA | 0.44 (-0.23; 1.11) | NA | NA |
| **1.26 ( 0.54; 1.99)** | 0.34 (-0.29; 0.97) | 0.27 (-0.33; 0.86) | 0.24 (-0.36; 0.84) | 0.38 (-0.83; 1.59) | 0.23 (-0.45; 0.91) | 0.20 (-0.86; 1.26) | 0.14 (-0.50; 0.78) | 0.14 (-0.46; 0.74) | 0.13 (-0.71; 0.98) | 0.12 (-0.49; 0.72) | 0.11 (-0.48; 0.71) | 0.11 (-0.49; 0.71) | 0.09 (-0.54; 0.72) | 0.08 (-0.50; 0.67) | 0.05 (-0.68; 0.79) | 0.03 (-0.68; 0.75) | 0.04 (-0.64; 0.72) | 0.00 (-0.72; 0.73) | **YOG** | NA | 0.39 (-0.33; 1.11) | NA | NA |
| **1.28 ( 0.60; 1.97)** | 0.35 (-0.24; 0.95) | 0.28 (-0.27; 0.84) | 0.26 (-0.30; 0.82) | 0.40 (-0.79; 1.59) | 0.25 (-0.40; 0.89) | 0.22 (-0.82; 1.25) | 0.16 (-0.44; 0.76) | 0.16 (-0.41; 0.72) | 0.15 (-0.67; 0.97) | 0.13 (-0.44; 0.70) | 0.13 (-0.42; 0.68) | 0.12 (-0.40; 0.65) | 0.11 (-0.52; 0.73) | 0.10 (-0.45; 0.65) | 0.07 (-0.63; 0.78) | 0.05 (-0.64; 0.74) | 0.06 (-0.59; 0.70) | 0.02 (-0.80; 0.84) | 0.02 (-0.75; 0.78) | **ICA** | 0.29 (-0.42; 1.00) | NA | NA |
| **1.53 ( 1.07; 2.00)** | **0.60 ( 0.30; 0.91)** | **0.53 ( 0.32; 0.75)** | **0.51 ( 0.29; 0.73)** | 0.65 (-0.42; 1.72) | **0.50 ( 0.10; 0.89)** | 0.47 (-0.43; 1.37) | **0.41 ( 0.09; 0.72)** | **0.41 ( 0.18; 0.63)** | 0.40 (-0.23; 1.04) | **0.38 ( 0.14; 0.62)** | **0.38 ( 0.13; 0.62)** | **0.38 ( 0.16; 0.60)** | **0.36 ( 0.01; 0.71)** | **0.35 ( 0.16; 0.55)** | 0.32 (-0.16; 0.80) | 0.30 (-0.16; 0.76) | 0.31 (-0.09; 0.70) | 0.27 (-0.36; 0.90) | 0.27 (-0.30; 0.83) | 0.25 (-0.27; 0.78) | **CON** | -0.19 (-1.12; 0.74) | NA |
| **1.74 ( 1.09; 2.40)** | **0.81 ( 0.27; 1.36)** | **0.74 ( 0.26; 1.23)** | **0.72 ( 0.21; 1.23)** | 0.86 (-0.31; 2.03) | **0.71 ( 0.10; 1.31)** | 0.68 (-0.33; 1.69) | **0.62 ( 0.07; 1.17)** | **0.62 ( 0.10; 1.14)** | 0.61 (-0.17; 1.39) | **0.60 ( 0.08; 1.11)** | **0.59 ( 0.07; 1.11)** | **0.59 ( 0.08; 1.10)** | **0.57 ( 0.00; 1.14)** | **0.56 ( 0.08; 1.05)** | 0.53 (-0.08; 1.14) | 0.51 (-0.14; 1.16) | 0.52 (-0.07; 1.10) | 0.48 (-0.31; 1.27) | 0.48 (-0.25; 1.21) | 0.46 (-0.24; 1.16) | 0.21 (-0.26; 0.69) | **TAN** | -0.11 (-0.89; 0.68) |
| **1.91 ( 1.34; 2.49)** | **0.98 ( 0.54; 1.43)** | **0.91 ( 0.53; 1.29)** | **0.89 ( 0.48; 1.30)** | 1.03 (-0.10; 2.16) | **0.88 ( 0.36; 1.40)** | 0.85 (-0.11; 1.81) | **0.79 ( 0.35; 1.22)** | **0.79 ( 0.37; 1.20)** | **0.78 ( 0.06; 1.50)** | **0.76 ( 0.35; 1.18)** | **0.76 ( 0.35; 1.17)** | **0.76 ( 0.35; 1.16)** | **0.74 ( 0.29; 1.19)** | **0.73 ( 0.38; 1.09)** | **0.70 ( 0.12; 1.28)** | **0.68 ( 0.12; 1.24)** | **0.69 ( 0.24; 1.13)** | 0.65 (-0.07; 1.38) | 0.65 (-0.01; 1.31) | **0.63 ( 0.00; 1.26)** | **0.38 ( 0.02; 0.74)** | 0.17 (-0.36; 0.70) | **STR** |

## Table 6.4: League Table of Proactive Balance

| **PIL** | NA | NA | 0.62 (-0.57; 1.80) | NA | NA | NA | NA | NA | NA | NA | NA | NA | NA | NA | NA | NA | NA | NA | NA | NA | NA | **1.47 ( 0.31; 2.62)** |
| --- | --- | --- | --- | --- | --- | --- | --- | --- | --- | --- | --- | --- | --- | --- | --- | --- | --- | --- | --- | --- | --- | --- |
| 0.54 (-0.36; 1.44) | **AQE** | NA | 0.19 (-0.37; 0.76) | NA | NA | NA | NA | NA | 0.10 (-0.88; 1.08) | NA | NA | NA | 0.28 (-0.78; 1.34) | NA | NA | NA | -0.04 (-1.19; 1.11) | NA | **0.76 ( 0.14; 1.39)** | NA | NA | 0.75 (-0.40; 1.91) |
| 0.60 (-0.38; 1.57) | 0.06 (-0.56; 0.67) | **QIG** | NA | NA | NA | NA | NA | NA | NA | NA | NA | NA | NA | NA | NA | NA | NA | NA | 0.04 (-0.90; 0.99) | NA | NA | **1.01 ( 0.42; 1.60)** |
| 0.66 (-0.17; 1.50) | 0.12 (-0.25; 0.50) | 0.07 (-0.48; 0.61) | **MC** | NA | 0.08 (-0.94; 1.10) | NA | NA | NA | NA | -0.39 (-1.49; 0.72) | NA | NA | 0.19 (-0.36; 0.73) | -1.09 (-2.27; 0.08) | NA | NA | 0.24 (-0.25; 0.73) | NA | -1.00 (-2.19; 0.18) | NA | 0.79 (-0.25; 1.83) | **0.97 ( 0.71; 1.23)** |
| 0.66 (-0.32; 1.63) | 0.12 (-0.52; 0.75) | 0.06 (-0.66; 0.78) | -0.01 (-0.56; 0.55) | **TAN** | NA | NA | NA | 0.77 (-0.47; 2.02) | NA | NA | NA | NA | NA | NA | NA | NA | NA | 0.65 (-0.25; 1.56) | NA | NA | NA | 0.47 (-0.20; 1.15) |
| 0.69 (-0.22; 1.60) | 0.15 (-0.37; 0.66) | 0.09 (-0.53; 0.72) | 0.03 (-0.38; 0.43) | 0.03 (-0.60; 0.66) | **TC** | NA | NA | **0.64 ( 0.03; 1.25)** | NA | NA | NA | 0.20 (-1.03; 1.44) | 0.21 (-1.03; 1.44) | NA | NA | NA | NA | NA | NA | 0.74 (-0.14; 1.63) | NA | 0.27 (-0.33; 0.87) |
| 0.77 (-0.17; 1.71) | 0.23 (-0.32; 0.79) | 0.17 (-0.49; 0.84) | 0.11 (-0.36; 0.58) | 0.12 (-0.56; 0.79) | 0.08 (-0.47; 0.64) | **DT** | NA | NA | NA | NA | 0.08 (-1.10; 1.26) | NA | 0.46 (-0.33; 1.26) | -0.03 (-1.09; 1.03) | 0.08 (-0.87; 1.02) | 0.77 (-0.28; 1.82) | NA | NA | NA | 0.50 (-0.45; 1.45) | NA | 0.10 (-0.94; 1.14) |
| 0.77 (-0.27; 1.81) | 0.23 (-0.48; 0.95) | 0.18 (-0.62; 0.97) | 0.11 (-0.55; 0.77) | 0.12 (-0.69; 0.93) | 0.08 (-0.64; 0.81) | 0.00 (-0.76; 0.76) | **PT** | NA | NA | NA | NA | 0.44 (-0.68; 1.55) | NA | NA | NA | NA | NA | NA | 0.37 (-0.84; 1.59) | NA | NA | 0.62 (-0.16; 1.39) |
| 0.81 (-0.05; 1.66) | 0.27 (-0.14; 0.68) | 0.21 (-0.33; 0.75) | 0.14 (-0.14; 0.42) | 0.15 (-0.39; 0.69) | 0.12 (-0.27; 0.50) | 0.03 (-0.42; 0.49) | 0.03 (-0.62; 0.68) | **RT** | NA | 0.14 (-0.74; 1.02) | NA | -0.10 (-0.98; 0.78) | -0.01 (-0.65; 0.63) | NA | NA | -0.19 (-0.90; 0.53) | -0.09 (-0.94; 0.76) | NA | 0.43 (-0.17; 1.02) | **0.79 ( 0.18; 1.39)** | NA | **0.76 ( 0.48; 1.03)** |
| 0.77 (-0.38; 1.91) | 0.23 (-0.54; 1.00) | 0.17 (-0.76; 1.11) | 0.11 (-0.70; 0.91) | 0.11 (-0.83; 1.06) | 0.08 (-0.79; 0.95) | -0.00 (-0.90; 0.89) | -0.00 (-1.01; 1.00) | -0.04 (-0.85; 0.78) | **MD** | NA | NA | NA | NA | NA | NA | NA | NA | NA | NA | NA | NA | 0.46 (-0.75; 1.66) |
| 0.83 (-0.05; 1.70) | 0.29 (-0.16; 0.74) | 0.23 (-0.35; 0.81) | 0.17 (-0.17; 0.50) | 0.17 (-0.42; 0.76) | 0.14 (-0.32; 0.60) | 0.06 (-0.43; 0.55) | 0.06 (-0.63; 0.74) | 0.02 (-0.31; 0.35) | 0.06 (-0.78; 0.90) | **ECA** | NA | NA | 0.46 (-0.07; 0.98) | 0.21 (-0.96; 1.37) | NA | 0.31 (-0.34; 0.97) | -0.01 (-0.72; 0.71) | NA | NA | NA | NA | 0.25 (-0.17; 0.66) |
| 0.85 (-0.66; 2.36) | 0.31 (-0.99; 1.61) | 0.25 (-1.10; 1.61) | 0.19 (-1.08; 1.46) | 0.19 (-1.17; 1.55) | 0.16 (-1.14; 1.47) | 0.08 (-1.10; 1.26) | 0.08 (-1.33; 1.48) | 0.04 (-1.22; 1.31) | 0.08 (-1.40; 1.56) | 0.02 (-1.26; 1.30) | **WBV** | NA | NA | NA | NA | NA | NA | NA | NA | NA | NA | NA |
| 0.90 (-0.06; 1.85) | 0.36 (-0.23; 0.95) | 0.30 (-0.39; 0.99) | 0.23 (-0.27; 0.74) | 0.24 (-0.45; 0.93) | 0.21 (-0.36; 0.78) | 0.13 (-0.50; 0.75) | 0.12 (-0.59; 0.84) | 0.09 (-0.39; 0.57) | 0.13 (-0.79; 1.04) | 0.07 (-0.47; 0.60) | 0.05 (-1.29; 1.38) | **YOG** | -0.02 (-0.82; 0.79) | NA | NA | NA | NA | NA | NA | NA | NA | 0.54 (-0.27; 1.36) |
| **0.90 ( 0.03; 1.77)** | 0.36 (-0.06; 0.78) | 0.31 (-0.26; 0.87) | 0.24 (-0.06; 0.54) | 0.25 (-0.33; 0.82) | 0.21 (-0.22; 0.65) | 0.13 (-0.32; 0.58) | 0.13 (-0.54; 0.80) | 0.10 (-0.20; 0.39) | 0.13 (-0.69; 0.96) | 0.07 (-0.25; 0.40) | 0.05 (-1.21; 1.32) | 0.00 (-0.49; 0.50) | **BGT** | 0.00 (-0.83; 0.83) | NA | NA | 0.07 (-0.44; 0.57) | NA | NA | NA | **2.43 ( 1.19; 3.67)** | **0.64 ( 0.00; 1.28)** |
| **0.92 ( 0.01; 1.83)** | 0.38 (-0.12; 0.88) | 0.32 (-0.30; 0.94) | 0.26 (-0.15; 0.66) | 0.26 (-0.37; 0.90) | 0.23 (-0.28; 0.74) | 0.15 (-0.35; 0.65) | 0.15 (-0.57; 0.87) | 0.11 (-0.29; 0.51) | 0.15 (-0.72; 1.02) | 0.09 (-0.33; 0.52) | 0.07 (-1.21; 1.35) | 0.02 (-0.56; 0.60) | 0.02 (-0.38; 0.42) | **VR** | NA | 0.15 (-0.74; 1.04) | NA | NA | 0.10 (-1.00; 1.20) | 0.20 (-0.58; 0.98) | NA | 0.53 (-0.24; 1.31) |
| **1.05 ( 0.05; 2.05)** | 0.51 (-0.13; 1.16) | 0.46 (-0.28; 1.20) | 0.39 (-0.19; 0.97) | 0.40 (-0.36; 1.15) | 0.36 (-0.29; 1.02) | 0.28 (-0.31; 0.87) | 0.28 (-0.55; 1.11) | 0.25 (-0.32; 0.82) | 0.28 (-0.67; 1.24) | 0.22 (-0.37; 0.82) | 0.20 (-1.12; 1.52) | 0.16 (-0.56; 0.87) | 0.15 (-0.44; 0.74) | 0.13 (-0.49; 0.76) | **RA** | -0.29 (-0.98; 0.41) | NA | NA | 0.41 (-0.67; 1.50) | NA | NA | NA |
| **1.03 ( 0.14; 1.92)** | **0.49 ( 0.01; 0.97)** | 0.43 (-0.17; 1.03) | 0.37 (-0.01; 0.74) | 0.37 (-0.23; 0.98) | 0.34 (-0.14; 0.82) | 0.26 (-0.21; 0.73) | 0.26 (-0.45; 0.96) | 0.22 (-0.13; 0.58) | 0.26 (-0.59; 1.11) | 0.20 (-0.17; 0.58) | 0.18 (-1.09; 1.45) | 0.13 (-0.43; 0.69) | 0.13 (-0.25; 0.51) | 0.11 (-0.32; 0.54) | -0.02 (-0.55; 0.51) | **TT** | 0.52 (-0.58; 1.62) | NA | NA | NA | -0.60 (-1.81; 0.61) | 0.28 (-0.39; 0.95) |
| **1.06 ( 0.18; 1.93)** | **0.52 ( 0.08; 0.96)** | 0.46 (-0.12; 1.05) | **0.40 ( 0.08; 0.72)** | 0.40 (-0.19; 0.99) | 0.37 (-0.09; 0.83) | 0.29 (-0.20; 0.78) | 0.29 (-0.40; 0.98) | 0.25 (-0.08; 0.58) | 0.29 (-0.54; 1.12) | 0.23 (-0.13; 0.59) | 0.21 (-1.07; 1.49) | 0.16 (-0.38; 0.70) | 0.16 (-0.18; 0.49) | 0.14 (-0.30; 0.58) | 0.01 (-0.60; 0.61) | 0.03 (-0.37; 0.43) | **AE** | NA | NA | 0.13 (-0.55; 0.81) | NA | -0.09 (-0.71; 0.54) |
| **1.10 ( 0.16; 2.04)** | 0.56 (-0.01; 1.13) | 0.50 (-0.16; 1.17) | 0.44 (-0.05; 0.92) | 0.44 (-0.14; 1.03) | 0.41 (-0.16; 0.99) | 0.33 (-0.29; 0.94) | 0.33 (-0.43; 1.09) | 0.29 (-0.19; 0.77) | 0.33 (-0.57; 1.23) | 0.27 (-0.25; 0.80) | 0.25 (-1.08; 1.58) | 0.20 (-0.44; 0.84) | 0.20 (-0.31; 0.71) | 0.18 (-0.39; 0.75) | 0.05 (-0.66; 0.75) | 0.07 (-0.47; 0.62) | 0.04 (-0.49; 0.57) | **DAN** | 0.31 (-0.76; 1.38) | NA | NA | 0.35 (-0.14; 0.84) |
| **1.10 ( 0.21; 1.99)** | **0.56 ( 0.15; 0.98)** | 0.51 (-0.04; 1.06) | **0.44 ( 0.08; 0.80)** | 0.45 (-0.15; 1.05) | 0.41 (-0.06; 0.89) | 0.33 (-0.19; 0.85) | 0.33 (-0.33; 1.00) | 0.30 (-0.05; 0.64) | 0.33 (-0.50; 1.16) | 0.28 (-0.14; 0.69) | 0.25 (-1.03; 1.54) | 0.21 (-0.35; 0.76) | 0.20 (-0.19; 0.59) | 0.18 (-0.27; 0.64) | 0.05 (-0.54; 0.64) | 0.07 (-0.36; 0.51) | 0.04 (-0.37; 0.46) | 0.00 (-0.52; 0.53) | **CPP** | NA | NA | 0.17 (-0.56; 0.90) |
| **1.35 ( 0.43; 2.27)** | **0.81 ( 0.29; 1.33)** | **0.75 ( 0.12; 1.39)** | **0.69 ( 0.26; 1.11)** | **0.69 ( 0.05; 1.34)** | **0.66 ( 0.17; 1.16)** | **0.58 ( 0.07; 1.09)** | 0.58 (-0.16; 1.31) | 0.54 ( 0.15; 0.94) | 0.58 (-0.30; 1.46) | **0.52 ( 0.07; 0.98)** | 0.50 (-0.78; 1.79) | 0.45 (-0.14; 1.05) | **0.45 ( 0.02; 0.88)** | 0.43 (-0.03; 0.89) | 0.30 (-0.35; 0.94) | 0.32 (-0.15; 0.79) | 0.29 (-0.13; 0.71) | 0.25 (-0.34; 0.84) | 0.25 (-0.24; 0.73) | **STR** | NA | NA |
| **1.49 ( 0.49; 2.49)** | **0.95 ( 0.29; 1.61)** | **0.89 ( 0.14; 1.65)** | **0.83 ( 0.25; 1.41)** | **0.83 ( 0.07; 1.60)** | **0.80 ( 0.13; 1.47)** | **0.72 ( 0.03; 1.41)** | 0.72 (-0.12; 1.56) | **0.68 ( 0.09; 1.28)** | 0.72 (-0.25; 1.69) | **0.66 ( 0.05; 1.28)** | 0.64 (-0.73; 2.01) | 0.59 (-0.13; 1.32) | **0.59 ( 0.00; 1.18)** | 0.57 (-0.09; 1.23) | 0.44 (-0.33; 1.21) | 0.46 (-0.15; 1.07) | 0.43 (-0.19; 1.05) | 0.39 (-0.32; 1.10) | 0.39 (-0.25; 1.03) | 0.14 (-0.54; 0.81) | **NW** | 0.22 (-0.57; 1.01) |
| **1.42 ( 0.59; 2.26)** | **0.89 ( 0.51; 1.26)** | **0.83 ( 0.32; 1.34)** | **0.76 ( 0.55; 0.98)** | **0.77 ( 0.25; 1.28)** | **0.74 ( 0.36; 1.11)** | **0.65 ( 0.21; 1.09)** | **0.65 ( 0.02; 1.28)** | **0.62 ( 0.41; 0.83)** | 0.66 (-0.14; 1.45) | **0.60 ( 0.30; 0.89)** | 0.58 (-0.68; 1.84) | **0.53 ( 0.06; 1.00)** | **0.52 ( 0.25; 0.80)** | **0.51 ( 0.13; 0.88)** | 0.37 (-0.19; 0.93) | **0.40 ( 0.06; 0.73)** | **0.37 ( 0.06; 0.67)** | 0.33 (-0.11; 0.76) | **0.32 ( 0.00; 0.65)** | 0.07 (-0.32; 0.47) | -0.06 (-0.63; 0.50) | **CON** |

## Table 6.5: League Table of Reactive Balance

| **ECA** | NA | NA | **1.18 ( 0.36; 2.01)** | NA | NA | NA | NA | NA | NA | NA | NA | NA |
| --- | --- | --- | --- | --- | --- | --- | --- | --- | --- | --- | --- | --- |
| 0.12 (-1.21; 1.45) | **RA** | NA | NA | NA | NA | NA | NA | **1.22 ( 0.43; 2.01)** | NA | NA | NA | NA |
| **1.15 ( 0.09; 2.20)** | 1.02 (-0.03; 2.08) | **DT** | NA | NA | NA | NA | NA | NA | NA | NA | NA | **0.65 ( 0.18; 1.13)** |
| **1.18 ( 0.36; 2.01)** | **1.06 ( 0.02; 2.11)** | 0.04 (-0.62; 0.69) | **MC** | NA | NA | NA | NA | NA | NA | NA | NA | **0.61 ( 0.17; 1.06)** |
| 1.18 (-0.06; 2.42) | 1.06 (-0.18; 2.30) | 0.03 (-0.90; 0.97) | -0.00 (-0.92; 0.92) | **TC** | 0.04 (-0.67; 0.74) | 0.07 (-0.65; 0.78) | NA | NA | NA | NA | NA | NA |
| **1.22 ( 0.03; 2.41)** | 1.09 (-0.10; 2.29) | 0.07 (-0.80; 0.94) | 0.03 (-0.82; 0.89) | 0.04 (-0.67; 0.74) | **BGT** | 0.03 (-0.60; 0.66) | NA | NA | NA | NA | NA | NA |
| **1.25 ( 0.24; 2.26)** | **1.13 ( 0.11; 2.14)** | 0.10 (-0.50; 0.70) | 0.06 (-0.52; 0.64) | 0.07 (-0.65; 0.78) | 0.03 (-0.60; 0.66) | **RT** | NA | NA | 0.20 (-0.41; 0.80) | NA | **0.93 ( 0.30; 1.56)** | 0.40 (-0.01; 0.80) |
| **1.33 ( 0.19; 2.46)** | **1.20 ( 0.06; 2.35)** | 0.18 (-0.62; 0.98) | 0.14 (-0.64; 0.93) | 0.15 (-0.88; 1.17) | 0.11 (-0.86; 1.08) | 0.08 (-0.66; 0.82) | **PT** | NA | NA | NA | NA | 0.47 (-0.17; 1.11) |
| **1.34 ( 0.27; 2.42)** | **1.22 ( 0.43; 2.01)** | 0.20 (-0.51; 0.90) | 0.16 (-0.53; 0.85) | 0.16 (-0.80; 1.12) | 0.12 (-0.77; 1.02) | 0.09 (-0.55; 0.73) | 0.02 (-0.81; 0.84) | **CPP** | NA | NA | NA | 0.46 (-0.07; 0.98) |
| **1.45 ( 0.27; 2.62)** | **1.32 ( 0.14; 2.50)** | 0.30 (-0.55; 1.15) | 0.26 (-0.58; 1.10) | 0.27 (-0.67; 1.20) | 0.23 (-0.65; 1.10) | 0.20 (-0.41; 0.80) | 0.12 (-0.83; 1.07) | 0.10 (-0.77; 0.98) | **STR** | NA | NA | NA |
| **1.68 ( 0.46; 2.90)** | **1.56 ( 0.33; 2.78)** | 0.53 (-0.38; 1.45) | 0.50 (-0.40; 1.40) | 0.50 (-0.62; 1.62) | 0.46 (-0.60; 1.53) | 0.43 (-0.43; 1.29) | 0.35 (-0.66; 1.36) | 0.34 (-0.60; 1.28) | 0.23 (-0.82; 1.29) | **YOG** | NA | 0.12 (-0.66; 0.90) |
| **1.81 ( 0.77; 2.85)** | **1.69 ( 0.64; 2.73)** | **0.66 ( 0.01; 1.32)** | 0.63 (-0.01; 1.26) | 0.63 (-0.22; 1.48) | 0.59 (-0.19; 1.38) | **0.56 ( 0.10; 1.03)** | 0.48 (-0.30; 1.27) | 0.47 (-0.22; 1.16) | 0.36 (-0.40; 1.13) | 0.13 (-0.77; 1.03) | **VR** | 0.27 (-0.28; 0.83) |
| **1.80 ( 0.86; 2.74)** | **1.68 ( 0.73; 2.62)** | **0.65 ( 0.18; 1.13)** | **0.61 ( 0.17; 1.06)** | 0.62 (-0.19; 1.42) | 0.58 (-0.15; 1.31) | **0.55 ( 0.18; 0.92)** | 0.47 (-0.17; 1.11) | 0.46 (-0.07; 0.98) | 0.35 (-0.35; 1.06) | 0.12 (-0.66; 0.90) | -0.01 (-0.46; 0.43) | **CON** |

All results are presented in the form of SMD (95% CrI). Exercise types are ranked according to the P score for balance starting with the best from left to right. The results of the network meta-analysis are showed in the lower left part, and results from pairwise comparisons in the upper right half (if available). Cells shown in bold indicate significant results. *NA* not available, *SMD* standardized Mean Difference, *CrI* Credible Interval, *AE* Aerobic Exercise, *AQE* Aquatic Exercise, *BGT* Balance and Gait Training, *BWS* Body Weight Support Treadmill Training, *CON* Control group, *CPP* Classic Physiotherapy Program, *DAN* Dance, *DT* Dual Task Balance and Gait Training, *ECA* Balance and Gait Training with External Cue or Attention, *ICA* Balance and Gait Training with Internal Cue or Attention, *MC* Multicomponent Exercise Program, *MD* Multidisciplinary Exercise Program, *PIL* Pilates, *NW* Nordic Walking, *PT* Power Training, *QIG* Qigong*, RS* Robotic Assisted Gait Training, *RT* Resistance Training, *STR* Stretch, *TAN* Tango, *TC* Tai Chi, *TT* Treadmill Training, *VR* Virtual Reality, *WBV* Whole Body Vibration, *YOG* Yoga.

# Appendix 7: Details of SIDE splitting results

## Table 7.1 Details of SIDE splitting results (Balance test batteries)

| **Comparison** | **k** | **prop** | **NMA** | | **Direct** | | **Indir** | | **Diff** | | **z** | **p** |
| --- | --- | --- | --- | --- | --- | --- | --- | --- | --- | --- | --- | --- |
|  |  |  | **TE** | **seTE** | **TE** | **seTE** | **TE** | **seTE** | **TE** | **seTE** |  |  |
| AE vs AQE | 0 | 0 | -0.2185039 | 0.20781059 | NA | NA | -0.2185039 | 0.20781059 | NA | NA | NA | NA |
| AE vs BGT | 2 | 0.23827195 | 0.23477347 | 0.16388348 | 0.50522578 | 0.33573662 | 0.15017477 | 0.18777389 | 0.35505101 | 0.38467923 | 0.92297941 | 0.35601793 |
| AE vs BGT_ECA | 1 | 0.24351095 | -0.210833 | 0.17455305 | 0.04583171 | 0.35372699 | -0.2934524 | 0.20069018 | 0.33928411 | 0.40669317 | 0.83425083 | 0.40413966 |
| AE vs BGT_ICA | 0 | 0 | -0.0760518 | 0.35933343 | NA | NA | -0.0760518 | 0.35933343 | NA | NA | NA | NA |
| AE vs BWS_TT | 0 | 0 | -1.086179 | 0.43684609 | NA | NA | -1.086179 | 0.43684609 | NA | NA | NA | NA |
| AE vs CON | 1 | 0.17298843 | 0.47066231 | 0.15749468 | 0.67451519 | 0.37866692 | 0.4280218 | 0.17318493 | 0.24649339 | 0.41639123 | 0.59197546 | 0.55386702 |
| AE vs CPP | 0 | 0 | 0.39475527 | 0.18929441 | NA | NA | 0.39475527 | 0.18929441 | NA | NA | NA | NA |
| AE vs Dance | 0 | 0 | -0.2401178 | 0.23231409 | NA | NA | -0.2401178 | 0.23231409 | NA | NA | NA | NA |
| AE vs DT_BGT | 0 | 0 | -0.1465425 | 0.18352151 | NA | NA | -0.1465425 | 0.18352151 | NA | NA | NA | NA |
| AE vs Mul_C | 1 | 0.16092143 | -0.0294447 | 0.17426443 | -0.0359233 | 0.434412 | -0.0282022 | 0.19024245 | -0.007721 | 0.47424253 | -0.0162808 | 0.98701039 |
| AE vs Mul_D | 0 | 0 | -0.3922029 | 0.23887463 | NA | NA | -0.3922029 | 0.23887463 | NA | NA | NA | NA |
| AE vs NW | 1 | 0.4354334 | -0.377579 | 0.31073771 | 0.19892159 | 0.47090477 | -0.8222167 | 0.41355794 | 1.02113832 | 0.62672281 | 1.62933008 | 0.10324316 |
| AE vs Pilates | 0 | 0 | -0.4429188 | 0.35280514 | NA | NA | -0.4429188 | 0.35280514 | NA | NA | NA | NA |
| AE vs PT | 0 | 0 | 0.01039993 | 0.44350044 | NA | NA | 0.01039993 | 0.44350044 | NA | NA | NA | NA |
| AE vs Qigong | 0 | 0 | 0.16488344 | 0.25674097 | NA | NA | 0.16488344 | 0.25674097 | NA | NA | NA | NA |
| AE vs RA_GT | 1 | 0.32811646 | -0.2156815 | 0.2288036 | -0.3475544 | 0.39943752 | -0.1512809 | 0.27913601 | -0.1962735 | 0.48730611 | -0.4027724 | 0.68711565 |
| AE vs RT | 1 | 0.09138148 | 0.15133939 | 0.18744711 | -0.1861597 | 0.62008273 | 0.18528232 | 0.19664728 | -0.371442 | 0.65051729 | -0.5709949 | 0.56800312 |
| AE vs Stretch | 1 | 0.35383953 | 0.44618941 | 0.17767136 | -0.0410777 | 0.29868558 | 0.71301843 | 0.22102797 | -0.7540962 | 0.37157293 | -2.0294701 | 0.04241043 |
| AE vs Tango | 0 | 0 | -0.0697233 | 0.22542982 | NA | NA | -0.0697233 | 0.22542982 | NA | NA | NA | NA |
| AE vs TC | 0 | 0 | 0.032767 | 0.25351525 | NA | NA | 0.032767 | 0.25351525 | NA | NA | NA | NA |
| AE vs TT | 1 | 0.16191524 | 0.04573487 | 0.1796778 | -0.2723458 | 0.4465299 | 0.10718702 | 0.19626843 | -0.3795328 | 0.48776044 | -0.7781132 | 0.43650229 |
| AE vs VR | 1 | 0.21852363 | 0.0321284 | 0.17418567 | 0.23732942 | 0.37261746 | -0.0252518 | 0.19704004 | 0.26258122 | 0.42150748 | 0.62295745 | 0.53331248 |
| AE vs WBV | 0 | 0 | -0.252968 | 0.53232939 | NA | NA | -0.252968 | 0.53232939 | NA | NA | NA | NA |
| AE vs Yoga | 0 | 0 | 0.17195056 | 0.25489243 | NA | NA | 0.17195056 | 0.25489243 | NA | NA | NA | NA |
| AQE vs BGT | 1 | 0.16670477 | 0.45327735 | 0.1754924 | 0.71181906 | 0.42981771 | 0.40155482 | 0.19224669 | 0.31026424 | 0.47085247 | 0.65894151 | 0.50993333 |
| AQE vs BGT_ECA | 0 | 0 | 0.00767087 | 0.19089711 | NA | NA | 0.00767087 | 0.19089711 | NA | NA | NA | NA |
| AQE vs BGT_ICA | 0 | 0 | 0.14245206 | 0.36312615 | NA | NA | 0.14245206 | 0.36312615 | NA | NA | NA | NA |
| AQE vs BWS_TT | 0 | 0 | -0.8676751 | 0.44595618 | NA | NA | -0.8676751 | 0.44595618 | NA | NA | NA | NA |
| AQE vs CON | 1 | 0.08790039 | 0.68916618 | 0.15642556 | 1.5648907 | 0.52760913 | 0.60477131 | 0.1637897 | 0.96011939 | 0.5524477 | 1.73793716 | 0.08222189 |
| AQE vs CPP | 5 | 0.48648272 | 0.61325915 | 0.15323248 | 0.42616631 | 0.21969346 | 0.79050233 | 0.2138323 | -0.364336 | 0.30657702 | -1.1883997 | 0.23467598 |
| AQE vs Dance | 0 | 0 | -0.0216139 | 0.22694932 | NA | NA | -0.0216139 | 0.22694932 | NA | NA | NA | NA |
| AQE vs DT_BGT | 0 | 0 | 0.0719614 | 0.19636843 | NA | NA | 0.0719614 | 0.19636843 | NA | NA | NA | NA |
| AQE vs Mul_C | 4 | 0.43829786 | 0.18905915 | 0.15370279 | 0.13647731 | 0.23216517 | 0.23008891 | 0.20508255 | -0.0936116 | 0.30977333 | -0.3021939 | 0.76250429 |
| AQE vs Mul_D | 2 | 0.42911183 | -0.173699 | 0.19724741 | -0.1459065 | 0.30111061 | -0.1945894 | 0.26105726 | 0.04868286 | 0.39852038 | 0.12215903 | 0.90277308 |
| AQE vs NW | 0 | 0 | -0.1590751 | 0.34084675 | NA | NA | -0.1590751 | 0.34084675 | NA | NA | NA | NA |
| AQE vs Pilates | 0 | 0 | -0.2244149 | 0.35538787 | NA | NA | -0.2244149 | 0.35538787 | NA | NA | NA | NA |
| AQE vs PT | 0 | 0 | 0.22890381 | 0.44394233 | NA | NA | 0.22890381 | 0.44394233 | NA | NA | NA | NA |
| AQE vs Qigong | 0 | 0 | 0.38338732 | 0.24770962 | NA | NA | 0.38338732 | 0.24770962 | NA | NA | NA | NA |
| AQE vs RA_GT | 0 | 0 | 0.00282241 | 0.2533567 | NA | NA | 0.00282241 | 0.2533567 | NA | NA | NA | NA |
| AQE vs RT | 0 | 0 | 0.36984327 | 0.18934474 | NA | NA | 0.36984327 | 0.18934474 | NA | NA | NA | NA |
| AQE vs Stretch | 0 | 0 | 0.66469329 | 0.21029578 | NA | NA | 0.66469329 | 0.21029578 | NA | NA | NA | NA |
| AQE vs Tango | 0 | 0 | 0.1487806 | 0.23171897 | NA | NA | 0.1487806 | 0.23171897 | NA | NA | NA | NA |
| AQE vs TC | 0 | 0 | 0.25127088 | 0.25064526 | NA | NA | 0.25127088 | 0.25064526 | NA | NA | NA | NA |
| AQE vs TT | 0 | 0 | 0.26423874 | 0.20081463 | NA | NA | 0.26423874 | 0.20081463 | NA | NA | NA | NA |
| AQE vs VR | 0 | 0 | 0.25063228 | 0.18982005 | NA | NA | 0.25063228 | 0.18982005 | NA | NA | NA | NA |
| AQE vs WBV | 0 | 0 | -0.0344641 | 0.53689384 | NA | NA | -0.0344641 | 0.53689384 | NA | NA | NA | NA |
| AQE vs Yoga | 0 | 0 | 0.39045444 | 0.25677415 | NA | NA | 0.39045444 | 0.25677415 | NA | NA | NA | NA |
| BGT vs BGT_ECA | 6 | 0.60884952 | -0.4456065 | 0.13249861 | -0.6111852 | 0.16980729 | -0.1878732 | 0.21185532 | -0.423312 | 0.2715091 | -1.559108 | 0.11897082 |
| BGT vs BGT_ICA | 1 | 0.45159245 | -0.3108253 | 0.33607484 | -0.1960061 | 0.50010669 | -0.4053745 | 0.45382069 | 0.20936845 | 0.67532209 | 0.31002755 | 0.75654001 |
| BGT vs BWS_TT | 0 | 0 | -1.3209524 | 0.42933847 | NA | NA | -1.3209524 | 0.42933847 | NA | NA | NA | NA |
| BGT vs CON | 3 | 0.22012535 | 0.23588884 | 0.11725082 | 0.36516247 | 0.24990839 | 0.1994004 | 0.13277108 | 0.16576207 | 0.28298827 | 0.58575596 | 0.55803952 |
| BGT vs CPP | 0 | 0 | 0.1599818 | 0.15490663 | NA | NA | 0.1599818 | 0.15490663 | NA | NA | NA | NA |
| BGT vs Dance | 0 | 0 | -0.4748913 | 0.20763278 | NA | NA | -0.4748913 | 0.20763278 | NA | NA | NA | NA |
| BGT vs DT_BGT | 0 | 0 | -0.3813159 | 0.15780395 | NA | NA | -0.3813159 | 0.15780395 | NA | NA | NA | NA |
| BGT vs Mul_C | 0 | 0 | -0.2642182 | 0.14459176 | NA | NA | -0.2642182 | 0.14459176 | NA | NA | NA | NA |
| BGT vs Mul_D | 0 | 0 | -0.6269763 | 0.21303376 | NA | NA | -0.6269763 | 0.21303376 | NA | NA | NA | NA |
| BGT vs NW | 0 | 0 | -0.6123525 | 0.31939189 | NA | NA | -0.6123525 | 0.31939189 | NA | NA | NA | NA |
| BGT vs Pilates | 1 | 0.52293394 | -0.6776923 | 0.32467587 | -0.7040486 | 0.4489796 | -0.6488019 | 0.47006808 | -0.0552467 | 0.6500359 | -0.0849903 | 0.93226912 |
| BGT vs PT | 0 | 0 | -0.2243735 | 0.42736493 | NA | NA | -0.2243735 | 0.42736493 | NA | NA | NA | NA |
| BGT vs Qigong | 0 | 0 | -0.06989 | 0.23363507 | NA | NA | -0.06989 | 0.23363507 | NA | NA | NA | NA |
| BGT vs RA_GT | 2 | 0.52506319 | -0.4504549 | 0.20866467 | -0.3006932 | 0.28796728 | -0.6160231 | 0.30278267 | 0.31532989 | 0.41785464 | 0.75464015 | 0.45046492 |
| BGT vs RT | 4 | 0.39828925 | -0.0834341 | 0.14684168 | -0.0862197 | 0.23267518 | -0.0815902 | 0.18930211 | -0.0046296 | 0.29995504 | -0.0154343 | 0.98768573 |
| BGT vs Stretch | 0 | 0 | 0.21141594 | 0.16976408 | NA | NA | 0.21141594 | 0.16976408 | NA | NA | NA | NA |
| BGT vs Tango | 0 | 0 | -0.3044967 | 0.20409874 | NA | NA | -0.3044967 | 0.20409874 | NA | NA | NA | NA |
| BGT vs TC | 1 | 0.18248402 | -0.2020065 | 0.22714067 | -0.2963999 | 0.53171938 | -0.1809362 | 0.2512157 | -0.1154637 | 0.58807723 | -0.1963411 | 0.84434321 |
| BGT vs TT | 0 | 0 | -0.1890386 | 0.16056502 | NA | NA | -0.1890386 | 0.16056502 | NA | NA | NA | NA |
| BGT vs VR | 5 | 0.48262842 | -0.2026451 | 0.13567871 | -0.0639582 | 0.19530136 | -0.3320187 | 0.18862985 | 0.26806055 | 0.27152135 | 0.98725406 | 0.32351811 |
| BGT vs WBV | 0 | 0 | -0.4877415 | 0.52401958 | NA | NA | -0.4877415 | 0.52401958 | NA | NA | NA | NA |
| BGT vs Yoga | 2 | 0.4335849 | -0.0628229 | 0.22038563 | -0.1592541 | 0.33469267 | 0.01099414 | 0.2928302 | -0.1702482 | 0.44471194 | -0.382828 | 0.70184727 |
| BGT_ECA vs BGT_ICA | 1 | 0.46088619 | 0.13478119 | 0.33903561 | -0.0522683 | 0.49939992 | 0.29468902 | 0.46174807 | -0.3469573 | 0.68015554 | -0.5101146 | 0.60997115 |
| BGT_ECA vs BWS_TT | 0 | 0 | -0.875346 | 0.43155848 | NA | NA | -0.875346 | 0.43155848 | NA | NA | NA | NA |
| BGT_ECA vs CON | 3 | 0.36813475 | 0.68149531 | 0.13632127 | 0.55571818 | 0.22467783 | 0.75477508 | 0.17149497 | -0.1990569 | 0.28264934 | -0.7042539 | 0.48127469 |
| BGT_ECA vs CPP | 1 | 0.07507124 | 0.60558828 | 0.17093836 | 1.0434896 | 0.62388241 | 0.57004631 | 0.17774009 | 0.47344329 | 0.64870702 | 0.72982607 | 0.46549651 |
| BGT_ECA vs Dance | 0 | 0 | -0.0292848 | 0.21919491 | NA | NA | -0.0292848 | 0.21919491 | NA | NA | NA | NA |
| BGT_ECA vs DT_BGT | 0 | 0 | 0.06429053 | 0.17490278 | NA | NA | 0.06429053 | 0.17490278 | NA | NA | NA | NA |
| BGT_ECA vs Mul_C | 0 | 0 | 0.18138828 | 0.16020486 | NA | NA | 0.18138828 | 0.16020486 | NA | NA | NA | NA |
| BGT_ECA vs Mul_D | 1 | 0.19749349 | -0.1813699 | 0.21845701 | 0.21909881 | 0.49157477 | -0.2799235 | 0.24386063 | 0.49902232 | 0.54873834 | 0.90939941 | 0.36313933 |
| BGT_ECA vs NW | 0 | 0 | -0.166746 | 0.32469921 | NA | NA | -0.166746 | 0.32469921 | NA | NA | NA | NA |
| BGT_ECA vs Pilates | 0 | 0 | -0.2320858 | 0.34159994 | NA | NA | -0.2320858 | 0.34159994 | NA | NA | NA | NA |
| BGT_ECA vs PT | 0 | 0 | 0.22123294 | 0.43578009 | NA | NA | 0.22123294 | 0.43578009 | NA | NA | NA | NA |
| BGT_ECA vs Qigong | 0 | 0 | 0.37571645 | 0.24404383 | NA | NA | 0.37571645 | 0.24404383 | NA | NA | NA | NA |
| BGT_ECA vs RA_GT | 0 | 0 | -0.0048485 | 0.23088155 | NA | NA | -0.0048485 | 0.23088155 | NA | NA | NA | NA |
| BGT_ECA vs RT | 0 | 0 | 0.3621724 | 0.17293015 | NA | NA | 0.3621724 | 0.17293015 | NA | NA | NA | NA |
| BGT_ECA vs Stretch | 0 | 0 | 0.65702242 | 0.18663085 | NA | NA | 0.65702242 | 0.18663085 | NA | NA | NA | NA |
| BGT_ECA vs Tango | 0 | 0 | 0.14110973 | 0.21573485 | NA | NA | 0.14110973 | 0.21573485 | NA | NA | NA | NA |
| BGT_ECA vs TC | 0 | 0 | 0.24360001 | 0.24092759 | NA | NA | 0.24360001 | 0.24092759 | NA | NA | NA | NA |
| BGT_ECA vs TT | 2 | 0.30676263 | 0.25656787 | 0.1664101 | 0.12058803 | 0.30045433 | 0.31673995 | 0.1998659 | -0.1961519 | 0.36085895 | -0.5435695 | 0.58673776 |
| BGT_ECA vs VR | 0 | 0 | 0.24296141 | 0.16440863 | NA | NA | 0.24296141 | 0.16440863 | NA | NA | NA | NA |
| BGT_ECA vs WBV | 0 | 0 | -0.042135 | 0.52941989 | NA | NA | -0.042135 | 0.52941989 | NA | NA | NA | NA |
| BGT_ECA vs Yoga | 0 | 0 | 0.38278357 | 0.24028522 | NA | NA | 0.38278357 | 0.24028522 | NA | NA | NA | NA |
| BGT_ICA vs BWS_TT | 0 | 0 | -1.0101271 | 0.53413024 | NA | NA | -1.0101271 | 0.53413024 | NA | NA | NA | NA |
| BGT_ICA vs CON | 1 | 0.43460739 | 0.54671413 | 0.33386516 | 0.49608617 | 0.50643364 | 0.58563095 | 0.44401336 | -0.0895448 | 0.67351533 | -0.1329514 | 0.89423185 |
| BGT_ICA vs CPP | 0 | 0 | 0.47080709 | 0.35340906 | NA | NA | 0.47080709 | 0.35340906 | NA | NA | NA | NA |
| BGT_ICA vs Dance | 0 | 0 | -0.164066 | 0.37678995 | NA | NA | -0.164066 | 0.37678995 | NA | NA | NA | NA |
| BGT_ICA vs DT_BGT | 0 | 0 | -0.0704907 | 0.3539171 | NA | NA | -0.0704907 | 0.3539171 | NA | NA | NA | NA |
| BGT_ICA vs Mul_C | 0 | 0 | 0.04660709 | 0.3464886 | NA | NA | 0.04660709 | 0.3464886 | NA | NA | NA | NA |
| BGT_ICA vs Mul_D | 0 | 0 | -0.316151 | 0.38108854 | NA | NA | -0.316151 | 0.38108854 | NA | NA | NA | NA |
| BGT_ICA vs NW | 0 | 0 | -0.3015272 | 0.45007504 | NA | NA | -0.3015272 | 0.45007504 | NA | NA | NA | NA |
| BGT_ICA vs Pilates | 0 | 0 | -0.366867 | 0.45917621 | NA | NA | -0.366867 | 0.45917621 | NA | NA | NA | NA |
| BGT_ICA vs PT | 0 | 0 | 0.08645175 | 0.5322466 | NA | NA | 0.08645175 | 0.5322466 | NA | NA | NA | NA |
| BGT_ICA vs Qigong | 0 | 0 | 0.24093526 | 0.39169077 | NA | NA | 0.24093526 | 0.39169077 | NA | NA | NA | NA |
| BGT_ICA vs RA_GT | 0 | 0 | -0.1396296 | 0.38616143 | NA | NA | -0.1396296 | 0.38616143 | NA | NA | NA | NA |
| BGT_ICA vs RT | 0 | 0 | 0.22739121 | 0.35297418 | NA | NA | 0.22739121 | 0.35297418 | NA | NA | NA | NA |
| BGT_ICA vs Stretch | 0 | 0 | 0.52224123 | 0.36199185 | NA | NA | 0.52224123 | 0.36199185 | NA | NA | NA | NA |
| BGT_ICA vs Tango | 0 | 0 | 0.00632854 | 0.37605487 | NA | NA | 0.00632854 | 0.37605487 | NA | NA | NA | NA |
| BGT_ICA vs TC | 0 | 0 | 0.10881882 | 0.38941239 | NA | NA | 0.10881882 | 0.38941239 | NA | NA | NA | NA |
| BGT_ICA vs TT | 0 | 0 | 0.12178669 | 0.35601224 | NA | NA | 0.12178669 | 0.35601224 | NA | NA | NA | NA |
| BGT_ICA vs VR | 0 | 0 | 0.10818022 | 0.35117912 | NA | NA | 0.10818022 | 0.35117912 | NA | NA | NA | NA |
| BGT_ICA vs WBV | 0 | 0 | -0.1769162 | 0.61233304 | NA | NA | -0.1769162 | 0.61233304 | NA | NA | NA | NA |
| BGT_ICA vs Yoga | 0 | 0 | 0.24800238 | 0.38924411 | NA | NA | 0.24800238 | 0.38924411 | NA | NA | NA | NA |
| BWS_TT vs CON | 0 | 0 | 1.55684126 | 0.42468779 | NA | NA | 1.55684126 | 0.42468779 | NA | NA | NA | NA |
| BWS_TT vs CPP | 0 | 0 | 1.48093423 | 0.43725047 | NA | NA | 1.48093423 | 0.43725047 | NA | NA | NA | NA |
| BWS_TT vs Dance | 0 | 0 | 0.84606117 | 0.45669495 | NA | NA | 0.84606117 | 0.45669495 | NA | NA | NA | NA |
| BWS_TT vs DT_BGT | 0 | 0 | 0.93963648 | 0.43378745 | NA | NA | 0.93963648 | 0.43378745 | NA | NA | NA | NA |
| BWS_TT vs Mul_C | 0 | 0 | 1.05673423 | 0.43127796 | NA | NA | 1.05673423 | 0.43127796 | NA | NA | NA | NA |
| BWS_TT vs Mul_D | 0 | 0 | 0.6939761 | 0.46114353 | NA | NA | 0.6939761 | 0.46114353 | NA | NA | NA | NA |
| BWS_TT vs NW | 0 | 0 | 0.70859995 | 0.50675907 | NA | NA | 0.70859995 | 0.50675907 | NA | NA | NA | NA |
| BWS_TT vs Pilates | 0 | 0 | 0.64326017 | 0.53009246 | NA | NA | 0.64326017 | 0.53009246 | NA | NA | NA | NA |
| BWS_TT vs PT | 0 | 0 | 1.09657889 | 0.59381411 | NA | NA | 1.09657889 | 0.59381411 | NA | NA | NA | NA |
| BWS_TT vs Qigong | 0 | 0 | 1.2510624 | 0.47053266 | NA | NA | 1.2510624 | 0.47053266 | NA | NA | NA | NA |
| BWS_TT vs RA_GT | 0 | 0 | 0.87049749 | 0.46309323 | NA | NA | 0.87049749 | 0.46309323 | NA | NA | NA | NA |
| BWS_TT vs RT | 0 | 0 | 1.23751835 | 0.43831837 | NA | NA | 1.23751835 | 0.43831837 | NA | NA | NA | NA |
| BWS_TT vs Stretch | 0 | 0 | 1.53236837 | 0.43704295 | NA | NA | 1.53236837 | 0.43704295 | NA | NA | NA | NA |
| BWS_TT vs Tango | 0 | 0 | 1.01645568 | 0.44924245 | NA | NA | 1.01645568 | 0.44924245 | NA | NA | NA | NA |
| BWS_TT vs TC | 0 | 0 | 1.11894596 | 0.46913983 | NA | NA | 1.11894596 | 0.46913983 | NA | NA | NA | NA |
| BWS_TT vs TT | 2 | 1 | 1.13191382 | 0.39818387 | 1.13191382 | 0.39818387 | NA | NA | NA | NA | NA | NA |
| BWS_TT vs VR | 0 | 0 | 1.11830736 | 0.42777951 | NA | NA | 1.11830736 | 0.42777951 | NA | NA | NA | NA |
| BWS_TT vs WBV | 0 | 0 | 0.83321097 | 0.66171443 | NA | NA | 0.83321097 | 0.66171443 | NA | NA | NA | NA |
| BWS_TT vs Yoga | 0 | 0 | 1.25812952 | 0.47029863 | NA | NA | 1.25812952 | 0.47029863 | NA | NA | NA | NA |
| CPP vs CON | 1 | 0.09312988 | 0.07590703 | 0.13257093 | 0.04655807 | 0.43441395 | 0.07892099 | 0.1392117 | -0.0323629 | 0.45617472 | -0.0709441 | 0.94344222 |
| Dance vs CON | 4 | 0.67517042 | 0.71078009 | 0.17893387 | 0.76903561 | 0.21776388 | 0.58969383 | 0.31395309 | 0.17934177 | 0.3820833 | 0.46937873 | 0.63879895 |
| DT_BGT vs CON | 5 | 0.64310219 | 0.61720478 | 0.12982119 | 0.4859739 | 0.16188463 | 0.85367266 | 0.21730697 | -0.3676988 | 0.27097777 | -1.3569333 | 0.1748024 |
| Mul_C vs CON | 7 | 0.66990228 | 0.50010704 | 0.10306808 | 0.45645325 | 0.12592689 | 0.58869828 | 0.17939195 | -0.132245 | 0.21917813 | -0.6033678 | 0.54626402 |
| Mul_D vs CON | 1 | 0.1467969 | 0.86286517 | 0.19607335 | 0.33515007 | 0.51175267 | 0.95366059 | 0.21227182 | -0.6185105 | 0.5540308 | -1.1163829 | 0.26425826 |
| NW vs CON | 1 | 0.31011741 | 0.84824131 | 0.30987148 | 1.2902813 | 0.55644024 | 0.64953461 | 0.37307316 | 0.64074669 | 0.66993233 | 0.95643495 | 0.33885253 |
| Pilates vs CON | 1 | 0.51073514 | 0.9135811 | 0.32494303 | 0.88655081 | 0.45468367 | 0.94179754 | 0.46455294 | -0.0552467 | 0.6500359 | -0.0849903 | 0.93226912 |
| PT vs CON | 1 | 0.73826333 | 0.46026238 | 0.41736196 | 0.6263508 | 0.48574375 | -0.0082123 | 0.81579413 | 0.63456305 | 0.94945619 | 0.66834369 | 0.50391423 |
| Qigong vs CON | 2 | 0.6828987 | 0.30577886 | 0.20835249 | 0.36294479 | 0.25212769 | 0.18266823 | 0.36999827 | 0.18027656 | 0.44773552 | 0.40264075 | 0.68721253 |
| RA_GT vs CON | 0 | 0 | 0.68634377 | 0.21242749 | NA | NA | 0.68634377 | 0.21242749 | NA | NA | NA | NA |
| RT vs CON | 4 | 0.43234155 | 0.31932291 | 0.13434305 | 0.45934193 | 0.20431569 | 0.21268126 | 0.17830834 | 0.24666067 | 0.27118032 | 0.90958174 | 0.36304313 |
| Stretch vs CON | 0 | 0 | 0.0244729 | 0.15809037 | NA | NA | 0.0244729 | 0.15809037 | NA | NA | NA | NA |
| Tango vs CON | 3 | 0.41628801 | 0.54038558 | 0.18096492 | 0.4690028 | 0.28047712 | 0.5912939 | 0.23686183 | -0.1222911 | 0.36711163 | -0.3331169 | 0.73904603 |
| TC vs CON | 2 | 0.57281279 | 0.4378953 | 0.20562786 | 0.39650493 | 0.27169122 | 0.49339541 | 0.31461023 | -0.0968905 | 0.41568704 | -0.2330851 | 0.81569529 |
| TT vs CON | 1 | 0.09802474 | 0.42492744 | 0.14767979 | 0.94644545 | 0.47168625 | 0.36824998 | 0.15549762 | 0.57819547 | 0.49665625 | 1.16417638 | 0.24435254 |
| VR vs CON | 1 | 0.13577097 | 0.43853391 | 0.13859792 | 0.43718577 | 0.37614323 | 0.4387457 | 0.14908787 | -0.0015599 | 0.40461207 | -0.0038554 | 0.99692387 |
| WBV vs CON | 0 | 0 | 0.72363029 | 0.51628285 | NA | NA | 0.72363029 | 0.51628285 | NA | NA | NA | NA |
| Yoga vs CON | 3 | 0.60379185 | 0.29871175 | 0.21041459 | 0.28530025 | 0.27078976 | 0.31914987 | 0.33428289 | -0.0338496 | 0.43020012 | -0.0786834 | 0.93728443 |
| CPP vs Dance | 2 | 0.38295151 | -0.6348731 | 0.20120258 | -0.7357023 | 0.32513346 | -0.5722966 | 0.25613794 | -0.1634057 | 0.41390628 | -0.394789 | 0.69299859 |
| CPP vs DT_BGT | 0 | 0 | -0.5412978 | 0.17620253 | NA | NA | -0.5412978 | 0.17620253 | NA | NA | NA | NA |
| CPP vs Mul_C | 2 | 0.20457245 | -0.4242 | 0.14320159 | -0.6157277 | 0.31660973 | -0.3749418 | 0.16056376 | -0.2407859 | 0.3549964 | -0.678277 | 0.4975961 |
| CPP vs Mul_D | 1 | 0.31484823 | -0.7869581 | 0.19688522 | -0.991917 | 0.35088314 | -0.6927733 | 0.23785908 | -0.2991437 | 0.42390555 | -0.7056848 | 0.48038416 |
| CPP vs NW | 0 | 0 | -0.7723343 | 0.32992006 | NA | NA | -0.7723343 | 0.32992006 | NA | NA | NA | NA |
| CPP vs Pilates | 0 | 0 | -0.8376741 | 0.34562032 | NA | NA | -0.8376741 | 0.34562032 | NA | NA | NA | NA |
| CPP vs PT | 0 | 0 | -0.3843553 | 0.43618887 | NA | NA | -0.3843553 | 0.43618887 | NA | NA | NA | NA |
| CPP vs Qigong | 1 | 0.41419994 | -0.2298718 | 0.22054706 | -0.1242658 | 0.34268591 | -0.3045424 | 0.28815527 | 0.18027656 | 0.44773552 | 0.40264075 | 0.68721253 |
| CPP vs RA_GT | 0 | 0 | -0.6104367 | 0.23839267 | NA | NA | -0.6104367 | 0.23839267 | NA | NA | NA | NA |
| CPP vs RT | 2 | 0.35597392 | -0.2434159 | 0.15900545 | 0.0535512 | 0.26650351 | -0.4075591 | 0.19813459 | 0.46111032 | 0.33208649 | 1.3885248 | 0.1649773 |
| CPP vs Stretch | 0 | 0 | 0.05143413 | 0.18955379 | NA | NA | 0.05143413 | 0.18955379 | NA | NA | NA | NA |
| CPP vs Tango | 0 | 0 | -0.4644785 | 0.21273531 | NA | NA | -0.4644785 | 0.21273531 | NA | NA | NA | NA |
| CPP vs TC | 0 | 0 | -0.3619883 | 0.2385796 | NA | NA | -0.3619883 | 0.2385796 | NA | NA | NA | NA |
| CPP vs TT | 0 | 0 | -0.3490204 | 0.18065872 | NA | NA | -0.3490204 | 0.18065872 | NA | NA | NA | NA |
| CPP vs VR | 2 | 0.30812657 | -0.3626269 | 0.16219831 | -0.670841 | 0.29220104 | -0.2253634 | 0.19499928 | -0.4454776 | 0.35129214 | -1.2681115 | 0.20475815 |
| CPP vs WBV | 0 | 0 | -0.6477233 | 0.5298507 | NA | NA | -0.6477233 | 0.5298507 | NA | NA | NA | NA |
| CPP vs Yoga | 0 | 0 | -0.2228047 | 0.24323583 | NA | NA | -0.2228047 | 0.24323583 | NA | NA | NA | NA |
| Dance vs DT_BGT | 0 | 0 | 0.09357531 | 0.21715619 | NA | NA | 0.09357531 | 0.21715619 | NA | NA | NA | NA |
| Dance vs Mul_C | 0 | 0 | 0.21067306 | 0.20109349 | NA | NA | 0.21067306 | 0.20109349 | NA | NA | NA | NA |
| Dance vs Mul_D | 0 | 0 | -0.1520851 | 0.25651826 | NA | NA | -0.1520851 | 0.25651826 | NA | NA | NA | NA |
| Dance vs NW | 0 | 0 | -0.1374612 | 0.35453224 | NA | NA | -0.1374612 | 0.35453224 | NA | NA | NA | NA |
| Dance vs Pilates | 0 | 0 | -0.202801 | 0.36913642 | NA | NA | -0.202801 | 0.36913642 | NA | NA | NA | NA |
| Dance vs PT | 0 | 0 | 0.25051772 | 0.45350985 | NA | NA | 0.25051772 | 0.45350985 | NA | NA | NA | NA |
| Dance vs Qigong | 0 | 0 | 0.40500123 | 0.26875392 | NA | NA | 0.40500123 | 0.26875392 | NA | NA | NA | NA |
| Dance vs RA_GT | 0 | 0 | 0.02443632 | 0.27299912 | NA | NA | 0.02443632 | 0.27299912 | NA | NA | NA | NA |
| Dance vs RT | 0 | 0 | 0.39145718 | 0.21510993 | NA | NA | 0.39145718 | 0.21510993 | NA | NA | NA | NA |
| Dance vs Stretch | 0 | 0 | 0.68630719 | 0.23032091 | NA | NA | 0.68630719 | 0.23032091 | NA | NA | NA | NA |
| Dance vs Tango | 2 | 0.34117845 | 0.17039451 | 0.22479195 | -0.0327972 | 0.38484864 | 0.27561973 | 0.27694712 | -0.308417 | 0.47413941 | -0.6504774 | 0.51538392 |
| Dance vs TC | 0 | 0 | 0.27288479 | 0.27090144 | NA | NA | 0.27288479 | 0.27090144 | NA | NA | NA | NA |
| Dance vs TT | 0 | 0 | 0.28585265 | 0.22365124 | NA | NA | 0.28585265 | 0.22365124 | NA | NA | NA | NA |
| Dance vs VR | 0 | 0 | 0.27224619 | 0.21777983 | NA | NA | 0.27224619 | 0.21777983 | NA | NA | NA | NA |
| Dance vs WBV | 0 | 0 | -0.0128502 | 0.54484057 | NA | NA | -0.0128502 | 0.54484057 | NA | NA | NA | NA |
| Dance vs Yoga | 0 | 0 | 0.41206835 | 0.27446911 | NA | NA | 0.41206835 | 0.27446911 | NA | NA | NA | NA |
| DT_BGT vs Mul_C | 0 | 0 | 0.11709775 | 0.16028048 | NA | NA | 0.11709775 | 0.16028048 | NA | NA | NA | NA |
| DT_BGT vs Mul_D | 0 | 0 | -0.2456604 | 0.22934982 | NA | NA | -0.2456604 | 0.22934982 | NA | NA | NA | NA |
| DT_BGT vs NW | 0 | 0 | -0.2310365 | 0.32684397 | NA | NA | -0.2310365 | 0.32684397 | NA | NA | NA | NA |
| DT_BGT vs Pilates | 0 | 0 | -0.2963763 | 0.34576862 | NA | NA | -0.2963763 | 0.34576862 | NA | NA | NA | NA |
| DT_BGT vs PT | 0 | 0 | 0.15694241 | 0.43581145 | NA | NA | 0.15694241 | 0.43581145 | NA | NA | NA | NA |
| DT_BGT vs Qigong | 0 | 0 | 0.31142592 | 0.24305718 | NA | NA | 0.31142592 | 0.24305718 | NA | NA | NA | NA |
| DT_BGT vs RA_GT | 1 | 0.38408509 | -0.069139 | 0.21382869 | 0 | 0.34502633 | -0.1122541 | 0.27246179 | 0.11225412 | 0.43963462 | 0.25533503 | 0.79846434 |
| DT_BGT vs RT | 0 | 0 | 0.29788187 | 0.17559225 | NA | NA | 0.29788187 | 0.17559225 | NA | NA | NA | NA |
| DT_BGT vs Stretch | 2 | 0.40627964 | 0.59273189 | 0.16418852 | 0.99127847 | 0.25759074 | 0.3200086 | 0.21308447 | 0.67126987 | 0.33430223 | 2.00797305 | 0.04464616 |
| DT_BGT vs Tango | 0 | 0 | 0.0768192 | 0.21193231 | NA | NA | 0.0768192 | 0.21193231 | NA | NA | NA | NA |
| DT_BGT vs TC | 0 | 0 | 0.17930948 | 0.24070602 | NA | NA | 0.17930948 | 0.24070602 | NA | NA | NA | NA |
| DT_BGT vs TT | 1 | 0.17740223 | 0.19227734 | 0.17210798 | 0.44513722 | 0.40862174 | 0.13774534 | 0.18976112 | 0.30739188 | 0.45053414 | 0.68228322 | 0.49505988 |
| DT_BGT vs VR | 1 | 0.1487806 | 0.17867088 | 0.16473149 | -0.3856683 | 0.42707434 | 0.27730904 | 0.17854837 | -0.6629773 | 0.46289525 | -1.4322405 | 0.152075 |
| DT_BGT vs WBV | 1 | 1 | -0.1064255 | 0.49969434 | -0.1064255 | 0.49969434 | NA | NA | NA | NA | NA | NA |
| DT_BGT vs Yoga | 0 | 0 | 0.31849304 | 0.24319179 | NA | NA | 0.31849304 | 0.24319179 | NA | NA | NA | NA |
| Mul_C vs Mul_D | 1 | 0.21024384 | -0.3627581 | 0.19822426 | -0.7952416 | 0.43230987 | -0.2476251 | 0.22305414 | -0.5476164 | 0.48646169 | -1.1257134 | 0.26028686 |
| Mul_C vs NW | 0 | 0 | -0.3481343 | 0.32101071 | NA | NA | -0.3481343 | 0.32101071 | NA | NA | NA | NA |
| Mul_C vs Pilates | 0 | 0 | -0.4134741 | 0.33831514 | NA | NA | -0.4134741 | 0.33831514 | NA | NA | NA | NA |
| Mul_C vs PT | 0 | 0 | 0.03984466 | 0.42878769 | NA | NA | 0.03984466 | 0.42878769 | NA | NA | NA | NA |
| Mul_C vs Qigong | 0 | 0 | 0.19432817 | 0.22656689 | NA | NA | 0.19432817 | 0.22656689 | NA | NA | NA | NA |
| Mul_C vs RA_GT | 0 | 0 | -0.1862367 | 0.22927012 | NA | NA | -0.1862367 | 0.22927012 | NA | NA | NA | NA |
| Mul_C vs RT | 0 | 0 | 0.18078412 | 0.15997874 | NA | NA | 0.18078412 | 0.15997874 | NA | NA | NA | NA |
| Mul_C vs Stretch | 0 | 0 | 0.47563414 | 0.17950254 | NA | NA | 0.47563414 | 0.17950254 | NA | NA | NA | NA |
| Mul_C vs Tango | 0 | 0 | -0.0402785 | 0.20302237 | NA | NA | -0.0402785 | 0.20302237 | NA | NA | NA | NA |
| Mul_C vs TC | 1 | 0.29532996 | 0.06221173 | 0.21668065 | 0.19501229 | 0.3987185 | 0.0065545 | 0.25812331 | 0.18845779 | 0.47497799 | 0.39677162 | 0.69153588 |
| Mul_C vs TT | 1 | 0.16915505 | 0.0751796 | 0.16568127 | 0.28794029 | 0.40283846 | 0.03186279 | 0.18176632 | 0.2560775 | 0.44194775 | 0.57942934 | 0.56229951 |
| Mul_C vs VR | 0 | 0 | 0.06157313 | 0.16018859 | NA | NA | 0.06157313 | 0.16018859 | NA | NA | NA | NA |
| Mul_C vs WBV | 0 | 0 | -0.2235233 | 0.52477068 | NA | NA | -0.2235233 | 0.52477068 | NA | NA | NA | NA |
| Mul_C vs Yoga | 0 | 0 | 0.20139529 | 0.23063657 | NA | NA | 0.20139529 | 0.23063657 | NA | NA | NA | NA |
| Mul_D vs NW | 0 | 0 | 0.01462386 | 0.36062328 | NA | NA | 0.01462386 | 0.36062328 | NA | NA | NA | NA |
| Mul_D vs Pilates | 0 | 0 | -0.0507159 | 0.37494691 | NA | NA | -0.0507159 | 0.37494691 | NA | NA | NA | NA |
| Mul_D vs PT | 0 | 0 | 0.40260279 | 0.45956552 | NA | NA | 0.40260279 | 0.45956552 | NA | NA | NA | NA |
| Mul_D vs Qigong | 0 | 0 | 0.5570863 | 0.27530921 | NA | NA | 0.5570863 | 0.27530921 | NA | NA | NA | NA |
| Mul_D vs RA_GT | 0 | 0 | 0.1765214 | 0.28002482 | NA | NA | 0.1765214 | 0.28002482 | NA | NA | NA | NA |
| Mul_D vs RT | 0 | 0 | 0.54354225 | 0.22422183 | NA | NA | 0.54354225 | 0.22422183 | NA | NA | NA | NA |
| Mul_D vs Stretch | 0 | 0 | 0.83839227 | 0.24150051 | NA | NA | 0.83839227 | 0.24150051 | NA | NA | NA | NA |
| Mul_D vs Tango | 0 | 0 | 0.32247959 | 0.26031166 | NA | NA | 0.32247959 | 0.26031166 | NA | NA | NA | NA |
| Mul_D vs TC | 0 | 0 | 0.42496986 | 0.27805803 | NA | NA | 0.42496986 | 0.27805803 | NA | NA | NA | NA |
| Mul_D vs TT | 0 | 0 | 0.43793773 | 0.23260042 | NA | NA | 0.43793773 | 0.23260042 | NA | NA | NA | NA |
| Mul_D vs VR | 0 | 0 | 0.42433126 | 0.22444261 | NA | NA | 0.42433126 | 0.22444261 | NA | NA | NA | NA |
| Mul_D vs WBV | 0 | 0 | 0.13923487 | 0.54981431 | NA | NA | 0.13923487 | 0.54981431 | NA | NA | NA | NA |
| Mul_D vs Yoga | 0 | 0 | 0.56415342 | 0.28312245 | NA | NA | 0.56415342 | 0.28312245 | NA | NA | NA | NA |
| NW vs Pilates | 0 | 0 | -0.0653398 | 0.44461319 | NA | NA | -0.0653398 | 0.44461319 | NA | NA | NA | NA |
| NW vs PT | 0 | 0 | 0.38797893 | 0.518344 | NA | NA | 0.38797893 | 0.518344 | NA | NA | NA | NA |
| NW vs Qigong | 0 | 0 | 0.54246245 | 0.3711651 | NA | NA | 0.54246245 | 0.3711651 | NA | NA | NA | NA |
| NW vs RA_GT | 0 | 0 | 0.16189754 | 0.36145864 | NA | NA | 0.16189754 | 0.36145864 | NA | NA | NA | NA |
| NW vs RT | 0 | 0 | 0.5289184 | 0.33015596 | NA | NA | 0.5289184 | 0.33015596 | NA | NA | NA | NA |
| NW vs Stretch | 0 | 0 | 0.82376841 | 0.33046245 | NA | NA | 0.82376841 | 0.33046245 | NA | NA | NA | NA |
| NW vs Tango | 0 | 0 | 0.30785573 | 0.35000059 | NA | NA | 0.30785573 | 0.35000059 | NA | NA | NA | NA |
| NW vs TC | 0 | 0 | 0.41034601 | 0.36928615 | NA | NA | 0.41034601 | 0.36928615 | NA | NA | NA | NA |
| NW vs TT | 1 | 0.35502885 | 0.42331387 | 0.31345551 | 0.74769373 | 0.52607066 | 0.24475672 | 0.39030648 | 0.50293701 | 0.65504922 | 0.76778506 | 0.44261489 |
| NW vs VR | 0 | 0 | 0.4097074 | 0.32330039 | NA | NA | 0.4097074 | 0.32330039 | NA | NA | NA | NA |
| NW vs WBV | 0 | 0 | 0.12461102 | 0.59709415 | NA | NA | 0.12461102 | 0.59709415 | NA | NA | NA | NA |
| NW vs Yoga | 0 | 0 | 0.54952956 | 0.37089545 | NA | NA | 0.54952956 | 0.37089545 | NA | NA | NA | NA |
| Pilates vs PT | 0 | 0 | 0.45331872 | 0.52639961 | NA | NA | 0.45331872 | 0.52639961 | NA | NA | NA | NA |
| Pilates vs Qigong | 0 | 0 | 0.60780223 | 0.38431225 | NA | NA | 0.60780223 | 0.38431225 | NA | NA | NA | NA |
| Pilates vs RA_GT | 0 | 0 | 0.22723733 | 0.37808797 | NA | NA | 0.22723733 | 0.37808797 | NA | NA | NA | NA |
| Pilates vs RT | 0 | 0 | 0.59425818 | 0.34417259 | NA | NA | 0.59425818 | 0.34417259 | NA | NA | NA | NA |
| Pilates vs Stretch | 0 | 0 | 0.8891082 | 0.35434119 | NA | NA | 0.8891082 | 0.35434119 | NA | NA | NA | NA |
| Pilates vs Tango | 0 | 0 | 0.37319551 | 0.36862697 | NA | NA | 0.37319551 | 0.36862697 | NA | NA | NA | NA |
| Pilates vs TC | 0 | 0 | 0.47568579 | 0.38160705 | NA | NA | 0.47568579 | 0.38160705 | NA | NA | NA | NA |
| Pilates vs TT | 0 | 0 | 0.48865366 | 0.34992516 | NA | NA | 0.48865366 | 0.34992516 | NA | NA | NA | NA |
| Pilates vs VR | 0 | 0 | 0.47504719 | 0.34268253 | NA | NA | 0.47504719 | 0.34268253 | NA | NA | NA | NA |
| Pilates vs WBV | 0 | 0 | 0.1899508 | 0.60765975 | NA | NA | 0.1899508 | 0.60765975 | NA | NA | NA | NA |
| Pilates vs Yoga | 0 | 0 | 0.61486935 | 0.38088909 | NA | NA | 0.61486935 | 0.38088909 | NA | NA | NA | NA |
| PT vs Qigong | 0 | 0 | 0.15448352 | 0.46591104 | NA | NA | 0.15448352 | 0.46591104 | NA | NA | NA | NA |
| PT vs RA_GT | 0 | 0 | -0.2260814 | 0.46512884 | NA | NA | -0.2260814 | 0.46512884 | NA | NA | NA | NA |
| PT vs RT | 0 | 0 | 0.14093947 | 0.436168 | NA | NA | 0.14093947 | 0.436168 | NA | NA | NA | NA |
| PT vs Stretch | 0 | 0 | 0.43578948 | 0.4441198 | NA | NA | 0.43578948 | 0.4441198 | NA | NA | NA | NA |
| PT vs Tango | 0 | 0 | -0.0801232 | 0.45385617 | NA | NA | -0.0801232 | 0.45385617 | NA | NA | NA | NA |
| PT vs TC | 0 | 0 | 0.02236707 | 0.45950902 | NA | NA | 0.02236707 | 0.45950902 | NA | NA | NA | NA |
| PT vs TT | 0 | 0 | 0.03533494 | 0.44052786 | NA | NA | 0.03533494 | 0.44052786 | NA | NA | NA | NA |
| PT vs VR | 0 | 0 | 0.02172847 | 0.43653727 | NA | NA | 0.02172847 | 0.43653727 | NA | NA | NA | NA |
| PT vs WBV | 0 | 0 | -0.2633679 | 0.66304303 | NA | NA | -0.2633679 | 0.66304303 | NA | NA | NA | NA |
| PT vs Yoga | 1 | 0.81639516 | 0.16155063 | 0.41089385 | 0.03131754 | 0.45475704 | 0.74062938 | 0.95893147 | -0.7093118 | 1.06129804 | -0.6683437 | 0.50391423 |
| Qigong vs RA_GT | 0 | 0 | -0.3805649 | 0.29406605 | NA | NA | -0.3805649 | 0.29406605 | NA | NA | NA | NA |
| Qigong vs RT | 0 | 0 | -0.013544 | 0.24047297 | NA | NA | -0.013544 | 0.24047297 | NA | NA | NA | NA |
| Qigong vs Stretch | 0 | 0 | 0.28130596 | 0.25704553 | NA | NA | 0.28130596 | 0.25704553 | NA | NA | NA | NA |
| Qigong vs Tango | 0 | 0 | -0.2346067 | 0.27272606 | NA | NA | -0.2346067 | 0.27272606 | NA | NA | NA | NA |
| Qigong vs TC | 0 | 0 | -0.1321164 | 0.29096713 | NA | NA | -0.1321164 | 0.29096713 | NA | NA | NA | NA |
| Qigong vs TT | 0 | 0 | -0.1191486 | 0.2507002 | NA | NA | -0.1191486 | 0.2507002 | NA | NA | NA | NA |
| Qigong vs VR | 0 | 0 | -0.132755 | 0.24277746 | NA | NA | -0.132755 | 0.24277746 | NA | NA | NA | NA |
| Qigong vs WBV | 0 | 0 | -0.4178514 | 0.55567188 | NA | NA | -0.4178514 | 0.55567188 | NA | NA | NA | NA |
| Qigong vs Yoga | 0 | 0 | 0.00706712 | 0.29451968 | NA | NA | 0.00706712 | 0.29451968 | NA | NA | NA | NA |
| RA_GT vs RT | 0 | 0 | 0.36702086 | 0.23516056 | NA | NA | 0.36702086 | 0.23516056 | NA | NA | NA | NA |
| RA_GT vs Stretch | 1 | 0.23126415 | 0.66187087 | 0.22500737 | 1.282423 | 0.46788874 | 0.47518586 | 0.25663045 | 0.80723714 | 0.53364694 | 1.51268015 | 0.13036092 |
| RA_GT vs Tango | 0 | 0 | 0.14595819 | 0.26748585 | NA | NA | 0.14595819 | 0.26748585 | NA | NA | NA | NA |
| RA_GT vs TC | 0 | 0 | 0.24844847 | 0.29087627 | NA | NA | 0.24844847 | 0.29087627 | NA | NA | NA | NA |
| RA_GT vs TT | 0 | 0 | 0.26141633 | 0.23644226 | NA | NA | 0.26141633 | 0.23644226 | NA | NA | NA | NA |
| RA_GT vs VR | 0 | 0 | 0.24780986 | 0.22691066 | NA | NA | 0.24780986 | 0.22691066 | NA | NA | NA | NA |
| RA_GT vs WBV | 0 | 0 | -0.0372865 | 0.5435229 | NA | NA | -0.0372865 | 0.5435229 | NA | NA | NA | NA |
| RA_GT vs Yoga | 0 | 0 | 0.38763202 | 0.28999662 | NA | NA | 0.38763202 | 0.28999662 | NA | NA | NA | NA |
| RT vs Stretch | 1 | 0.21468335 | 0.29485001 | 0.18212984 | 0.63688359 | 0.39308088 | 0.20134771 | 0.20552218 | 0.43553588 | 0.4435673 | 0.98189358 | 0.32615228 |
| RT vs Tango | 1 | 0.15290388 | -0.2210627 | 0.20910758 | -0.6844722 | 0.53476199 | -0.1374156 | 0.22719744 | -0.5470566 | 0.58102415 | -0.9415386 | 0.34642893 |
| RT vs TC | 0 | 0 | -0.1185724 | 0.24127873 | NA | NA | -0.1185724 | 0.24127873 | NA | NA | NA | NA |
| RT vs TT | 0 | 0 | -0.1056045 | 0.18322826 | NA | NA | -0.1056045 | 0.18322826 | NA | NA | NA | NA |
| RT vs VR | 0 | 0 | -0.119211 | 0.1704772 | NA | NA | -0.119211 | 0.1704772 | NA | NA | NA | NA |
| RT vs WBV | 0 | 0 | -0.4043074 | 0.52964807 | NA | NA | -0.4043074 | 0.52964807 | NA | NA | NA | NA |
| RT vs Yoga | 0 | 0 | 0.02061116 | 0.24240522 | NA | NA | 0.02061116 | 0.24240522 | NA | NA | NA | NA |
| Stretch vs Tango | 1 | 0.37054285 | -0.5159127 | 0.21341474 | -0.260824 | 0.35059457 | -0.6660758 | 0.26899321 | 0.40525183 | 0.44189807 | 0.91707083 | 0.35910553 |
| Stretch vs TC | 0 | 0 | -0.4134224 | 0.25518252 | NA | NA | -0.4134224 | 0.25518252 | NA | NA | NA | NA |
| Stretch vs TT | 1 | 0.25056161 | -0.4004545 | 0.18015589 | -0.260824 | 0.35990776 | -0.4471376 | 0.20810404 | 0.18631354 | 0.41574137 | 0.44814771 | 0.6540466 |
| Stretch vs VR | 2 | 0.28055699 | -0.414061 | 0.16919632 | -0.0543396 | 0.31943343 | -0.5543395 | 0.19947695 | 0.49999988 | 0.3766016 | 1.32766264 | 0.18428958 |
| Stretch vs WBV | 0 | 0 | -0.6991574 | 0.52597748 | NA | NA | -0.6991574 | 0.52597748 | NA | NA | NA | NA |
| Stretch vs Yoga | 0 | 0 | -0.2742388 | 0.25652207 | NA | NA | -0.2742388 | 0.25652207 | NA | NA | NA | NA |
| Tango vs TC | 0 | 0 | 0.10249028 | 0.27175309 | NA | NA | 0.10249028 | 0.27175309 | NA | NA | NA | NA |
| Tango vs TT | 1 | 0.37192535 | 0.11545814 | 0.20801053 | 0 | 0.34108093 | 0.18382869 | 0.26247002 | -0.1838287 | 0.43037973 | -0.4271314 | 0.66928363 |
| Tango vs VR | 0 | 0 | 0.10185167 | 0.21217037 | NA | NA | 0.10185167 | 0.21217037 | NA | NA | NA | NA |
| Tango vs WBV | 0 | 0 | -0.1832447 | 0.54277964 | NA | NA | -0.1832447 | 0.54277964 | NA | NA | NA | NA |
| Tango vs Yoga | 0 | 0 | 0.24167383 | 0.27444626 | NA | NA | 0.24167383 | 0.27444626 | NA | NA | NA | NA |
| TC vs TT | 0 | 0 | 0.01296786 | 0.24807616 | NA | NA | 0.01296786 | 0.24807616 | NA | NA | NA | NA |
| TC vs VR | 0 | 0 | -0.0006386 | 0.24194535 | NA | NA | -0.0006386 | 0.24194535 | NA | NA | NA | NA |
| TC vs WBV | 0 | 0 | -0.285735 | 0.55464748 | NA | NA | -0.285735 | 0.55464748 | NA | NA | NA | NA |
| TC vs Yoga | 1 | 0.26489591 | 0.13918356 | 0.27758993 | 0.65962242 | 0.53934428 | -0.0483574 | 0.32376454 | 0.70797982 | 0.6290594 | 1.12545782 | 0.26039508 |
| TT vs VR | 2 | 0.40257295 | -0.0136065 | 0.15634868 | -0.1025979 | 0.24641771 | 0.04635995 | 0.20227945 | -0.1489579 | 0.31880819 | -0.4672335 | 0.64033282 |
| TT vs WBV | 0 | 0 | -0.2987029 | 0.52850316 | NA | NA | -0.2987029 | 0.52850316 | NA | NA | NA | NA |
| TT vs Yoga | 0 | 0 | 0.12621569 | 0.25026067 | NA | NA | 0.12621569 | 0.25026067 | NA | NA | NA | NA |
| VR vs WBV | 0 | 0 | -0.2850964 | 0.52614723 | NA | NA | -0.2850964 | 0.52614723 | NA | NA | NA | NA |
| VR vs Yoga | 0 | 0 | 0.13982216 | 0.24171187 | NA | NA | 0.13982216 | 0.24171187 | NA | NA | NA | NA |
| WBV vs Yoga | 0 | 0 | 0.42491855 | 0.55573076 | NA | NA | 0.42491855 | 0.55573076 | NA | NA | NA | NA |

*NA* not available, *k* Number of studies providing direct evidence, *prop* Direct evidence proportion, *nma* Estimated treatment effect (SMD) in network meta-analysis, *direct* Estimated treatment effect (SMD) derived from direct evidence, *indir.* Estimated treatment effect (SMD) derived from indirect evidence, *Diff* Difference between direct and indirect treatment estimates, *z* z-value of test for disagreement (direct versus indirect), *p* p-value of test for disagreement (direct versus indirect)

## Table 7.2 Details of SIDE splitting results (Static steady-state balance)

| **Comparison** | **k** | **prop** | **NMA** | | **Direc** | | **Indir** | | **Diff** | | **z** | **p** |
| --- | --- | --- | --- | --- | --- | --- | --- | --- | --- | --- | --- | --- |
|  |  |  | **TE** | **seTE** | **TE** | **seTE** | **TE** | **seTE** | **TE** | **seTE** |  |  |
| AE vs AQE | 1 | 0.45201313 | -0.5291267 | 0.40210682 | -0.3265523 | 0.59808913 | -0.6962225 | 0.54319575 | 0.36967017 | 0.80794321 | 0.45754474 | 0.64727955 |
| AE vs BGT | 0 | 0 | -0.2910263 | 0.39439729 | NA | NA | -0.2910263 | 0.39439729 | NA | NA | NA | NA |
| AE vs BGT_ECA | 0 | 0 | -0.0733129 | 0.43180451 | NA | NA | -0.0733129 | 0.43180451 | NA | NA | NA | NA |
| AE vs BGT_ICA | 0 | 0 | 0.00867572 | 0.53541381 | NA | NA | 0.00867572 | 0.53541381 | NA | NA | NA | NA |
| AE vs CON | 1 | 0.57501398 | 0.40623874 | 0.36843336 | 0.16016047 | 0.48586961 | 0.7391872 | 0.56516026 | -0.5790267 | 0.74530222 | -0.7769019 | 0.43721664 |
| AE vs CPP | 0 | 0 | 0.5692161 | 0.44887099 | NA | NA | 0.5692161 | 0.44887099 | NA | NA | NA | NA |
| AE vs DT_BGT | 0 | 0 | -0.1084737 | 0.41122025 | NA | NA | -0.1084737 | 0.41122025 | NA | NA | NA | NA |
| AE vs Mul_C | 0 | 0 | -0.3999594 | 0.37967566 | NA | NA | -0.3999594 | 0.37967566 | NA | NA | NA | NA |
| AE vs Mul_D | 0 | 0 | 0.16658021 | 0.68073716 | NA | NA | 0.16658021 | 0.68073716 | NA | NA | NA | NA |
| AE vs NW | 1 | 1 | -0.5254894 | 0.56865617 | -0.5254894 | 0.56865617 | NA | NA | NA | NA | NA | NA |
| AE vs PT | 0 | 0 | 0.16714574 | 0.51820162 | NA | NA | 0.16714574 | 0.51820162 | NA | NA | NA | NA |
| AE vs Qigong | 0 | 0 | -0.1668127 | 0.51437422 | NA | NA | -0.1668127 | 0.51437422 | NA | NA | NA | NA |
| AE vs RA_GT | 0 | 0 | 0.09759282 | 0.75032586 | NA | NA | 0.09759282 | 0.75032586 | NA | NA | NA | NA |
| AE vs RT | 0 | 0 | 0.14555099 | 0.40602315 | NA | NA | 0.14555099 | 0.40602315 | NA | NA | NA | NA |
| AE vs Stretch | 0 | 0 | 0.47794256 | 0.43369708 | NA | NA | 0.47794256 | 0.43369708 | NA | NA | NA | NA |
| AE vs Tai Chi | 0 | 0 | -0.1540166 | 0.46687526 | NA | NA | -0.1540166 | 0.46687526 | NA | NA | NA | NA |
| AE vs TT | 0 | 0 | 0.45273314 | 0.45127297 | NA | NA | 0.45273314 | 0.45127297 | NA | NA | NA | NA |
| AE vs VR | 1 | 0.6138905 | -0.4274901 | 0.38003993 | -0.3819211 | 0.48504695 | -0.499942 | 0.61160917 | 0.11802091 | 0.78059998 | 0.15119257 | 0.87982381 |
| AE vs WBV | 0 | 0 | 0.97606005 | 0.7418838 | NA | NA | 0.97606005 | 0.7418838 | NA | NA | NA | NA |
| AE vs Yoga | 0 | 0 | -0.045148 | 0.53224345 | NA | NA | -0.045148 | 0.53224345 | NA | NA | NA | NA |
| AQE vs BGT | 1 | 0.3805306 | 0.23810035 | 0.32259057 | 0.30675283 | 0.52294605 | 0.19592817 | 0.40986586 | 0.11082466 | 0.66442651 | 0.16679746 | 0.86752942 |
| AQE vs BGT_ECA | 0 | 0 | 0.4558138 | 0.37664139 | NA | NA | 0.4558138 | 0.37664139 | NA | NA | NA | NA |
| AQE vs BGT_ICA | 0 | 0 | 0.5378024 | 0.49182774 | NA | NA | 0.5378024 | 0.49182774 | NA | NA | NA | NA |
| AQE vs CON | 0 | 0 | 0.93536542 | 0.30879029 | NA | NA | 0.93536542 | 0.30879029 | NA | NA | NA | NA |
| AQE vs CPP | 1 | 0.36683794 | 1.09834279 | 0.36248784 | 1.7877859 | 0.59848921 | 0.69889705 | 0.45554995 | 1.08888885 | 0.75214034 | 1.44772032 | 0.14769528 |
| AQE vs DT_BGT | 0 | 0 | 0.42065294 | 0.36111761 | NA | NA | 0.42065294 | 0.36111761 | NA | NA | NA | NA |
| AQE vs Mul_C | 1 | 0.36857482 | 0.1291673 | 0.30677887 | -0.2818168 | 0.5053155 | 0.36906647 | 0.38606862 | -0.6508832 | 0.6359188 | -1.023532 | 0.30605639 |
| AQE vs Mul_D | 0 | 0 | 0.6957069 | 0.62714859 | NA | NA | 0.6957069 | 0.62714859 | NA | NA | NA | NA |
| AQE vs NW | 0 | 0 | 0.00363734 | 0.6964623 | NA | NA | 0.00363734 | 0.6964623 | NA | NA | NA | NA |
| AQE vs PT | 0 | 0 | 0.69627243 | 0.47764131 | NA | NA | 0.69627243 | 0.47764131 | NA | NA | NA | NA |
| AQE vs Qigong | 0 | 0 | 0.36231398 | 0.47348616 | NA | NA | 0.36231398 | 0.47348616 | NA | NA | NA | NA |
| AQE vs RA_GT | 0 | 0 | 0.62671951 | 0.7020692 | NA | NA | 0.62671951 | 0.7020692 | NA | NA | NA | NA |
| AQE vs RT | 0 | 0 | 0.67467768 | 0.3543554 | NA | NA | 0.67467768 | 0.3543554 | NA | NA | NA | NA |
| AQE vs Stretch | 0 | 0 | 1.00706925 | 0.38476339 | NA | NA | 1.00706925 | 0.38476339 | NA | NA | NA | NA |
| AQE vs Tai Chi | 0 | 0 | 0.37511011 | 0.42187945 | NA | NA | 0.37511011 | 0.42187945 | NA | NA | NA | NA |
| AQE vs TT | 0 | 0 | 0.98185983 | 0.40004235 | NA | NA | 0.98185983 | 0.40004235 | NA | NA | NA | NA |
| AQE vs VR | 0 | 0 | 0.10163657 | 0.34927206 | NA | NA | 0.10163657 | 0.34927206 | NA | NA | NA | NA |
| AQE vs WBV | 0 | 0 | 1.50518674 | 0.71532888 | NA | NA | 1.50518674 | 0.71532888 | NA | NA | NA | NA |
| AQE vs Yoga | 0 | 0 | 0.48397868 | 0.49284013 | NA | NA | 0.48397868 | 0.49284013 | NA | NA | NA | NA |
| BGT vs BGT_ECA | 2 | 0.46590358 | 0.21771346 | 0.26371982 | 0.18099562 | 0.38636231 | 0.2497432 | 0.36085517 | -0.0687476 | 0.5286703 | -0.1300387 | 0.89653583 |
| BGT vs BGT_ICA | 1 | 0.47799665 | 0.29970206 | 0.40886696 | 0.48250469 | 0.59138403 | 0.13231032 | 0.5659074 | 0.35019437 | 0.81852688 | 0.4278349 | 0.66877132 |
| BGT vs CON | 3 | 0.30730977 | 0.69726508 | 0.19662418 | 0.39303833 | 0.35468983 | 0.83223429 | 0.23624759 | -0.439196 | 0.4261664 | -1.0305739 | 0.30274069 |
| BGT vs CPP | 0 | 0 | 0.86024244 | 0.33611472 | NA | NA | 0.86024244 | 0.33611472 | NA | NA | NA | NA |
| BGT vs DT_BGT | 1 | 0.14930499 | 0.18255259 | 0.25957225 | 0.77444955 | 0.67177076 | 0.0786691 | 0.2814306 | 0.69578045 | 0.72833999 | 0.95529624 | 0.33942791 |
| BGT vs Mul_C | 1 | 0.10864469 | -0.108933 | 0.21865384 | -0.4068363 | 0.66336547 | -0.0726225 | 0.23159633 | -0.3342138 | 0.7026312 | -0.4756603 | 0.63431642 |
| BGT vs Mul_D | 0 | 0 | 0.45760655 | 0.61228345 | NA | NA | 0.45760655 | 0.61228345 | NA | NA | NA | NA |
| BGT vs NW | 0 | 0 | -0.234463 | 0.69203978 | NA | NA | -0.234463 | 0.69203978 | NA | NA | NA | NA |
| BGT vs PT | 0 | 0 | 0.45817208 | 0.41406623 | NA | NA | 0.45817208 | 0.41406623 | NA | NA | NA | NA |
| BGT vs Qigong | 0 | 0 | 0.12421364 | 0.40926614 | NA | NA | 0.12421364 | 0.40926614 | NA | NA | NA | NA |
| BGT vs RA_GT | 0 | 0 | 0.38861916 | 0.68882279 | NA | NA | 0.38861916 | 0.68882279 | NA | NA | NA | NA |
| BGT vs RT | 1 | 0.22640647 | 0.43657733 | 0.24708457 | 1.0347018 | 0.51927953 | 0.26152513 | 0.28092423 | 0.77317667 | 0.59039788 | 1.30958579 | 0.190336 |
| BGT vs Stretch | 0 | 0 | 0.7689689 | 0.29391843 | NA | NA | 0.7689689 | 0.29391843 | NA | NA | NA | NA |
| BGT vs Tai Chi | 0 | 0 | 0.13700976 | 0.34314236 | NA | NA | 0.13700976 | 0.34314236 | NA | NA | NA | NA |
| BGT vs TT | 0 | 0 | 0.74375948 | 0.31541427 | NA | NA | 0.74375948 | 0.31541427 | NA | NA | NA | NA |
| BGT vs VR | 2 | 0.36986242 | -0.1364638 | 0.2405733 | -0.1240322 | 0.39557362 | -0.1437606 | 0.30306078 | 0.01972839 | 0.4983215 | 0.03958968 | 0.96842025 |
| BGT vs WBV | 0 | 0 | 1.26708639 | 0.66982627 | NA | NA | 1.26708639 | 0.66982627 | NA | NA | NA | NA |
| BGT vs Yoga | 0 | 0 | 0.24587833 | 0.43151017 | NA | NA | 0.24587833 | 0.43151017 | NA | NA | NA | NA |
| BGT_ECA vs BGT_ICA | 1 | 0.50690482 | 0.0819886 | 0.41842147 | -0.1139247 | 0.58769331 | 0.28338867 | 0.59586597 | -0.3973134 | 0.83692275 | -0.4747313 | 0.63497853 |
| BGT_ECA vs CON | 2 | 0.48786969 | 0.47955162 | 0.2417457 | 0.18240841 | 0.34610418 | 0.76261857 | 0.33780691 | -0.5802102 | 0.48363376 | -1.1996891 | 0.23026009 |
| BGT_ECA vs CPP | 0 | 0 | 0.64252899 | 0.37107112 | NA | NA | 0.64252899 | 0.37107112 | NA | NA | NA | NA |
| BGT_ECA vs DT_BGT | 0 | 0 | -0.0351609 | 0.30489255 | NA | NA | -0.0351609 | 0.30489255 | NA | NA | NA | NA |
| BGT_ECA vs Mul_C | 0 | 0 | -0.3266465 | 0.26493606 | NA | NA | -0.3266465 | 0.26493606 | NA | NA | NA | NA |
| BGT_ECA vs Mul_D | 0 | 0 | 0.2398931 | 0.63214847 | NA | NA | 0.2398931 | 0.63214847 | NA | NA | NA | NA |
| BGT_ECA vs NW | 0 | 0 | -0.4521765 | 0.71402029 | NA | NA | -0.4521765 | 0.71402029 | NA | NA | NA | NA |
| BGT_ECA vs PT | 0 | 0 | 0.24045862 | 0.4372994 | NA | NA | 0.24045862 | 0.4372994 | NA | NA | NA | NA |
| BGT_ECA vs Qigong | 0 | 0 | -0.0934998 | 0.43275708 | NA | NA | -0.0934998 | 0.43275708 | NA | NA | NA | NA |
| BGT_ECA vs RA_GT | 0 | 0 | 0.17090571 | 0.7065391 | NA | NA | 0.17090571 | 0.7065391 | NA | NA | NA | NA |
| BGT_ECA vs RT | 0 | 0 | 0.21886387 | 0.30064604 | NA | NA | 0.21886387 | 0.30064604 | NA | NA | NA | NA |
| BGT_ECA vs Stretch | 0 | 0 | 0.55125544 | 0.3357024 | NA | NA | 0.55125544 | 0.3357024 | NA | NA | NA | NA |
| BGT_ECA vs Tai Chi | 0 | 0 | -0.0807037 | 0.37680771 | NA | NA | -0.0807037 | 0.37680771 | NA | NA | NA | NA |
| BGT_ECA vs TT | 2 | 0.44833865 | 0.52604602 | 0.3063055 | 0.9973147 | 0.45745851 | 0.14304301 | 0.41239987 | 0.85427169 | 0.61590741 | 1.38701317 | 0.16543775 |
| BGT_ECA vs VR | 0 | 0 | -0.3541772 | 0.30507549 | NA | NA | -0.3541772 | 0.30507549 | NA | NA | NA | NA |
| BGT_ECA vs WBV | 0 | 0 | 1.04937293 | 0.68865735 | NA | NA | 1.04937293 | 0.68865735 | NA | NA | NA | NA |
| BGT_ECA vs Yoga | 0 | 0 | 0.02816487 | 0.45385123 | NA | NA | 0.02816487 | 0.45385123 | NA | NA | NA | NA |
| BGT_ICA vs CON | 1 | 0.45371341 | 0.39756302 | 0.39817367 | 0.38555946 | 0.59112827 | 0.40753247 | 0.53871897 | -0.021973 | 0.79978169 | -0.0274738 | 0.97808187 |
| BGT_ICA vs CPP | 0 | 0 | 0.56054039 | 0.48833925 | NA | NA | 0.56054039 | 0.48833925 | NA | NA | NA | NA |
| BGT_ICA vs DT_BGT | 0 | 0 | -0.1171495 | 0.44363197 | NA | NA | -0.1171495 | 0.44363197 | NA | NA | NA | NA |
| BGT_ICA vs Mul_C | 0 | 0 | -0.4086351 | 0.41451279 | NA | NA | -0.4086351 | 0.41451279 | NA | NA | NA | NA |
| BGT_ICA vs Mul_D | 0 | 0 | 0.1579045 | 0.70738471 | NA | NA | 0.1579045 | 0.70738471 | NA | NA | NA | NA |
| BGT_ICA vs NW | 0 | 0 | -0.5341651 | 0.78104916 | NA | NA | -0.5341651 | 0.78104916 | NA | NA | NA | NA |
| BGT_ICA vs PT | 0 | 0 | 0.15847003 | 0.53975184 | NA | NA | 0.15847003 | 0.53975184 | NA | NA | NA | NA |
| BGT_ICA vs Qigong | 0 | 0 | -0.1754884 | 0.53607833 | NA | NA | -0.1754884 | 0.53607833 | NA | NA | NA | NA |
| BGT_ICA vs RA_GT | 0 | 0 | 0.08891711 | 0.77458308 | NA | NA | 0.08891711 | 0.77458308 | NA | NA | NA | NA |
| BGT_ICA vs RT | 0 | 0 | 0.13687527 | 0.43680131 | NA | NA | 0.13687527 | 0.43680131 | NA | NA | NA | NA |
| BGT_ICA vs Stretch | 0 | 0 | 0.46926685 | 0.4634577 | NA | NA | 0.46926685 | 0.4634577 | NA | NA | NA | NA |
| BGT_ICA vs Tai Chi | 0 | 0 | -0.1626923 | 0.49266614 | NA | NA | -0.1626923 | 0.49266614 | NA | NA | NA | NA |
| BGT_ICA vs TT | 0 | 0 | 0.44405743 | 0.46724822 | NA | NA | 0.44405743 | 0.46724822 | NA | NA | NA | NA |
| BGT_ICA vs VR | 0 | 0 | -0.4361658 | 0.43996412 | NA | NA | -0.4361658 | 0.43996412 | NA | NA | NA | NA |
| BGT_ICA vs WBV | 0 | 0 | 0.96738434 | 0.76032809 | NA | NA | 0.96738434 | 0.76032809 | NA | NA | NA | NA |
| BGT_ICA vs Yoga | 0 | 0 | -0.0538237 | 0.55324699 | NA | NA | -0.0538237 | 0.55324699 | NA | NA | NA | NA |
| CON vs CPP | 1 | 0.37983079 | -0.1629774 | 0.29012468 | 0.45349018 | 0.4707492 | -0.540541 | 0.36840844 | 0.9940312 | 0.59777052 | 1.66289768 | 0.09633292 |
| CON vs DT_BGT | 2 | 0.33642733 | 0.51471249 | 0.21737635 | 0.36350711 | 0.37477159 | 0.59137269 | 0.26685051 | -0.2278656 | 0.46006841 | -0.4952863 | 0.62039803 |
| CON vs Mul_C | 12 | 0.78746637 | 0.80619812 | 0.1307697 | 0.84616738 | 0.14736391 | 0.65810653 | 0.28365687 | 0.18806084 | 0.31965191 | 0.58833012 | 0.55631073 |
| CON vs Mul_D | 0 | 0 | 0.23965852 | 0.58829435 | NA | NA | 0.23965852 | 0.58829435 | NA | NA | NA | NA |
| CON vs NW | 0 | 0 | 0.93172809 | 0.67757876 | NA | NA | 0.93172809 | 0.67757876 | NA | NA | NA | NA |
| CON vs PT | 2 | 0.92614799 | 0.239093 | 0.36440332 | 0.28014559 | 0.37865363 | -0.2757309 | 1.34091477 | 0.55587652 | 1.39335243 | 0.39894897 | 0.6899308 |
| CON vs Qigong | 2 | 1 | 0.57305144 | 0.35893969 | 0.57305144 | 0.35893969 | NA | NA | NA | NA | NA | NA |
| CON vs RA_GT | 0 | 0 | 0.30864591 | 0.66758974 | NA | NA | 0.30864591 | 0.66758974 | NA | NA | NA | NA |
| CON vs RT | 4 | 0.53731255 | 0.26068775 | 0.1976922 | 0.43613266 | 0.26969695 | 0.05694599 | 0.2906335 | 0.37918667 | 0.39648994 | 0.95635887 | 0.33889096 |
| CON vs Stretch | 0 | 0 | -0.0717038 | 0.25167909 | NA | NA | -0.0717038 | 0.25167909 | NA | NA | NA | NA |
| CON vs Tai Chi | 2 | 0.50898658 | 0.56025532 | 0.2966999 | 0.50095529 | 0.41587635 | 0.62172597 | 0.42341936 | -0.1207707 | 0.59349566 | -0.2034904 | 0.83875173 |
| CON vs TT | 1 | 0.23597761 | -0.0464944 | 0.27540262 | -0.1465889 | 0.56693422 | -0.015579 | 0.31507579 | -0.1310099 | 0.64860401 | -0.2019875 | 0.83992652 |
| CON vs VR | 3 | 0.4619315 | 0.83372885 | 0.21426307 | 0.94539484 | 0.3152525 | 0.73786366 | 0.29209798 | 0.20753118 | 0.42977362 | 0.48288487 | 0.62917748 |
| CON vs WBV | 0 | 0 | -0.5698213 | 0.65463116 | NA | NA | -0.5698213 | 0.65463116 | NA | NA | NA | NA |
| CON vs Yoga | 2 | 0.91276782 | 0.45138675 | 0.3841093 | 0.46510097 | 0.402045 | 0.30788577 | 1.30051828 | 0.1572152 | 1.36124501 | 0.11549369 | 0.90805382 |
| CPP vs DT_BGT | 0 | 0 | -0.6776899 | 0.35324259 | NA | NA | -0.6776899 | 0.35324259 | NA | NA | NA | NA |
| CPP vs Mul_C | 2 | 0.49743663 | -0.9691755 | 0.2862167 | -1.1103152 | 0.40581312 | -0.8294756 | 0.40373793 | -0.2808396 | 0.57244092 | -0.4906002 | 0.62370922 |
| CPP vs Mul_D | 1 | 1 | -0.4026359 | 0.51177916 | -0.4026359 | 0.51177916 | NA | NA | NA | NA | NA | NA |
| CPP vs NW | 0 | 0 | -1.0947055 | 0.72446877 | NA | NA | -1.0947055 | 0.72446877 | NA | NA | NA | NA |
| CPP vs PT | 0 | 0 | -0.4020704 | 0.46579191 | NA | NA | -0.4020704 | 0.46579191 | NA | NA | NA | NA |
| CPP vs Qigong | 0 | 0 | -0.7360288 | 0.4615301 | NA | NA | -0.7360288 | 0.4615301 | NA | NA | NA | NA |
| CPP vs RA_GT | 1 | 1 | -0.4716233 | 0.6012518 | -0.4716233 | 0.6012518 | NA | NA | NA | NA | NA | NA |
| CPP vs RT | 0 | 0 | -0.4236651 | 0.3455792 | NA | NA | -0.4236651 | 0.3455792 | NA | NA | NA | NA |
| CPP vs Stretch | 0 | 0 | -0.0912735 | 0.37583464 | NA | NA | -0.0912735 | 0.37583464 | NA | NA | NA | NA |
| CPP vs Tai Chi | 0 | 0 | -0.7232327 | 0.41159812 | NA | NA | -0.7232327 | 0.41159812 | NA | NA | NA | NA |
| CPP vs TT | 0 | 0 | -0.116483 | 0.39005983 | NA | NA | -0.116483 | 0.39005983 | NA | NA | NA | NA |
| CPP vs VR | 0 | 0 | -0.9967062 | 0.3499938 | NA | NA | -0.9967062 | 0.3499938 | NA | NA | NA | NA |
| CPP vs WBV | 0 | 0 | 0.40684395 | 0.71138584 | NA | NA | 0.40684395 | 0.71138584 | NA | NA | NA | NA |
| CPP vs Yoga | 0 | 0 | -0.6143641 | 0.48136502 | NA | NA | -0.6143641 | 0.48136502 | NA | NA | NA | NA |
| DT_BGT vs Mul_C | 1 | 0.16754265 | -0.2914856 | 0.23521105 | 0.75122926 | 0.57463891 | -0.5013453 | 0.25779633 | 1.25257456 | 0.6298165 | 1.98879288 | 0.04672407 |
| DT_BGT vs Mul_D | 0 | 0 | 0.27505396 | 0.62185066 | NA | NA | 0.27505396 | 0.62185066 | NA | NA | NA | NA |
| DT_BGT vs NW | 0 | 0 | -0.4170156 | 0.70176344 | NA | NA | -0.4170156 | 0.70176344 | NA | NA | NA | NA |
| DT_BGT vs PT | 0 | 0 | 0.27561949 | 0.42431386 | NA | NA | 0.27561949 | 0.42431386 | NA | NA | NA | NA |
| DT_BGT vs Qigong | 0 | 0 | -0.058339 | 0.419631 | NA | NA | -0.058339 | 0.419631 | NA | NA | NA | NA |
| DT_BGT vs RA_GT | 0 | 0 | 0.20606657 | 0.69734071 | NA | NA | 0.20606657 | 0.69734071 | NA | NA | NA | NA |
| DT_BGT vs RT | 0 | 0 | 0.25402474 | 0.25716353 | NA | NA | 0.25402474 | 0.25716353 | NA | NA | NA | NA |
| DT_BGT vs Stretch | 3 | 0.68471702 | 0.58641631 | 0.24129478 | 0.73984144 | 0.29160325 | 0.2532147 | 0.42973196 | 0.48662674 | 0.51932842 | 0.93703083 | 0.3487427 |
| DT_BGT vs Tai Chi | 0 | 0 | -0.0455428 | 0.33947365 | NA | NA | -0.0455428 | 0.33947365 | NA | NA | NA | NA |
| DT_BGT vs TT | 1 | 0.360043 | 0.56120689 | 0.30582583 | 0 | 0.50967928 | 0.87694469 | 0.38229513 | -0.8769447 | 0.6371205 | -1.3764189 | 0.16869199 |
| DT_BGT vs VR | 2 | 0.4461971 | -0.3190164 | 0.25259171 | -0.0665415 | 0.3781427 | -0.5224345 | 0.33942316 | 0.45589293 | 0.50813382 | 0.89719069 | 0.36961717 |
| DT_BGT vs WBV | 1 | 1 | 1.0845338 | 0.61748642 | 1.0845338 | 0.61748642 | NA | NA | NA | NA | NA | NA |
| DT_BGT vs Yoga | 0 | 0 | 0.06332574 | 0.44135296 | NA | NA | 0.06332574 | 0.44135296 | NA | NA | NA | NA |
| Mul_C vs Mul_D | 0 | 0 | 0.5665396 | 0.58637694 | NA | NA | 0.5665396 | 0.58637694 | NA | NA | NA | NA |
| Mul_C vs NW | 0 | 0 | -0.12553 | 0.68375686 | NA | NA | -0.12553 | 0.68375686 | NA | NA | NA | NA |
| Mul_C vs PT | 0 | 0 | 0.56710513 | 0.38715694 | NA | NA | 0.56710513 | 0.38715694 | NA | NA | NA | NA |
| Mul_C vs Qigong | 0 | 0 | 0.23314668 | 0.38201887 | NA | NA | 0.23314668 | 0.38201887 | NA | NA | NA | NA |
| Mul_C vs RA_GT | 0 | 0 | 0.49755221 | 0.66590069 | NA | NA | 0.49755221 | 0.66590069 | NA | NA | NA | NA |
| Mul_C vs RT | 0 | 0 | 0.54551037 | 0.22764671 | NA | NA | 0.54551037 | 0.22764671 | NA | NA | NA | NA |
| Mul_C vs Stretch | 1 | 0.2148702 | 0.87790195 | 0.26747554 | 0.85484708 | 0.5770268 | 0.88421148 | 0.30186541 | -0.0293644 | 0.65121629 | -0.0450916 | 0.96403427 |
| Mul_C vs Tai Chi | 0 | 0 | 0.24594281 | 0.31854017 | NA | NA | 0.24594281 | 0.31854017 | NA | NA | NA | NA |
| Mul_C vs TT | 1 | 0.3047079 | 0.85269253 | 0.28414494 | 0.74590246 | 0.51475203 | 0.89949268 | 0.34076601 | -0.1535902 | 0.61732578 | -0.2487993 | 0.80351603 |
| Mul_C vs VR | 1 | 0.16339517 | -0.0275307 | 0.23485932 | -1.0216068 | 0.58101613 | 0.16661975 | 0.25677197 | -1.1882265 | 0.63522561 | -1.8705583 | 0.06140633 |
| Mul_C vs WBV | 0 | 0 | 1.37601944 | 0.66076752 | NA | NA | 1.37601944 | 0.66076752 | NA | NA | NA | NA |
| Mul_C vs Yoga | 0 | 0 | 0.35481138 | 0.40575938 | NA | NA | 0.35481138 | 0.40575938 | NA | NA | NA | NA |
| Mul_D vs NW | 0 | 0 | -0.6920696 | 0.88700221 | NA | NA | -0.6920696 | 0.88700221 | NA | NA | NA | NA |
| Mul_D vs PT | 0 | 0 | 0.00056553 | 0.69201158 | NA | NA | 0.00056553 | 0.69201158 | NA | NA | NA | NA |
| Mul_D vs Qigong | 0 | 0 | -0.3333929 | 0.68915016 | NA | NA | -0.3333929 | 0.68915016 | NA | NA | NA | NA |
| Mul_D vs RA_GT | 0 | 0 | -0.0689874 | 0.78957055 | NA | NA | -0.0689874 | 0.78957055 | NA | NA | NA | NA |
| Mul_D vs RT | 0 | 0 | -0.0210292 | 0.61752968 | NA | NA | -0.0210292 | 0.61752968 | NA | NA | NA | NA |
| Mul_D vs Stretch | 0 | 0 | 0.31136235 | 0.63495637 | NA | NA | 0.31136235 | 0.63495637 | NA | NA | NA | NA |
| Mul_D vs Tai Chi | 0 | 0 | -0.3205968 | 0.65675789 | NA | NA | -0.3205968 | 0.65675789 | NA | NA | NA | NA |
| Mul_D vs TT | 0 | 0 | 0.28615293 | 0.6434785 | NA | NA | 0.28615293 | 0.6434785 | NA | NA | NA | NA |
| Mul_D vs VR | 0 | 0 | -0.5940703 | 0.62001095 | NA | NA | -0.5940703 | 0.62001095 | NA | NA | NA | NA |
| Mul_D vs WBV | 0 | 0 | 0.80947984 | 0.87634909 | NA | NA | 0.80947984 | 0.87634909 | NA | NA | NA | NA |
| Mul_D vs Yoga | 0 | 0 | -0.2117282 | 0.70258822 | NA | NA | -0.2117282 | 0.70258822 | NA | NA | NA | NA |
| NW vs PT | 0 | 0 | 0.69263509 | 0.76935216 | NA | NA | 0.69263509 | 0.76935216 | NA | NA | NA | NA |
| NW vs Qigong | 0 | 0 | 0.35867664 | 0.76677942 | NA | NA | 0.35867664 | 0.76677942 | NA | NA | NA | NA |
| NW vs RA_GT | 0 | 0 | 0.62308217 | 0.94146627 | NA | NA | 0.62308217 | 0.94146627 | NA | NA | NA | NA |
| NW vs RT | 0 | 0 | 0.67104034 | 0.69873073 | NA | NA | 0.67104034 | 0.69873073 | NA | NA | NA | NA |
| NW vs Stretch | 0 | 0 | 1.00343191 | 0.71516641 | NA | NA | 1.00343191 | 0.71516641 | NA | NA | NA | NA |
| NW vs Tai Chi | 0 | 0 | 0.37147277 | 0.73575971 | NA | NA | 0.37147277 | 0.73575971 | NA | NA | NA | NA |
| NW vs TT | 0 | 0 | 0.97822249 | 0.72595946 | NA | NA | 0.97822249 | 0.72595946 | NA | NA | NA | NA |
| NW vs VR | 0 | 0 | 0.09799923 | 0.6839592 | NA | NA | 0.09799923 | 0.6839592 | NA | NA | NA | NA |
| NW vs WBV | 0 | 0 | 1.5015494 | 0.93475206 | NA | NA | 1.5015494 | 0.93475206 | NA | NA | NA | NA |
| NW vs Yoga | 0 | 0 | 0.48034134 | 0.77887928 | NA | NA | 0.48034134 | 0.77887928 | NA | NA | NA | NA |
| PT vs Qigong | 0 | 0 | -0.3339584 | 0.51149534 | NA | NA | -0.3339584 | 0.51149534 | NA | NA | NA | NA |
| PT vs RA_GT | 0 | 0 | -0.0695529 | 0.76056942 | NA | NA | -0.0695529 | 0.76056942 | NA | NA | NA | NA |
| PT vs RT | 0 | 0 | -0.0215948 | 0.41457446 | NA | NA | -0.0215948 | 0.41457446 | NA | NA | NA | NA |
| PT vs Stretch | 0 | 0 | 0.31079682 | 0.44286808 | NA | NA | 0.31079682 | 0.44286808 | NA | NA | NA | NA |
| PT vs Tai Chi | 0 | 0 | -0.3211623 | 0.46991553 | NA | NA | -0.3211623 | 0.46991553 | NA | NA | NA | NA |
| PT vs TT | 0 | 0 | 0.2855874 | 0.45676732 | NA | NA | 0.2855874 | 0.45676732 | NA | NA | NA | NA |
| PT vs VR | 0 | 0 | -0.5946359 | 0.42272739 | NA | NA | -0.5946359 | 0.42272739 | NA | NA | NA | NA |
| PT vs WBV | 0 | 0 | 0.80891431 | 0.74922075 | NA | NA | 0.80891431 | 0.74922075 | NA | NA | NA | NA |
| PT vs Yoga | 1 | 0.6499391 | -0.2122938 | 0.44428352 | -0.2703553 | 0.5510917 | -0.1044941 | 0.7509109 | -0.1658612 | 0.93143397 | -0.1780708 | 0.85866737 |
| Qigong vs RA_GT | 0 | 0 | 0.26440553 | 0.75796686 | NA | NA | 0.26440553 | 0.75796686 | NA | NA | NA | NA |
| Qigong vs RT | 0 | 0 | 0.31236369 | 0.40978032 | NA | NA | 0.31236369 | 0.40978032 | NA | NA | NA | NA |
| Qigong vs Stretch | 0 | 0 | 0.64475527 | 0.43838347 | NA | NA | 0.64475527 | 0.43838347 | NA | NA | NA | NA |
| Qigong vs Tai Chi | 0 | 0 | 0.01279613 | 0.46569146 | NA | NA | 0.01279613 | 0.46569146 | NA | NA | NA | NA |
| Qigong vs TT | 0 | 0 | 0.61954585 | 0.4524205 | NA | NA | 0.61954585 | 0.4524205 | NA | NA | NA | NA |
| Qigong vs VR | 0 | 0 | -0.2606774 | 0.41802676 | NA | NA | -0.2606774 | 0.41802676 | NA | NA | NA | NA |
| Qigong vs WBV | 0 | 0 | 1.14287276 | 0.74657864 | NA | NA | 1.14287276 | 0.74657864 | NA | NA | NA | NA |
| Qigong vs Yoga | 0 | 0 | 0.12166469 | 0.52571633 | NA | NA | 0.12166469 | 0.52571633 | NA | NA | NA | NA |
| RA_GT vs RT | 0 | 0 | 0.04795817 | 0.69349024 | NA | NA | 0.04795817 | 0.69349024 | NA | NA | NA | NA |
| RA_GT vs Stretch | 0 | 0 | 0.38034974 | 0.70905247 | NA | NA | 0.38034974 | 0.70905247 | NA | NA | NA | NA |
| RA_GT vs Tai Chi | 0 | 0 | -0.2516094 | 0.72864034 | NA | NA | -0.2516094 | 0.72864034 | NA | NA | NA | NA |
| RA_GT vs TT | 0 | 0 | 0.35514032 | 0.71669408 | NA | NA | 0.35514032 | 0.71669408 | NA | NA | NA | NA |
| RA_GT vs VR | 0 | 0 | -0.5250829 | 0.69570065 | NA | NA | -0.5250829 | 0.69570065 | NA | NA | NA | NA |
| RA_GT vs WBV | 0 | 0 | 0.87846723 | 0.93143628 | NA | NA | 0.87846723 | 0.93143628 | NA | NA | NA | NA |
| RA_GT vs Yoga | 0 | 0 | -0.1427408 | 0.77020518 | NA | NA | -0.1427408 | 0.77020518 | NA | NA | NA | NA |
| RT vs Stretch | 2 | 0.59895017 | 0.33239157 | 0.25144177 | 0.24073838 | 0.32489429 | 0.46927156 | 0.39704365 | -0.2285332 | 0.51303017 | -0.4454576 | 0.6559891 |
| RT vs Tai Chi | 1 | 0.51042035 | -0.2995676 | 0.30751256 | -0.3256699 | 0.43042637 | -0.2723541 | 0.43949221 | -0.0533158 | 0.61515873 | -0.08667 | 0.93093385 |
| RT vs TT | 0 | 0 | 0.30718215 | 0.32658452 | NA | NA | 0.30718215 | 0.32658452 | NA | NA | NA | NA |
| RT vs VR | 1 | 0.26155639 | -0.5730411 | 0.25304561 | -0.3657485 | 0.49478455 | -0.646464 | 0.29446937 | 0.28071554 | 0.57578118 | 0.48753858 | 0.62587671 |
| RT vs WBV | 0 | 0 | 0.83050906 | 0.66889653 | NA | NA | 0.83050906 | 0.66889653 | NA | NA | NA | NA |
| RT vs Yoga | 0 | 0 | -0.190699 | 0.43199787 | NA | NA | -0.190699 | 0.43199787 | NA | NA | NA | NA |
| Stretch vs Tai Chi | 1 | 0.56678674 | -0.6319591 | 0.32493895 | -0.7017132 | 0.43161043 | -0.5406977 | 0.4936861 | -0.1610155 | 0.65575416 | -0.2455424 | 0.80603645 |
| Stretch vs TT | 0 | 0 | -0.0252094 | 0.34817966 | NA | NA | -0.0252094 | 0.34817966 | NA | NA | NA | NA |
| Stretch vs VR | 0 | 0 | -0.9054327 | 0.29496564 | NA | NA | -0.9054327 | 0.29496564 | NA | NA | NA | NA |
| Stretch vs WBV | 0 | 0 | 0.49811749 | 0.6629575 | NA | NA | 0.49811749 | 0.6629575 | NA | NA | NA | NA |
| Stretch vs Yoga | 0 | 0 | -0.5230906 | 0.45921925 | NA | NA | -0.5230906 | 0.45921925 | NA | NA | NA | NA |
| Tai Chi vs TT | 0 | 0 | 0.60674972 | 0.39576606 | NA | NA | 0.60674972 | 0.39576606 | NA | NA | NA | NA |
| Tai Chi vs VR | 0 | 0 | -0.2734735 | 0.34925818 | NA | NA | -0.2734735 | 0.34925818 | NA | NA | NA | NA |
| Tai Chi vs WBV | 0 | 0 | 1.13007663 | 0.70465015 | NA | NA | 1.13007663 | 0.70465015 | NA | NA | NA | NA |
| Tai Chi vs Yoga | 0 | 0 | 0.10886857 | 0.48535635 | NA | NA | 0.10886857 | 0.48535635 | NA | NA | NA | NA |
| TT vs VR | 0 | 0 | -0.8802233 | 0.330282 | NA | NA | -0.8802233 | 0.330282 | NA | NA | NA | NA |
| TT vs WBV | 0 | 0 | 0.52332691 | 0.68907105 | NA | NA | 0.52332691 | 0.68907105 | NA | NA | NA | NA |
| TT vs Yoga | 0 | 0 | -0.4978812 | 0.47263788 | NA | NA | -0.4978812 | 0.47263788 | NA | NA | NA | NA |
| VR vs WBV | 0 | 0 | 1.40355017 | 0.66715219 | NA | NA | 1.40355017 | 0.66715219 | NA | NA | NA | NA |
| VR vs Yoga | 0 | 0 | 0.3823421 | 0.43982795 | NA | NA | 0.3823421 | 0.43982795 | NA | NA | NA | NA |
| WBV vs Yoga | 0 | 0 | -1.0212081 | 0.7590006 | NA | NA | -1.0212081 | 0.7590006 | NA | NA | NA | NA |

*NA* not available, *k* Number of studies providing direct evidence, *prop* Direct evidence proportion, *nma* Estimated treatment effect (SMD) in network meta-analysis, *direct* Estimated treatment effect (SMD) derived from direct evidence, *indir.* Estimated treatment effect (SMD) derived from indirect evidence, *Diff* Difference between direct and indirect treatment estimates, *z* z-value of test for disagreement (direct versus indirect), *p* p-value of test for disagreement (direct versus indirect).

## Table 7.3 Details of SIDE splitting results (dynamic steady-state balance)

| **Comparison** | **k** | **prop** | **NMA** | | **Direct** | | **Indir** | | **Diff** | | **z** | **p** |
| --- | --- | --- | --- | --- | --- | --- | --- | --- | --- | --- | --- | --- |
|  |  |  | **TE** | **seTE** | **TE** | **seTE** | **TE** | **seTE** | **TE** | **seTE** |  |  |
| AE vs AQE | 0 | 0 | -0.059596 | 0.48336114 | NA | NA | -0.059596 | 0.48336114 | NA | NA | NA | NA |
| AE vs BGT | 2 | 0.24982345 | 0.02939448 | 0.18322272 | 0.26041663 | 0.3665749 | -0.0475404 | 0.21154247 | 0.30795705 | 0.42323442 | 0.72762761 | 0.46684158 |
| AE vs BGT_ECA | 1 | 0.14489985 | 0.03235162 | 0.18243644 | 0.33611619 | 0.47926709 | -0.0191224 | 0.1972891 | 0.35523859 | 0.51828557 | 0.68541092 | 0.49308466 |
| AE vs BGT_ICA | 0 | 0 | 0.15711113 | 0.30655503 | NA | NA | 0.15711113 | 0.30655503 | NA | NA | NA | NA |
| AE vs BWS_TT | 0 | 0 | -1.124339 | 0.27918035 | NA | NA | -1.124339 | 0.27918035 | NA | NA | NA | NA |
| AE vs CON | 4 | 0.3961693 | 0.40763687 | 0.16038615 | 0.50393172 | 0.25481587 | 0.34445846 | 0.2063998 | 0.15947327 | 0.32792073 | 0.48631652 | 0.62674277 |
| AE vs CPP | 0 | 0 | 0.02315266 | 0.19414376 | NA | NA | 0.02315266 | 0.19414376 | NA | NA | NA | NA |
| AE vs Dance | 0 | 0 | 0.08624768 | 0.28652745 | NA | NA | 0.08624768 | 0.28652745 | NA | NA | NA | NA |
| AE vs DT_BGT | 0 | 0 | -0.1009863 | 0.18676289 | NA | NA | -0.1009863 | 0.18676289 | NA | NA | NA | NA |
| AE vs Mul_C | 1 | 0.15303585 | 0.0011573 | 0.18602947 | -0.0429593 | 0.47553793 | 0.00912862 | 0.20213859 | -0.0520879 | 0.51671688 | -0.1008055 | 0.91970484 |
| AE vs Mul_D | 0 | 0 | -0.089411 | 0.25056294 | NA | NA | -0.089411 | 0.25056294 | NA | NA | NA | NA |
| AE vs NW | 0 | 0 | 0.00566342 | 0.35536949 | NA | NA | 0.00566342 | 0.35536949 | NA | NA | NA | NA |
| AE vs PT | 0 | 0 | 0.13621665 | 0.35823912 | NA | NA | 0.13621665 | 0.35823912 | NA | NA | NA | NA |
| AE vs Qigong | 0 | 0 | 0.10810507 | 0.27897939 | NA | NA | 0.10810507 | 0.27897939 | NA | NA | NA | NA |
| AE vs RA_GT | 1 | 0.26613742 | 0.10031296 | 0.23091997 | -0.6998407 | 0.44761902 | 0.39049103 | 0.26955918 | -1.0903317 | 0.52251788 | -2.0866879 | 0.03691636 |
| AE vs RT | 2 | 0.20342868 | 0.0560333 | 0.1728895 | 0.10076573 | 0.38332093 | 0.04460952 | 0.1937119 | 0.05615622 | 0.42948717 | 0.13075179 | 0.89597166 |
| AE vs Stretch | 1 | 0.24143525 | 0.78812919 | 0.22147261 | 0.89070636 | 0.45073334 | 0.75548103 | 0.25428673 | 0.13522533 | 0.51751549 | 0.26129716 | 0.79386336 |
| AE vs Tango | 0 | 0 | 0.618186 | 0.28073956 | NA | NA | 0.618186 | 0.28073956 | NA | NA | NA | NA |
| AE vs TC | 0 | 0 | 0.04843406 | 0.23202973 | NA | NA | 0.04843406 | 0.23202973 | NA | NA | NA | NA |
| AE vs TT | 1 | 0.13000144 | -0.1245902 | 0.17540315 | -0.2595796 | 0.48647813 | -0.1044192 | 0.18805207 | -0.1551604 | 0.52155973 | -0.2974931 | 0.76609006 |
| AE vs VR | 1 | 0.20815525 | -0.1965057 | 0.20235588 | -0.3070744 | 0.44352913 | -0.1674401 | 0.2274028 | -0.1396343 | 0.49842765 | -0.2801496 | 0.7793627 |
| AE vs WBV | 0 | 0 | -0.2428888 | 0.56767741 | NA | NA | -0.2428888 | 0.56767741 | NA | NA | NA | NA |
| AE vs Yoga | 0 | 0 | 0.14056511 | 0.32561866 | NA | NA | 0.14056511 | 0.32561866 | NA | NA | NA | NA |
| AQE vs BGT | 0 | 0 | 0.08899047 | 0.47200725 | NA | NA | 0.08899047 | 0.47200725 | NA | NA | NA | NA |
| AQE vs BGT_ECA | 0 | 0 | 0.09194762 | 0.46942983 | NA | NA | 0.09194762 | 0.46942983 | NA | NA | NA | NA |
| AQE vs BGT_ICA | 0 | 0 | 0.21670713 | 0.52972419 | NA | NA | 0.21670713 | 0.52972419 | NA | NA | NA | NA |
| AQE vs BWS_TT | 0 | 0 | -1.064743 | 0.51152475 | NA | NA | -1.064743 | 0.51152475 | NA | NA | NA | NA |
| AQE vs CON | 0 | 0 | 0.46723287 | 0.45926779 | NA | NA | 0.46723287 | 0.45926779 | NA | NA | NA | NA |
| AQE vs CPP | 1 | 1 | 0.08274866 | 0.4426581 | 0.08274866 | 0.4426581 | NA | NA | NA | NA | NA | NA |
| AQE vs Dance | 0 | 0 | 0.14584368 | 0.51838873 | NA | NA | 0.14584368 | 0.51838873 | NA | NA | NA | NA |
| AQE vs DT_BGT | 0 | 0 | -0.0413903 | 0.46867806 | NA | NA | -0.0413903 | 0.46867806 | NA | NA | NA | NA |
| AQE vs Mul_C | 0 | 0 | 0.0607533 | 0.47044579 | NA | NA | 0.0607533 | 0.47044579 | NA | NA | NA | NA |
| AQE vs Mul_D | 0 | 0 | -0.029815 | 0.48303454 | NA | NA | -0.029815 | 0.48303454 | NA | NA | NA | NA |
| AQE vs NW | 0 | 0 | 0.06525942 | 0.55956321 | NA | NA | 0.06525942 | 0.55956321 | NA | NA | NA | NA |
| AQE vs PT | 0 | 0 | 0.19581265 | 0.56009763 | NA | NA | 0.19581265 | 0.56009763 | NA | NA | NA | NA |
| AQE vs Qigong | 0 | 0 | 0.16770107 | 0.50441797 | NA | NA | 0.16770107 | 0.50441797 | NA | NA | NA | NA |
| AQE vs RA_GT | 0 | 0 | 0.15990896 | 0.49736033 | NA | NA | 0.15990896 | 0.49736033 | NA | NA | NA | NA |
| AQE vs RT | 0 | 0 | 0.1156293 | 0.46221212 | NA | NA | 0.1156293 | 0.46221212 | NA | NA | NA | NA |
| AQE vs Stretch | 0 | 0 | 0.84772519 | 0.49026271 | NA | NA | 0.84772519 | 0.49026271 | NA | NA | NA | NA |
| AQE vs Tango | 0 | 0 | 0.67778199 | 0.51586455 | NA | NA | 0.67778199 | 0.51586455 | NA | NA | NA | NA |
| AQE vs TC | 0 | 0 | 0.10803006 | 0.48836425 | NA | NA | 0.10803006 | 0.48836425 | NA | NA | NA | NA |
| AQE vs TT | 0 | 0 | -0.0649942 | 0.46836274 | NA | NA | -0.0649942 | 0.46836274 | NA | NA | NA | NA |
| AQE vs VR | 0 | 0 | -0.1369097 | 0.48085041 | NA | NA | -0.1369097 | 0.48085041 | NA | NA | NA | NA |
| AQE vs WBV | 0 | 0 | -0.1832928 | 0.71206488 | NA | NA | -0.1832928 | 0.71206488 | NA | NA | NA | NA |
| AQE vs Yoga | 0 | 0 | 0.20016111 | 0.54020546 | NA | NA | 0.20016111 | 0.54020546 | NA | NA | NA | NA |
| BGT vs BGT_ECA | 3 | 0.26369416 | 0.00295714 | 0.14681019 | -0.1770664 | 0.2858946 | 0.06742922 | 0.17109097 | -0.2444957 | 0.33317839 | -0.7338281 | 0.46305353 |
| BGT vs BGT_ICA | 1 | 0.27684643 | 0.12771666 | 0.28182279 | 0.0958966 | 0.53561983 | 0.1398984 | 0.33140636 | -0.0440018 | 0.62985616 | -0.0698601 | 0.94430503 |
| BGT vs BWS_TT | 1 | 0.20881383 | -1.1537335 | 0.25125948 | -0.3691416 | 0.54984823 | -1.3608069 | 0.28247702 | 0.99166527 | 0.61816368 | 1.60421147 | 0.10866745 |
| BGT vs CON | 3 | 0.15592643 | 0.37824239 | 0.12505234 | 0.42287489 | 0.31668827 | 0.36999739 | 0.13611365 | 0.05287749 | 0.34470043 | 0.1534013 | 0.87808182 |
| BGT vs CPP | 0 | 0 | -0.0062418 | 0.16384336 | NA | NA | -0.0062418 | 0.16384336 | NA | NA | NA | NA |
| BGT vs Dance | 0 | 0 | 0.0568532 | 0.26883532 | NA | NA | 0.0568532 | 0.26883532 | NA | NA | NA | NA |
| BGT vs DT_BGT | 5 | 0.3963512 | -0.1303807 | 0.13907473 | -0.4887267 | 0.22090632 | 0.1049065 | 0.17900125 | -0.5936332 | 0.28432561 | -2.0878639 | 0.03681011 |
| BGT vs Mul_C | 0 | 0 | -0.0282372 | 0.1633959 | NA | NA | -0.0282372 | 0.1633959 | NA | NA | NA | NA |
| BGT vs Mul_D | 0 | 0 | -0.1188055 | 0.22564193 | NA | NA | -0.1188055 | 0.22564193 | NA | NA | NA | NA |
| BGT vs NW | 0 | 0 | -0.0237311 | 0.34210563 | NA | NA | -0.0237311 | 0.34210563 | NA | NA | NA | NA |
| BGT vs PT | 0 | 0 | 0.10682218 | 0.34213315 | NA | NA | 0.10682218 | 0.34213315 | NA | NA | NA | NA |
| BGT vs Qigong | 0 | 0 | 0.0787106 | 0.25925331 | NA | NA | 0.0787106 | 0.25925331 | NA | NA | NA | NA |
| BGT vs RA_GT | 1 | 0.20872953 | 0.07091848 | 0.21245728 | 1.0034788 | 0.46502861 | -0.1750819 | 0.23884114 | 1.17856075 | 0.52277786 | 2.25441976 | 0.02416977 |
| BGT vs RT | 3 | 0.23231331 | 0.02663883 | 0.1390651 | 0.30942798 | 0.28852345 | -0.0589373 | 0.15871799 | 0.36836532 | 0.32929801 | 1.11863815 | 0.26329454 |
| BGT vs Stretch | 0 | 0 | 0.75873472 | 0.20775482 | NA | NA | 0.75873472 | 0.20775482 | NA | NA | NA | NA |
| BGT vs Tango | 0 | 0 | 0.58879152 | 0.26406224 | NA | NA | 0.58879152 | 0.26406224 | NA | NA | NA | NA |
| BGT vs TC | 1 | 0.13432165 | 0.01903958 | 0.20712428 | -0.2364425 | 0.56514256 | 0.05868106 | 0.22261413 | -0.2951236 | 0.60740692 | -0.4858746 | 0.62705611 |
| BGT vs TT | 1 | 0.0803311 | -0.1539847 | 0.14486457 | -0.5665811 | 0.51111698 | -0.1179453 | 0.15105894 | -0.4486358 | 0.5329722 | -0.8417622 | 0.39992109 |
| BGT vs VR | 3 | 0.42613934 | -0.2259002 | 0.1663315 | -0.2211843 | 0.25479957 | -0.2294021 | 0.21956913 | 0.00821771 | 0.33635312 | 0.02443181 | 0.98050818 |
| BGT vs WBV | 0 | 0 | -0.2722832 | 0.55382222 | NA | NA | -0.2722832 | 0.55382222 | NA | NA | NA | NA |
| BGT vs Yoga | 1 | 0.28863928 | 0.11117064 | 0.30300059 | 0.0147777 | 0.56398266 | 0.1502827 | 0.35925145 | -0.135505 | 0.66868382 | -0.2026444 | 0.83941302 |
| BGT_ECA vs BGT_ICA | 2 | 0.61811569 | 0.12475951 | 0.26952916 | 0.13498948 | 0.34282373 | 0.10820134 | 0.43615399 | 0.02678814 | 0.55475978 | 0.04828782 | 0.96148686 |
| BGT_ECA vs BWS_TT | 2 | 0.52984554 | -1.1566906 | 0.24166485 | -1.6122672 | 0.33200058 | -0.6432738 | 0.3524465 | -0.9689935 | 0.48419307 | -2.0012544 | 0.04536499 |
| BGT_ECA vs CON | 9 | 0.57156148 | 0.37528525 | 0.11230752 | 0.42651646 | 0.14855161 | 0.30693989 | 0.17157917 | 0.11957657 | 0.22695152 | 0.52688155 | 0.59827585 |
| BGT_ECA vs CPP | 0 | 0 | -0.009199 | 0.15626314 | NA | NA | -0.009199 | 0.15626314 | NA | NA | NA | NA |
| BGT_ECA vs Dance | 0 | 0 | 0.05389606 | 0.26332232 | NA | NA | 0.05389606 | 0.26332232 | NA | NA | NA | NA |
| BGT_ECA vs DT_BGT | 0 | 0 | -0.1333379 | 0.14934063 | NA | NA | -0.1333379 | 0.14934063 | NA | NA | NA | NA |
| BGT_ECA vs Mul_C | 1 | 0.09602084 | -0.0311943 | 0.15252811 | 0.40826367 | 0.49222893 | -0.0778736 | 0.16042449 | 0.4861373 | 0.51771163 | 0.93901173 | 0.34772472 |
| BGT_ECA vs Mul_D | 1 | 0.16991022 | -0.1217626 | 0.2173105 | 0.0959913 | 0.52719462 | -0.1663344 | 0.23851636 | 0.26232572 | 0.57863997 | 0.45334876 | 0.65029763 |
| BGT_ECA vs NW | 0 | 0 | -0.0266882 | 0.33739638 | NA | NA | -0.0266882 | 0.33739638 | NA | NA | NA | NA |
| BGT_ECA vs PT | 0 | 0 | 0.10386504 | 0.33963505 | NA | NA | 0.10386504 | 0.33963505 | NA | NA | NA | NA |
| BGT_ECA vs Qigong | 0 | 0 | 0.07575345 | 0.25532574 | NA | NA | 0.07575345 | 0.25532574 | NA | NA | NA | NA |
| BGT_ECA vs RA_GT | 0 | 0 | 0.06796134 | 0.21757906 | NA | NA | 0.06796134 | 0.21757906 | NA | NA | NA | NA |
| BGT_ECA vs RT | 2 | 0.2167851 | 0.02368168 | 0.13371155 | 0.05688039 | 0.28718001 | 0.01449265 | 0.15108747 | 0.04238773 | 0.32449928 | 0.13062505 | 0.89607193 |
| BGT_ECA vs Stretch | 0 | 0 | 0.75577758 | 0.20589179 | NA | NA | 0.75577758 | 0.20589179 | NA | NA | NA | NA |
| BGT_ECA vs Tango | 0 | 0 | 0.58583438 | 0.25978408 | NA | NA | 0.58583438 | 0.25978408 | NA | NA | NA | NA |
| BGT_ECA vs TC | 0 | 0 | 0.01608244 | 0.20553122 | NA | NA | 0.01608244 | 0.20553122 | NA | NA | NA | NA |
| BGT_ECA vs TT | 4 | 0.31420113 | -0.1569419 | 0.13359413 | -0.2707562 | 0.23833262 | -0.1047974 | 0.16132029 | -0.1659588 | 0.28779624 | -0.5766539 | 0.56417325 |
| BGT_ECA vs VR | 0 | 0 | -0.2288573 | 0.17760116 | NA | NA | -0.2288573 | 0.17760116 | NA | NA | NA | NA |
| BGT_ECA vs WBV | 0 | 0 | -0.2752404 | 0.5564889 | NA | NA | -0.2752404 | 0.5564889 | NA | NA | NA | NA |
| BGT_ECA vs Yoga | 0 | 0 | 0.1082135 | 0.30554483 | NA | NA | 0.1082135 | 0.30554483 | NA | NA | NA | NA |
| BGT_ICA vs BWS_TT | 0 | 0 | -1.2814501 | 0.3495544 | NA | NA | -1.2814501 | 0.3495544 | NA | NA | NA | NA |
| BGT_ICA vs CON | 2 | 0.54942674 | 0.25052574 | 0.26760082 | 0.28882438 | 0.36102107 | 0.20382456 | 0.3986619 | 0.08499982 | 0.53783597 | 0.15804042 | 0.87442495 |
| BGT_ICA vs CPP | 0 | 0 | -0.1339585 | 0.29096654 | NA | NA | -0.1339585 | 0.29096654 | NA | NA | NA | NA |
| BGT_ICA vs Dance | 0 | 0 | -0.0708635 | 0.35990117 | NA | NA | -0.0708635 | 0.35990117 | NA | NA | NA | NA |
| BGT_ICA vs DT_BGT | 0 | 0 | -0.2580974 | 0.28583171 | NA | NA | -0.2580974 | 0.28583171 | NA | NA | NA | NA |
| BGT_ICA vs Mul_C | 0 | 0 | -0.1559538 | 0.28888813 | NA | NA | -0.1559538 | 0.28888813 | NA | NA | NA | NA |
| BGT_ICA vs Mul_D | 0 | 0 | -0.2465221 | 0.32964055 | NA | NA | -0.2465221 | 0.32964055 | NA | NA | NA | NA |
| BGT_ICA vs NW | 0 | 0 | -0.1514477 | 0.41738917 | NA | NA | -0.1514477 | 0.41738917 | NA | NA | NA | NA |
| BGT_ICA vs PT | 0 | 0 | -0.0208945 | 0.41752588 | NA | NA | -0.0208945 | 0.41752588 | NA | NA | NA | NA |
| BGT_ICA vs Qigong | 0 | 0 | -0.0490061 | 0.35329637 | NA | NA | -0.0490061 | 0.35329637 | NA | NA | NA | NA |
| BGT_ICA vs RA_GT | 0 | 0 | -0.0567982 | 0.32853184 | NA | NA | -0.0567982 | 0.32853184 | NA | NA | NA | NA |
| BGT_ICA vs RT | 0 | 0 | -0.1010778 | 0.28025501 | NA | NA | -0.1010778 | 0.28025501 | NA | NA | NA | NA |
| BGT_ICA vs Stretch | 0 | 0 | 0.63101806 | 0.32094851 | NA | NA | 0.63101806 | 0.32094851 | NA | NA | NA | NA |
| BGT_ICA vs Tango | 0 | 0 | 0.46107487 | 0.35770953 | NA | NA | 0.46107487 | 0.35770953 | NA | NA | NA | NA |
| BGT_ICA vs TC | 0 | 0 | -0.1086771 | 0.31927836 | NA | NA | -0.1086771 | 0.31927836 | NA | NA | NA | NA |
| BGT_ICA vs TT | 0 | 0 | -0.2817014 | 0.28254149 | NA | NA | -0.2817014 | 0.28254149 | NA | NA | NA | NA |
| BGT_ICA vs VR | 0 | 0 | -0.3536168 | 0.30243774 | NA | NA | -0.3536168 | 0.30243774 | NA | NA | NA | NA |
| BGT_ICA vs WBV | 0 | 0 | -0.3999999 | 0.60751711 | NA | NA | -0.3999999 | 0.60751711 | NA | NA | NA | NA |
| BGT_ICA vs Yoga | 0 | 0 | -0.016546 | 0.39023207 | NA | NA | -0.016546 | 0.39023207 | NA | NA | NA | NA |
| BWS_TT vs CON | 2 | 0.46146031 | 1.53197588 | 0.23762901 | 2.29853589 | 0.34981006 | 0.87513103 | 0.32381026 | 1.42340486 | 0.47667616 | 2.98610458 | 0.00282556 |
| BWS_TT vs CPP | 1 | 0.22142234 | 1.14749167 | 0.2563423 | 0.21130974 | 0.54476546 | 1.41373562 | 0.2905155 | -1.2024259 | 0.61738859 | -1.9475998 | 0.05146287 |
| BWS_TT vs Dance | 0 | 0 | 1.21058668 | 0.33653676 | NA | NA | 1.21058668 | 0.33653676 | NA | NA | NA | NA |
| BWS_TT vs DT_BGT | 0 | 0 | 1.02335276 | 0.25598444 | NA | NA | 1.02335276 | 0.25598444 | NA | NA | NA | NA |
| BWS_TT vs Mul_C | 0 | 0 | 1.12549631 | 0.26028772 | NA | NA | 1.12549631 | 0.26028772 | NA | NA | NA | NA |
| BWS_TT vs Mul_D | 0 | 0 | 1.03492803 | 0.30219141 | NA | NA | 1.03492803 | 0.30219141 | NA | NA | NA | NA |
| BWS_TT vs NW | 0 | 0 | 1.13000243 | 0.39742008 | NA | NA | 1.13000243 | 0.39742008 | NA | NA | NA | NA |
| BWS_TT vs PT | 0 | 0 | 1.26055566 | 0.39885842 | NA | NA | 1.26055566 | 0.39885842 | NA | NA | NA | NA |
| BWS_TT vs Qigong | 0 | 0 | 1.23244408 | 0.32893605 | NA | NA | 1.23244408 | 0.32893605 | NA | NA | NA | NA |
| BWS_TT vs RA_GT | 0 | 0 | 1.22465197 | 0.30159959 | NA | NA | 1.22465197 | 0.30159959 | NA | NA | NA | NA |
| BWS_TT vs RT | 0 | 0 | 1.18037231 | 0.2493546 | NA | NA | 1.18037231 | 0.2493546 | NA | NA | NA | NA |
| BWS_TT vs Stretch | 0 | 0 | 1.9124682 | 0.29401263 | NA | NA | 1.9124682 | 0.29401263 | NA | NA | NA | NA |
| BWS_TT vs Tango | 0 | 0 | 1.742525 | 0.33391548 | NA | NA | 1.742525 | 0.33391548 | NA | NA | NA | NA |
| BWS_TT vs TC | 0 | 0 | 1.17277306 | 0.29318841 | NA | NA | 1.17277306 | 0.29318841 | NA | NA | NA | NA |
| BWS_TT vs TT | 1 | 0.2083151 | 0.99974876 | 0.2486394 | 0.21130974 | 0.54476546 | 1.20720978 | 0.27944334 | -0.9959 | 0.61225648 | -1.626606 | 0.10382081 |
| BWS_TT vs VR | 0 | 0 | 0.92783332 | 0.27376229 | NA | NA | 0.92783332 | 0.27376229 | NA | NA | NA | NA |
| BWS_TT vs WBV | 0 | 0 | 0.88145026 | 0.59405833 | NA | NA | 0.88145026 | 0.59405833 | NA | NA | NA | NA |
| BWS_TT vs Yoga | 0 | 0 | 1.26490412 | 0.3698679 | NA | NA | 1.26490412 | 0.3698679 | NA | NA | NA | NA |
| CON vs CPP | 6 | 0.4526531 | 0.38448421 | 0.12239569 | 0.38524898 | 0.18192123 | 0.38385175 | 0.16543782 | 0.00139723 | 0.24589634 | 0.00568219 | 0.9954663 |
| CON vs Dance | 2 | 0.5483203 | 0.32138919 | 0.24409471 | 0.24120551 | 0.32964098 | 0.41872884 | 0.36319769 | -0.1775233 | 0.49048521 | -0.3619341 | 0.71740126 |
| CON vs DT_BGT | 7 | 0.55511671 | 0.50862312 | 0.11364711 | 0.38479127 | 0.15253386 | 0.66313809 | 0.17038657 | -0.2783468 | 0.22868791 | -1.2171471 | 0.22354826 |
| CON vs Mul_C | 9 | 0.7110621 | 0.40647957 | 0.11541758 | 0.33598968 | 0.13687313 | 0.57995174 | 0.21471861 | -0.2439621 | 0.25463373 | -0.9580901 | 0.33801732 |
| CON vs Mul_D | 1 | 0.13565391 | 0.49704785 | 0.20033244 | 0 | 0.54392011 | 0.57505651 | 0.21548023 | -0.5750565 | 0.58504771 | -0.9829224 | 0.32564562 |
| CON vs NW | 1 | 0.51883825 | 0.40197345 | 0.32314282 | 0.12536566 | 0.44861989 | 0.70024051 | 0.46585308 | -0.5748748 | 0.64674485 | -0.8888743 | 0.37407066 |
| CON vs PT | 2 | 0.88871827 | 0.27142021 | 0.32123525 | 0.43778376 | 0.34075414 | -1.0571925 | 0.96296669 | 1.49497627 | 1.02147845 | 1.46354167 | 0.14331922 |
| CON vs Qigong | 1 | 0.38359744 | 0.2995318 | 0.23334641 | 0.44252655 | 0.37675869 | 0.21054381 | 0.29721376 | 0.23198274 | 0.47987825 | 0.48341999 | 0.62879756 |
| CON vs RA_GT | 0 | 0 | 0.30732391 | 0.20122695 | NA | NA | 0.30732391 | 0.20122695 | NA | NA | NA | NA |
| CON vs RT | 11 | 0.52989525 | 0.35160357 | 0.09901761 | 0.50964206 | 0.13602459 | 0.17346488 | 0.14441593 | 0.33617718 | 0.19839015 | 1.69452559 | 0.09016545 |
| CON vs Stretch | 0 | 0 | -0.3804923 | 0.18565375 | NA | NA | -0.3804923 | 0.18565375 | NA | NA | NA | NA |
| CON vs Tango | 1 | 0.25866315 | -0.2105491 | 0.24218395 | 0.18873589 | 0.47618759 | -0.3498654 | 0.28127916 | 0.53860133 | 0.55305749 | 0.97386138 | 0.33012537 |
| CON vs TC | 3 | 0.30191275 | 0.35920281 | 0.17939854 | 0.38261199 | 0.32649624 | 0.34907868 | 0.21471583 | 0.03353331 | 0.39077191 | 0.08581301 | 0.93161507 |
| CON vs TT | 5 | 0.21547931 | 0.53222711 | 0.11068828 | 0.43235846 | 0.23845084 | 0.5596574 | 0.12496818 | -0.1272989 | 0.26921339 | -0.4728552 | 0.63631651 |
| CON vs VR | 2 | 0.22570186 | 0.60414256 | 0.15412884 | 0.24585426 | 0.32442651 | 0.7085808 | 0.17515793 | -0.4627265 | 0.36869074 | -1.2550533 | 0.2094594 |
| CON vs WBV | 0 | 0 | 0.65052562 | 0.5479899 | NA | NA | 0.65052562 | 0.5479899 | NA | NA | NA | NA |
| CON vs Yoga | 2 | 0.60505864 | 0.26707176 | 0.28690094 | 0.39181212 | 0.36883595 | 0.07596684 | 0.45652616 | 0.31584529 | 0.58690381 | 0.53815511 | 0.59046997 |
| CPP vs Dance | 0 | 0 | 0.06309502 | 0.26977894 | NA | NA | 0.06309502 | 0.26977894 | NA | NA | NA | NA |
| CPP vs DT_BGT | 1 | 0.10181059 | -0.1241389 | 0.15399002 | -0.4340708 | 0.48260978 | -0.0890079 | 0.16248326 | -0.3450629 | 0.50922786 | -0.6776199 | 0.49801273 |
| CPP vs Mul_C | 1 | 0.12607353 | -0.0219954 | 0.15928921 | -0.6029776 | 0.44861562 | 0.06181774 | 0.17039188 | -0.6647954 | 0.47988474 | -1.385323 | 0.16595373 |
| CPP vs Mul_D | 4 | 0.60293227 | -0.1125636 | 0.19332918 | -0.2987246 | 0.24897924 | 0.17011477 | 0.30680689 | -0.4688394 | 0.39512167 | -1.1865697 | 0.23539739 |
| CPP vs NW | 0 | 0 | -0.0174892 | 0.34229343 | NA | NA | -0.0174892 | 0.34229343 | NA | NA | NA | NA |
| CPP vs PT | 0 | 0 | 0.11306399 | 0.34316638 | NA | NA | 0.11306399 | 0.34316638 | NA | NA | NA | NA |
| CPP vs Qigong | 1 | 0.37504381 | 0.08495241 | 0.24184973 | 0.3952709 | 0.39491589 | -0.1012735 | 0.30592912 | 0.49654439 | 0.49955098 | 0.99398141 | 0.32023192 |
| CPP vs RA_GT | 0 | 0 | 0.0771603 | 0.22676222 | NA | NA | 0.0771603 | 0.22676222 | NA | NA | NA | NA |
| CPP vs RT | 5 | 0.47287226 | 0.03288064 | 0.13301823 | 0.15272532 | 0.19343687 | -0.0746288 | 0.18321171 | 0.22735415 | 0.26642889 | 0.85333895 | 0.39347136 |
| CPP vs Stretch | 0 | 0 | 0.76497653 | 0.21073995 | NA | NA | 0.76497653 | 0.21073995 | NA | NA | NA | NA |
| CPP vs Tango | 0 | 0 | 0.59503333 | 0.26489628 | NA | NA | 0.59503333 | 0.26489628 | NA | NA | NA | NA |
| CPP vs TC | 0 | 0 | 0.0252814 | 0.20628486 | NA | NA | 0.0252814 | 0.20628486 | NA | NA | NA | NA |
| CPP vs TT | 1 | 0.07915341 | -0.1477429 | 0.15302765 | 0 | 0.54392011 | -0.1604425 | 0.159469 | 0.16044247 | 0.56681518 | 0.28305959 | 0.77713116 |
| CPP vs VR | 0 | 0 | -0.2196584 | 0.18780555 | NA | NA | -0.2196584 | 0.18780555 | NA | NA | NA | NA |
| CPP vs WBV | 0 | 0 | -0.2660414 | 0.5577546 | NA | NA | -0.2660414 | 0.5577546 | NA | NA | NA | NA |
| CPP vs Yoga | 0 | 0 | 0.11741245 | 0.30963809 | NA | NA | 0.11741245 | 0.30963809 | NA | NA | NA | NA |
| Dance vs DT_BGT | 0 | 0 | -0.1872339 | 0.26492381 | NA | NA | -0.1872339 | 0.26492381 | NA | NA | NA | NA |
| Dance vs Mul_C | 0 | 0 | -0.0850904 | 0.26733025 | NA | NA | -0.0850904 | 0.26733025 | NA | NA | NA | NA |
| Dance vs Mul_D | 0 | 0 | -0.1756587 | 0.3128792 | NA | NA | -0.1756587 | 0.3128792 | NA | NA | NA | NA |
| Dance vs NW | 0 | 0 | -0.0805843 | 0.40054439 | NA | NA | -0.0805843 | 0.40054439 | NA | NA | NA | NA |
| Dance vs PT | 0 | 0 | 0.04996898 | 0.40315057 | NA | NA | 0.04996898 | 0.40315057 | NA | NA | NA | NA |
| Dance vs Qigong | 0 | 0 | 0.02185739 | 0.33581676 | NA | NA | 0.02185739 | 0.33581676 | NA | NA | NA | NA |
| Dance vs RA_GT | 0 | 0 | 0.01406528 | 0.30636473 | NA | NA | 0.01406528 | 0.30636473 | NA | NA | NA | NA |
| Dance vs RT | 0 | 0 | -0.0302144 | 0.25750757 | NA | NA | -0.0302144 | 0.25750757 | NA | NA | NA | NA |
| Dance vs Stretch | 0 | 0 | 0.70188152 | 0.29600967 | NA | NA | 0.70188152 | 0.29600967 | NA | NA | NA | NA |
| Dance vs Tango | 1 | 0.27755975 | 0.53193832 | 0.30990175 | 0.0606513 | 0.58822812 | 0.71300562 | 0.36460537 | -0.6523543 | 0.6920617 | -0.9426245 | 0.345873 |
| Dance vs TC | 0 | 0 | -0.0378136 | 0.29954489 | NA | NA | -0.0378136 | 0.29954489 | NA | NA | NA | NA |
| Dance vs TT | 1 | 0.35438816 | -0.2108379 | 0.24983565 | 0.15902541 | 0.41967665 | -0.4138627 | 0.31093433 | 0.57288809 | 0.52231087 | 1.09683354 | 0.27271416 |
| Dance vs VR | 0 | 0 | -0.2827534 | 0.28143777 | NA | NA | -0.2827534 | 0.28143777 | NA | NA | NA | NA |
| Dance vs WBV | 0 | 0 | -0.3291364 | 0.59796479 | NA | NA | -0.3291364 | 0.59796479 | NA | NA | NA | NA |
| Dance vs Yoga | 0 | 0 | 0.05431743 | 0.37556691 | NA | NA | 0.05431743 | 0.37556691 | NA | NA | NA | NA |
| DT_BGT vs Mul_C | 0 | 0 | 0.10214355 | 0.15741927 | NA | NA | 0.10214355 | 0.15741927 | NA | NA | NA | NA |
| DT_BGT vs Mul_D | 2 | 0.28072851 | 0.01157527 | 0.21177906 | 0.09776866 | 0.39970516 | -0.0220656 | 0.24971038 | 0.11983429 | 0.47129554 | 0.25426571 | 0.79929029 |
| DT_BGT vs NW | 0 | 0 | 0.10664967 | 0.33910147 | NA | NA | 0.10664967 | 0.33910147 | NA | NA | NA | NA |
| DT_BGT vs PT | 0 | 0 | 0.2372029 | 0.33991392 | NA | NA | 0.2372029 | 0.33991392 | NA | NA | NA | NA |
| DT_BGT vs Qigong | 0 | 0 | 0.20909132 | 0.25544525 | NA | NA | 0.20909132 | 0.25544525 | NA | NA | NA | NA |
| DT_BGT vs RA_GT | 1 | 0.15318498 | 0.20129921 | 0.21332977 | -0.194562 | 0.54505882 | 0.27290869 | 0.23182336 | -0.4674707 | 0.59231004 | -0.789233 | 0.42997582 |
| DT_BGT vs RT | 0 | 0 | 0.15701955 | 0.13969671 | NA | NA | 0.15701955 | 0.13969671 | NA | NA | NA | NA |
| DT_BGT vs Stretch | 0 | 0 | 0.88911544 | 0.20762869 | NA | NA | 0.88911544 | 0.20762869 | NA | NA | NA | NA |
| DT_BGT vs Tango | 0 | 0 | 0.71917225 | 0.26169092 | NA | NA | 0.71917225 | 0.26169092 | NA | NA | NA | NA |
| DT_BGT vs TC | 0 | 0 | 0.14942031 | 0.20677713 | NA | NA | 0.14942031 | 0.20677713 | NA | NA | NA | NA |
| DT_BGT vs TT | 2 | 0.19346157 | -0.023604 | 0.13963679 | -0.0750652 | 0.31746985 | -0.0112602 | 0.15548457 | -0.063805 | 0.35350044 | -0.1804949 | 0.85676408 |
| DT_BGT vs VR | 1 | 0.13418863 | -0.0955194 | 0.17281138 | -0.5513108 | 0.47175278 | -0.0248782 | 0.18572086 | -0.5264326 | 0.50699401 | -1.0383409 | 0.29911136 |
| DT_BGT vs WBV | 1 | 1 | -0.1419025 | 0.53607581 | -0.1419025 | 0.53607581 | NA | NA | NA | NA | NA | NA |
| DT_BGT vs Yoga | 0 | 0 | 0.24155136 | 0.30539939 | NA | NA | 0.24155136 | 0.30539939 | NA | NA | NA | NA |
| Mul_C vs Mul_D | 0 | 0 | -0.0905683 | 0.2262923 | NA | NA | -0.0905683 | 0.2262923 | NA | NA | NA | NA |
| Mul_C vs NW | 1 | 0.53646891 | 0.00450612 | 0.32804458 | 0.4745986 | 0.44787888 | -0.5395566 | 0.48182955 | 1.01415523 | 0.65784132 | 1.54164113 | 0.12316083 |
| Mul_C vs PT | 0 | 0 | 0.13505935 | 0.34080545 | NA | NA | 0.13505935 | 0.34080545 | NA | NA | NA | NA |
| Mul_C vs Qigong | 0 | 0 | 0.10694777 | 0.25506116 | NA | NA | 0.10694777 | 0.25506116 | NA | NA | NA | NA |
| Mul_C vs RA_GT | 0 | 0 | 0.09915566 | 0.22526051 | NA | NA | 0.09915566 | 0.22526051 | NA | NA | NA | NA |
| Mul_C vs RT | 0 | 0 | 0.054876 | 0.14523811 | NA | NA | 0.054876 | 0.14523811 | NA | NA | NA | NA |
| Mul_C vs Stretch | 0 | 0 | 0.78697189 | 0.21155062 | NA | NA | 0.78697189 | 0.21155062 | NA | NA | NA | NA |
| Mul_C vs Tango | 0 | 0 | 0.6170287 | 0.26451827 | NA | NA | 0.6170287 | 0.26451827 | NA | NA | NA | NA |
| Mul_C vs TC | 1 | 0.20569328 | 0.04727676 | 0.20071211 | 0.0132279 | 0.4425512 | 0.05609403 | 0.22520574 | -0.0428661 | 0.49655734 | -0.0863267 | 0.93120675 |
| Mul_C vs TT | 1 | 0.11098309 | -0.1257475 | 0.148636 | 0.1997953 | 0.4461651 | -0.1663877 | 0.15764093 | 0.36618296 | 0.47319548 | 0.77385135 | 0.4390187 |
| Mul_C vs VR | 1 | 0.12750696 | -0.197663 | 0.18317342 | -0.1979422 | 0.5129742 | -0.1976222 | 0.19610175 | -0.00032 | 0.54917978 | -0.0005827 | 0.99953511 |
| Mul_C vs WBV | 0 | 0 | -0.244046 | 0.5587111 | NA | NA | -0.244046 | 0.5587111 | NA | NA | NA | NA |
| Mul_C vs Yoga | 0 | 0 | 0.13940781 | 0.30719838 | NA | NA | 0.13940781 | 0.30719838 | NA | NA | NA | NA |
| Mul_D vs NW | 0 | 0 | 0.0950744 | 0.37740366 | NA | NA | 0.0950744 | 0.37740366 | NA | NA | NA | NA |
| Mul_D vs PT | 0 | 0 | 0.22562763 | 0.37805287 | NA | NA | 0.22562763 | 0.37805287 | NA | NA | NA | NA |
| Mul_D vs Qigong | 0 | 0 | 0.19751605 | 0.2964704 | NA | NA | 0.19751605 | 0.2964704 | NA | NA | NA | NA |
| Mul_D vs RA_GT | 0 | 0 | 0.18972394 | 0.2756995 | NA | NA | 0.18972394 | 0.2756995 | NA | NA | NA | NA |
| Mul_D vs RT | 0 | 0 | 0.14544428 | 0.21166812 | NA | NA | 0.14544428 | 0.21166812 | NA | NA | NA | NA |
| Mul_D vs Stretch | 0 | 0 | 0.87754017 | 0.26523329 | NA | NA | 0.87754017 | 0.26523329 | NA | NA | NA | NA |
| Mul_D vs Tango | 0 | 0 | 0.70759697 | 0.30945582 | NA | NA | 0.70759697 | 0.30945582 | NA | NA | NA | NA |
| Mul_D vs TC | 0 | 0 | 0.13784504 | 0.26262525 | NA | NA | 0.13784504 | 0.26262525 | NA | NA | NA | NA |
| Mul_D vs TT | 0 | 0 | -0.0351793 | 0.2195939 | NA | NA | -0.0351793 | 0.2195939 | NA | NA | NA | NA |
| Mul_D vs VR | 0 | 0 | -0.1070947 | 0.24483285 | NA | NA | -0.1070947 | 0.24483285 | NA | NA | NA | NA |
| Mul_D vs WBV | 0 | 0 | -0.1534778 | 0.57639191 | NA | NA | -0.1534778 | 0.57639191 | NA | NA | NA | NA |
| Mul_D vs Yoga | 0 | 0 | 0.22997609 | 0.3479329 | NA | NA | 0.22997609 | 0.3479329 | NA | NA | NA | NA |
| NW vs PT | 0 | 0 | 0.13055323 | 0.455325 | NA | NA | 0.13055323 | 0.455325 | NA | NA | NA | NA |
| NW vs Qigong | 0 | 0 | 0.10244165 | 0.39642339 | NA | NA | 0.10244165 | 0.39642339 | NA | NA | NA | NA |
| NW vs RA_GT | 0 | 0 | 0.09464954 | 0.37315826 | NA | NA | 0.09464954 | 0.37315826 | NA | NA | NA | NA |
| NW vs RT | 0 | 0 | 0.05036988 | 0.33416033 | NA | NA | 0.05036988 | 0.33416033 | NA | NA | NA | NA |
| NW vs Stretch | 0 | 0 | 0.78246577 | 0.3671047 | NA | NA | 0.78246577 | 0.3671047 | NA | NA | NA | NA |
| NW vs Tango | 0 | 0 | 0.61252258 | 0.3990025 | NA | NA | 0.61252258 | 0.3990025 | NA | NA | NA | NA |
| NW vs TC | 0 | 0 | 0.04277064 | 0.36557279 | NA | NA | 0.04277064 | 0.36557279 | NA | NA | NA | NA |
| NW vs TT | 1 | 0.34763828 | -0.1302537 | 0.32783728 | 0.63754736 | 0.55602528 | -0.5394086 | 0.40589537 | 1.17695597 | 0.68841497 | 1.70966064 | 0.08732865 |
| NW vs VR | 0 | 0 | -0.2021691 | 0.35163998 | NA | NA | -0.2021691 | 0.35163998 | NA | NA | NA | NA |
| NW vs WBV | 0 | 0 | -0.2485522 | 0.63432411 | NA | NA | -0.2485522 | 0.63432411 | NA | NA | NA | NA |
| NW vs Yoga | 0 | 0 | 0.13490169 | 0.43095416 | NA | NA | 0.13490169 | 0.43095416 | NA | NA | NA | NA |
| PT vs Qigong | 0 | 0 | -0.0281116 | 0.39541187 | NA | NA | -0.0281116 | 0.39541187 | NA | NA | NA | NA |
| PT vs RA_GT | 0 | 0 | -0.0359037 | 0.3779465 | NA | NA | -0.0359037 | 0.3779465 | NA | NA | NA | NA |
| PT vs RT | 0 | 0 | -0.0801834 | 0.33526341 | NA | NA | -0.0801834 | 0.33526341 | NA | NA | NA | NA |
| PT vs Stretch | 0 | 0 | 0.65191254 | 0.36965857 | NA | NA | 0.65191254 | 0.36965857 | NA | NA | NA | NA |
| PT vs Tango | 0 | 0 | 0.48196934 | 0.40169851 | NA | NA | 0.48196934 | 0.40169851 | NA | NA | NA | NA |
| PT vs TC | 0 | 0 | -0.0877826 | 0.36332211 | NA | NA | -0.0877826 | 0.36332211 | NA | NA | NA | NA |
| PT vs TT | 0 | 0 | -0.2608069 | 0.33900825 | NA | NA | -0.2608069 | 0.33900825 | NA | NA | NA | NA |
| PT vs VR | 0 | 0 | -0.3327223 | 0.3552404 | NA | NA | -0.3327223 | 0.3552404 | NA | NA | NA | NA |
| PT vs WBV | 0 | 0 | -0.3791054 | 0.63475881 | NA | NA | -0.3791054 | 0.63475881 | NA | NA | NA | NA |
| PT vs Yoga | 1 | 0.56403735 | 0.00434846 | 0.37137321 | -0.1433729 | 0.494489 | 0.19546659 | 0.5624526 | -0.3388395 | 0.74891408 | -0.4524411 | 0.65095127 |
| Qigong vs RA_GT | 0 | 0 | -0.0077921 | 0.30255945 | NA | NA | -0.0077921 | 0.30255945 | NA | NA | NA | NA |
| Qigong vs RT | 0 | 0 | -0.0520718 | 0.24484052 | NA | NA | -0.0520718 | 0.24484052 | NA | NA | NA | NA |
| Qigong vs Stretch | 0 | 0 | 0.68002412 | 0.28737622 | NA | NA | 0.68002412 | 0.28737622 | NA | NA | NA | NA |
| Qigong vs Tango | 0 | 0 | 0.51008092 | 0.33193674 | NA | NA | 0.51008092 | 0.33193674 | NA | NA | NA | NA |
| Qigong vs TC | 2 | 0.44673245 | -0.059671 | 0.2509529 | 0.07881683 | 0.37546414 | -0.1714922 | 0.3373841 | 0.25030899 | 0.50477851 | 0.49587886 | 0.61997988 |
| Qigong vs TT | 0 | 0 | -0.2326953 | 0.25346862 | NA | NA | -0.2326953 | 0.25346862 | NA | NA | NA | NA |
| Qigong vs VR | 0 | 0 | -0.3046108 | 0.27545354 | NA | NA | -0.3046108 | 0.27545354 | NA | NA | NA | NA |
| Qigong vs WBV | 0 | 0 | -0.3509938 | 0.59382619 | NA | NA | -0.3509938 | 0.59382619 | NA | NA | NA | NA |
| Qigong vs Yoga | 0 | 0 | 0.03246004 | 0.36372069 | NA | NA | 0.03246004 | 0.36372069 | NA | NA | NA | NA |
| RA_GT vs RT | 0 | 0 | -0.0442797 | 0.20853158 | NA | NA | -0.0442797 | 0.20853158 | NA | NA | NA | NA |
| RA_GT vs Stretch | 2 | 0.46652107 | 0.68781623 | 0.22799821 | 0.95364537 | 0.33380728 | 0.45535177 | 0.3121568 | 0.4982936 | 0.45702207 | 1.09030533 | 0.27557867 |
| RA_GT vs Tango | 0 | 0 | 0.51787304 | 0.29785264 | NA | NA | 0.51787304 | 0.29785264 | NA | NA | NA | NA |
| RA_GT vs TC | 0 | 0 | -0.0518789 | 0.25785058 | NA | NA | -0.0518789 | 0.25785058 | NA | NA | NA | NA |
| RA_GT vs TT | 2 | 0.44685281 | -0.2249032 | 0.19646905 | -0.279101 | 0.29390834 | -0.1811203 | 0.2641641 | -0.0979807 | 0.3951769 | -0.2479414 | 0.80417977 |
| RA_GT vs VR | 0 | 0 | -0.2968186 | 0.23494964 | NA | NA | -0.2968186 | 0.23494964 | NA | NA | NA | NA |
| RA_GT vs WBV | 0 | 0 | -0.3432017 | 0.57696348 | NA | NA | -0.3432017 | 0.57696348 | NA | NA | NA | NA |
| RA_GT vs Yoga | 0 | 0 | 0.04025215 | 0.34625952 | NA | NA | 0.04025215 | 0.34625952 | NA | NA | NA | NA |
| RT vs Stretch | 3 | 0.52658202 | 0.73209589 | 0.18185433 | 0.59857854 | 0.25060552 | 0.88060702 | 0.2643025 | -0.2820285 | 0.36422375 | -0.7743275 | 0.43873712 |
| RT vs Tango | 1 | 0.19808892 | 0.56215269 | 0.24658754 | 0 | 0.55403989 | 0.70101625 | 0.27536454 | -0.7010162 | 0.61869688 | -1.1330528 | 0.25719207 |
| RT vs TC | 1 | 0.28366199 | -0.0075992 | 0.1895187 | -0.0266939 | 0.35583732 | -3.80E-05 | 0.2239201 | -0.0266559 | 0.42042884 | -0.0634016 | 0.94944672 |
| RT vs TT | 2 | 0.17525029 | -0.1806235 | 0.12775834 | -0.1979624 | 0.30518269 | -0.1769392 | 0.14067865 | -0.0210232 | 0.33604606 | -0.0625605 | 0.95011651 |
| RT vs VR | 1 | 0.14921804 | -0.252539 | 0.16614594 | 0.1307446 | 0.43010953 | -0.3197628 | 0.18012774 | 0.45050743 | 0.46630485 | 0.96612211 | 0.33398308 |
| RT vs WBV | 0 | 0 | -0.2989221 | 0.55397873 | NA | NA | -0.2989221 | 0.55397873 | NA | NA | NA | NA |
| RT vs Yoga | 0 | 0 | 0.08453181 | 0.30009694 | NA | NA | 0.08453181 | 0.30009694 | NA | NA | NA | NA |
| Stretch vs Tango | 1 | 0.45115839 | -0.1699432 | 0.26868729 | 0.108325 | 0.40002082 | -0.398685 | 0.36268 | 0.50701003 | 0.53995689 | 0.93898243 | 0.34773977 |
| Stretch vs TC | 1 | 0.41361202 | -0.7396951 | 0.22990763 | -0.6673461 | 0.35748416 | -0.7907269 | 0.30023468 | 0.12338078 | 0.46683593 | 0.26429153 | 0.79155529 |
| Stretch vs TT | 1 | 0.2066018 | -0.9127194 | 0.19301153 | -1.575226 | 0.42463542 | -0.740202 | 0.21668939 | -0.835024 | 0.47672794 | -1.7515735 | 0.07984717 |
| Stretch vs VR | 0 | 0 | -0.9846349 | 0.22677444 | NA | NA | -0.9846349 | 0.22677444 | NA | NA | NA | NA |
| Stretch vs WBV | 0 | 0 | -1.0310179 | 0.57487994 | NA | NA | -1.0310179 | 0.57487994 | NA | NA | NA | NA |
| Stretch vs Yoga | 0 | 0 | -0.6475641 | 0.33656894 | NA | NA | -0.6475641 | 0.33656894 | NA | NA | NA | NA |
| Tango vs TC | 0 | 0 | -0.5697519 | 0.29241023 | NA | NA | -0.5697519 | 0.29241023 | NA | NA | NA | NA |
| Tango vs TT | 1 | 0.36084259 | -0.7427762 | 0.24727497 | -1.683551 | 0.41164351 | -0.2116526 | 0.30929739 | -1.4718984 | 0.51489344 | -2.8586466 | 0.00425452 |
| Tango vs VR | 0 | 0 | -0.8146917 | 0.27755419 | NA | NA | -0.8146917 | 0.27755419 | NA | NA | NA | NA |
| Tango vs WBV | 0 | 0 | -0.8610747 | 0.59653953 | NA | NA | -0.8610747 | 0.59653953 | NA | NA | NA | NA |
| Tango vs Yoga | 0 | 0 | -0.4776209 | 0.37321939 | NA | NA | -0.4776209 | 0.37321939 | NA | NA | NA | NA |
| TC vs TT | 0 | 0 | -0.1730243 | 0.20191293 | NA | NA | -0.1730243 | 0.20191293 | NA | NA | NA | NA |
| TC vs VR | 0 | 0 | -0.2449397 | 0.22872972 | NA | NA | -0.2449397 | 0.22872972 | NA | NA | NA | NA |
| TC vs WBV | 0 | 0 | -0.2913228 | 0.57457293 | NA | NA | -0.2913228 | 0.57457293 | NA | NA | NA | NA |
| TC vs Yoga | 1 | 0.32178196 | 0.09213106 | 0.32066684 | 0.2512202 | 0.56529248 | 0.01665088 | 0.38937618 | 0.23456932 | 0.68641781 | 0.34172965 | 0.73255435 |
| TT vs VR | 3 | 0.38599576 | -0.0719154 | 0.1606662 | -0.3965385 | 0.25860282 | 0.13215992 | 0.20504012 | -0.5286985 | 0.33002556 | -1.6019925 | 0.10915727 |
| TT vs WBV | 0 | 0 | -0.1182985 | 0.55396363 | NA | NA | -0.1182985 | 0.55396363 | NA | NA | NA | NA |
| TT vs Yoga | 0 | 0 | 0.26515536 | 0.3045885 | NA | NA | 0.26515536 | 0.3045885 | NA | NA | NA | NA |
| VR vs WBV | 0 | 0 | -0.0463831 | 0.56324155 | NA | NA | -0.0463831 | 0.56324155 | NA | NA | NA | NA |
| VR vs Yoga | 0 | 0 | 0.3370708 | 0.32166157 | NA | NA | 0.3370708 | 0.32166157 | NA | NA | NA | NA |
| WBV vs Yoga | 0 | 0 | 0.38345386 | 0.6169652 | NA | NA | 0.38345386 | 0.6169652 | NA | NA | NA | NA |

*NA* not available, *k* Number of studies providing direct evidence, *prop* Direct evidence proportion, *nma* Estimated treatment effect (SMD) in network meta-analysis, *direct* Estimated treatment effect (SMD) derived from direct evidence, *indir.* Estimated treatment effect (SMD) derived from indirect evidence, *Diff* Difference between direct and indirect treatment estimates, *z* z-value of test for disagreement (direct versus indirect), *p* p-value of test for disagreement (direct versus indirect).

## Table 7.4 Details of SIDE splitting results (Proactive balance)

| **Comparison** | **k** | **prop** | **NMA** | | **Direc** | | **Indir** | | **Diff** | | **z** | **p** |
| --- | --- | --- | --- | --- | --- | --- | --- | --- | --- | --- | --- | --- |
|  |  |  | **TE** | **seTE** | **TE** | **seTE** | **TE** | **seTE** | **TE** | **seTE** |  |  |
| AE vs AQE | 1 | 0.14431043 | -0.518896851 | 0.223434433 | 0.0420557 | 0.588167796 | -0.613500439 | 0.241541624 | 0.655556139 | 0.635833086 | 1.031019232 | 0.302531801 |
| AE vs BGT | 4 | 0.439756889 | -0.15659262 | 0.171227325 | -0.066322967 | 0.258206248 | -0.227448827 | 0.228762488 | 0.16112586 | 0.344967741 | 0.467075151 | 0.640446112 |
| AE vs BGT_ECA | 2 | 0.256715766 | -0.230442815 | 0.185223922 | 0.007841396 | 0.365570216 | -0.312741498 | 0.214842212 | 0.320582895 | 0.424026837 | 0.756043878 | 0.449622889 |
| AE vs CON | 3 | 0.235999239 | 0.367263516 | 0.154751817 | -0.086223127 | 0.318552087 | 0.507345186 | 0.177047117 | -0.593568313 | 0.364446311 | -1.6286852 | 0.103379677 |
| AE vs CPP | 0 | 0 | 0.044627369 | 0.213028742 | NA | NA | 0.044627369 | 0.213028742 | NA | NA | NA | NA |
| AE vs Dance | 0 | 0 | 0.041462028 | 0.269756807 | NA | NA | 0.041462028 | 0.269756807 | NA | NA | NA | NA |
| AE vs DT_BGT | 0 | 0 | -0.286873373 | 0.250943388 | NA | NA | -0.286873373 | 0.250943388 | NA | NA | NA | NA |
| AE vs Mul_C | 4 | 0.428493989 | -0.395897861 | 0.162844487 | -0.238058974 | 0.24877154 | -0.514239599 | 0.215408412 | 0.276180626 | 0.329071517 | 0.839272352 | 0.401316495 |
| AE vs Mul_D | 0 | 0 | -0.289329932 | 0.425216209 | NA | NA | -0.289329932 | 0.425216209 | NA | NA | NA | NA |
| AE vs NW | 0 | 0 | 0.43120792 | 0.316930756 | NA | NA | 0.43120792 | 0.316930756 | NA | NA | NA | NA |
| AE vs Pilates | 0 | 0 | -1.057432224 | 0.447346569 | NA | NA | -1.057432224 | 0.447346569 | NA | NA | NA | NA |
| AE vs PT | 0 | 0 | -0.285811903 | 0.351685002 | NA | NA | -0.285811903 | 0.351685002 | NA | NA | NA | NA |
| AE vs Qigong | 0 | 0 | -0.461679135 | 0.298111354 | NA | NA | -0.461679135 | 0.298111354 | NA | NA | NA | NA |
| AE vs RA_GT | 0 | 0 | -0.005543409 | 0.308771225 | NA | NA | -0.005543409 | 0.308771225 | NA | NA | NA | NA |
| AE vs RT | 2 | 0.153459363 | -0.252388005 | 0.169674305 | 0.091553333 | 0.433131116 | -0.314737072 | 0.184413273 | 0.406290405 | 0.470755583 | 0.863060194 | 0.388104372 |
| AE vs Stretch | 2 | 0.385082313 | 0.292441985 | 0.214246016 | 0.128418009 | 0.345251814 | 0.395159363 | 0.273214826 | -0.266741354 | 0.440278499 | -0.605846878 | 0.544616438 |
| AE vs Tai Chi | 0 | 0 | -0.370297186 | 0.232886596 | NA | NA | -0.370297186 | 0.232886596 | NA | NA | NA | NA |
| AE vs Tango | 0 | 0 | -0.402106652 | 0.302442122 | NA | NA | -0.402106652 | 0.302442122 | NA | NA | NA | NA |
| AE vs TT | 1 | 0.132852236 | -0.028978319 | 0.205093952 | -0.5186961 | 0.562688993 | 0.04604938 | 0.220245122 | -0.56474548 | 0.604257244 | -0.934611021 | 0.349988797 |
| AE vs VR | 0 | 0 | -0.138959753 | 0.223311408 | NA | NA | -0.138959753 | 0.223311408 | NA | NA | NA | NA |
| AE vs WBV | 0 | 0 | -0.209346063 | 0.652469904 | NA | NA | -0.209346063 | 0.652469904 | NA | NA | NA | NA |
| AE vs Yoga | 0 | 0 | -0.161180921 | 0.275147031 | NA | NA | -0.161180921 | 0.275147031 | NA | NA | NA | NA |
| AQE vs BGT | 1 | 0.158724116 | 0.362304231 | 0.214785158 | 0.28098631 | 0.539116731 | 0.377646541 | 0.234172045 | -0.096660231 | 0.587778356 | -0.164450136 | 0.869376798 |
| AQE vs BGT_ECA | 0 | 0 | 0.288454036 | 0.231063329 | NA | NA | 0.288454036 | 0.231063329 | NA | NA | NA | NA |
| AQE vs CON | 1 | 0.106513609 | 0.886160367 | 0.192337416 | 0.75272248 | 0.589333634 | 0.902067662 | 0.2034791 | -0.149345182 | 0.623472434 | -0.239537747 | 0.810688631 |
| AQE vs CPP | 3 | 0.438783866 | 0.56352422 | 0.211254239 | 0.7640356 | 0.318918789 | 0.406755458 | 0.281994318 | 0.357280142 | 0.425711157 | 0.839254823 | 0.401326329 |
| AQE vs Dance | 0 | 0 | 0.560358879 | 0.290394104 | NA | NA | 0.560358879 | 0.290394104 | NA | NA | NA | NA |
| AQE vs DT_BGT | 0 | 0 | 0.232023478 | 0.283028593 | NA | NA | 0.232023478 | 0.283028593 | NA | NA | NA | NA |
| AQE vs Mul_C | 4 | 0.4379188 | 0.12299899 | 0.191784329 | 0.19371405 | 0.289811972 | 0.067904718 | 0.255807684 | 0.125809331 | 0.386559892 | 0.325458833 | 0.744833835 |
| AQE vs Mul_D | 1 | 0.626540759 | 0.229566919 | 0.394812445 | 0.09824383 | 0.498788198 | 0.449883513 | 0.646054609 | -0.351639683 | 0.816196192 | -0.430827399 | 0.666593876 |
| AQE vs NW | 0 | 0 | 0.950104771 | 0.338672242 | NA | NA | 0.950104771 | 0.338672242 | NA | NA | NA | NA |
| AQE vs Pilates | 0 | 0 | -0.538535373 | 0.46023091 | NA | NA | -0.538535373 | 0.46023091 | NA | NA | NA | NA |
| AQE vs PT | 0 | 0 | 0.233084948 | 0.36537207 | NA | NA | 0.233084948 | 0.36537207 | NA | NA | NA | NA |
| AQE vs Qigong | 0 | 0 | 0.057217716 | 0.313133053 | NA | NA | 0.057217716 | 0.313133053 | NA | NA | NA | NA |
| AQE vs RA_GT | 0 | 0 | 0.513353442 | 0.330142753 | NA | NA | 0.513353442 | 0.330142753 | NA | NA | NA | NA |
| AQE vs RT | 0 | 0 | 0.266508846 | 0.208907615 | NA | NA | 0.266508846 | 0.208907615 | NA | NA | NA | NA |
| AQE vs Stretch | 0 | 0 | 0.811338836 | 0.265990904 | NA | NA | 0.811338836 | 0.265990904 | NA | NA | NA | NA |
| AQE vs Tai Chi | 0 | 0 | 0.148599665 | 0.262772654 | NA | NA | 0.148599665 | 0.262772654 | NA | NA | NA | NA |
| AQE vs Tango | 0 | 0 | 0.116790199 | 0.32304538 | NA | NA | 0.116790199 | 0.32304538 | NA | NA | NA | NA |
| AQE vs TT | 0 | 0 | 0.489918532 | 0.244535233 | NA | NA | 0.489918532 | 0.244535233 | NA | NA | NA | NA |
| AQE vs VR | 0 | 0 | 0.379937099 | 0.25668279 | NA | NA | 0.379937099 | 0.25668279 | NA | NA | NA | NA |
| AQE vs WBV | 0 | 0 | 0.309550788 | 0.665469441 | NA | NA | 0.309550788 | 0.665469441 | NA | NA | NA | NA |
| AQE vs Yoga | 0 | 0 | 0.35771593 | 0.300046946 | NA | NA | 0.35771593 | 0.300046946 | NA | NA | NA | NA |
| BGT vs BGT_ECA | 4 | 0.389793141 | -0.073850195 | 0.167294123 | -0.455805598 | 0.267956064 | 0.170138535 | 0.214161866 | -0.625944133 | 0.343024426 | -1.824780059 | 0.068034226 |
| BGT vs CON | 3 | 0.17972238 | 0.523856137 | 0.138446715 | 0.642790426 | 0.326573977 | 0.497797699 | 0.152862946 | 0.144992727 | 0.360579593 | 0.402110186 | 0.687602937 |
| BGT vs CPP | 0 | 0 | 0.201219989 | 0.200871214 | NA | NA | 0.201219989 | 0.200871214 | NA | NA | NA | NA |
| BGT vs Dance | 0 | 0 | 0.198054648 | 0.260641471 | NA | NA | 0.198054648 | 0.260641471 | NA | NA | NA | NA |
| BGT vs DT_BGT | 3 | 0.321700566 | -0.130280753 | 0.230328104 | -0.462076484 | 0.406088807 | 0.027081718 | 0.279663768 | -0.489158202 | 0.493071945 | -0.992062532 | 0.321167022 |
| BGT vs Mul_C | 3 | 0.304216016 | -0.239305241 | 0.153180666 | -0.188917947 | 0.277723683 | -0.261335961 | 0.18363977 | 0.072418014 | 0.332947457 | 0.217505834 | 0.827814161 |
| BGT vs Mul_D | 0 | 0 | -0.132737312 | 0.420268357 | NA | NA | -0.132737312 | 0.420268357 | NA | NA | NA | NA |
| BGT vs NW | 1 | 0.229477755 | 0.58780054 | 0.30201275 | 2.4302097 | 0.63045629 | 0.039092284 | 0.344058813 | 2.391117416 | 0.718228098 | 3.329189465 | 0.000870991 |
| BGT vs Pilates | 0 | 0 | -0.900839604 | 0.442919 | NA | NA | -0.900839604 | 0.442919 | NA | NA | NA | NA |
| BGT vs PT | 0 | 0 | -0.129219283 | 0.342495443 | NA | NA | -0.129219283 | 0.342495443 | NA | NA | NA | NA |
| BGT vs Qigong | 0 | 0 | -0.305086515 | 0.289860938 | NA | NA | -0.305086515 | 0.289860938 | NA | NA | NA | NA |
| BGT vs RA_GT | 0 | 0 | 0.151049211 | 0.29909536 | NA | NA | 0.151049211 | 0.29909536 | NA | NA | NA | NA |
| BGT vs RT | 3 | 0.218634876 | -0.095795385 | 0.152656036 | 0.013585472 | 0.326478254 | -0.126401397 | 0.172697865 | 0.139986869 | 0.369340768 | 0.379018189 | 0.704674357 |
| BGT vs Stretch | 0 | 0 | 0.449034605 | 0.220601529 | NA | NA | 0.449034605 | 0.220601529 | NA | NA | NA | NA |
| BGT vs Tai Chi | 1 | 0.12280874 | -0.213704566 | 0.220211684 | -0.2056683 | 0.628384872 | -0.214829661 | 0.235121969 | 0.009161361 | 0.670932104 | 0.013654677 | 0.989105483 |
| BGT vs Tango | 0 | 0 | -0.245514032 | 0.294215099 | NA | NA | -0.245514032 | 0.294215099 | NA | NA | NA | NA |
| BGT vs TT | 0 | 0 | 0.127614301 | 0.194985344 | NA | NA | 0.127614301 | 0.194985344 | NA | NA | NA | NA |
| BGT vs VR | 2 | 0.234156817 | 0.017632868 | 0.204821254 | 0.001968221 | 0.423274025 | 0.022422339 | 0.234048087 | -0.020454118 | 0.483672831 | -0.04228916 | 0.966268186 |
| BGT vs WBV | 0 | 0 | -0.052753443 | 0.644822012 | NA | NA | -0.052753443 | 0.644822012 | NA | NA | NA | NA |
| BGT vs Yoga | 2 | 0.37923893 | -0.004588301 | 0.252642352 | 0.015709352 | 0.410251049 | -0.01698866 | 0.320659359 | 0.032698012 | 0.520699863 | 0.062796275 | 0.949928732 |
| BGT_ECA vs CON | 5 | 0.489545814 | 0.597706331 | 0.149400552 | 0.245855754 | 0.213528344 | 0.935144993 | 0.209109526 | -0.689289239 | 0.298866438 | -2.306345419 | 0.021091339 |
| BGT_ECA vs CPP | 0 | 0 | 0.275070184 | 0.212765649 | NA | NA | 0.275070184 | 0.212765649 | NA | NA | NA | NA |
| BGT_ECA vs Dance | 0 | 0 | 0.271904842 | 0.267116994 | NA | NA | 0.271904842 | 0.267116994 | NA | NA | NA | NA |
| BGT_ECA vs DT_BGT | 0 | 0 | -0.056430558 | 0.249458707 | NA | NA | -0.056430558 | 0.249458707 | NA | NA | NA | NA |
| BGT_ECA vs Mul_C | 1 | 0.090490852 | -0.165455046 | 0.169573509 | 0.38949479 | 0.563709909 | -0.220669316 | 0.1778093 | 0.610164106 | 0.591087987 | 1.032272893 | 0.301944296 |
| BGT_ECA vs Mul_D | 0 | 0 | -0.058887117 | 0.426912247 | NA | NA | -0.058887117 | 0.426912247 | NA | NA | NA | NA |
| BGT_ECA vs NW | 0 | 0 | 0.661650735 | 0.314302807 | NA | NA | 0.661650735 | 0.314302807 | NA | NA | NA | NA |
| BGT_ECA vs Pilates | 0 | 0 | -0.826989409 | 0.447625945 | NA | NA | -0.826989409 | 0.447625945 | NA | NA | NA | NA |
| BGT_ECA vs PT | 0 | 0 | -0.055369089 | 0.350100719 | NA | NA | -0.055369089 | 0.350100719 | NA | NA | NA | NA |
| BGT_ECA vs Qigong | 0 | 0 | -0.23123632 | 0.296086386 | NA | NA | -0.23123632 | 0.296086386 | NA | NA | NA | NA |
| BGT_ECA vs RA_GT | 0 | 0 | 0.224899406 | 0.30374636 | NA | NA | 0.224899406 | 0.30374636 | NA | NA | NA | NA |
| BGT_ECA vs RT | 1 | 0.140002368 | -0.02194519 | 0.168076064 | -0.1385256 | 0.449198379 | -0.002966611 | 0.181241331 | -0.135558989 | 0.484383736 | -0.279858673 | 0.779585935 |
| BGT_ECA vs Stretch | 0 | 0 | 0.5228848 | 0.233265204 | NA | NA | 0.5228848 | 0.233265204 | NA | NA | NA | NA |
| BGT_ECA vs Tai Chi | 0 | 0 | -0.139854371 | 0.233603542 | NA | NA | -0.139854371 | 0.233603542 | NA | NA | NA | NA |
| BGT_ECA vs Tango | 0 | 0 | -0.171663837 | 0.300132159 | NA | NA | -0.171663837 | 0.300132159 | NA | NA | NA | NA |
| BGT_ECA vs TT | 3 | 0.329546804 | 0.201464495 | 0.191030919 | 0.314473846 | 0.332770725 | 0.145917187 | 0.233302521 | 0.168556659 | 0.406406719 | 0.414748701 | 0.678325876 |
| BGT_ECA vs VR | 1 | 0.133431974 | 0.091483062 | 0.217554516 | 0.2072099 | 0.595577315 | 0.073663734 | 0.233704337 | 0.133546166 | 0.639789071 | 0.208734679 | 0.834655367 |
| BGT_ECA vs WBV | 0 | 0 | 0.021096752 | 0.651900329 | NA | NA | 0.021096752 | 0.651900329 | NA | NA | NA | NA |
| BGT_ECA vs Yoga | 0 | 0 | 0.069261894 | 0.273280096 | NA | NA | 0.069261894 | 0.273280096 | NA | NA | NA | NA |
| CPP vs CON | 2 | 0.200213057 | 0.322636148 | 0.167132706 | 0.169049034 | 0.373521191 | 0.361084069 | 0.186884933 | -0.192035035 | 0.417665008 | -0.459782436 | 0.645672391 |
| Dance vs CON | 5 | 0.787635711 | 0.325801489 | 0.223174241 | 0.348061751 | 0.251467212 | 0.243240633 | 0.484287571 | 0.104821118 | 0.545683251 | 0.192091507 | 0.847670528 |
| DT_BGT vs CON | 1 | 0.179164858 | 0.654136889 | 0.22423001 | 0.10196528 | 0.529745554 | 0.774660175 | 0.247494621 | -0.672694895 | 0.584708422 | -1.150479229 | 0.249946544 |
| Mul_C vs CON | 16 | 0.661484542 | 0.763161378 | 0.108191406 | 0.971914699 | 0.133024892 | 0.355241764 | 0.185953138 | 0.616672935 | 0.228635499 | 2.69718805 | 0.006992777 |
| Mul_D vs CON | 1 | 0.432473272 | 0.656593449 | 0.404359229 | 0.45702853 | 0.614876258 | 0.808668213 | 0.536752653 | -0.351639683 | 0.816196192 | -0.430827399 | 0.666593876 |
| NW vs CON | 2 | 0.516925395 | -0.063944404 | 0.288451639 | 0.219939829 | 0.401198293 | -0.367721435 | 0.415017019 | 0.587661265 | 0.577234091 | 1.018064029 | 0.308647528 |
| Pilates vs CON | 1 | 0.522114513 | 1.42469574 | 0.424981614 | 1.4661821 | 0.588148859 | 1.379369755 | 0.614763707 | 0.086812345 | 0.850795801 | 0.10203664 | 0.918727592 |
| PT vs CON | 2 | 0.658579055 | 0.65307542 | 0.320613724 | 0.616628665 | 0.395073728 | 0.723378867 | 0.548702612 | -0.106750203 | 0.67613446 | -0.157883097 | 0.874548917 |
| Qigong vs CON | 3 | 0.744422554 | 0.828942652 | 0.258345401 | 1.008749308 | 0.299427009 | 0.305218306 | 0.511021852 | 0.703531003 | 0.592283603 | 1.187827923 | 0.234901211 |
| RA_GT vs CON | 0 | 0 | 0.372806925 | 0.285128042 | NA | NA | 0.372806925 | 0.285128042 | NA | NA | NA | NA |
| RT vs CON | 16 | 0.593632793 | 0.619651521 | 0.10860473 | 0.758262965 | 0.140958022 | 0.417163975 | 0.170368544 | 0.34109899 | 0.221121244 | 1.542588053 | 0.122930759 |
| Stretch vs CON | 0 | 0 | 0.074821531 | 0.203094459 | NA | NA | 0.074821531 | 0.203094459 | NA | NA | NA | NA |
| Tai Chi vs CON | 3 | 0.391695068 | 0.737560702 | 0.191656779 | 0.271846491 | 0.306231692 | 1.037439843 | 0.245733019 | -0.765593351 | 0.392635411 | -1.949883608 | 0.051189993 |
| Tango vs CON | 3 | 0.582637278 | 0.769370168 | 0.262530371 | 0.471067259 | 0.343938203 | 1.185800256 | 0.406371015 | -0.714732997 | 0.532382278 | -1.342518388 | 0.179427972 |
| TT vs CON | 3 | 0.244728299 | 0.396241836 | 0.169414715 | 0.281660274 | 0.342459355 | 0.433369337 | 0.194939355 | -0.151709063 | 0.394055532 | -0.384994121 | 0.700241771 |
| VR vs CON | 2 | 0.235283909 | 0.506223269 | 0.19149169 | 0.531525226 | 0.394778796 | 0.498438493 | 0.218977663 | 0.033086733 | 0.451443811 | 0.073290921 | 0.941574616 |
| WBV vs CON | 0 | 0 | 0.576609579 | 0.642669036 | NA | NA | 0.576609579 | 0.642669036 | NA | NA | NA | NA |
| Yoga vs CON | 2 | 0.340399254 | 0.528444437 | 0.241377705 | 0.544406844 | 0.413716511 | 0.52020674 | 0.297205324 | 0.024200104 | 0.509403922 | 0.047506708 | 0.962109384 |
| CPP vs Dance | 1 | 0.238501554 | -0.003165341 | 0.266721334 | -0.30661307 | 0.546150263 | 0.091874584 | 0.305649186 | -0.398487654 | 0.625860636 | -0.636703494 | 0.524317997 |
| CPP vs DT_BGT | 0 | 0 | -0.331500742 | 0.263704579 | NA | NA | -0.331500742 | 0.263704579 | NA | NA | NA | NA |
| CPP vs Mul_C | 1 | 0.092803571 | -0.44052523 | 0.183897884 | 1.0025423 | 0.603662764 | -0.588146865 | 0.19307501 | 1.590689165 | 0.633787576 | 2.509814367 | 0.012079464 |
| CPP vs Mul_D | 0 | 0 | -0.333957301 | 0.423316914 | NA | NA | -0.333957301 | 0.423316914 | NA | NA | NA | NA |
| CPP vs NW | 0 | 0 | 0.386580551 | 0.327186901 | NA | NA | 0.386580551 | 0.327186901 | NA | NA | NA | NA |
| CPP vs Pilates | 0 | 0 | -1.102059593 | 0.45355792 | NA | NA | -1.102059593 | 0.45355792 | NA | NA | NA | NA |
| CPP vs PT | 1 | 0.298915724 | -0.330439272 | 0.339335727 | -0.37379366 | 0.620662071 | -0.311954607 | 0.405270016 | -0.061839053 | 0.741259193 | -0.083424332 | 0.933514142 |
| CPP vs Qigong | 1 | 0.342815494 | -0.506306504 | 0.281127667 | -0.04395683 | 0.480146058 | -0.747487833 | 0.346784702 | 0.703531003 | 0.592283603 | 1.187827923 | 0.234901211 |
| CPP vs RA_GT | 1 | 0.299285166 | -0.050170778 | 0.301677314 | -0.41449099 | 0.551442275 | 0.105435511 | 0.36038939 | -0.519926501 | 0.658763308 | -0.789246297 | 0.429968067 |
| CPP vs RT | 3 | 0.338925953 | -0.297015374 | 0.176336306 | -0.427409199 | 0.302893062 | -0.230163783 | 0.216878591 | -0.197245416 | 0.37253259 | -0.529471573 | 0.596478359 |
| CPP vs Stretch | 0 | 0 | 0.247814616 | 0.247163905 | NA | NA | 0.247814616 | 0.247163905 | NA | NA | NA | NA |
| CPP vs Tai Chi | 0 | 0 | -0.414924555 | 0.244492079 | NA | NA | -0.414924555 | 0.244492079 | NA | NA | NA | NA |
| CPP vs Tango | 0 | 0 | -0.446734021 | 0.305789329 | NA | NA | -0.446734021 | 0.305789329 | NA | NA | NA | NA |
| CPP vs TT | 0 | 0 | -0.073605688 | 0.220954524 | NA | NA | -0.073605688 | 0.220954524 | NA | NA | NA | NA |
| CPP vs VR | 1 | 0.171246033 | -0.183587121 | 0.232154907 | -0.1012161 | 0.56100613 | -0.200607505 | 0.255014607 | 0.099391405 | 0.61624697 | 0.161285021 | 0.87186892 |
| CPP vs WBV | 0 | 0 | -0.253973432 | 0.657483458 | NA | NA | -0.253973432 | 0.657483458 | NA | NA | NA | NA |
| CPP vs Yoga | 0 | 0 | -0.20580829 | 0.282532991 | NA | NA | -0.20580829 | 0.282532991 | NA | NA | NA | NA |
| Dance vs DT_BGT | 0 | 0 | -0.328335401 | 0.314388103 | NA | NA | -0.328335401 | 0.314388103 | NA | NA | NA | NA |
| Dance vs Mul_C | 0 | 0 | -0.437359889 | 0.246467421 | NA | NA | -0.437359889 | 0.246467421 | NA | NA | NA | NA |
| Dance vs Mul_D | 0 | 0 | -0.33079196 | 0.460241682 | NA | NA | -0.33079196 | 0.460241682 | NA | NA | NA | NA |
| Dance vs NW | 0 | 0 | 0.389745893 | 0.363883457 | NA | NA | 0.389745893 | 0.363883457 | NA | NA | NA | NA |
| Dance vs Pilates | 0 | 0 | -1.098894252 | 0.479628989 | NA | NA | -1.098894252 | 0.479628989 | NA | NA | NA | NA |
| Dance vs PT | 0 | 0 | -0.327273931 | 0.388189037 | NA | NA | -0.327273931 | 0.388189037 | NA | NA | NA | NA |
| Dance vs Qigong | 0 | 0 | -0.503141163 | 0.338675524 | NA | NA | -0.503141163 | 0.338675524 | NA | NA | NA | NA |
| Dance vs RA_GT | 0 | 0 | -0.047005436 | 0.358830434 | NA | NA | -0.047005436 | 0.358830434 | NA | NA | NA | NA |
| Dance vs RT | 0 | 0 | -0.293850033 | 0.244469603 | NA | NA | -0.293850033 | 0.244469603 | NA | NA | NA | NA |
| Dance vs Stretch | 0 | 0 | 0.250979958 | 0.299503307 | NA | NA | 0.250979958 | 0.299503307 | NA | NA | NA | NA |
| Dance vs Tai Chi | 0 | 0 | -0.411759214 | 0.292637582 | NA | NA | -0.411759214 | 0.292637582 | NA | NA | NA | NA |
| Dance vs Tango | 2 | 0.414464305 | -0.44356868 | 0.297620455 | -0.652626212 | 0.462295015 | -0.295589853 | 0.388943116 | -0.357036359 | 0.60414686 | -0.590976107 | 0.55453643 |
| Dance vs TT | 0 | 0 | -0.070440347 | 0.278079024 | NA | NA | -0.070440347 | 0.278079024 | NA | NA | NA | NA |
| Dance vs VR | 0 | 0 | -0.18042178 | 0.291575443 | NA | NA | -0.18042178 | 0.291575443 | NA | NA | NA | NA |
| Dance vs WBV | 0 | 0 | -0.250808091 | 0.679399935 | NA | NA | -0.250808091 | 0.679399935 | NA | NA | NA | NA |
| Dance vs Yoga | 0 | 0 | -0.202642949 | 0.32710645 | NA | NA | -0.202642949 | 0.32710645 | NA | NA | NA | NA |
| DT_BGT vs Mul_C | 0 | 0 | -0.109024488 | 0.238798606 | NA | NA | -0.109024488 | 0.238798606 | NA | NA | NA | NA |
| DT_BGT vs Mul_D | 0 | 0 | -0.002456559 | 0.457670809 | NA | NA | -0.002456559 | 0.457670809 | NA | NA | NA | NA |
| DT_BGT vs NW | 0 | 0 | 0.718081293 | 0.353385889 | NA | NA | 0.718081293 | 0.353385889 | NA | NA | NA | NA |
| DT_BGT vs Pilates | 0 | 0 | -0.770558851 | 0.477994434 | NA | NA | -0.770558851 | 0.477994434 | NA | NA | NA | NA |
| DT_BGT vs PT | 0 | 0 | 0.00106147 | 0.386340524 | NA | NA | 0.00106147 | 0.386340524 | NA | NA | NA | NA |
| DT_BGT vs Qigong | 0 | 0 | -0.174805762 | 0.338516101 | NA | NA | -0.174805762 | 0.338516101 | NA | NA | NA | NA |
| DT_BGT vs RA_GT | 1 | 0.392436484 | 0.281329964 | 0.301935191 | 0.0768314 | 0.481980031 | 0.413419359 | 0.387362781 | -0.336587959 | 0.618348344 | -0.54433389 | 0.58621173 |
| DT_BGT vs RT | 0 | 0 | 0.034485368 | 0.233873327 | NA | NA | 0.034485368 | 0.233873327 | NA | NA | NA | NA |
| DT_BGT vs Stretch | 1 | 0.289006527 | 0.579315358 | 0.259304452 | 0.50006406 | 0.482343186 | 0.611529638 | 0.307522691 | -0.111465578 | 0.572035973 | -0.194857637 | 0.845504399 |
| DT_BGT vs Tai Chi | 0 | 0 | -0.083423813 | 0.283216378 | NA | NA | -0.083423813 | 0.283216378 | NA | NA | NA | NA |
| DT_BGT vs Tango | 0 | 0 | -0.115233279 | 0.343011811 | NA | NA | -0.115233279 | 0.343011811 | NA | NA | NA | NA |
| DT_BGT vs TT | 1 | 0.202836509 | 0.257895054 | 0.240874521 | 0.7667866 | 0.534832511 | 0.128408711 | 0.269784605 | 0.638377889 | 0.59902383 | 1.065696985 | 0.286560641 |
| DT_BGT vs VR | 1 | 0.219849387 | 0.14791362 | 0.253797846 | -0.02861374 | 0.541284141 | 0.197659697 | 0.287341732 | -0.226273437 | 0.612824438 | -0.36923044 | 0.711955969 |
| DT_BGT vs WBV | 1 | 1 | 0.07752731 | 0.602282651 | 0.07752731 | 0.602282651 | NA | NA | NA | NA | NA | NA |
| DT_BGT vs Yoga | 0 | 0 | 0.125692452 | 0.317964979 | NA | NA | 0.125692452 | 0.317964979 | NA | NA | NA | NA |
| Mul_C vs Mul_D | 0 | 0 | 0.106567929 | 0.409907175 | NA | NA | 0.106567929 | 0.409907175 | NA | NA | NA | NA |
| Mul_C vs NW | 1 | 0.314483973 | 0.827105782 | 0.296827488 | 0.7913827 | 0.529303613 | 0.843493927 | 0.358505077 | -0.052111227 | 0.639287263 | -0.081514572 | 0.935032737 |
| Mul_C vs Pilates | 1 | 0.494326726 | -0.661534363 | 0.425370516 | -0.61763568 | 0.605006915 | -0.704448025 | 0.598180682 | 0.086812345 | 0.850795801 | 0.10203664 | 0.918727592 |
| Mul_C vs PT | 0 | 0 | 0.110085958 | 0.335049217 | NA | NA | 0.110085958 | 0.335049217 | NA | NA | NA | NA |
| Mul_C vs Qigong | 0 | 0 | -0.065781274 | 0.277160637 | NA | NA | -0.065781274 | 0.277160637 | NA | NA | NA | NA |
| Mul_C vs RA_GT | 0 | 0 | 0.390354452 | 0.29702491 | NA | NA | 0.390354452 | 0.29702491 | NA | NA | NA | NA |
| Mul_C vs RT | 0 | 0 | 0.143509856 | 0.143055557 | NA | NA | 0.143509856 | 0.143055557 | NA | NA | NA | NA |
| Mul_C vs Stretch | 0 | 0 | 0.688339847 | 0.21735037 | NA | NA | 0.688339847 | 0.21735037 | NA | NA | NA | NA |
| Mul_C vs Tai Chi | 1 | 0.159698555 | 0.025600675 | 0.208320588 | 0.0821244 | 0.521292768 | 0.014858391 | 0.227255623 | 0.067266009 | 0.568675011 | 0.118285502 | 0.905841444 |
| Mul_C vs Tango | 0 | 0 | -0.006208791 | 0.282694687 | NA | NA | -0.006208791 | 0.282694687 | NA | NA | NA | NA |
| Mul_C vs TT | 0 | 0 | 0.366919542 | 0.190146121 | NA | NA | 0.366919542 | 0.190146121 | NA | NA | NA | NA |
| Mul_C vs VR | 1 | 0.119110249 | 0.256938109 | 0.206352582 | -1.0949349 | 0.597909344 | 0.439732755 | 0.21986147 | -1.534667655 | 0.637051529 | -2.409016518 | 0.015995574 |
| Mul_C vs WBV | 0 | 0 | 0.186551798 | 0.647895954 | NA | NA | 0.186551798 | 0.647895954 | NA | NA | NA | NA |
| Mul_C vs Yoga | 0 | 0 | 0.23471694 | 0.257776078 | NA | NA | 0.23471694 | 0.257776078 | NA | NA | NA | NA |
| Mul_D vs NW | 0 | 0 | 0.720537853 | 0.493349872 | NA | NA | 0.720537853 | 0.493349872 | NA | NA | NA | NA |
| Mul_D vs Pilates | 0 | 0 | -0.768102292 | 0.58362947 | NA | NA | -0.768102292 | 0.58362947 | NA | NA | NA | NA |
| Mul_D vs PT | 0 | 0 | 0.003518029 | 0.512353267 | NA | NA | 0.003518029 | 0.512353267 | NA | NA | NA | NA |
| Mul_D vs Qigong | 0 | 0 | -0.172349203 | 0.476257044 | NA | NA | -0.172349203 | 0.476257044 | NA | NA | NA | NA |
| Mul_D vs RA_GT | 0 | 0 | 0.283786523 | 0.489077621 | NA | NA | 0.283786523 | 0.489077621 | NA | NA | NA | NA |
| Mul_D vs RT | 0 | 0 | 0.036941927 | 0.414966932 | NA | NA | 0.036941927 | 0.414966932 | NA | NA | NA | NA |
| Mul_D vs Stretch | 0 | 0 | 0.581771918 | 0.447477882 | NA | NA | 0.581771918 | 0.447477882 | NA | NA | NA | NA |
| Mul_D vs Tai Chi | 0 | 0 | -0.080967254 | 0.444317678 | NA | NA | -0.080967254 | 0.444317678 | NA | NA | NA | NA |
| Mul_D vs Tango | 0 | 0 | -0.11277672 | 0.481133233 | NA | NA | -0.11277672 | 0.481133233 | NA | NA | NA | NA |
| Mul_D vs TT | 0 | 0 | 0.260351613 | 0.434339508 | NA | NA | 0.260351613 | 0.434339508 | NA | NA | NA | NA |
| Mul_D vs VR | 0 | 0 | 0.15037018 | 0.44213706 | NA | NA | 0.15037018 | 0.44213706 | NA | NA | NA | NA |
| Mul_D vs WBV | 0 | 0 | 0.079983869 | 0.756443628 | NA | NA | 0.079983869 | 0.756443628 | NA | NA | NA | NA |
| Mul_D vs Yoga | 0 | 0 | 0.128149011 | 0.467563951 | NA | NA | 0.128149011 | 0.467563951 | NA | NA | NA | NA |
| NW vs Pilates | 0 | 0 | -1.488640144 | 0.510400076 | NA | NA | -1.488640144 | 0.510400076 | NA | NA | NA | NA |
| NW vs PT | 0 | 0 | -0.717019824 | 0.428914867 | NA | NA | -0.717019824 | 0.428914867 | NA | NA | NA | NA |
| NW vs Qigong | 0 | 0 | -0.892887056 | 0.385749242 | NA | NA | -0.892887056 | 0.385749242 | NA | NA | NA | NA |
| NW vs RA_GT | 0 | 0 | -0.436751329 | 0.392064532 | NA | NA | -0.436751329 | 0.392064532 | NA | NA | NA | NA |
| NW vs RT | 0 | 0 | -0.683595925 | 0.301949678 | NA | NA | -0.683595925 | 0.301949678 | NA | NA | NA | NA |
| NW vs Stretch | 0 | 0 | -0.138765935 | 0.344257292 | NA | NA | -0.138765935 | 0.344257292 | NA | NA | NA | NA |
| NW vs Tai Chi | 0 | 0 | -0.801505106 | 0.341210128 | NA | NA | -0.801505106 | 0.341210128 | NA | NA | NA | NA |
| NW vs Tango | 0 | 0 | -0.833314572 | 0.389023922 | NA | NA | -0.833314572 | 0.389023922 | NA | NA | NA | NA |
| NW vs TT | 1 | 0.25339664 | -0.46018624 | 0.311704089 | 0.59961577 | 0.619215863 | -0.819882274 | 0.360742681 | 1.419498044 | 0.716633495 | 1.980786626 | 0.047615207 |
| NW vs VR | 0 | 0 | -0.570167673 | 0.33545892 | NA | NA | -0.570167673 | 0.33545892 | NA | NA | NA | NA |
| NW vs WBV | 0 | 0 | -0.640553983 | 0.698302212 | NA | NA | -0.640553983 | 0.698302212 | NA | NA | NA | NA |
| NW vs Yoga | 0 | 0 | -0.592388841 | 0.369980112 | NA | NA | -0.592388841 | 0.369980112 | NA | NA | NA | NA |
| Pilates vs PT | 0 | 0 | 0.771620321 | 0.531332195 | NA | NA | 0.771620321 | 0.531332195 | NA | NA | NA | NA |
| Pilates vs Qigong | 0 | 0 | 0.595753089 | 0.496547969 | NA | NA | 0.595753089 | 0.496547969 | NA | NA | NA | NA |
| Pilates vs RA_GT | 0 | 0 | 1.051888815 | 0.509494944 | NA | NA | 1.051888815 | 0.509494944 | NA | NA | NA | NA |
| Pilates vs RT | 0 | 0 | 0.805044219 | 0.436954805 | NA | NA | 0.805044219 | 0.436954805 | NA | NA | NA | NA |
| Pilates vs Stretch | 0 | 0 | 1.349874209 | 0.468061572 | NA | NA | 1.349874209 | 0.468061572 | NA | NA | NA | NA |
| Pilates vs Tai Chi | 0 | 0 | 0.687135038 | 0.463565287 | NA | NA | 0.687135038 | 0.463565287 | NA | NA | NA | NA |
| Pilates vs Tango | 0 | 0 | 0.655325572 | 0.499185307 | NA | NA | 0.655325572 | 0.499185307 | NA | NA | NA | NA |
| Pilates vs TT | 0 | 0 | 1.028453905 | 0.455241893 | NA | NA | 1.028453905 | 0.455241893 | NA | NA | NA | NA |
| Pilates vs VR | 0 | 0 | 0.918472471 | 0.463102303 | NA | NA | 0.918472471 | 0.463102303 | NA | NA | NA | NA |
| Pilates vs WBV | 0 | 0 | 0.848086161 | 0.768910314 | NA | NA | 0.848086161 | 0.768910314 | NA | NA | NA | NA |
| Pilates vs Yoga | 0 | 0 | 0.896251303 | 0.486993078 | NA | NA | 0.896251303 | 0.486993078 | NA | NA | NA | NA |
| PT vs Qigong | 0 | 0 | -0.175867232 | 0.406415626 | NA | NA | -0.175867232 | 0.406415626 | NA | NA | NA | NA |
| PT vs RA_GT | 0 | 0 | 0.280268495 | 0.422013063 | NA | NA | 0.280268495 | 0.422013063 | NA | NA | NA | NA |
| PT vs RT | 0 | 0 | 0.033423898 | 0.332118327 | NA | NA | 0.033423898 | 0.332118327 | NA | NA | NA | NA |
| PT vs Stretch | 0 | 0 | 0.578253889 | 0.374476135 | NA | NA | 0.578253889 | 0.374476135 | NA | NA | NA | NA |
| PT vs Tai Chi | 0 | 0 | -0.084485283 | 0.368488217 | NA | NA | -0.084485283 | 0.368488217 | NA | NA | NA | NA |
| PT vs Tango | 0 | 0 | -0.116294749 | 0.412871648 | NA | NA | -0.116294749 | 0.412871648 | NA | NA | NA | NA |
| PT vs TT | 0 | 0 | 0.256833584 | 0.358164611 | NA | NA | 0.256833584 | 0.358164611 | NA | NA | NA | NA |
| PT vs VR | 0 | 0 | 0.146852151 | 0.367830426 | NA | NA | 0.146852151 | 0.367830426 | NA | NA | NA | NA |
| PT vs WBV | 0 | 0 | 0.07646584 | 0.715544124 | NA | NA | 0.07646584 | 0.715544124 | NA | NA | NA | NA |
| PT vs Yoga | 1 | 0.415666404 | 0.124630982 | 0.366386368 | 0.43599788 | 0.568285852 | -0.096860234 | 0.4793016 | 0.532858114 | 0.743423724 | 0.716762321 | 0.473520765 |
| Qigong vs RA_GT | 0 | 0 | 0.456135726 | 0.378071508 | NA | NA | 0.456135726 | 0.378071508 | NA | NA | NA | NA |
| Qigong vs RT | 0 | 0 | 0.20929113 | 0.275898207 | NA | NA | 0.20929113 | 0.275898207 | NA | NA | NA | NA |
| Qigong vs Stretch | 0 | 0 | 0.754121121 | 0.325152689 | NA | NA | 0.754121121 | 0.325152689 | NA | NA | NA | NA |
| Qigong vs Tai Chi | 0 | 0 | 0.091381949 | 0.319539739 | NA | NA | 0.091381949 | 0.319539739 | NA | NA | NA | NA |
| Qigong vs Tango | 0 | 0 | 0.059572483 | 0.367051724 | NA | NA | 0.059572483 | 0.367051724 | NA | NA | NA | NA |
| Qigong vs TT | 0 | 0 | 0.432700816 | 0.305378121 | NA | NA | 0.432700816 | 0.305378121 | NA | NA | NA | NA |
| Qigong vs VR | 0 | 0 | 0.322719383 | 0.31688096 | NA | NA | 0.322719383 | 0.31688096 | NA | NA | NA | NA |
| Qigong vs WBV | 0 | 0 | 0.252333072 | 0.690896188 | NA | NA | 0.252333072 | 0.690896188 | NA | NA | NA | NA |
| Qigong vs Yoga | 0 | 0 | 0.300498214 | 0.351029134 | NA | NA | 0.300498214 | 0.351029134 | NA | NA | NA | NA |
| RA_GT vs RT | 0 | 0 | -0.246844596 | 0.291583953 | NA | NA | -0.246844596 | 0.291583953 | NA | NA | NA | NA |
| RA_GT vs Stretch | 0 | 0 | 0.297985394 | 0.328601809 | NA | NA | 0.297985394 | 0.328601809 | NA | NA | NA | NA |
| RA_GT vs Tai Chi | 0 | 0 | -0.364753777 | 0.335389448 | NA | NA | -0.364753777 | 0.335389448 | NA | NA | NA | NA |
| RA_GT vs Tango | 0 | 0 | -0.396563243 | 0.385014578 | NA | NA | -0.396563243 | 0.385014578 | NA | NA | NA | NA |
| RA_GT vs TT | 2 | 0.581424496 | -0.023434911 | 0.2705344 | -0.28505778 | 0.354793645 | 0.339973707 | 0.41815336 | -0.625031487 | 0.548389244 | -1.139758837 | 0.254386787 |
| RA_GT vs VR | 0 | 0 | -0.133416344 | 0.317779144 | NA | NA | -0.133416344 | 0.317779144 | NA | NA | NA | NA |
| RA_GT vs WBV | 0 | 0 | -0.203802654 | 0.673727877 | NA | NA | -0.203802654 | 0.673727877 | NA | NA | NA | NA |
| RA_GT vs Yoga | 0 | 0 | -0.155637512 | 0.365035724 | NA | NA | -0.155637512 | 0.365035724 | NA | NA | NA | NA |
| RT vs Stretch | 3 | 0.431937045 | 0.54482999 | 0.20247094 | 0.788503557 | 0.308072174 | 0.359548343 | 0.268636157 | 0.428955214 | 0.408746681 | 1.049440239 | 0.293975547 |
| RT vs Tai Chi | 3 | 0.402993483 | -0.117909181 | 0.197280561 | -0.642011118 | 0.310767277 | 0.235871991 | 0.255325833 | -0.87788311 | 0.402203408 | -2.182684414 | 0.029059059 |
| RT vs Tango | 1 | 0.189308223 | -0.149718647 | 0.276447554 | -0.7728335 | 0.635371911 | -0.004212341 | 0.307032866 | -0.768621159 | 0.705667518 | -1.089211476 | 0.276060639 |
| RT vs TT | 2 | 0.245408232 | 0.223409686 | 0.179668911 | -0.185724109 | 0.362683979 | 0.356468123 | 0.206831606 | -0.542192232 | 0.417515248 | -1.298616602 | 0.194075538 |
| RT vs VR | 0 | 0 | 0.113428252 | 0.20371581 | NA | NA | 0.113428252 | 0.20371581 | NA | NA | NA | NA |
| RT vs WBV | 0 | 0 | 0.043041942 | 0.646096839 | NA | NA | 0.043041942 | 0.646096839 | NA | NA | NA | NA |
| RT vs Yoga | 1 | 0.300890919 | 0.091207084 | 0.245740116 | -0.10012163 | 0.447993299 | 0.173553422 | 0.293902708 | -0.273675052 | 0.535795481 | -0.510782681 | 0.609503238 |
| Stretch vs Tai Chi | 1 | 0.311738278 | -0.662739171 | 0.252099824 | -0.74109403 | 0.451520503 | -0.627249462 | 0.303875533 | -0.113844568 | 0.544252794 | -0.209175901 | 0.834310925 |
| Stretch vs Tango | 0 | 0 | -0.694548637 | 0.328408977 | NA | NA | -0.694548637 | 0.328408977 | NA | NA | NA | NA |
| Stretch vs TT | 0 | 0 | -0.321420305 | 0.240768929 | NA | NA | -0.321420305 | 0.240768929 | NA | NA | NA | NA |
| Stretch vs VR | 2 | 0.342104561 | -0.431401738 | 0.233300743 | -0.198444092 | 0.398874882 | -0.552539336 | 0.287632319 | 0.354095244 | 0.491765719 | 0.720048653 | 0.47149504 |
| Stretch vs WBV | 0 | 0 | -0.501788048 | 0.655731036 | NA | NA | -0.501788048 | 0.655731036 | NA | NA | NA | NA |
| Stretch vs Yoga | 0 | 0 | -0.453622906 | 0.30234083 | NA | NA | -0.453622906 | 0.30234083 | NA | NA | NA | NA |
| Tai Chi vs Tango | 0 | 0 | -0.031809466 | 0.322272255 | NA | NA | -0.031809466 | 0.322272255 | NA | NA | NA | NA |
| Tai Chi vs TT | 0 | 0 | 0.341318867 | 0.245557957 | NA | NA | 0.341318867 | 0.245557957 | NA | NA | NA | NA |
| Tai Chi vs VR | 0 | 0 | 0.231337434 | 0.25863876 | NA | NA | 0.231337434 | 0.25863876 | NA | NA | NA | NA |
| Tai Chi vs WBV | 0 | 0 | 0.160951123 | 0.665549329 | NA | NA | 0.160951123 | 0.665549329 | NA | NA | NA | NA |
| Tai Chi vs Yoga | 1 | 0.211312218 | 0.209116265 | 0.288856689 | 0.2046136 | 0.628376774 | 0.210322659 | 0.325259412 | -0.005709059 | 0.707566997 | -0.008068577 | 0.993562276 |
| Tango vs TT | 0 | 0 | 0.373128333 | 0.309783937 | NA | NA | 0.373128333 | 0.309783937 | NA | NA | NA | NA |
| Tango vs VR | 0 | 0 | 0.263146899 | 0.322565728 | NA | NA | 0.263146899 | 0.322565728 | NA | NA | NA | NA |
| Tango vs WBV | 0 | 0 | 0.192760589 | 0.693110016 | NA | NA | 0.192760589 | 0.693110016 | NA | NA | NA | NA |
| Tango vs Yoga | 0 | 0 | 0.240925731 | 0.354019404 | NA | NA | 0.240925731 | 0.354019404 | NA | NA | NA | NA |
| TT vs VR | 1 | 0.238235415 | -0.109981433 | 0.220899624 | -0.14988718 | 0.452576309 | -0.097501249 | 0.253095596 | -0.052385931 | 0.518539002 | -0.101026018 | 0.919529806 |
| TT vs WBV | 0 | 0 | -0.180367744 | 0.648663955 | NA | NA | -0.180367744 | 0.648663955 | NA | NA | NA | NA |
| TT vs Yoga | 0 | 0 | -0.132202602 | 0.285118308 | NA | NA | -0.132202602 | 0.285118308 | NA | NA | NA | NA |
| VR vs WBV | 0 | 0 | -0.07038631 | 0.653573056 | NA | NA | -0.07038631 | 0.653573056 | NA | NA | NA | NA |
| VR vs Yoga | 0 | 0 | -0.022221168 | 0.297094802 | NA | NA | -0.022221168 | 0.297094802 | NA | NA | NA | NA |
| WBV vs Yoga | 0 | 0 | 0.048165142 | 0.681062494 | NA | NA | 0.048165142 | 0.681062494 | NA | NA | NA | NA |

*NA* not available, *k* Number of studies providing direct evidence, *prop* Direct evidence proportion, *nma* Estimated treatment effect (SMD) in network meta-analysis, *direct* Estimated treatment effect (SMD) derived from direct evidence, *indir.* Estimated treatment effect (SMD) derived from indirect evidence, *Diff* Difference between direct and indirect treatment estimates, *z* z-value of test for disagreement (direct versus indirect), *p* p-value of test for disagreement (direct versus indirect).

## Table 7.5 Details of SIDE splitting results (Reactive balance)

| **Comparison** | **k** | **prop** | **NMA** | | **Direc** | | **Indir** | | **Diff** | | **z** | **p** |
| --- | --- | --- | --- | --- | --- | --- | --- | --- | --- | --- | --- | --- |
|  |  |  | **TE** | **seTE** | **TE** | **seTE** | **TE** | **seTE** | **TE** | **seTE** |  |  |
| BGT vs BGT_ECA | 0 | 0 | -1.216225632 | 0.60679408 | NA | NA | -1.216225632 | 0.60679408 | NA | NA | NA | NA |
| BGT vs CON | 0 | 0 | 0.581935458 | 0.372707739 | NA | NA | 0.581935458 | 0.372707739 | NA | NA | NA | NA |
| BGT vs CPP | 0 | 0 | 0.124730408 | 0.458342191 | NA | NA | 0.124730408 | 0.458342191 | NA | NA | NA | NA |
| BGT vs DT_BGT | 0 | 0 | -0.070728802 | 0.444487076 | NA | NA | -0.070728802 | 0.444487076 | NA | NA | NA | NA |
| BGT vs Mul_C | 0 | 0 | -0.032999132 | 0.437602684 | NA | NA | -0.032999132 | 0.437602684 | NA | NA | NA | NA |
| BGT vs PT | 0 | 0 | 0.109466718 | 0.495720862 | NA | NA | 0.109466718 | 0.495720862 | NA | NA | NA | NA |
| BGT vs RA_GT | 0 | 0 | -1.094472292 | 0.609342584 | NA | NA | -1.094472292 | 0.609342584 | NA | NA | NA | NA |
| BGT vs RT | 1 | 1 | 0.031607438 | 0.322507002 | 0.03160744 | 0.322507002 | NA | NA | NA | NA | NA | NA |
| BGT vs Stretch | 0 | 0 | 0.229393708 | 0.44628207 | NA | NA | 0.229393708 | 0.44628207 | NA | NA | NA | NA |
| BGT vs TC | 1 | 1 | -0.036122796 | 0.358197564 | -0.0361228 | 0.358197564 | NA | NA | NA | NA | NA | NA |
| BGT vs VR | 0 | 0 | 0.594071868 | 0.400345421 | NA | NA | 0.594071868 | 0.400345421 | NA | NA | NA | NA |
| BGT vs Yoga | 0 | 0 | 0.462883098 | 0.544816631 | NA | NA | 0.462883098 | 0.544816631 | NA | NA | NA | NA |
| BGT_ECA vs CON | 0 | 0 | 1.79816109 | 0.478840262 | NA | NA | 1.79816109 | 0.478840262 | NA | NA | NA | NA |
| BGT_ECA vs CPP | 0 | 0 | 1.34095604 | 0.548137301 | NA | NA | 1.34095604 | 0.548137301 | NA | NA | NA | NA |
| BGT_ECA vs DT_BGT | 0 | 0 | 1.14549683 | 0.536605719 | NA | NA | 1.14549683 | 0.536605719 | NA | NA | NA | NA |
| BGT_ECA vs Mul_C | 1 | 1 | 1.1832265 | 0.420360495 | 1.1832265 | 0.420360495 | NA | NA | NA | NA | NA | NA |
| BGT_ECA vs PT | 0 | 0 | 1.32569235 | 0.579755216 | NA | NA | 1.32569235 | 0.579755216 | NA | NA | NA | NA |
| BGT_ECA vs RA_GT | 0 | 0 | 0.12175334 | 0.67946694 | NA | NA | 0.12175334 | 0.67946694 | NA | NA | NA | NA |
| BGT_ECA vs RT | 0 | 0 | 1.247833069 | 0.513992499 | NA | NA | 1.247833069 | 0.513992499 | NA | NA | NA | NA |
| BGT_ECA vs Stretch | 0 | 0 | 1.445619339 | 0.599454092 | NA | NA | 1.445619339 | 0.599454092 | NA | NA | NA | NA |
| BGT_ECA vs TC | 0 | 0 | 1.180102835 | 0.630508566 | NA | NA | 1.180102835 | 0.630508566 | NA | NA | NA | NA |
| BGT_ECA vs VR | 0 | 0 | 1.8102975 | 0.530223033 | NA | NA | 1.8102975 | 0.530223033 | NA | NA | NA | NA |
| BGT_ECA vs Yoga | 0 | 0 | 1.67910873 | 0.622255654 | NA | NA | 1.67910873 | 0.622255654 | NA | NA | NA | NA |
| CON vs CPP | 1 | 1 | 0.45720505 | 0.26677051 | 0.45720505 | 0.26677051 | NA | NA | NA | NA | NA | NA |
| CON vs DT_BGT | 1 | 1 | 0.65266426 | 0.242193521 | 0.65266426 | 0.242193521 | NA | NA | NA | NA | NA | NA |
| CON vs Mul_C | 1 | 1 | 0.61493459 | 0.229314305 | 0.61493459 | 0.229314305 | NA | NA | NA | NA | NA | NA |
| CON vs PT | 1 | 1 | 0.47246874 | 0.326845704 | 0.47246874 | 0.326845704 | NA | NA | NA | NA | NA | NA |
| CON vs RA_GT | 0 | 0 | 1.67640775 | 0.482065687 | NA | NA | 1.67640775 | 0.482065687 | NA | NA | NA | NA |
| CON vs RT | 3 | 0.810027007 | 0.550328021 | 0.1868162 | 0.396278114 | 0.207570095 | 1.20718236 | 0.42861622 | -0.810904246 | 0.476232305 | -1.702749347 | 0.088614986 |
| CON vs Stretch | 0 | 0 | 0.352541751 | 0.360634458 | NA | NA | 0.352541751 | 0.360634458 | NA | NA | NA | NA |
| CON vs TC | 0 | 0 | 0.618058255 | 0.410186611 | NA | NA | 0.618058255 | 0.410186611 | NA | NA | NA | NA |
| CON vs VR | 1 | 0.646247802 | -0.01213641 | 0.227702587 | 0.27472275 | 0.283249026 | -0.536181496 | 0.382840955 | 0.810904246 | 0.476232305 | 1.702749347 | 0.088614986 |
| CON vs Yoga | 1 | 1 | 0.11905236 | 0.397384074 | 0.11905236 | 0.397384074 | NA | NA | NA | NA | NA | NA |
| CPP vs DT_BGT | 0 | 0 | -0.19545921 | 0.360311263 | NA | NA | -0.19545921 | 0.360311263 | NA | NA | NA | NA |
| CPP vs Mul_C | 0 | 0 | -0.15772954 | 0.351783392 | NA | NA | -0.15772954 | 0.351783392 | NA | NA | NA | NA |
| CPP vs PT | 0 | 0 | -0.01526369 | 0.421894085 | NA | NA | -0.01526369 | 0.421894085 | NA | NA | NA | NA |
| CPP vs RA_GT | 1 | 1 | -1.2192027 | 0.401523127 | -1.2192027 | 0.401523127 | NA | NA | NA | NA | NA | NA |
| CPP vs RT | 0 | 0 | -0.093122971 | 0.325678979 | NA | NA | -0.093122971 | 0.325678979 | NA | NA | NA | NA |
| CPP vs Stretch | 0 | 0 | 0.104663299 | 0.448579666 | NA | NA | 0.104663299 | 0.448579666 | NA | NA | NA | NA |
| CPP vs TC | 0 | 0 | -0.160853205 | 0.489305181 | NA | NA | -0.160853205 | 0.489305181 | NA | NA | NA | NA |
| CPP vs VR | 0 | 0 | 0.46934146 | 0.350734904 | NA | NA | 0.46934146 | 0.350734904 | NA | NA | NA | NA |
| CPP vs Yoga | 0 | 0 | 0.33815269 | 0.47862366 | NA | NA | 0.33815269 | 0.47862366 | NA | NA | NA | NA |
| DT_BGT vs Mul_C | 0 | 0 | 0.03772967 | 0.333530736 | NA | NA | 0.03772967 | 0.333530736 | NA | NA | NA | NA |
| DT_BGT vs PT | 0 | 0 | 0.18019552 | 0.406799478 | NA | NA | 0.18019552 | 0.406799478 | NA | NA | NA | NA |
| DT_BGT vs RA_GT | 0 | 0 | -1.02374349 | 0.539485892 | NA | NA | -1.02374349 | 0.539485892 | NA | NA | NA | NA |
| DT_BGT vs RT | 0 | 0 | 0.102336239 | 0.305872513 | NA | NA | 0.102336239 | 0.305872513 | NA | NA | NA | NA |
| DT_BGT vs Stretch | 0 | 0 | 0.300122509 | 0.434413298 | NA | NA | 0.300122509 | 0.434413298 | NA | NA | NA | NA |
| DT_BGT vs TC | 0 | 0 | 0.034606005 | 0.476351506 | NA | NA | 0.034606005 | 0.476351506 | NA | NA | NA | NA |
| DT_BGT vs VR | 0 | 0 | 0.66480067 | 0.332424683 | NA | NA | 0.66480067 | 0.332424683 | NA | NA | NA | NA |
| DT_BGT vs Yoga | 0 | 0 | 0.5336119 | 0.465372758 | NA | NA | 0.5336119 | 0.465372758 | NA | NA | NA | NA |
| Mul_C vs PT | 0 | 0 | 0.14246585 | 0.399265782 | NA | NA | 0.14246585 | 0.399265782 | NA | NA | NA | NA |
| Mul_C vs RA_GT | 0 | 0 | -1.06147316 | 0.53382804 | NA | NA | -1.06147316 | 0.53382804 | NA | NA | NA | NA |
| Mul_C vs RT | 0 | 0 | 0.064606569 | 0.295779213 | NA | NA | 0.064606569 | 0.295779213 | NA | NA | NA | NA |
| Mul_C vs Stretch | 0 | 0 | 0.262392839 | 0.42736666 | NA | NA | 0.262392839 | 0.42736666 | NA | NA | NA | NA |
| Mul_C vs TC | 0 | 0 | -0.003123665 | 0.469934151 | NA | NA | -0.003123665 | 0.469934151 | NA | NA | NA | NA |
| Mul_C vs VR | 0 | 0 | 0.627071 | 0.323161753 | NA | NA | 0.627071 | 0.323161753 | NA | NA | NA | NA |
| Mul_C vs Yoga | 0 | 0 | 0.49588223 | 0.458801867 | NA | NA | 0.49588223 | 0.458801867 | NA | NA | NA | NA |
| PT vs RA_GT | 0 | 0 | -1.20393901 | 0.582422047 | NA | NA | -1.20393901 | 0.582422047 | NA | NA | NA | NA |
| PT vs RT | 0 | 0 | -0.077859281 | 0.376468334 | NA | NA | -0.077859281 | 0.376468334 | NA | NA | NA | NA |
| PT vs Stretch | 0 | 0 | 0.119926989 | 0.486708667 | NA | NA | 0.119926989 | 0.486708667 | NA | NA | NA | NA |
| PT vs TC | 0 | 0 | -0.145589515 | 0.524481811 | NA | NA | -0.145589515 | 0.524481811 | NA | NA | NA | NA |
| PT vs VR | 0 | 0 | 0.48460515 | 0.398342293 | NA | NA | 0.48460515 | 0.398342293 | NA | NA | NA | NA |
| PT vs Yoga | 0 | 0 | 0.35341638 | 0.514531065 | NA | NA | 0.35341638 | 0.514531065 | NA | NA | NA | NA |
| RA_GT vs RT | 0 | 0 | 1.126079729 | 0.516998664 | NA | NA | 1.126079729 | 0.516998664 | NA | NA | NA | NA |
| RA_GT vs Stretch | 0 | 0 | 1.323865999 | 0.602033669 | NA | NA | 1.323865999 | 0.602033669 | NA | NA | NA | NA |
| RA_GT vs TC | 0 | 0 | 1.058349495 | 0.632961596 | NA | NA | 1.058349495 | 0.632961596 | NA | NA | NA | NA |
| RA_GT vs VR | 0 | 0 | 1.68854416 | 0.533137688 | NA | NA | 1.68854416 | 0.533137688 | NA | NA | NA | NA |
| RA_GT vs Yoga | 0 | 0 | 1.55735539 | 0.62474109 | NA | NA | 1.55735539 | 0.62474109 | NA | NA | NA | NA |
| RT vs Stretch | 1 | 1 | 0.19778627 | 0.308475152 | 0.19778627 | 0.308475152 | NA | NA | NA | NA | NA | NA |
| RT vs TC | 1 | 1 | -0.067730234 | 0.365174976 | -0.06773023 | 0.365174976 | NA | NA | NA | NA | NA | NA |
| RT vs VR | 1 | 0.543725191 | 0.56246443 | 0.2372039 | 0.93245961 | 0.321685954 | 0.121555364 | 0.351162861 | 0.810904246 | 0.476232305 | 1.702749347 | 0.088614986 |
| RT vs Yoga | 0 | 0 | 0.431275661 | 0.43910636 | NA | NA | 0.431275661 | 0.43910636 | NA | NA | NA | NA |
| Stretch vs TC | 0 | 0 | -0.265516504 | 0.478026864 | NA | NA | -0.265516504 | 0.478026864 | NA | NA | NA | NA |
| Stretch vs VR | 0 | 0 | 0.36467816 | 0.389130582 | NA | NA | 0.36467816 | 0.389130582 | NA | NA | NA | NA |
| Stretch vs Yoga | 0 | 0 | 0.233489391 | 0.536629588 | NA | NA | 0.233489391 | 0.536629588 | NA | NA | NA | NA |
| TC vs VR | 0 | 0 | 0.630194664 | 0.43545201 | NA | NA | 0.630194664 | 0.43545201 | NA | NA | NA | NA |
| TC vs Yoga | 0 | 0 | 0.499005895 | 0.571110461 | NA | NA | 0.499005895 | 0.571110461 | NA | NA | NA | NA |
| VR vs Yoga | 0 | 0 | -0.13118877 | 0.45799844 | NA | NA | -0.13118877 | 0.45799844 | NA | NA | NA | NA |

*NA* not available, *k* Number of studies providing direct evidence, *prop* Direct evidence proportion, *nma* Estimated treatment effect (SMD) in network meta-analysis, *direct* Estimated treatment effect (SMD) derived from direct evidence, *indir.* Estimated treatment effect (SMD) derived from indirect evidence, *Diff* Difference between direct and indirect treatment estimates, *z* z-value of test for disagreement (direct versus indirect), *p* p-value of test for disagreement (direct versus indirect).

# Appendix 8: Sensitivity analyses

We assessed the sensitivity of our findings by repeating each network meta-analysis after excluding studies at overall high risk of bias, exercise duration less than 4 weeks and more than 24 weeks, exercise frequency less than 2 and more than 4, OFF state during the test, and data were extracted using GetData and estimated standard deviations value.

## Table 8.1 Changes in heterogeneity

Below we present the results from the changes in heterogeneity in each sensitivity analysis

| **Including only studies with** | **Number of studies included** | **I^2^** | **Change** |
| --- | --- | --- | --- |
| **Balance test batteries** | | | |
| None | 104 | 36.2% | - |
| studies at overall low to moderate risk of bias | 101 | 35.8% | -0.4% |
| Exercise period 4-24 weeks | 99 | 33.8% | -2.4% |
| Exercise frequency 2-4 | 77 | 34.1% | -2.1% |
| ON state during the test | 94 | 39.3% | 3.1% |
| Original data and standard deviations | 71 | 35% | -1.2% |
| **Static steady-state balance** | | | |
| None | 51 | 56.3% | - |
| studies at overall low to moderate risk of bias | 50 | 51.1% | -5.2% |
| Exercise period 4-24 weeks | 44 | 53.5% | -1.8% |
| Exercise frequency 2-4 | 31 | 47.8% | -8.5% |
| ON state during the test | 49 | 47.7% | -8.6% |
| Original data and standard deviations | 42 | 53.5% | -2.8% |
| **Dynamic steady-state balance** | | | |
| None | 110 | 47.9% | - |
| studies at overall low to moderate risk of bias | 101 | 42.3% | -5.6% |
| Exercise period 4-24 weeks | 99 | 45.2% | -2.7% |
| Exercise frequency 2-4 | 77 | 45.5% | -2.4% |
| ON state during the test | 94 | 41.2% | -6.7% |
| Original data and standard deviations | 102 | 30.9% | -17.0% |
| **Proactive balance** | | | |
| None | 119 | 61.8% | - |
| studies at overall low to moderate risk of bias | 114 | 61.2% | -0.6% |
| Exercise period 4-24 weeks | 93 | 54.9% | -6.9% |
| Exercise frequency 2-4 | 79 | 54.6% | -7.2% |
| ON state during the test | 115 | 61.4% | -0.4% |
| Original data and standard deviations | 102 | 48.2% | -13.6% |
| **Reactive balance** | | | |
| None | 13 | 3.2% | - |
| studies at overall low to moderate risk of bias | - | - | - |
| Exercise period 4-24 weeks | 12 | 3.2% | 0% |
| Exercise frequency 2-4 | 12 | 3.2% | 0% |
| ON state during the test | - | - | - |
| Original data and standard deviations | - | - | - |

## Figure 8.1 Exclude studies at overall high risk of bias (balance test batteries)

After excluding trials with high overall risk of bias, the hierarchy did not change significantly.

Figure 8.1: Forest plot of studies at moderate or low RoB. Exercise type are ranked according to SMD compared to CON. Treatments crossing the y-axis are not significantly different from CON. *SMD* standardized Mean Difference, *CrI* Credible Interval, *AE* Aerobic Exercise, *AQE* Aquatic Exercise, *BGT* Balance and Gait Training, *BGT_ECA* Balance and Gait Training with External Cue or Attention, *BWS_TT* Body Weight Support Treadmill Training, *CON* Control group, *CPP* Classic Physiotherapy Program, *DT_BGT* Dual Task Balance and Gait Training, *Mul_C* Multicomponent Exercise Program, *Mul_D* Multidisciplinary Exercise Program, *RA_GT* Robotic Assisted Gait Training, *RT* Resistance Training, *TC* Tai Chi, *TT* Treadmill Training, *VR* Virtual Reality, *WBV* Whole Body Vibration.

## Figure 8.2 Exclude studies with exercise period less than 4 and more than 24 weeks (balance test batteries)

After excluding studies with exercise period less than 4 and more than 24 weeks, the hierarchy did not change significantly.

Figure 8.2: Forest plot of exercise period 4-24 weeks. Exercise type are ranked according to SMD compared to CON. Treatments crossing the y-axis are not significantly different from CON. *SMD* standardized Mean Difference, *CrI* Credible Interval, *AE* Aerobic Exercise, *AQE* Aquatic Exercise, *BGT* Balance and Gait Training, *BGT_ECA* Balance and Gait Training with External Cue or Attention, *BWS_TT* Body Weight Support Treadmill Training, *CON* Control group, *CPP* Classic Physiotherapy Program, *DT_BGT* Dual Task Balance and Gait Training, *Mul_C* Multicomponent Exercise Program, *Mul_D* Multidisciplinary Exercise Program, *RA_GT* Robotic Assisted Gait Training, *RT* Resistance Training, *TC* Tai Chi, *TT* Treadmill Training, *VR* Virtual Reality, *WBV* Whole Body Vibration.

## Figure 8.3 Exclude studies with exercise frequency less than 2 and more than 4 (balance test batteries)

After excluding studies with exercise frequency less than 2 and more than 4, the hierarchy did not change significantly.

Figure 8.3: Forest plot of exercise frequency 2-4. Treatments are ranked according to SMD compared to CON. Treatments crossing the y-axis are not significantly different from CON. *SMD* standardized Mean Difference, *CrI* Credible Interval, *AE* Aerobic Exercise, *AQE* Aquatic Exercise, *BGT* Balance and Gait Training, *BGT_ECA* Balance and Gait Training with External Cue or Attention, *BWS_TT* Body Weight Support Treadmill Training, *CON* Control group, *CPP* Classic Physiotherapy Program, *DT_BGT* Dual Task Balance and Gait Training, *Mul_C* Multicomponent Exercise Program, *Mul_D* Multidisciplinary Exercise Program, *NW* Nordic Walking, *PT* Power Training, *RA_GT* Robotic Assisted Gait Training, *RT* Resistance Training, *TC* Tai Chi, *TT* Treadmill Training, *VR* Virtual Reality, *WBV* Whole Body Vibration.

## Figure 8.4 Exclude studies that were OFF state during testing (balance test batteries)

After excluding studies that were OFF state during testing, the hierarchy did not change significantly.

Figure 8.4: Forest plot of studies that were ON state during the test. Treatments are ranked according to SMD compared to CON. Treatments crossing the y-axis are not significantly different from CON. *SMD* standardized Mean Difference, *CrI* Credible Interval, *AE* Aerobic Exercise, *AQE* Aquatic Exercise, *BGT* Balance and Gait Training, *BGT_ECA* Balance and Gait Training with External Cue or Attention, *BGT_ICA* Balance and Gait Training with Internal Cue or Attention, *BWS_TT* Body Weight Support Treadmill Training, *CON* Control group, *CPP* Classic Physiotherapy Program, *DT_BGT* Dual Task Balance and Gait Training, *Mul_C* Multicomponent Exercise Program, *Mul_D* Multidisciplinary Exercise Program, *NW* Nordic Walking, *PT* Power Training, *RA_GT* Robotic Assisted Gait Training, *RT* Resistance Training, *TC* Tai Chi, *TT* Treadmill Training, *VR* Virtual Reality, *WBV* Whole Body Vibration.

## Figure 8.5 Exclude studies that use GetData to extract data and estimated standard deviations value (balance test batteries)

After excluding studies that use GetData to extract data and estimated standard deviations value, the hierarchy did not change significantly.

Figure 8.5: Forest plot of original data and standard deviations. Treatments are ranked according to SMD compared to CON. Treatments crossing the y-axis are not significantly different from CON. *SMD* standardized Mean Difference, *CrI* Credible Interval, *AE* Aerobic Exercise, *AQE* Aquatic Exercise, *BGT* Balance and Gait Training, *BGT_ECA* Balance and Gait Training with External Cue or Attention, *BGT_ICA* Balance and Gait Training with Internal Cue or Attention, *BWS_TT* Body Weight Support Treadmill Training, *CON* Control group, *CPP* Classic Physiotherapy Program, *DT_BGT* Dual Task Balance and Gait Training, *Mul_C* Multicomponent Exercise Program, *Mul_D* Multidisciplinary Exercise Program, *NW* Nordic Walking, *PT* Power Training, *RA_GT* Robotic Assisted Gait Training, *RT* Resistance Training, *TC* Tai Chi, *TT* Treadmill Training, *VR* Virtual Reality, *WBV* Whole Body Vibration.

## Figure 8.6 Exclude studies at overall high risk of bias (static steady-state balance)

After excluding trials with high overall risk of bias, the hierarchy did not change significantly.

Figure 8.6: Forest plot of studies at moderate or low RoB. Exercise type are ranked according to SMD compared to CON. Treatments crossing the y-axis are not significantly different from CON. *SMD* standardized Mean Difference, *CrI* Credible Interval, *AE* Aerobic Exercise, *AQE* Aquatic Exercise, *BGT* Balance and Gait Training, *BGT_ECA* Balance and Gait Training with External Cue or Attention, *BWS_TT* Body Weight Support Treadmill Training, *CON* Control group, *CPP* Classic Physiotherapy Program, *DT_BGT* Dual Task Balance and Gait Training, *Mul_C* Multicomponent Exercise Program, *Mul_D* Multidisciplinary Exercise Program, *RA_GT* Robotic Assisted Gait Training, *RT* Resistance Training, *TC* Tai Chi, *TT* Treadmill Training, *VR* Virtual Reality, *WBV* Whole Body Vibration.

## Figure 8.7 Exclude studies with exercise period less than 4 and more than 24 weeks (Static steady-state balance)

After excluding studies with exercise period less than 4 and more than 24 weeks, the hierarchy did not change significantly.

Figure 8.7: Forest plot of exercise period 4-24 weeks. Exercise type are ranked according to SMD compared to CON. Treatments crossing the y-axis are not significantly different from CON. *SMD* standardized Mean Difference, *CrI* Credible Interval, *AE* Aerobic Exercise, *AQE* Aquatic Exercise, *BGT* Balance and Gait Training, *BGT_ECA* Balance and Gait Training with External Cue or Attention, *BWS_TT* Body Weight Support Treadmill Training, *CON* Control group, *CPP* Classic Physiotherapy Program, *DT_BGT* Dual Task Balance and Gait Training, *Mul_C* Multicomponent Exercise Program, *Mul_D* Multidisciplinary Exercise Program, *RA_GT* Robotic Assisted Gait Training, *RT* Resistance Training, *TC* Tai Chi, *TT* Treadmill Training, *VR* Virtual Reality, *WBV* Whole Body Vibration.

## Figure 8.8 Exclude studies with exercise frequency less than 2 and more than 4 (static steady-state balance)

After excluding studies with exercise frequency less than 2 and more than 4, the hierarchy did not change significantly.

Figure 8.8: Forest plot of exercise frequency 2-4. Treatments are ranked according to SMD compared to CON. Treatments crossing the y-axis are not significantly different from CON. *SMD* standardized Mean Difference, *CrI* Credible Interval, *AE* Aerobic Exercise, *AQE* Aquatic Exercise, *BGT* Balance and Gait Training, *BGT_ECA* Balance and Gait Training with External Cue or Attention, *BWS_TT* Body Weight Support Treadmill Training, *CON* Control group, *CPP* Classic Physiotherapy Program, *DT_BGT* Dual Task Balance and Gait Training, *Mul_C* Multicomponent Exercise Program, *Mul_D* Multidisciplinary Exercise Program, *NW* Nordic Walking, *PT* Power Training, *RA_GT* Robotic Assisted Gait Training, *RT* Resistance Training, *TC* Tai Chi, *TT* Treadmill Training, *VR* Virtual Reality, *WBV* Whole Body Vibration.

## Figure 8.9 Exclude studies that were OFF state during testing (static steady-state balance)

After excluding studies that were OFF state during testing, the hierarchy did not change significantly.

Figure 8.9: Forest plot of studies that were ON state during the test. Treatments are ranked according to SMD compared to CON. Treatments crossing the y-axis are not significantly different from CON. *SMD* standardized Mean Difference, *CrI* Credible Interval, *AE* Aerobic Exercise, *AQE* Aquatic Exercise, *BGT* Balance and Gait Training, *BGT_ECA* Balance and Gait Training with External Cue or Attention, *BGT_ICA* Balance and Gait Training with Internal Cue or Attention, *BWS_TT* Body Weight Support Treadmill Training, *CON* Control group, *CPP* Classic Physiotherapy Program, *DT_BGT* Dual Task Balance and Gait Training, *Mul_C* Multicomponent Exercise Program, *Mul_D* Multidisciplinary Exercise Program, *NW* Nordic Walking, *PT* Power Training, *RA_GT* Robotic Assisted Gait Training, *RT* Resistance Training, *TC* Tai Chi, *TT* Treadmill Training, *VR* Virtual Reality, *WBV* Whole Body Vibration.

## Figure 8.10 Exclude studies that use GetData to extract data and estimated standard deviations value (Static steady-state balance)

After excluding studies that use GetData to extract data and estimated standard deviations value, the hierarchy did not change significantly.

Figure 8.10: Forest plot of original data and standard deviations. Treatments are ranked according to SMD compared to CON. Treatments crossing the y-axis are not significantly different from CON. *SMD* standardized Mean Difference, *CrI* Credible Interval, *AE* Aerobic Exercise, *AQE* Aquatic Exercise, *BGT* Balance and Gait Training, *BGT_ECA* Balance and Gait Training with External Cue or Attention, *BGT_ICA* Balance and Gait Training with Internal Cue or Attention, *BWS_TT* Body Weight Support Treadmill Training, *CON* Control group, *CPP* Classic Physiotherapy Program, *DT_BGT* Dual Task Balance and Gait Training, *Mul_C* Multicomponent Exercise Program, *Mul_D* Multidisciplinary Exercise Program, *NW* Nordic Walking, *PT* Power Training, *RA_GT* Robotic Assisted Gait Training, *RT* Resistance Training, *TC* Tai Chi, *TT* Treadmill Training, *VR* Virtual Reality, *WBV* Whole Body Vibration.

## Figure 8.11 Exclude studies at overall high risk of bias (dynamic steady-state balance)

After excluding trials with high overall risk of bias, the hierarchy did not change significantly.

Figure 8.11: Forest plot of studies at moderate or low RoB. Exercise type are ranked according to SMD compared to CON. Treatments crossing the y-axis are not significantly different from CON. *SMD* standardized Mean Difference, *CrI* Credible Interval, *AE* Aerobic Exercise, *AQE* Aquatic Exercise, *BGT* Balance and Gait Training, *BGT_ECA* Balance and Gait Training with External Cue or Attention, *BWS_TT* Body Weight Support Treadmill Training, *CON* Control group, *CPP* Classic Physiotherapy Program, *DT_BGT* Dual Task Balance and Gait Training, *Mul_C* Multicomponent Exercise Program, *Mul_D* Multidisciplinary Exercise Program, *RA_GT* Robotic Assisted Gait Training, *RT* Resistance Training, *TC* Tai Chi, *TT* Treadmill Training, *VR* Virtual Reality, *WBV* Whole Body Vibration.

## Figure 8.12 Exclude studies with exercise period less than 4 and more than 24 weeks (dynamic steady-state balance)

After excluding studies with exercise period less than 4 and more than 24 weeks, the hierarchy did not change significantly.

Figure 8.12: Forest plot of exercise period 4-24 weeks. Exercise type are ranked according to SMD compared to CON. Treatments crossing the y-axis are not significantly different from CON. *SMD* standardized Mean Difference, *CrI* Credible Interval, *AE* Aerobic Exercise, *AQE* Aquatic Exercise, *BGT* Balance and Gait Training, *BGT_ECA* Balance and Gait Training with External Cue or Attention, *BWS_TT* Body Weight Support Treadmill Training, *CON* Control group, *CPP* Classic Physiotherapy Program, *DT_BGT* Dual Task Balance and Gait Training, *Mul_C* Multicomponent Exercise Program, *Mul_D* Multidisciplinary Exercise Program, *RA_GT* Robotic Assisted Gait Training, *RT* Resistance Training, *TC* Tai Chi, *TT* Treadmill Training, *VR* Virtual Reality, *WBV* Whole Body Vibration.

## Figure 8.13 Exclude studies with exercise frequency less than 2 and more than 4 (dynamic steady-state balance)

After excluding studies with exercise frequency less than 2 and more than 4, the hierarchy did not change significantly.

Figure 8.13: Forest plot of exercise frequency 2-4. Treatments are ranked according to SMD compared to CON. Treatments crossing the y-axis are not significantly different from CON. *SMD* standardized Mean Difference, *CrI* Credible Interval, *AE* Aerobic Exercise, *AQE* Aquatic Exercise, *BGT* Balance and Gait Training, *BGT_ECA* Balance and Gait Training with External Cue or Attention, *BWS_TT* Body Weight Support Treadmill Training, *CON* Control group, *CPP* Classic Physiotherapy Program, *DT_BGT* Dual Task Balance and Gait Training, *Mul_C* Multicomponent Exercise Program, *Mul_D* Multidisciplinary Exercise Program, *NW* Nordic Walking, *PT* Power Training, *RA_GT* Robotic Assisted Gait Training, *RT* Resistance Training, *TC* Tai Chi, *TT* Treadmill Training, *VR* Virtual Reality, *WBV* Whole Body Vibration.

## Figure 8.14 Exclude studies that were OFF state during testing (dynamic steady-state balance)

After excluding studies that were OFF state during testing, the hierarchy did not change significantly.

Figure 8.14: Forest plot of studies that were ON state during the test. Treatments are ranked according to SMD compared to CON. Treatments crossing the y-axis are not significantly different from CON. *SMD* standardized Mean Difference, *CrI* Credible Interval, *AE* Aerobic Exercise, *AQE* Aquatic Exercise, *BGT* Balance and Gait Training, *BGT_ECA* Balance and Gait Training with External Cue or Attention, *BGT_ICA* Balance and Gait Training with Internal Cue or Attention, *BWS_TT* Body Weight Support Treadmill Training, *CON* Control group, *CPP* Classic Physiotherapy Program, *DT_BGT* Dual Task Balance and Gait Training, *Mul_C* Multicomponent Exercise Program, *Mul_D* Multidisciplinary Exercise Program, *NW* Nordic Walking, *PT* Power Training, *RA_GT* Robotic Assisted Gait Training, *RT* Resistance Training, *TC* Tai Chi, *TT* Treadmill Training, *VR* Virtual Reality, *WBV* Whole Body Vibration.

## Figure 8.15 Exclude studies that use GetData to extract data and estimated standard deviations value (dynamic steady-state balance)

After excluding studies that use GetData to extract data and estimated standard deviations value, the hierarchy did not change significantly.

Figure 8.15: Forest plot of original data and standard deviations. Treatments are ranked according to SMD compared to CON. Treatments crossing the y-axis are not significantly different from CON. *SMD* standardized Mean Difference, *CrI* Credible Interval, *AE* Aerobic Exercise, *AQE* Aquatic Exercise, *BGT* Balance and Gait Training, *BGT_ECA* Balance and Gait Training with External Cue or Attention, *BGT_ICA* Balance and Gait Training with Internal Cue or Attention, *BWS_TT* Body Weight Support Treadmill Training, *CON* Control group, *CPP* Classic Physiotherapy Program, *DT_BGT* Dual Task Balance and Gait Training, *Mul_C* Multicomponent Exercise Program, *Mul_D* Multidisciplinary Exercise Program, *NW* Nordic Walking, *PT* Power Training, *RA_GT* Robotic Assisted Gait Training, *RT* Resistance Training, *TC* Tai Chi, *TT* Treadmill Training, *VR* Virtual Reality, *WBV* Whole Body Vibration.

## Figure 8.16 Exclude studies at overall high risk of bias (proactive balance)

After excluding trials with high overall risk of bias, the hierarchy did not change significantly.

Figure 8.16: Forest plot of studies at moderate or low RoB. Exercise type are ranked according to SMD compared to CON. Treatments crossing the y-axis are not significantly different from CON. *SMD* standardized Mean Difference, *CrI* Credible Interval, *AE* Aerobic Exercise, *AQE* Aquatic Exercise, *BGT* Balance and Gait Training, *BGT_ECA* Balance and Gait Training with External Cue or Attention, *BWS_TT* Body Weight Support Treadmill Training, *CON* Control group, *CPP* Classic Physiotherapy Program, *DT_BGT* Dual Task Balance and Gait Training, *Mul_C* Multicomponent Exercise Program, *Mul_D* Multidisciplinary Exercise Program, *RA_GT* Robotic Assisted Gait Training, *RT* Resistance Training, *TC* Tai Chi, *TT* Treadmill Training, *VR* Virtual Reality, *WBV* Whole Body Vibration.

## Figure 8.17 Exclude studies with exercise period less than 4 and more than 24 weeks (proactive balance)

After excluding studies with exercise period less than 4 and more than 24 weeks, the hierarchy did not change significantly.

Figure 8.17: Forest plot of exercise period 4-24 weeks. Exercise type are ranked according to SMD compared to CON. Treatments crossing the y-axis are not significantly different from CON. *SMD* standardized Mean Difference, *CrI* Credible Interval, *AE* Aerobic Exercise, *AQE* Aquatic Exercise, *BGT* Balance and Gait Training, *BGT_ECA* Balance and Gait Training with External Cue or Attention, *BWS_TT* Body Weight Support Treadmill Training, *CON* Control group, *CPP* Classic Physiotherapy Program, *DT_BGT* Dual Task Balance and Gait Training, *Mul_C* Multicomponent Exercise Program, *Mul_D* Multidisciplinary Exercise Program, *RA_GT* Robotic Assisted Gait Training, *RT* Resistance Training, *TC* Tai Chi, *TT* Treadmill Training, *VR* Virtual Reality, *WBV* Whole Body Vibration.

## Figure 8.18 Exclude studies with exercise frequency less than 2 and more than 4 (proactive balance)

After excluding studies with exercise frequency less than 2 and more than 4, the hierarchy did not change significantly.

Figure 8.18: Forest plot of exercise frequency 2-4. Treatments are ranked according to SMD compared to CON. Treatments crossing the y-axis are not significantly different from CON. *SMD* standardized Mean Difference, *CrI* Credible Interval, *AE* Aerobic Exercise, *AQE* Aquatic Exercise, *BGT* Balance and Gait Training, *BGT_ECA* Balance and Gait Training with External Cue or Attention, *BWS_TT* Body Weight Support Treadmill Training, *CON* Control group, *CPP* Classic Physiotherapy Program, *DT_BGT* Dual Task Balance and Gait Training, *Mul_C* Multicomponent Exercise Program, *Mul_D* Multidisciplinary Exercise Program, *NW* Nordic Walking, *PT* Power Training, *RA_GT* Robotic Assisted Gait Training, *RT* Resistance Training, *TC* Tai Chi, *TT* Treadmill Training, *VR* Virtual Reality, *WBV* Whole Body Vibration.

## Figure 8.19 Exclude studies that were OFF state during testing (proactive balance)

After excluding studies that were OFF state during testing, the hierarchy did not change significantly.

Figure 8.19: Forest plot of studies that were ON state during the test. Treatments are ranked according to SMD compared to CON. Treatments crossing the y-axis are not significantly different from CON. *SMD* standardized Mean Difference, *CrI* Credible Interval, *AE* Aerobic Exercise, *AQE* Aquatic Exercise, *BGT* Balance and Gait Training, *BGT_ECA* Balance and Gait Training with External Cue or Attention, *BGT_ICA* Balance and Gait Training with Internal Cue or Attention, *BWS_TT* Body Weight Support Treadmill Training, *CON* Control group, *CPP* Classic Physiotherapy Program, *DT_BGT* Dual Task Balance and Gait Training, *Mul_C* Multicomponent Exercise Program, *Mul_D* Multidisciplinary Exercise Program, *NW* Nordic Walking, *PT* Power Training, *RA_GT* Robotic Assisted Gait Training, *RT* Resistance Training, *TC* Tai Chi, *TT* Treadmill Training, *VR* Virtual Reality, *WBV* Whole Body Vibration.

## Figure 8.20 Exclude studies that use GetData to extract data and estimated standard deviations value (proactive balance)

After excluding studies that use GetData to extract data and estimated standard deviations value, the hierarchy did not change significantly.

Figure 8.20: Forest plot of original data and standard deviations. Treatments are ranked according to SMD compared to CON. Treatments crossing the y-axis are not significantly different from CON. *SMD* standardized Mean Difference, *CrI* Credible Interval, *AE* Aerobic Exercise, *AQE* Aquatic Exercise, *BGT* Balance and Gait Training, *BGT_ECA* Balance and Gait Training with External Cue or Attention, *BGT_ICA* Balance and Gait Training with Internal Cue or Attention, *BWS_TT* Body Weight Support Treadmill Training, *CON* Control group, *CPP* Classic Physiotherapy Program, *DT_BGT* Dual Task Balance and Gait Training, *Mul_C* Multicomponent Exercise Program, *Mul_D* Multidisciplinary Exercise Program, *NW* Nordic Walking, *PT* Power Training, *RA_GT* Robotic Assisted Gait Training, *RT* Resistance Training, *TC* Tai Chi, *TT* Treadmill Training, *VR* Virtual Reality, *WBV* Whole Body Vibration.

## Figure 8.21 Exclude studies with exercise period less than 4 and more than 24 weeks (reactive balance)

After excluding studies with exercise period less than 4 and more than 24 weeks, the hierarchy did not change significantly.

Figure 8.22: Forest plot of exercise period 4-24 weeks. Exercise type are ranked according to SMD compared to CON. Treatments crossing the y-axis are not significantly different from CON. *SMD* standardized Mean Difference, *CrI* Credible Interval, *AE* Aerobic Exercise, *AQE* Aquatic Exercise, *BGT* Balance and Gait Training, *BGT_ECA* Balance and Gait Training with External Cue or Attention, *BWS_TT* Body Weight Support Treadmill Training, *CON* Control group, *CPP* Classic Physiotherapy Program, *DT_BGT* Dual Task Balance and Gait Training, *Mul_C* Multicomponent Exercise Program, *Mul_D* Multidisciplinary Exercise Program, *RA_GT* Robotic Assisted Gait Training, *RT* Resistance Training, *TC* Tai Chi, *TT* Treadmill Training, *VR* Virtual Reality, *WBV* Whole Body Vibration.

## Figure 8.23 Exclude studies with exercise frequency less than 2 and more than 4 (reactive balance)

After excluding studies with exercise frequency less than 2 and more than 4, the hierarchy did not change significantly.

Figure 8.23: Forest plot of exercise frequency 2-4. Treatments are ranked according to SMD compared to CON. Treatments crossing the y-axis are not significantly different from CON. *SMD* standardized Mean Difference, *CrI* Credible Interval, *AE* Aerobic Exercise, *AQE* Aquatic Exercise, *BGT* Balance and Gait Training, *BGT_ECA* Balance and Gait Training with External Cue or Attention, *BWS_TT* Body Weight Support Treadmill Training, *CON* Control group, *CPP* Classic Physiotherapy Program, *DT_BGT* Dual Task Balance and Gait Training, *Mul_C* Multicomponent Exercise Program, *Mul_D* Multidisciplinary Exercise Program, *NW* Nordic Walking, *PT* Power Training, *RA_GT* Robotic Assisted Gait Training, *RT* Resistance Training, *TC* Tai Chi, *TT* Treadmill Training, *VR* Virtual Reality, *WBV* Whole Body Vibration.

# Appendix 9: Grading the evidence for primary outcome of the network meta-analysis using CINeMA

## 9.1.1 Overall balance ability

The figure below showed that TC, BGT, Yoga, DT_BGT, Dance had high risk of bias (RoB) studies. In addition, there were comparison of high RoB for TC, BGT, and Yoga.


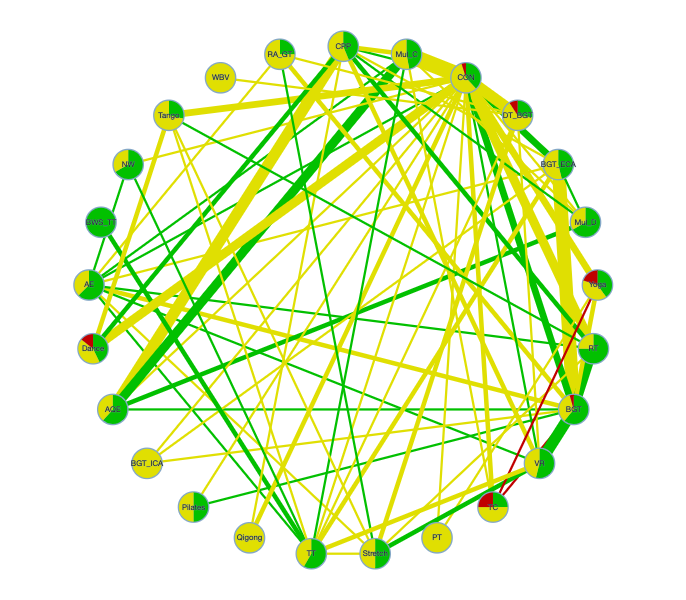


Figure 9.1.1 Network plot of study limitations of the included studies for overall balance ability. Node size by equal size, node color by RoB. The colors in the circles indicate the percentage of low RoB studies (green), moderate RoB studies (yellow), high RoB studies (red) about each exercise type. Edge width by sample size. Edge color by average RoB. The colors of the lines indicate the summative RoB assessment of each comparison. Low RoB is green, moderate RoB is yellow, high RoB is red. *AE* Aerobic Exercise, *AQE* Aquatic Exercise, *BGT* Balance and Gait Training, *BGT_ECA* Balance and Gait Training with External Cue or Attention, *BGT_ICA* Balance and Gait Training with Internal Cue or Attention, *BWS_TT* Body Weight Support Treadmill Training, *CON* Control group, *CPP* Classic Physiotherapy Program, *DT_BGT* Dual Task Balance and Gait Training, *Mul_C* Multicomponent Exercise Program, *Mul_D* Multidisciplinary Exercise Program, *NW* Nordic Walking, *PT* Power Training, *RA_GT* Robotic Assisted Gait Training, *RT* Resistance Training, *TC* Tai Chi, *TT* Treadmill Training, *VR* Virtual Reality, *WBV* Whole Body Vibration.

## 9.1.2 Static steady-state balance

The figure below showed that BGT_ECA, TT had high risk of bias (RoB) studies. In addition, there was no comparison of high RoB for each type of exercise


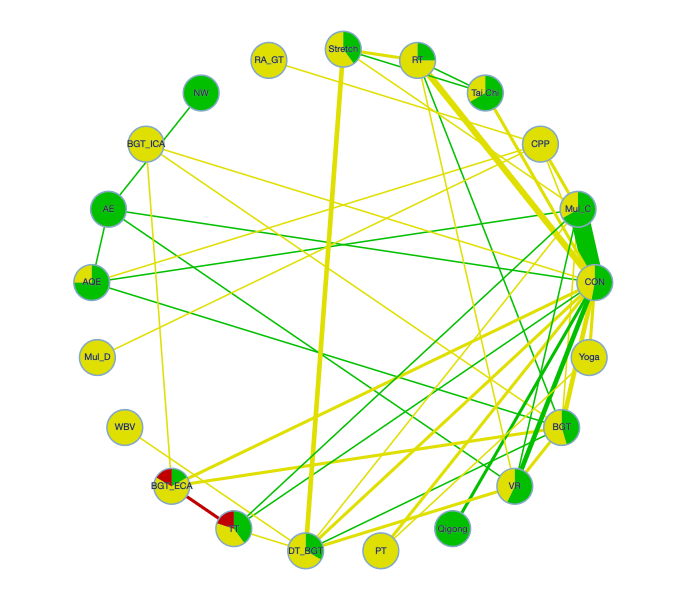


Figure 9.1.2 Network plot of study limitations of the included studies for Static steady-state balance. Node size by equal size, node color by RoB. The colors in the circles indicate the percentage of low RoB studies (green), moderate RoB studies (yellow), high RoB studies (red) about each exercise type. Edge width by sample size. Edge color by average RoB. The colors of the lines indicate the summative RoB assessment of each comparison. Low RoB is green, moderate RoB is yellow, high RoB is red. *AE* Aerobic Exercise, *AQE* Aquatic Exercise, *BGT* Balance and Gait Training, *BGT_ECA* Balance and Gait Training with External Cue or Attention, *BGT_ICA* Balance and Gait Training with Internal Cue or Attention, *BWS_TT* Body Weight Support Treadmill Training, *CON* Control group, *CPP* Classic Physiotherapy Program, *DT_BGT* Dual Task Balance and Gait Training, *Mul_C* Multicomponent Exercise Program, *Mul_D* Multidisciplinary Exercise Program, *NW* Nordic Walking, *PT* Power Training, *RA_GT* Robotic Assisted Gait Training, *RT* Resistance Training, *TC* Tai Chi, *TT* Treadmill Training, *VR* Virtual Reality, *WBV* Whole Body Vibration.

## 9.1.3 Dynamic steady-state balance

The figure below showed that TC, BWS_TT, CPP, TT, BGT, BGT_ECA, Yoga, DT_BGT, Dance had high risk of bias (RoB) studies. In addition, there were comparison of high RoB for TC, Dance, BWS_TT, CPP, TT, DT_BGT, BGT, and Yoga.


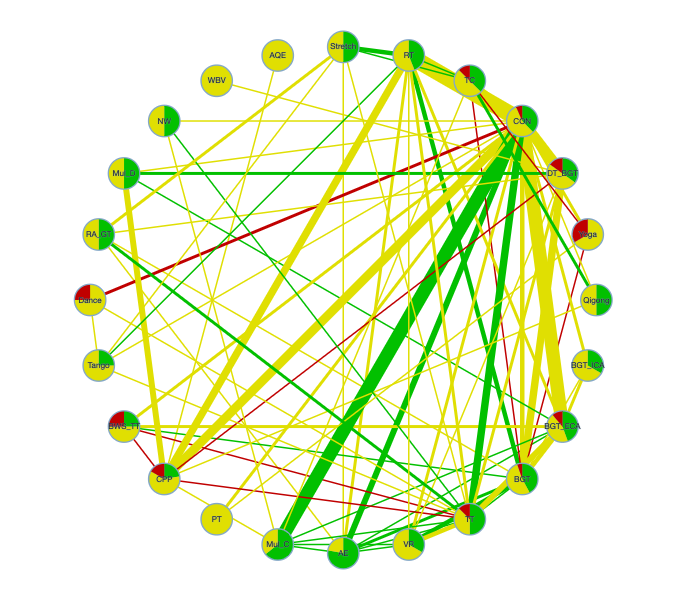


Figure 9.1.3 Network plot of study limitations of the included studies for dynamic steady-state balance. Node size by equal size, node color by RoB. The colors in the circles indicate the percentage of low RoB studies (green), moderate RoB studies (yellow), high RoB studies (red) about each exercise type. Edge width by sample size. Edge color by average RoB. The colors of the lines indicate the summative RoB assessment of each comparison. Low RoB is green, moderate RoB is yellow, high RoB is red. *AE* Aerobic Exercise, *AQE* Aquatic Exercise, *BGT* Balance and Gait Training, *BGT_ECA* Balance and Gait Training with External Cue or Attention, *BGT_ICA* Balance and Gait Training with Internal Cue or Attention, *BWS_TT* Body Weight Support Treadmill Training, *CON* Control group, *CPP* Classic Physiotherapy Program, *DT_BGT* Dual Task Balance and Gait Training, *Mul_C* Multicomponent Exercise Program, *Mul_D* Multidisciplinary Exercise Program, *NW* Nordic Walking, *PT* Power Training, *RA_GT* Robotic Assisted Gait Training, *RT* Resistance Training, *TC* Tai Chi, *TT* Treadmill Training, *VR* Virtual Reality, *WBV* Whole Body Vibration.

## 9.1.4 Proactive balance

The figure below showed that Dance, RT, Tai Chi, CPP, Yoga, BGT, TT, and BGT_ECA had high risk of bias (RoB) studies. In addition, there were comparison of high RoB for Tai Chi, BGT, and Yoga.


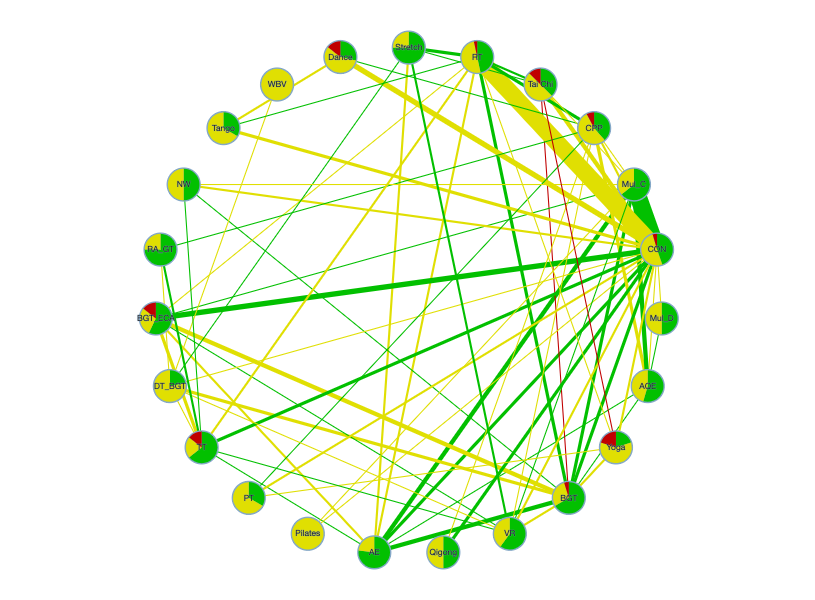


Figure 9.1.4 Network plot of study limitations of the included studies for proactive balance. Node size by equal size, node color by RoB. The colors in the circles indicate the percentage of low RoB studies (green), moderate RoB studies (yellow), high RoB studies (red) about each exercise type. Edge width by sample size. Edge color by average RoB. The colors of the lines indicate the summative RoB assessment of each comparison. Low RoB is green, moderate RoB is yellow, high RoB is red. *AE* Aerobic Exercise, *AQE* Aquatic Exercise, *BGT* Balance and Gait Training, *BGT_ECA* Balance and Gait Training with External Cue or Attention, *BGT_ICA* Balance and Gait Training with Internal Cue or Attention, *BWS_TT* Body Weight Support Treadmill Training, *CON* Control group, *CPP* Classic Physiotherapy Program, *DT_BGT* Dual Task Balance and Gait Training, *Mul_C* Multicomponent Exercise Program, *Mul_D* Multidisciplinary Exercise Program, *NW* Nordic Walking, *PT* Power Training, *RA_GT* Robotic Assisted Gait Training, *RT* Resistance Training, *TC* Tai Chi, *TT* Treadmill Training, *VR* Virtual Reality, *WBV* Whole Body Vibration.

## 9.1.5 Reactive balance

The figure below showed that have no high risk of bias (RoB) studies. In addition, there was no comparison of high RoB for each type of exercise.


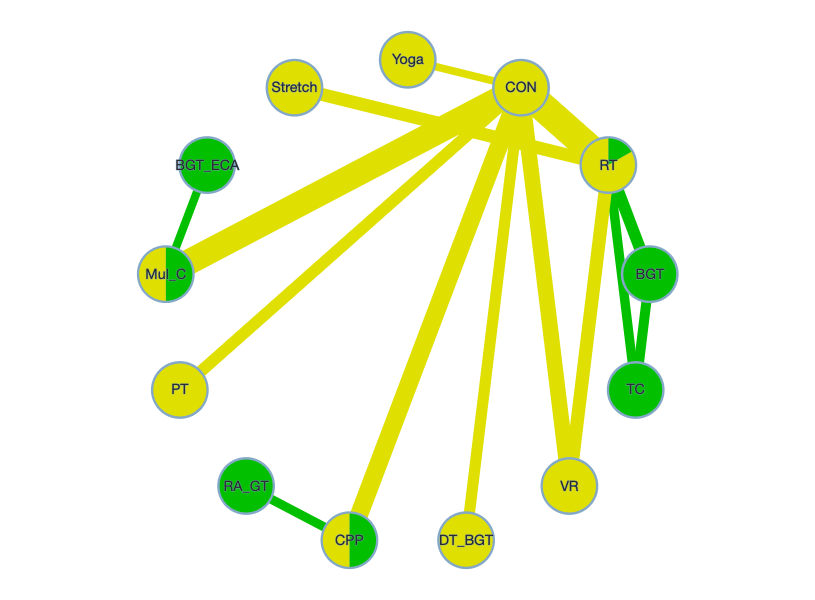


Figure 9.1.5 Network plot of study limitations of the included studies for reactive balance. Node size by equal size, node color by RoB. The colors in the circles indicate the percentage of low RoB studies (green), moderate RoB studies (yellow), high RoB studies (red) about each exercise type. Edge width by sample size. Edge color by average RoB. The colors of the lines indicate the summative RoB assessment of each comparison. Low RoB is green, moderate RoB is yellow, high RoB is red. *AE* Aerobic Exercise, *AQE* Aquatic Exercise, *BGT* Balance and Gait Training, *BGT_ECA* Balance and Gait Training with External Cue or Attention, *BGT_ICA* Balance and Gait Training with Internal Cue or Attention, *BWS_TT* Body Weight Support Treadmill Training, *CON* Control group, *CPP* Classic Physiotherapy Program, *DT_BGT* Dual Task Balance and Gait Training, *Mul_C* Multicomponent Exercise Program, *Mul_D* Multidisciplinary Exercise Program, *NW* Nordic Walking, *PT* Power Training, *RA_GT* Robotic Assisted Gait Training, *RT* Resistance Training, *TC* Tai Chi, *TT* Treadmill Training, *VR* Virtual Reality, *WBV* Whole Body Vibration.

## 9.2 Reasons for downgrading

Based on the recommendations of the CINeMA online document (https://cinema.ispm.unibe.ch/), we graded the results of balance outcomes, and judged whether each module needs to be downgraded according to the following criteria.

***With-study bias***

We classified the quality evaluation results (appendix 7) of each included study into low-risk, moderate-risk, and high-risk according to the standards recommended by Huhn, Nikolakopoulou ^1^. We selected the rule is average RoB. No need to downgrade when the result was “no concerns”, downgrade one level when “some concerns” and downgrade two level “major concerns”.

***Across-study bias (publication bias)***

Our search was relatively comprehensive, including published and unpublished studies. Even if it is possible that we missed other small unpublished experiments, then it does not seem to affect our results. We evaluated the primary outcome of publication bias, and comparison-adjusted funnel plots show no evidence of asymmetry, and the results of Egger’s test showed that no small study effect was found. Therefore, the outcomes were deemed to have no publication bias.

***Indirectness***

Meta-regression was performed on the primary outcome with the baseline information (published years, sample size, average age, disease grade, diagnosis years, and the ratio of male to female subjects) of the included studies as covariates, and the results showed that the baseline information did not significantly affect the results . Therefore no indirectness was assumed and no comparison was downgraded for this reason.

***Imprecision***

The outcomes of this network meta-analysis is a continuous variable, and the effect size measure for continuous outcomes chooses the standardized mean difference (SMD) of the change score (end-point minus baseline score) because the studies use different rating scales or units. Therefore, for CON comparisons the clinically meaningful threshold was set at a standardized mean difference of higher or lower than 0, and for the comparisons of the two types of exercise, the threshold was set at SMD -0.1 and 0.1. If the confidence interval crossed one threshold, it will be downgraded by one level, and two thresholds will be downgraded by two levels.

***Heterogeneity***

For heterogeneity, we used the same threshold as the above clinically significant threshold and follow the recommendations automatically provided by CINeMA (https://cinema.ispm.unibe.ch/). No need to downgrade when the result was “no concerns”, downgrade one level when “some concerns” and downgrade two level when “major concerns”.

***Incoherence***

For incoherence, we will use global and local methods to test the inconsistency of the research results. For global inconsistency, we evaluated inconsistency statistically using the design-by-treatment test. In addition, we will assessment of local inconsistency by separating indirect from direct evidence (SIDE test) using the R netmeta package (appendix 12). No need to downgrade when p >0.1, downgrade one level when p was 0.05-0.1 and downgrade two level when p <0.05 .

***Summarising judgments across the 6 domains***

Τhe final output of CINeMA is a table with the level of concern for each of the 6 domains. we choose to summarise judgments across domains using the 4 levels of confidence of the GRADE approach: very low, low, moderate, or high.^2^ Due to factors that may reduce the confidence in a treatment effect may affect more than 1 domain. Indirectness includes consideration of intransitivity, which is manifested as statistical incoherence in the data. Heterogeneity will increase the imprecision of treatment effect, and may be related to the variability of bias within the study or the existence of reporting bias. In addition, in the presence of heterogeneity, the ability to detect important discontinuities will be reduced.^3^ Therefore, the 6 CINeMA domains should be considered jointly rather than in isolation to avoid downgrading the overall level of confidence more than once for related concerns. For example, the comparison between BGT_ECA and CON showed ‘major confers’ in both heterogeneity and incoherence (appendix 11.3 pp 204), we only downgraded two levels, because imprecision, heterogeneity, and incoherence are interconnected.^4^

**Reference**

1. Huhn M, Nikolakopoulou A, Schneider-Thoma J, et al. Comparative efficacy and tolerability of 32 oral antipsychotics for the acute treatment of adults with multi-episode schizophrenia: a systematic review and network meta-analysis. *Lancet* 2019; **394**(10202): 939-51.

2. Puhan MA, Schünemann HJ, Murad MH, et al. A GRADE Working Group approach for rating the quality of treatment effect estimates from network meta-analysis. *BMJ* 2014; **349**.

3. Veroniki AA, Mavridis D, Higgins JPT, Salanti G. Characteristics of a loop of evidence that affect detection and estimation of inconsistency: a simulation study. *BMC Med Res Methodol* 2014; **14**: 106.

4. Nikolakopoulou A, Higgins JPT, Papakonstantinou T, et al. CINeMA: An approach for assessing confidence in the results of a network meta-analysis. *PLoS Med* 2020; **17**(4): e1003082.

## 9.3.1 CINeMA for overall balance ability

| **Comparison** | **Number of studies** | **Within-study bias** | **Reporting bias** | **Indirectness** | **Imprecision** | **Heterogeneity** | **Incoherence** | **Confidence rating** |
| --- | --- | --- | --- | --- | --- | --- | --- | --- |
| AE:AQE | 0 | No concerns | Low risk | No concerns | Major concerns | No concerns | No concerns | Low |
| AE:BGT | 2 | Some concerns | Low risk | No concerns | Major concerns | No concerns | No concerns | Very low |
| AE:BGT_ECA | 1 | Some concerns | Low risk | No concerns | Major concerns | No concerns | No concerns | Very low |
| AE:BGT_ICA | 0 | Some concerns | Low risk | No concerns | Major concerns | No concerns | No concerns | Very low |
| AE:BWS_TT | 0 | No concerns | Low risk | No concerns | No concerns | No concerns | No concerns | High |
| AE:CON | 1 | No concerns | Low risk | No concerns | No concerns | Major concerns | No concerns | Low |
| AE:CPP | 0 | No concerns | Low risk | No concerns | No concerns | Major concerns | No concerns | Low |
| AE:Dance | 0 | Some concerns | Low risk | No concerns | Major concerns | No concerns | No concerns | Very low |
| AE:DT_BGT | 0 | Some concerns | Low risk | No concerns | Major concerns | No concerns | No concerns | Very low |
| AE:Mul_C | 1 | No concerns | Low risk | No concerns | Major concerns | No concerns | No concerns | Low |
| AE:Mul_D | 0 | No concerns | Low risk | No concerns | Major concerns | No concerns | No concerns | Low |
| AE:NW | 1 | No concerns | Low risk | No concerns | Major concerns | No concerns | No concerns | Low |
| AE:Pilates | 0 | No concerns | Low risk | No concerns | Major concerns | No concerns | No concerns | Low |
| AE:PT | 0 | Some concerns | Low risk | No concerns | Major concerns | No concerns | No concerns | Very low |
| AE:Qigong | 0 | Some concerns | Low risk | No concerns | Major concerns | No concerns | No concerns | Very low |
| AE:RA_GT | 1 | Some concerns | Low risk | No concerns | Major concerns | No concerns | No concerns | Very low |
| AE:RT | 1 | No concerns | Low risk | No concerns | Major concerns | No concerns | No concerns | Low |
| AE:Stretch | 1 | Some concerns | Low risk | No concerns | No concerns | Major concerns | Major concerns | Very low |
| AE:Tango | 0 | Some concerns | Low risk | No concerns | Major concerns | No concerns | No concerns | Very low |
| AE:TC | 0 | Some concerns | Low risk | No concerns | Major concerns | No concerns | No concerns | Very low |
| AE:TT | 1 | No concerns | Low risk | No concerns | Major concerns | No concerns | No concerns | Low |
| AE:VR | 1 | No concerns | Low risk | No concerns | Major concerns | No concerns | No concerns | Low |
| AE:WBV | 0 | Some concerns | Low risk | No concerns | Major concerns | No concerns | No concerns | Very low |
| AE:Yoga | 0 | Some concerns | Low risk | No concerns | Major concerns | No concerns | No concerns | Very low |
| AQE:BGT | 1 | No concerns | Low risk | No concerns | No concerns | Major concerns | No concerns | Low |
| AQE:BGT_ECA | 0 | No concerns | Low risk | No concerns | Major concerns | No concerns | No concerns | Low |
| AQE:BGT_ICA | 0 | Some concerns | Low risk | No concerns | Major concerns | No concerns | No concerns | Very low |
| AQE:BWS_TT | 0 | No concerns | Low risk | No concerns | No concerns | Major concerns | No concerns | Low |
| AQE:CON | 1 | No concerns | Low risk | No concerns | No concerns | No concerns | Some concerns | Moderate |
| AQE:CPP | 5 | Some concerns | Low risk | No concerns | No concerns | No concerns | No concerns | Moderate |
| AQE:Dance | 0 | No concerns | Low risk | No concerns | Major concerns | No concerns | No concerns | Low |
| AQE:DT_BGT | 0 | Some concerns | Low risk | No concerns | Major concerns | No concerns | No concerns | Very low |
| AQE:Mul_C | 4 | No concerns | Low risk | No concerns | Major concerns | No concerns | No concerns | Low |
| AQE:Mul_D | 2 | No concerns | Low risk | No concerns | Major concerns | No concerns | No concerns | Low |
| AQE:NW | 0 | No concerns | Low risk | No concerns | Major concerns | No concerns | No concerns | Low |
| AQE:Pilates | 0 | No concerns | Low risk | No concerns | Major concerns | No concerns | No concerns | Low |
| AQE:PT | 0 | Some concerns | Low risk | No concerns | Major concerns | No concerns | No concerns | Very low |
| AQE:Qigong | 0 | Some concerns | Low risk | No concerns | Major concerns | No concerns | No concerns | Very low |
| AQE:RA_GT | 0 | Some concerns | Low risk | No concerns | Major concerns | No concerns | No concerns | Very low |
| AQE:RT | 0 | No concerns | Low risk | No concerns | No concerns | Major concerns | No concerns | Low |
| AQE:Stretch | 0 | Some concerns | Low risk | No concerns | No concerns | No concerns | No concerns | Moderate |
| AQE:Tango | 0 | Some concerns | Low risk | No concerns | Major concerns | No concerns | No concerns | Very low |
| AQE:TC | 0 | Some concerns | Low risk | No concerns | Major concerns | No concerns | No concerns | Very low |
| AQE:TT | 0 | No concerns | Low risk | No concerns | Major concerns | No concerns | No concerns | Low |
| AQE:VR | 0 | No concerns | Low risk | No concerns | Major concerns | No concerns | No concerns | Low |
| AQE:WBV | 0 | Some concerns | Low risk | No concerns | Major concerns | No concerns | No concerns | Very low |
| AQE:Yoga | 0 | Some concerns | Low risk | No concerns | Major concerns | No concerns | No concerns | Very low |
| BGT:BGT_ECA | 6 | No concerns | Low risk | No concerns | No concerns | Major concerns | No concerns | Low |
| BGT:BGT_ICA | 1 | Some concerns | Low risk | No concerns | Major concerns | No concerns | No concerns | Very low |
| BGT:BWS_TT | 0 | No concerns | Low risk | No concerns | No concerns | No concerns | No concerns | High |
| BGT:CON | 3 | No concerns | Low risk | No concerns | No concerns | Major concerns | No concerns | Low |
| BGT:CPP | 0 | No concerns | Low risk | No concerns | Major concerns | No concerns | No concerns | Low |
| BGT:Dance | 0 | Some concerns | Low risk | No concerns | No concerns | Major concerns | No concerns | Very low |
| BGT:DT_BGT | 0 | Some concerns | Low risk | No concerns | No concerns | Major concerns | No concerns | Very low |
| BGT:Mul_C | 0 | No concerns | Low risk | No concerns | Major concerns | No concerns | No concerns | Low |
| BGT:Mul_D | 0 | No concerns | Low risk | No concerns | No concerns | Major concerns | No concerns | Low |
| BGT:NW | 0 | No concerns | Low risk | No concerns | Major concerns | No concerns | No concerns | Low |
| BGT:Pilates | 1 | No concerns | Low risk | No concerns | No concerns | Major concerns | No concerns | Low |
| BGT:PT | 0 | Some concerns | Low risk | No concerns | Major concerns | No concerns | No concerns | Very low |
| BGT:Qigong | 0 | Some concerns | Low risk | No concerns | Major concerns | No concerns | No concerns | Very low |
| BGT:RA_GT | 2 | Some concerns | Low risk | No concerns | No concerns | Major concerns | No concerns | Very low |
| BGT:RT | 4 | No concerns | Low risk | No concerns | Major concerns | No concerns | No concerns | Low |
| BGT:Stretch | 0 | Some concerns | Low risk | No concerns | Major concerns | No concerns | No concerns | Very low |
| BGT:Tango | 0 | Some concerns | Low risk | No concerns | Major concerns | No concerns | No concerns | Very low |
| BGT:TC | 1 | Some concerns | Low risk | No concerns | Major concerns | No concerns | No concerns | Very low |
| BGT:TT | 0 | No concerns | Low risk | No concerns | Major concerns | No concerns | No concerns | Low |
| BGT:VR | 5 | No concerns | Low risk | No concerns | Major concerns | No concerns | No concerns | Low |
| BGT:WBV | 0 | Some concerns | Low risk | No concerns | Major concerns | No concerns | No concerns | Very low |
| BGT:Yoga | 2 | Some concerns | Low risk | No concerns | Major concerns | No concerns | No concerns | Very low |
| BGT_ECA:BGT_ICA | 1 | Some concerns | Low risk | No concerns | Major concerns | No concerns | No concerns | Very low |
| BGT_ECA:BWS_TT | 0 | No concerns | Low risk | No concerns | No concerns | Major concerns | No concerns | Low |
| BGT_ECA:CON | 3 | No concerns | Low risk | No concerns | No concerns | No concerns | No concerns | High |
| BGT_ECA:CPP | 1 | No concerns | Low risk | No concerns | No concerns | No concerns | No concerns | High |
| BGT_ECA:Dance | 0 | Some concerns | Low risk | No concerns | Major concerns | No concerns | No concerns | Very low |
| BGT_ECA:DT_BGT | 0 | Some concerns | Low risk | No concerns | Major concerns | No concerns | No concerns | Very low |
| BGT_ECA:Mul_C | 0 | No concerns | Low risk | No concerns | Major concerns | No concerns | No concerns | Low |
| BGT_ECA:Mul_D | 1 | No concerns | Low risk | No concerns | Major concerns | No concerns | No concerns | Low |
| BGT_ECA:NW | 0 | No concerns | Low risk | No concerns | Major concerns | No concerns | No concerns | Low |
| BGT_ECA:Pilates | 0 | No concerns | Low risk | No concerns | Major concerns | No concerns | No concerns | Low |
| BGT_ECA:PT | 0 | Some concerns | Low risk | No concerns | Major concerns | No concerns | No concerns | Very low |
| BGT_ECA:Qigong | 0 | Some concerns | Low risk | No concerns | Major concerns | No concerns | No concerns | Very low |
| BGT_ECA:RA_GT | 0 | Some concerns | Low risk | No concerns | Major concerns | No concerns | No concerns | Very low |
| BGT_ECA:RT | 0 | No concerns | Low risk | No concerns | No concerns | Major concerns | No concerns | Low |
| BGT_ECA:Stretch | 0 | Some concerns | Low risk | No concerns | No concerns | No concerns | No concerns | Moderate |
| BGT_ECA:Tango | 0 | Some concerns | Low risk | No concerns | Major concerns | No concerns | No concerns | Very low |
| BGT_ECA:TC | 0 | Some concerns | Low risk | No concerns | Major concerns | No concerns | No concerns | Very low |
| BGT_ECA:TT | 2 | No concerns | Low risk | No concerns | Major concerns | No concerns | No concerns | Low |
| BGT_ECA:VR | 0 | No concerns | Low risk | No concerns | Major concerns | No concerns | No concerns | Low |
| BGT_ECA:WBV | 0 | Some concerns | Low risk | No concerns | Major concerns | No concerns | No concerns | Very low |
| BGT_ECA:Yoga | 0 | Some concerns | Low risk | No concerns | Major concerns | No concerns | No concerns | Very low |
| BGT_ICA:BWS_TT | 0 | Some concerns | Low risk | No concerns | Major concerns | No concerns | No concerns | Very low |
| BGT_ICA:CON | 1 | Some concerns | Low risk | No concerns | Major concerns | No concerns | No concerns | Very low |
| BGT_ICA:CPP | 0 | Some concerns | Low risk | No concerns | Major concerns | No concerns | No concerns | Very low |
| BGT_ICA:Dance | 0 | Some concerns | Low risk | No concerns | Major concerns | No concerns | No concerns | Very low |
| BGT_ICA:DT_BGT | 0 | Some concerns | Low risk | No concerns | Major concerns | No concerns | No concerns | Very low |
| BGT_ICA:Mul_C | 0 | Some concerns | Low risk | No concerns | Major concerns | No concerns | No concerns | Very low |
| BGT_ICA:Mul_D | 0 | Some concerns | Low risk | No concerns | Major concerns | No concerns | No concerns | Very low |
| BGT_ICA:NW | 0 | Some concerns | Low risk | No concerns | Major concerns | No concerns | No concerns | Very low |
| BGT_ICA:Pilates | 0 | Some concerns | Low risk | No concerns | Major concerns | No concerns | No concerns | Very low |
| BGT_ICA:PT | 0 | Some concerns | Low risk | No concerns | Major concerns | No concerns | No concerns | Very low |
| BGT_ICA:Qigong | 0 | Some concerns | Low risk | No concerns | Major concerns | No concerns | No concerns | Very low |
| BGT_ICA:RA_GT | 0 | Some concerns | Low risk | No concerns | Major concerns | No concerns | No concerns | Very low |
| BGT_ICA:RT | 0 | Some concerns | Low risk | No concerns | Major concerns | No concerns | No concerns | Very low |
| BGT_ICA:Stretch | 0 | Some concerns | Low risk | No concerns | Major concerns | No concerns | No concerns | Very low |
| BGT_ICA:Tango | 0 | Some concerns | Low risk | No concerns | Major concerns | No concerns | No concerns | Very low |
| BGT_ICA:TC | 0 | Some concerns | Low risk | No concerns | Major concerns | No concerns | No concerns | Very low |
| BGT_ICA:TT | 0 | Some concerns | Low risk | No concerns | Major concerns | No concerns | No concerns | Very low |
| BGT_ICA:VR | 0 | Some concerns | Low risk | No concerns | Major concerns | No concerns | No concerns | Very low |
| BGT_ICA:WBV | 0 | Some concerns | Low risk | No concerns | Major concerns | No concerns | No concerns | Very low |
| BGT_ICA:Yoga | 0 | Some concerns | Low risk | No concerns | Major concerns | No concerns | No concerns | Very low |
| BWS_TT:CON | 0 | No concerns | Low risk | No concerns | No concerns | No concerns | No concerns | High |
| BWS_TT:CPP | 0 | No concerns | Low risk | No concerns | No concerns | No concerns | No concerns | High |
| BWS_TT:Dance | 0 | No concerns | Low risk | No concerns | Major concerns | No concerns | No concerns | Low |
| BWS_TT:DT_BGT | 0 | No concerns | Low risk | No concerns | No concerns | Major concerns | No concerns | Low |
| BWS_TT:Mul_C | 0 | No concerns | Low risk | No concerns | No concerns | No concerns | No concerns | High |
| BWS_TT:Mul_D | 0 | No concerns | Low risk | No concerns | Major concerns | No concerns | No concerns | Low |
| BWS_TT:NW | 0 | No concerns | Low risk | No concerns | Major concerns | No concerns | No concerns | Low |
| BWS_TT:Pilates | 0 | No concerns | Low risk | No concerns | Major concerns | No concerns | No concerns | Low |
| BWS_TT:PT | 0 | Some concerns | Low risk | No concerns | Major concerns | No concerns | No concerns | Very low |
| BWS_TT:Qigong | 0 | Some concerns | Low risk | No concerns | No concerns | No concerns | No concerns | Moderate |
| BWS_TT:RA_GT | 0 | No concerns | Low risk | No concerns | Major concerns | No concerns | No concerns | Low |
| BWS_TT:RT | 0 | No concerns | Low risk | No concerns | No concerns | No concerns | No concerns | High |
| BWS_TT:Stretch | 0 | No concerns | Low risk | No concerns | No concerns | No concerns | No concerns | High |
| BWS_TT:Tango | 0 | No concerns | Low risk | No concerns | No concerns | No concerns | No concerns | High |
| BWS_TT:TC | 0 | No concerns | Low risk | No concerns | No concerns | No concerns | No concerns | High |
| BWS_TT:TT | 2 | No concerns | Low risk | No concerns | No concerns | No concerns | No concerns | High |
| BWS_TT:VR | 0 | No concerns | Low risk | No concerns | No concerns | No concerns | No concerns | High |
| BWS_TT:WBV | 0 | Some concerns | Low risk | No concerns | Major concerns | No concerns | No concerns | Very low |
| BWS_TT:Yoga | 0 | No concerns | Low risk | No concerns | No concerns | No concerns | No concerns | High |
| CON:CPP | 1 | Some concerns | Low risk | No concerns | Major concerns | No concerns | No concerns | Very low |
| CON:Dance | 4 | Some concerns | Low risk | No concerns | No concerns | No concerns | No concerns | Moderate |
| CON:DT_BGT | 5 | Some concerns | Low risk | No concerns | No concerns | No concerns | No concerns | Moderate |
| CON:Mul_C | 7 | Some concerns | Low risk | No concerns | No concerns | Major concerns | No concerns | Very low |
| CON:Mul_D | 1 | No concerns | Low risk | No concerns | No concerns | No concerns | No concerns | High |
| CON:NW | 1 | Some concerns | Low risk | No concerns | No concerns | No concerns | No concerns | Moderate |
| CON:Pilates | 1 | Some concerns | Low risk | No concerns | No concerns | No concerns | No concerns | Moderate |
| CON:PT | 1 | Some concerns | Low risk | No concerns | Major concerns | No concerns | No concerns | Very low |
| CON:Qigong | 2 | Some concerns | Low risk | No concerns | Major concerns | No concerns | No concerns | Very low |
| CON:RA_GT | 0 | Some concerns | Low risk | No concerns | No concerns | No concerns | No concerns | Moderate |
| CON:RT | 4 | No concerns | Low risk | No concerns | No concerns | Major concerns | No concerns | Low |
| CON:Stretch | 0 | Some concerns | Low risk | No concerns | Major concerns | No concerns | No concerns | Very low |
| CON:Tango | 3 | Some concerns | Low risk | No concerns | No concerns | Major concerns | No concerns | Very low |
| CON:TC | 2 | Some concerns | Low risk | No concerns | No concerns | Major concerns | No concerns | Very low |
| CON:TT | 1 | Some concerns | Low risk | No concerns | No concerns | Major concerns | No concerns | Very low |
| CON:VR | 1 | No concerns | Low risk | No concerns | No concerns | Major concerns | No concerns | Low |
| CON:WBV | 0 | Some concerns | Low risk | No concerns | Major concerns | No concerns | No concerns | Very low |
| CON:Yoga | 3 | Some concerns | Low risk | No concerns | Major concerns | No concerns | No concerns | Very low |
| CPP:Dance | 2 | No concerns | Low risk | No concerns | No concerns | No concerns | No concerns | High |
| CPP:DT_BGT | 0 | Some concerns | Low risk | No concerns | No concerns | Major concerns | No concerns | Very low |
| CPP:Mul_C | 2 | Some concerns | Low risk | No concerns | No concerns | Major concerns | No concerns | Very low |
| CPP:Mul_D | 1 | No concerns | Low risk | No concerns | No concerns | No concerns | No concerns | High |
| CPP:NW | 0 | No concerns | Low risk | No concerns | No concerns | Major concerns | No concerns | Low |
| CPP:Pilates | 0 | Some concerns | Low risk | No concerns | No concerns | No concerns | No concerns | Moderate |
| CPP:PT | 0 | Some concerns | Low risk | No concerns | Major concerns | No concerns | No concerns | Very low |
| CPP:Qigong | 1 | Some concerns | Low risk | No concerns | Major concerns | No concerns | No concerns | Very low |
| CPP:RA_GT | 0 | Some concerns | Low risk | No concerns | No concerns | Major concerns | No concerns | Very low |
| CPP:RT | 2 | No concerns | Low risk | No concerns | Major concerns | No concerns | No concerns | Low |
| CPP:Stretch | 0 | Some concerns | Low risk | No concerns | Major concerns | No concerns | No concerns | Very low |
| CPP:Tango | 0 | Some concerns | Low risk | No concerns | No concerns | Major concerns | No concerns | Very low |
| CPP:TC | 0 | Some concerns | Low risk | No concerns | Major concerns | No concerns | No concerns | Very low |
| CPP:TT | 0 | Some concerns | Low risk | No concerns | Major concerns | No concerns | No concerns | Very low |
| CPP:VR | 2 | Some concerns | Low risk | No concerns | No concerns | Major concerns | No concerns | Very low |
| CPP:WBV | 0 | Some concerns | Low risk | No concerns | Major concerns | No concerns | No concerns | Very low |
| CPP:Yoga | 0 | Some concerns | Low risk | No concerns | Major concerns | No concerns | No concerns | Very low |
| Dance:DT_BGT | 0 | Some concerns | Low risk | No concerns | Major concerns | No concerns | No concerns | Very low |
| Dance:Mul_C | 0 | Some concerns | Low risk | No concerns | Major concerns | No concerns | No concerns | Very low |
| Dance:Mul_D | 0 | No concerns | Low risk | No concerns | Major concerns | No concerns | No concerns | Low |
| Dance:NW | 0 | Some concerns | Low risk | No concerns | Major concerns | No concerns | No concerns | Very low |
| Dance:Pilates | 0 | Some concerns | Low risk | No concerns | Major concerns | No concerns | No concerns | Very low |
| Dance:PT | 0 | Some concerns | Low risk | No concerns | Major concerns | No concerns | No concerns | Very low |
| Dance:Qigong | 0 | Some concerns | Low risk | No concerns | Major concerns | No concerns | No concerns | Very low |
| Dance:RA_GT | 0 | Some concerns | Low risk | No concerns | Major concerns | No concerns | No concerns | Very low |
| Dance:RT | 0 | No concerns | Low risk | No concerns | Major concerns | No concerns | No concerns | Low |
| Dance:Stretch | 0 | Some concerns | Low risk | No concerns | No concerns | No concerns | No concerns | Moderate |
| Dance:Tango | 2 | Some concerns | Low risk | No concerns | Major concerns | No concerns | No concerns | Very low |
| Dance:TC | 0 | Some concerns | Low risk | No concerns | Major concerns | No concerns | No concerns | Very low |
| Dance:TT | 0 | Some concerns | Low risk | No concerns | Major concerns | No concerns | No concerns | Very low |
| Dance:VR | 0 | Some concerns | Low risk | No concerns | Major concerns | No concerns | No concerns | Very low |
| Dance:WBV | 0 | Some concerns | Low risk | No concerns | Major concerns | No concerns | No concerns | Very low |
| Dance:Yoga | 0 | Some concerns | Low risk | No concerns | Major concerns | No concerns | No concerns | Very low |
| DT_BGT:Mul_C | 0 | Some concerns | Low risk | No concerns | Major concerns | No concerns | No concerns | Very low |
| DT_BGT:Mul_D | 0 | Some concerns | Low risk | No concerns | Major concerns | No concerns | No concerns | Very low |
| DT_BGT:NW | 0 | Some concerns | Low risk | No concerns | Major concerns | No concerns | No concerns | Very low |
| DT_BGT:Pilates | 0 | Some concerns | Low risk | No concerns | Major concerns | No concerns | No concerns | Very low |
| DT_BGT:PT | 0 | Some concerns | Low risk | No concerns | Major concerns | No concerns | No concerns | Very low |
| DT_BGT:Qigong | 0 | Some concerns | Low risk | No concerns | Major concerns | No concerns | No concerns | Very low |
| DT_BGT:RA_GT | 1 | Some concerns | Low risk | No concerns | Major concerns | No concerns | No concerns | Very low |
| DT_BGT:RT | 0 | Some concerns | Low risk | No concerns | Major concerns | No concerns | No concerns | Very low |
| DT_BGT:Stretch | 2 | Some concerns | Low risk | No concerns | No concerns | No concerns | Major concerns | Very low |
| DT_BGT:Tango | 0 | Some concerns | Low risk | No concerns | Major concerns | No concerns | No concerns | Very low |
| DT_BGT:TC | 0 | Some concerns | Low risk | No concerns | Major concerns | No concerns | No concerns | Very low |
| DT_BGT:TT | 1 | Some concerns | Low risk | No concerns | Major concerns | No concerns | No concerns | Very low |
| DT_BGT:VR | 1 | Some concerns | Low risk | No concerns | Major concerns | No concerns | No concerns | Very low |
| DT_BGT:WBV | 1 | Some concerns | Low risk | No concerns | Major concerns | No concerns | No concerns | Very low |
[truncated: 106,059 more chars]
